# Integrated proteogenomic approach identifying a protein signature of COPD and a new splice variant of SORBS1

**DOI:** 10.1136/thoraxjnl-2019-213200

**Published:** 2020-01-14

**Authors:** Corry-Anke Brandsma, Victor Guryev, Wim Timens, Ana Ciconelle, Dirkje S Postma, Rainer Bischoff, Maria Johansson, Ekaterina S Ovchinnikova, Johan Malm, Gyorgy Marko-Varga, Thomas E Fehniger, Maarten van den Berge, Peter Horvatovich

**Affiliations:** 1 Department of Pathology and Medical Biology, University of Groningen, University Medical Center Groningen, Groningen, the Netherlands; 2 University of Groningen, University Medical Center Groningen, Groningen Research Institute for Asthma and COPD, Groningen, the Netherlands; 3 European Research Institute for the Biology of Ageing, University Medical Center Groningen, Groningen, the Netherlands; 4 Department of Analytical Biochemistry, University of Groningen, Groningen Research Institute of Pharmacy, Groningen, the Netherlands; 5 Department of Pulmonary Diseases, University of Groningen, University Medical Center Groningen, Groningen, the Netherlands; 6 Center of Excellence in Biological and Medical Mass Spectrometry, Biomedical Center, Lund University, Lund, Sweden; 7 Department of Cardiology, University of Groningen, University Medical Center Groningen, Groningen, the Netherlands; 8 Department of Translational Medicine, Lund University, Malmö, Sweden

**Keywords:** COPD ÀÜ mechanisms, COPD pathology

## Abstract

Translation of genomic alterations to protein changes in chronic obstructive pulmonary disease (COPD) is largely unexplored. Using integrated proteomic and RNA sequencing analysis of COPD and control lung tissues, we identified a protein signature in COPD characterised by extracellular matrix changes and a potential regulatory role for SUMO2. Furthermore, we identified 61 differentially expressed novel, non-reference, peptides in COPD compared with control lungs. This included two peptides encoding for a new splice variant of SORBS1, of which the transcript usage was higher in COPD compared with control lungs. These explorative findings and integrative proteogenomic approach open new avenues to further unravel the pathology of COPD.

## Introduction

Chronic obstructive pulmonary disease (COPD) has a high burden and rising mortality, with no curative treatment available. COPD is driven by a complex interaction between genetic and environmental factors. Genome-wide association studies have shown that multiple single nucleotide polymorphisms are associated with COPD and have improved our insight into disease aetiology. The functional translation of these findings is an emerging field. In particular, the translation of genomic alterations to protein changes is important, since proteins are the biologically active molecules that reflect actual disease pathology. Hence, the vast majority of factors that contribute to the phenotypic profile of COPD initiation and progression lies within the proteome.

Proteomics is a rapidly developing area, also in the clinical setting, with, for example, the Cancer Moonshot initiative in precision oncology.^1^ Mass spectrometry-based ‘shotgun’ proteomics is currently the most powerful, high-throughput technique enabling quantification and identification of several tens of thousands of peptides and several thousands of proteins in complex biological samples.^2 3^ Recently, this method proved to be successful in fibrotic lung and skin samples.^4^

## Methods

Here, we report on the first explorative study using an integrative proteogenomic approach to study pathogenetic changes in Stage IV COPD (n=10) compared with control (n=8) lung tissue (all ex-smokers, table 1). With this proteogenomics approach,^5^ we integrated mass spectrometry-based proteomic and RNA-sequencing data of polyadenylated transcripts of the same frozen lung tissue samples that were stored at −80°C, of which consecutive slides (10×10 µm) were cut and used for RNA and protein isolation (detailed methods in online supplementary files). The most important step in this integrated approach was the prediction of the protein sequence variants present in each sample based on the RNA-sequencing data, creating sample-specific protein reference databases. These protein reference databases were used for peptide and protein identification and quantification, allowing identification of patient-specific non-synonymous variants (including splice variants) and new transcript isoforms. Raw spectral counts (ie, number of peptide-spectrum match or PSMs) were calculated for (1) peptides uniquely mapping to Ensembl genes and (2) non-mapping, that is, non-reference, peptides based on the sample-specific protein reference databases. Proteomics and RNAseq data were normalised using upper quartile normalisation. The proteogenomics workflow and principal component analysis are shown in online supplementary figures S1 and S2 (data access via ArrayExpress E-MTAB-8251, scripts are available on request).

10.1136/thoraxjnl-2019-213200.supp1 Supplementary data

**Table 1:** Clinical characteristics of patients with COPD and controls

	Control	COPD stage IV
Number	8	10
Age, years	65 (7)*	58 (2)*
Sex (m/f)	4/4	2/8
Pack-years smoking	34 (17)†	40 (12)
FEV_1_%pred	95 (11)†	21 (4)
FEV_1_/FVC %	76 (4)	31 (10)

Mean (SD).

*P<0.05 control vs COPD.

†No information available w.r.t pack-years in one control and FEV_1_%pred in two controls.

FEV_1_, forced expiratory volume in one second; FVC, forced vital capacity.

## Results and discussion

**Figure 1:**
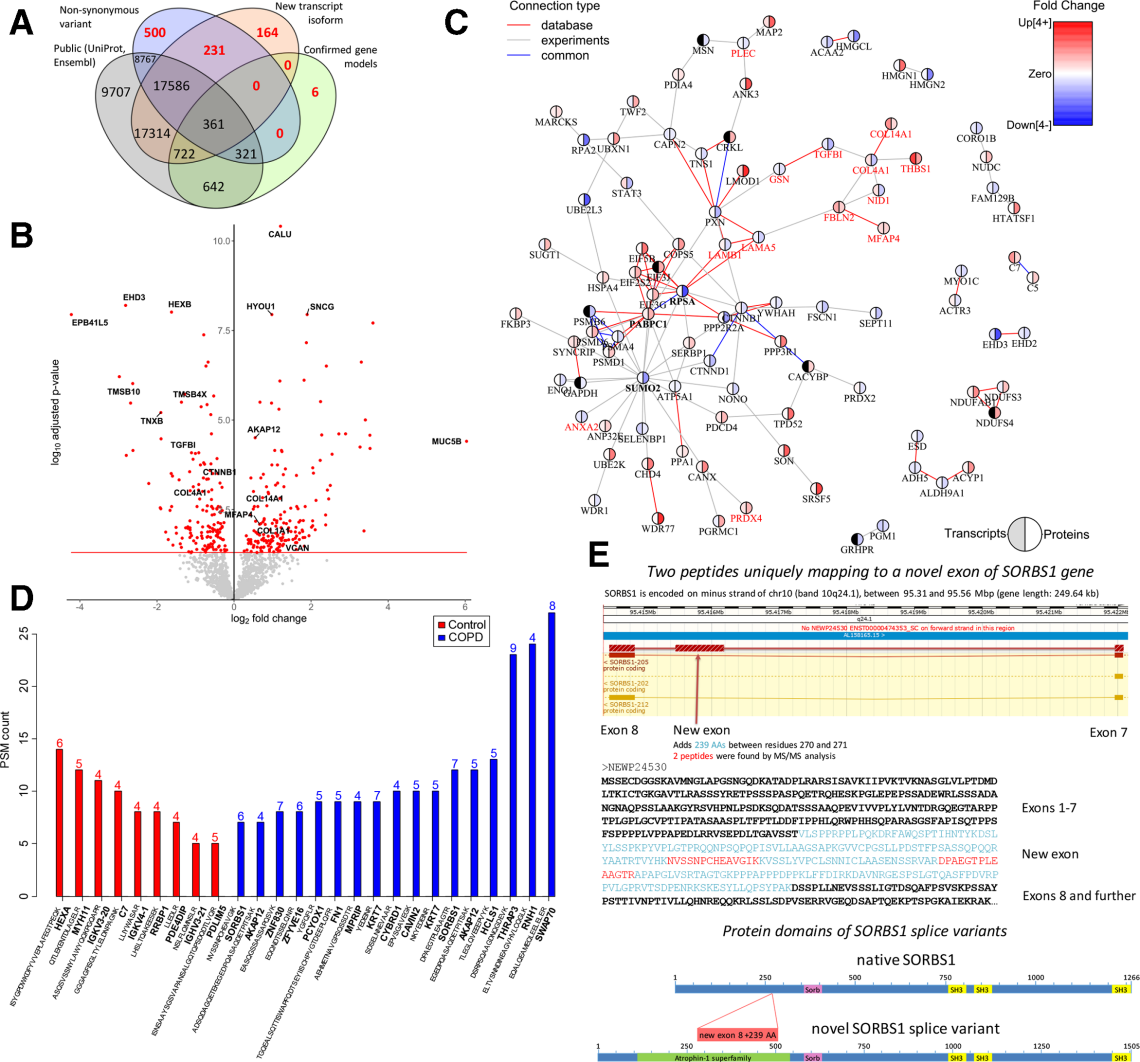
(A) Venn diagram showing the total number of identified peptides that mapped to canonical sequences in the Uniprot and Ensembl public databases (normal text) and non-reference sequences (red bold text), which included non-synonymous variants (single amino acid variants), new transcript isoforms and confirmed gene models. (B) Volcano plot of all proteins consistently expressed in COPD and control lung tissue. Differentially expressed proteins (FDR<0.05) are in red. (C) STRING protein-protein interaction network based on differential protein expression in severe COPD using an FDR<0.01 cut off. Red connections show known protein-protein interactions from databases, grey connections represent experimentally-derived protein-protein interactions and blue connections are common database and experimentally derived interactions. Pie charts express the fold change at the transcript (left) and protein (right) level in severe COPD. The direction and fold change is indicated in blue (downregulated) and red (upregulated). The genes related to the extracellular matrix organisation gene ontology are highlighted in red. (D) Number of MS/MS spectra (PSMs) attributed to non-reference sample specific peptides that were exclusively identified in severe COPD and control lung tissue. Only peptides with at least five PSMs and present in at least four patients with COPD or controls were considered. The number of samples where the non-reference peptide was identified is indicated at the top of each bar. (E) Upper plot shows the genomic region of the new exon that was identified in the human SORBS1 gene. The arrow indicates the location of an additional exon corresponding to 238 amino acid residues. SORBS1 is encoded on minus strand of chr10 (band 10q24.1) between 95.31 and 95.56 Mbp (gene length: 249.64 kb). The lower plot shows the amino acid sequence of the new SORBS1 splice variant highlighting the additional novel exon (upper-case light-blue) and the two peptides identified by mass spectrometry (red). PSM, peptide-spectrum match.

This integrative approach resulted in the identification of 56 322 peptides, including 901 novel, non-reference peptides that would not have been identified without the RNAseq integration (figure 1A). These 56 322 peptides mapped to 1724 proteins that were expressed with ≥3 PSMs in at least 5 patients with COPD or four controls. Among these 1724 proteins, we identified 177 upregulated and 150 downregulated proteins in COPD compared with control lung tissue, with calumenin (CALU), synuclein gamma (SNCG) and hypoxia upregulated 1 (HYOU1) being the most significantly upregulated, and EH domain containing protein 3 (EHD3), hexosaminidase subunit beta (HEXB) and erythrocyte membrane protein band 4.1 like 5 (EPB41L5) being the most significantly downregulated proteins (false discovery rate (FDR)<0.05, figure 1B, online supplementary figure S3, online supplementary table S1). Examples of four upregulated and four downregulated proteins, including the marginal zone B and B1 cell-specific protein (MZB1), are plotted in online supplementary figure S4. MZB1 is a marker of IgG-producing plasma cells that was recently identified as upregulated in fibrotic lung tissue.^4^ Of the 327 differentially-expressed proteins, 37 showed differential transcript expression in the same direction, including MZB1 and several extracellular matrix (ECM) proteins (p<0.05, online supplementary figure S5). The volcano plot of the 226 upregulated and 124 downregulated transcripts (FDR<0.05) is shown in online supplementary figure S6. Our transcript findings were in high agreement with an independent lung tissue RNA-sequencing dataset (online supplementary figure S7).^6^

Enrichment analysis of differentially expressed proteins demonstrated enrichment of gene ontologies related to ECM and structure organisation (FDR p-value=1.05×10^–4^, online supplementary table S2). The STRING protein interaction network based on differential protein expression indicated a central role for small ubiquitin-related modifier 2 (SUMO2) with 19 connections (figure 1C). Online supplementary figure S8 demonstrates higher connectivity of the edges in our proteomics dataset compared with the entire STRING database. SUMO2 belongs to the group of ubiquitin-like modifiers, which can target proteins in a similar manner to ubiquitination.^7 8^ Conjugation of SUMO2/3 to protein targets is induced by various stressors (eg, oxidative stress). As cells contain a large pool of unconjugated SUMO2/3,^9^ it has been proposed that one function of SUMO2/3 is to provide a pool of free SUMO to respond to stress.^7^ Thus, the identification of SUMO2 may suggest a role in attenuating oxidative stress in COPD.

Of the 901 identified non-reference peptides, 17 and 9 were only identified in COPD and control lung tissue, respectively (figure 1D, online supplementary table S3). In addition, 35 non-reference peptides were differentially expressed between COPD and control (online supplementary figure S9). The majority of these peptides were single amino acid variants caused by non-synonymous variants (online supplementary table S4) and 10 mapped to immunoglobulin proteins (online supplementary table S5), indicating changes in the specific immune response between COPD and control, which aligns with our previous observations^10^ and the changes in MZB1.

Interestingly, we identified two peptides that were only present in the COPD samples that mapped uniquely to an unknown splice variant of SORBS1 (sorbin and SH3 domain containing 1, figure 1E). SORBS1 is an adaptor protein involved in insulin signalling. Polymorphisms in the SORBS1 gene have been associated with various, non-lung related, diseases,^11 12^ but its role in lung disease is unexplored. The new SORBS1 splice variant includes an additional exon encoding for an atrophin-1 domain. Atrophin-1 is a transcriptional regulator associated with the polyglutamine disease DRPLA (Dentatorubral-pallidoluysian atrophy).^13^ Nothing is known yet on the function of atrophin-1 in lung; however, given its function as a transcriptional regulator, it is possibly affecting the transcription of SORBS1. Whereas these sequence variants were detected at the peptide level only in the COPD samples, transcripts were detected in both COPD and control samples. In an independent RNA-sequencing dataset (n=189),^6^ we demonstrated that the usage of this new exon for SORBS1 was significantly higher in COPD compared with control lung tissue (Mann-Whitney U test p=0.003, online supplementary figure S10).

The MS/MS spectra of the differentially expressed non-reference peptides (online supplementary file 1.6), the confirmation of these findings using ion count label-free quantification (online supplementary figures S11 and S12) and confirmation with synthetic peptides (online supplementary file 1.7) is shown in the online supplementary file. Although COPD samples were derived from lung transplantation and control samples from tumour resection surgery, tissue sample processing and storage were similar. Although the (histologically normal) lung tissue was taken far from the tumour, a tumour effect cannot be excluded, but considering tumour heterogeneity, this would rather have precluded than induced positive findings.

## Conclusion

In summary, our protein signature in COPD confirmed important ECM protein changes in COPD, identified SUMO2 as a potential regulatory protein, and resulted in the identification of a new splice variant of SORBS1. Although our study used a small, albeit homogenous, subset of samples from end-stage COPD, a comprehensive lung tissue protein signature was identified that was in part also apparent at the transcript level. Our study was hypothesis-generating and, given the small samples size, future studies are needed to further validate and extend our findings in a larger and independent cohort.

Taken together, our findings and our integrative approach provide promising new avenues to further unravel the molecular mechanisms of COPD pathology, which may have important implications for future patient care.

10.1136/thoraxjnl-2019-213200.supp2 Supplementary data

10.1136/thoraxjnl-2019-213200.supp3 Supplementary data
