# Supplementary material for: Integrated proteogenomic approach identifying a protein signature of COPD and a new splice variant of SORBS1
Source: Thorax. 2020 Jan 14;75(2):180–3. doi: 10.1136/thoraxjnl-2019-213200 (PMC7029225; doi:10.1136/thoraxjnl-2019-213200)
Supplement: Supplementary data [file thoraxjnl-2019-213200supp001.pdf]

Supporting information

An integrated proteogenomic approach identifying a protein signature of COPD and a new splice variant of SORBS1

C.A. Brandsma, V. Guryev, W. Timens, A. Ciconelle, D.S. Postma, R. Bischoff, M. Johansson, E.S. Ovchinnikova, J. Malm, G. Marko-Varga, T.E. Fehniger, M. van den Berge, P. Horvatovich.

Table of content of supporting information

1 DETAILED MATERIAL AND METHODS 1

1.1 LUNG TISSUE SAMPLES 1
1.2 SAMPLE PREPARATION OF FROZEN HUMAN LUNG TISSUE SAMPLES FOR PROTEOMICS ANALYSIS 2
1.3 TRANSCRIPTOMICS ANALYSIS 2
1.4 LYSIS, PROTEIN EXTRACTION AND REDUCTION ALKYLATION 2
1.5 BUFFER EXCHANGE AND TRYPSIN DIGESTION 2
1.6 FRACTIONATION (SCX) AND DESALTING 3
1.7 DATA-DEPENDENT LC-MS/MS ANALYSIS 3
1.8 DATA PRE-PROCESSING, PEPTIDE AND PROTEIN IDENTIFICATION 4
1.9 STATISTICAL ANALYSIS AND PROTEOGENOMICS DATA INTEGRATION 5
1.10 DATA FILES. 6

2 SUPPORTING INFORMATION FIGURES 7

3 SUPPORTING INFORMATION TABLES 20

4 REFERENCES 24

5 MS/MS SPECTRA AND IDENTIFICATION PARAMETERS OF 61 NON-REFERENCE PEPTIDES 26

6 MS/MS SPECTRA VISUALISATION OF SYNTHETIC AND ENDOGENOUS 23 NON-REFERENCE PEPTIDES PRESENT ONLY ON COPD OR CONTROL SAMPLES 87

1 Detailed material and methods

1.1 Lung tissue samples

Left-over frozen lung tissue from 10 ex-smoker stage IV COPD patients and 8 ex-smoker non-COPD controls was used. All subjects were Caucasian. All samples were obtained from peripheral lung tissue according to national and local ethical guidelines and the research code of the UMCG [1]. Non-COPD control (referred throughout as 'control') was defined with lung function FEV1/FVC ≥ 70% and FEV1 >90% predicted. Subjects in this category did not have a history of lung disease, apart from lung cancer for which the patients underwent surgery. For these cases, lung tissue samples were taken as distant from the tumor as possible. Thus, any possible effect of the tumor on the lung tissue was minimised. All COPD patients suffered from severe emphysema and underwent lung transplantation.

Brandsma C-A, *et al. Thorax* 2020; 75:180–183. doi: 10.1136/thoraxjnl-2019-213200

1.2 Sample preparation of frozen human lung tissue samples for proteomics analysis

All chemicals were from Sigma Aldrich (St. Louis, Missouri, US) if not stated otherwise.

1.3 Transcriptomics analysis

Total RNA from parenchymal lung samples was isolated using TRIzol Reagent (Ambion, Foster City, CA), according to manufacturer's instructions. We extracted PolyA-tailed mRNA fraction from total RNA and prepared RNA-seq libraries using NEXTFlex Rapid Directional qRNA-Seq Kit (Bioo Scientific, Austin, TX) according to the manufacturer's protocol. Libraries were pooled and sequenced on HiSeq2500 sequencer (Illumina, San Diego, CA) using paired-end 2×110bp mode. Quality of resulting reads was evaluated by FastQC software (https://www.bioinformatics.babraham.ac.uk/projects/fastqc/). Unique molecular identifiers (UMIs) and flanking T base were trimmed using custom Perl script. Quality/adaptor trimming was performed using Trimmomatic software v. 0.33[2]. Trimmed reads were mapped to reference genome GRCh38 either by STAR program v. 2.4.1d[3] or by TopHat2 (version 2.0.13)[4] using ensembl gene annotation release 80 (http://www.ensembl.org). The reason to use two reference genome alignment approaches is that the outcomes of TopHat2 and STAR aligner were significantly different. In the absence of the "ground truth for alignment" and in order to present the most complete set of transcriptome isoforms for peptide identification, we have used both alignment methods. Duplicate reads (reads mapping to the same genomic positions and having same combination of UMIs) were excluded using custom Perl script. Reads were quantified using HTSeq program, v. 0.6.1p1[5]. Transcript assembly was performed from alignments using StringTie v. 1.3.3[6] and Trinity v. 2.0.0[7]. The longest open reading frames of new transcripts were predicted using custom Perl script (all scripts are available upon request).

We used the BCFtools program (http://htslib.org) v. 0.1.17 to call variants on the combined set of RNA-seq alignments. Only high quality variants were retained (variant quality >50). They were annotated using snpEff tool (http://snpeff.sourceforge.net, version 4.1b) using gene annotation from aforementioned Ensembl release. Variants that change amino acid sequences were extracted and applied to reference protein sequences using a custom perl script. The resulting protein isoforms affected by non-synonymous variants were added to reference protein database.

Based on the Ensembl transcript annotation, RNA sequencing generated a total of 32,435 predicted translated non-redundant protein sequences (18,624-23,780 per sample, Table S6). These corresponded to 15,708 human protein-coding genes with detectable expression level in our samples and used for differential transcript expression analysis.

1.4 Lysis, protein extraction and reduction alkylation

Frozen lung tissue samples were lysed in 6 M urea and 50 mM ammonium bicarbonate buffer for 30 min on ice using thorough mixing. Samples were centrifuged at 10,000 g for 10 min and supernatants transferred into a new vial. Pierce BCA kit was used for protein determination. To reduce cysteine residues 10 mM DTT was added (1 h at 60 °C) followed by 50 mM iodoacetamide (30 min at room temperature in the dark) to disrupt native disulphide bonds and prevent their formation. Finally, 0.02 µg/µL chicken lysozyme was added to the extracts (internal control)[8].

1.5 Buffer exchange and trypsin digestion

Hundred microliters of each sample was filtered on Amicon Ultra centrifuge filters (0.5 mL volume with 10 kDa filter cutoff, Millipore, Dublin, Ireland). Samples were washed three times in 50 mM

Brandsma C-A, *et al. Thorax* 2020; 75:180–183. doi: 10.1136/thoraxjnl-2019-213200

ammonium bicarbonate buffer (14,000 g for 20 minutes). The proteins remained on the filter were washed off with 50 mM ammonium bicarbonate buffer with centrifugation (3 minutes, 1,000 g). Trypsin digestion was performed overnight at 37˚C after addition of 10 µL trypsin (0.1 µg/µL, Promega, Madison, Wisconsin, US) having 1:100 protein:enzyme ratio (w/w). The digestion was stopped with 24 µL 10% formic acid (FA) and the samples were dried in a vacuum concentrator at room temperature for 9 hours. The samples were dissolved in 100 µL water/0.1% FA providing a final protein concentration of 1 µg/µL. The digests were centrifuged (5 minutes, 10,000×g) and 20 µL of peptide retention time calibration mixture (PRTC, product # 88320, Pierce, Rockford, Illinois, US) was added to each sample (25 fmoL/µL).

1.6 Fractionation (SCX) and desalting

Fifty microliters of each protein digest was dried in a vacuum concentrator, and the samples was dissolved in 10 mM KH_2PO_4/20% acetonitrile (ACN). Strong cation exchange (SCX) columns (SEM HIL-SCX, 10-100 µg capacity, The Nest group Inc., SouthBorough, Massachusetts, USA) were used for fractionation. Each column was washed with (1) 100 µL MeOH, 100 µL Milli-Q water, (2) 100 µL 50:50 of 10 mM KH_2PO_4/20% ACN and (3) 1 M KCl and 10 mM KH_2PO_4/20% ACN at pH 2.8. Each washing step was followed by centrifugation (1 minute, 110 × g). The columns were equilibrated and washed twice with 100 µL 10 mM KH_2PO_4/20% ACN at pH 2.8 (1 minute, 200 × g). Fifty microliters of each sample was loaded onto the column, centrifuged (1 minute, 110 × g), and the flow-through (FT) was collected. The columns were then washed with 100 µL 10 mM KH_2PO_4/20%ACN, and the eluent was combined with the FT of the previous step. Six fractions were collected using stepwise gradient of 20 (pooled with combined FT), 40, 60, 100, 500 and 1,000 mM KCl buffer resulting in ~8.3 µg peptide per fraction. All fractions were dried in a vacuum concentrator and resuspended in 100 µL 0.1% TFA in water. The resuspended solutions were desalted on C18 spin columns.

C18 spin columns (UltraMicroSpin Column C18, SUM SS18V, capacity 3-30 µg, The Nest group Inc., South Borough, Massachusetts, USA) for desalting were conditioned with 100 µL ACN/0.1% TFA and centrifugation (1 minute, 110 × g). The spin columns were then flushed twice with 100 µL 0.1% TFA and centrifuged (1 minute, 110 × g). Fractions were loaded onto separate C18 spin columns and centrifuged (1 minute, 200 × g), and washed twice with 100 µL of 0.1% TFA (1 minute, 200 × g). The desalted peptides were eluted with 2 × 50 µL of 50% ACN/0.1% TFA (1 minute, 200 × g), and dried in a vacuum concentrator. The dried fractions were resolved in 17 µL of 0.1% FA (peptide concentration of 0.5 µg/µL). Finally, 17 µL of each sample was mixed with PRTC mixture (12.5 fmoL/µL) in 1:1 v/v ratio.

1.7 Data-dependent LC-MS/MS analysis

The samples were first loaded onto a trap column (75 µm × 2 cm, C18, 3 µm and 100 Å, Thermo Fisher Scientific, San José, California, USA) at 3 µL/min using solvent A (0.1% FA) and then separated using analytical column (75 µm × 50 cm [or 25 cm], C18, 2 µm, 100 Å, Thermo Fisher Scientific) using 300 nL/min flow rate and column temperature of 35˚C. A linear gradient was applied for liquid chromatography separation, using solvent A and solvent B (0.1% FA in ACN). The gradient of non-fractionated samples went from 5 to 40% B in the first 120 minutes, followed by raise to 90% B in the next 5 minutes and maintained for 10 minutes. The fractionated samples were separated in a 90 minutes gradient (5 to 40% B). Samples injections were randomized for each analyzed data set (1D50, 1D25 and 2D25) separately and 45 minutes blank injections were applied after each sample analysis.

Brandsma C-A, *et al. Thorax* 2020; 75:180–183. doi: 10.1136/thoraxjnl-2019-213200

Two and 4 µL of each sample equivalent to 1 µg of protein content was injected onto the column (for unfractioned and fractioned samples, respectively).

Both unfractionated and fractionated samples were analysed using a Q-Exactive Plus Orbitrap mass spectrometer connected to an Easy-nLC 1000 pump (Thermo Fisher Scientific) in a data-dependent acquisition mode (DDA). Full MS scans were acquired over m/z range of 350–1800 Da with resolution of 70,000 (m/z 200 Da), target automatic gain control (AGC) was 10^6 and maximum ion injection time was 100 ms. The ten most intense peaks with charge state ≥ 2 were fragmented in a HCD collision cell with 30% normalised collision energy. Tandem mass spectra were acquired over m/z range of 200–2000 Da with resolution of 35,000 (at m/z 200 Da), target AGC value of 10^6 and 120 ms maximum injection time. The precursor ion selection threshold for fragmentation was set to $4.2 \cdot 10^4$ and dynamic exclusion of precursor ions was set to 20 seconds.

In total three datasets where acquired: 18 non-fractionated samples without replicate were analysed with 50 cm analytical column (1D50), all (19) unfractionated samples were analysed with 25 cm analytical column (1D25) in triplicate, and all (19) fractionated samples were analysed without replicate (except sample COPD5, which was analysed in triplicate) using 25 cm analytical column (2D25).

The synthetic peptides were analysed with the same type, but different instrument than the original analysis. We used an Q-Exactive Plus Orbitrap mass spectrometer connected to an Easy-nLC 1000 pump. Full MS scans were acquired with mass range of 300-1650 Da. The 23 synthetic peptides (non-reference peptides exclusively detected in control or COPD with length shorter than 30 amino acids) and the reference synthetic peptides of HWYITTGPVREK were pooled in three samples and analysed using 45 minutes of gradient instead of 90 minutes used to analyse the human lung tissue samples. All other settings were the same used to analyse human lung tissue samples.

1.8 Data pre-processing, peptide and protein identification

PEAKS Studio v8.0[9] (Bioinformatics solution Inc, Waterloo, Canada) was used for peptide and protein identification with false discovery rate (FDR) <1% at peptide-spectrum-match (PSM), peptide and protein level and using decoy approach for FDR calculation. PSM is a general term used in bottom-up proteomics and expresses the match between a fragment ion spectrum (MS/MS) and the (most probable) peptide sequence derived from the protein sequence database used in database search (PEAKS). FDR calculation was performed for each analysed sample for all three datasets (i.e. replicates and fractions were combined in one identification list). For each sample, a fasta file obtained from the RNAseq data of the same sample was used. The following settings were used for PSM search: precursor error tolerance 10 ppm; precursor mass search type: monoisotopic; fragment mass error tolerance: 0.02 Da; cleavage enzyme: trypsin with 2 missed cleavages and usage of only tryptic peptides. Carbamidomethylation was used for fixed modification, and methionine oxidation was used as variable modification, with 6 maximal variable post-translational modifications per peptide. Only high-quality MS/MS spectra were used for PSM (>0.65). PEAKS label-free analysis was performed with 10 ppm mass error tolerance and 5.0 minutes of retention time shift tolerance. PEAKS label-free ion count analysis has been performed individually only for 1D25 and 2D25 data sets, because in 1D50 dataset the retention time shift was larger than 5 minutes due to 2 times blockage and exchange of the 50 cm LC column, which prevent accurate analysis.

In house developed Perl scripts were used to: (1) calculate raw spectral counts (SPC, i.e. number of PSM) from peptides mapping to unique Ensembl genes (ENSG); (2) to identify peptides with a sequence that did not map to canonical sequences in Ensembl and Uniprot. The latter is called "non-

reference" in the manuscript. To compare protein and gene expression levels, quantitative data was aggregated (summed) to the Ensembl gene level using Ensembl identifiers.

1.9 Statistical analysis and proteogenomics data integration

The data pre-processing workflow used for raw RNAseq and LC-MS/MS pre-processing and proteogenomics data integration is shown in **Figure S1**.

In house developed R scripts were used for (1) differential protein and transcript analysis, (2) identification of non-reference peptides that are different between COPD patients and controls and (3) mapping differentially expressed genes at proteome and/or transcript level on a STRING protein-protein interaction network (https://string-db.org), (4) visualise annotated MS/MS and perform analysis with SpectrumAI R script.

(1) Differential protein and transcript analysis. Only genes having three PSM for proteomics occurring at least half of the samples in one of the sample groups (control or COPD) and at least one fragment per million (FPM) occurring at least half of the samples in one of the sample groups for transcriptomics was considered for the differential expression analysis. The data was normalized by upper quartile normalization and the RUVseq (k=1) package was used to remove unwanted (technical) variation according to the first principal component using residuals (RUVr approach) [10]. Next, the edgeR package [11,12] was used for linear regression of the data following a negative binomial distribution and correction for age, gender and age×gender interaction was included. The three proteomics datasets were analysed combined with two dummy variables using 1D25 dataset as reference using the following linear regression model:

$$Y_{protein} = a + b_{D\_1D50} \cdot D_{1D50} + b_{D\_2D25} \cdot D_{2D25} + b_{age} \cdot Age + b_{gender} \cdot Gender + b_{age\times gender} \cdot (Age \times Gender) + b_{disease} \cdot Disease + residuals.$$

The independent variable D_{1D50} is corresponding with the dummy variable with value of 1 if the sample is from the 1D50 dataset and 0 otherwise, the independent variable D_{2D25} is corresponding with the dummy variable with value of 1 if the sample is from 2D25 dataset and 0 otherwise, *Age* reflects the age of the patient in year, *Gender* is the dummy for gender with 0 for male and 1 for female, the *Age×Gender* interaction and the *Disease* corresponding to dummy with 0 for control and 1 for COPD patients. The $Y_{protein}$ is the dependent variable having the transcriptomics or proteomics data in counts and using linear regression model for negative binomial (count) data. P-values were corrected for multiple testing using Benjamini-Hochberg (FDR) method and threshold for adjusted (or nominal) p-value was 0.05.

The principal component analysis (PCA) plots with the raw and post-normalisation data using upper quartile normalization and removal of unwanted variation using residuals (RUVr approach) are shown in **Figure S2**. Plots were created with ggplot2, vennDiagram and heatmap2 R packages. Differential expression of spectral count data of non-reference differential peptides summed in all data sets were confirmed with separate heat maps in 1D25 and 2D25 data sets using label-free quantification module of PEAKS.

(2) Identification of non-reference peptides differing between COPD and control. Non-reference peptides that were detected with minimally 5 PSM in at least 4 COPD or control samples were combined. From this list non-reference peptides that were only identified in COPD or control samples were selected and the remaining non-reference peptides was subjected to Mann-Whitney U test to assess which non-reference peptides were different between COPD and controls samples. Differential

Brandsma C-A, *et al. Thorax* 2020; 75:180–183. doi: 10.1136/thoraxjnl-2019-213200

non-reference peptides mapping only to new gene models, were mapped to Swissprot (2017 July) using NCBI pBlast[13], to determine from which gene the peptide sequence was derived. Immunopeptides were further mapped with ANARCI[14] and IgBlast[15] tools to identify their mapping region in immunoglobulins. Visualisation was performed with qplot R package. Highest PEAKS PSM score ($-10\cdot\log_{10}$(p-value of PSM)) for the 61 differential peptides was extracted from the combined 1D50, 1D25 and 2D25 datasets and is summarized in section 6 “MS/MS spectra and identification parameters of 61 non-reference peptides” in this supporting information document. The MS/MS spectra are annotated with fragment ions and the fragment ion table of the best scoring PSM are reported. Gene ontology analysis was performed with GProfiler R package[16].

(3) STRING protein-protein interaction network visualisation. String analysis was performed for differentially expressed proteins and genes at an FDR<0.01 for either (1) proteomics only or (2) combined set of differential expressed genes at either proteomics or transcript level. The Igraph R library[17] was used for network visualisation and STRINGdb package[18] to interact with the STRING protein-protein interaction database. In the STRING network analysis we used only “experiments” and “database” links with a score larger than 399. The STRING database has in total 4 274 001 connections for 19 247 proteins, from which 16 837 have these two types of connections with scores larger than 399 from 4 685 proteins. Our STRING analysis was performed on 94 differentially expressed proteins at FDR 0.01 to avoid a highly dense network. These 94 proteins mapped to the STRING database with a median of 0 connections and an average of 1.75 connections per node, while the STRING network of our proteomics dataset showed a median connection of 2 and an average connection of 2.77. Connection degree distribution is, however, not normal and the density plot (**Figure S8**) of the connection distribution shows the overall larger connection degree distribution in our proteomics dataset compared to the distribution of the STRING database using the connections described above, i.e. what would be expected from STRING by selecting genes randomly.

(4) Visualisation of endogenous and synthetic peptide, and SpectrumAI validation of single amino acid variant non-referenced peptides was performed with R packages mzR and RforProteomics, while SpectrumAI was downloaded from https://github.com/yafeng/SpectrumAI. SpectrumAI is a MS/MS spectra analysis tool that was used to identify fragment ions evidence in MS/MS spectra for the presence of the altered amino acids among newly identified, non-reference, single amino acid variant (SAAV) peptides. mzR and RforProteomics R packages was used to visualise annotated MS/MS spectra. The excel sheet containing the SpectrumAI analysis results is available as supplementary file 2.

1.10 Data files.

mRNA and protein data is available for future collaboration on request.

2 Supporting information figures

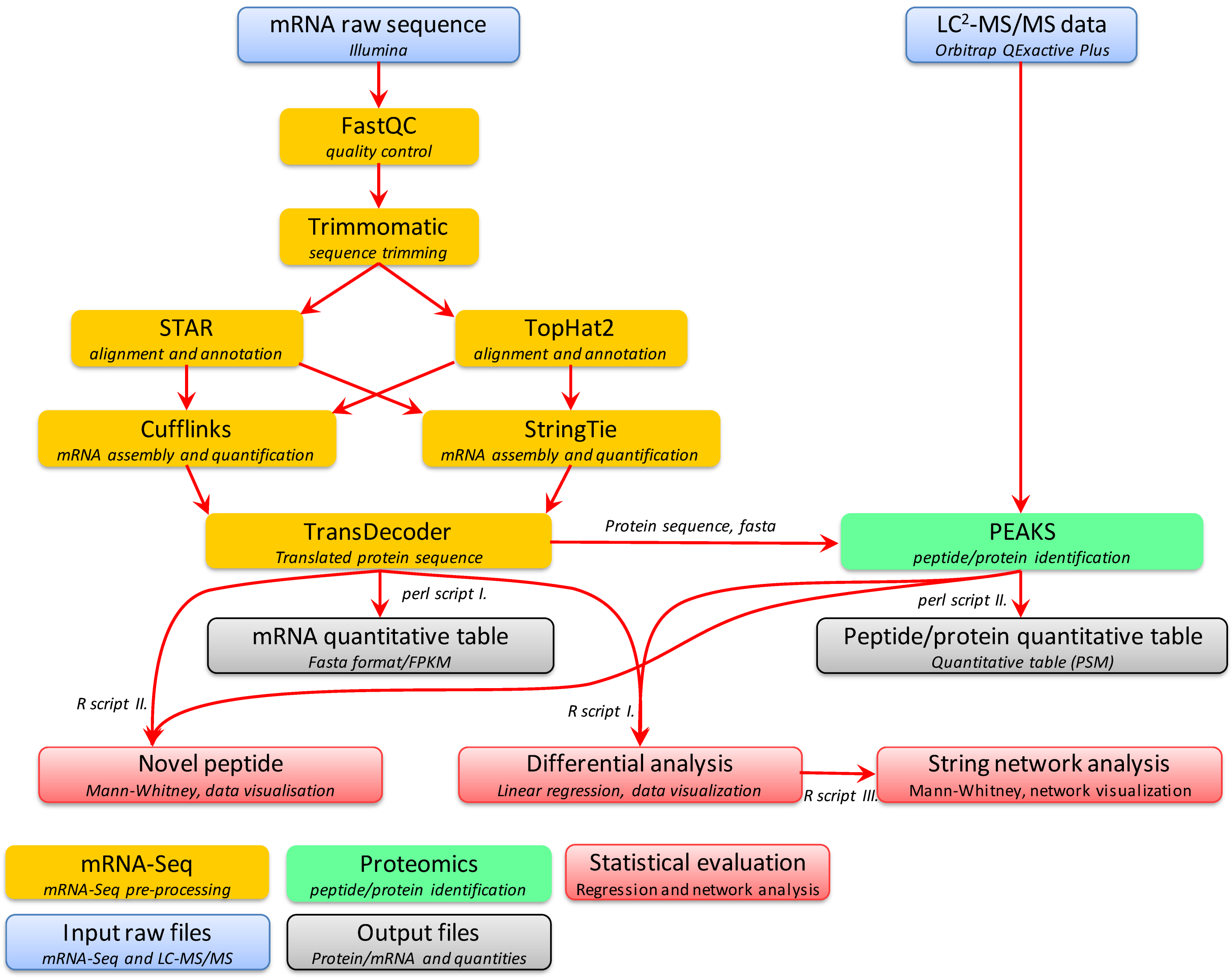

Figure S1. Scheme of the proteogenomics data integration workflow that was used for the joint pre-processing of the LC-MS/MS proteomics and RNAseq data obtained from the same human lung tissue samples.

Brandsma C-A, *et al. Thorax* 2020; 75:180–183. doi: 10.1136/thoraxjnl-2019-213200

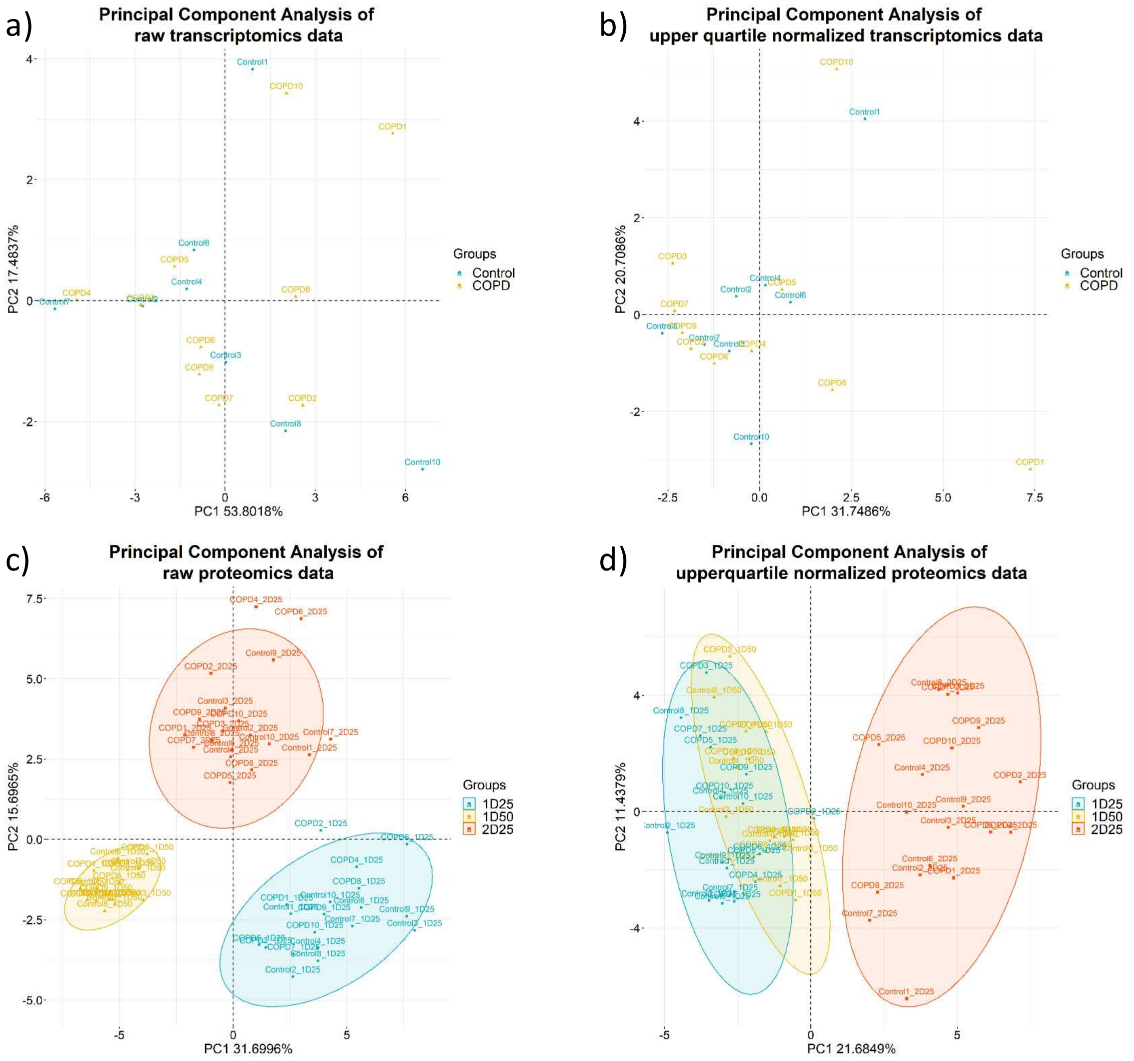

Figure S2. **Principal component score plots** showing the first two principal components of raw transcriptomics (a), raw proteomics data (c) and the same datasets (b, d) after upper quartile normalization and removal of unwanted variation using residuals (RUVr).

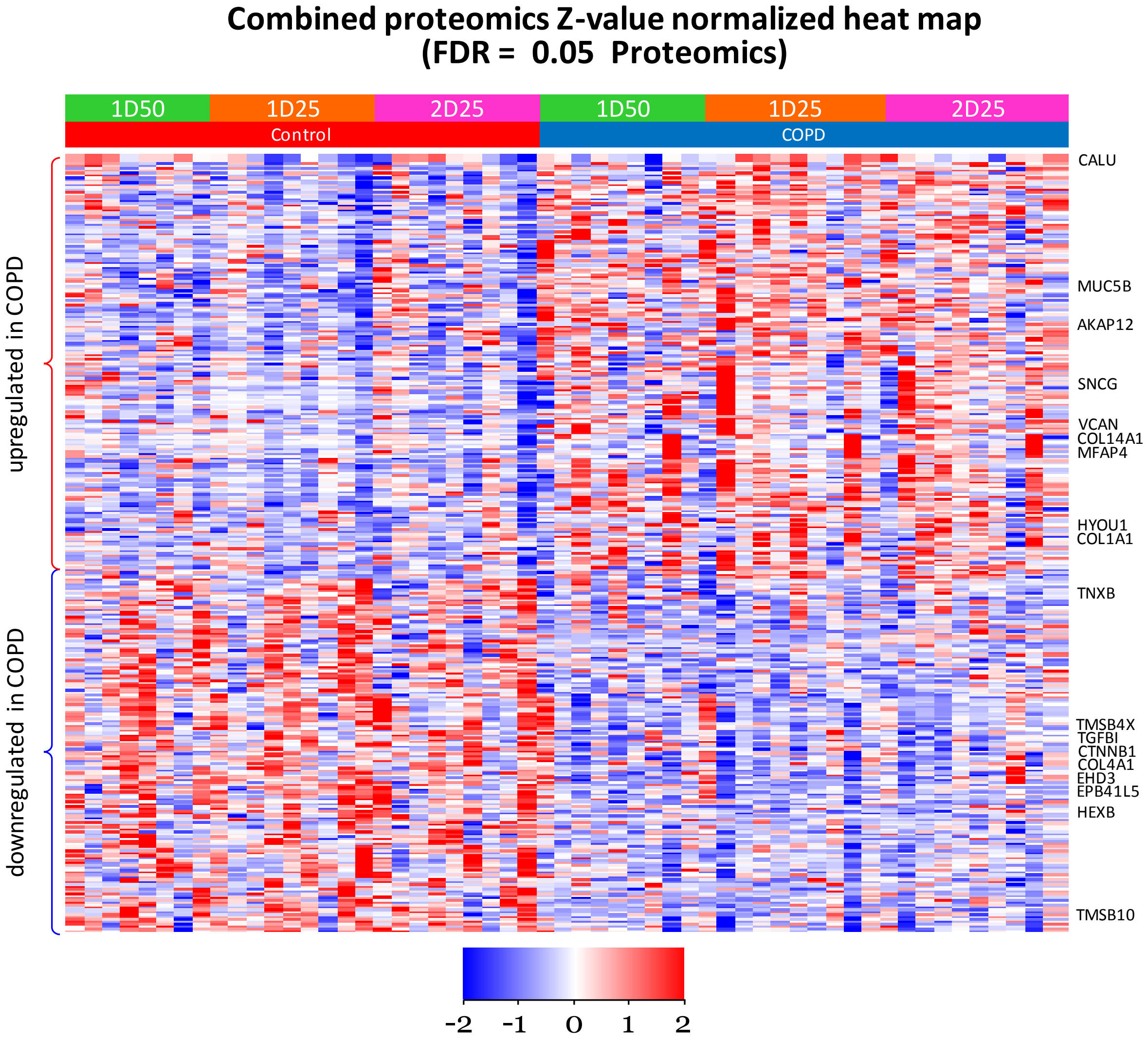

Figure S3. **Heat map of differential protein expression in severe COPD lung tissue**, showing upregulated proteins (top) and downregulated proteins (bottom). The heat map shows the Z-scores of the residuals of the linear regression with age, gender and age×gender interaction. Data generated from the 1D50, 1D25 and 2D25 data sets is given in the column headers and shows consistency across the data sets. The named genes are discussed in the text.

Brandsma C-A, *et al. Thorax* 2020; 75:180–183. doi: 10.1136/thoraxjnl-2019-213200

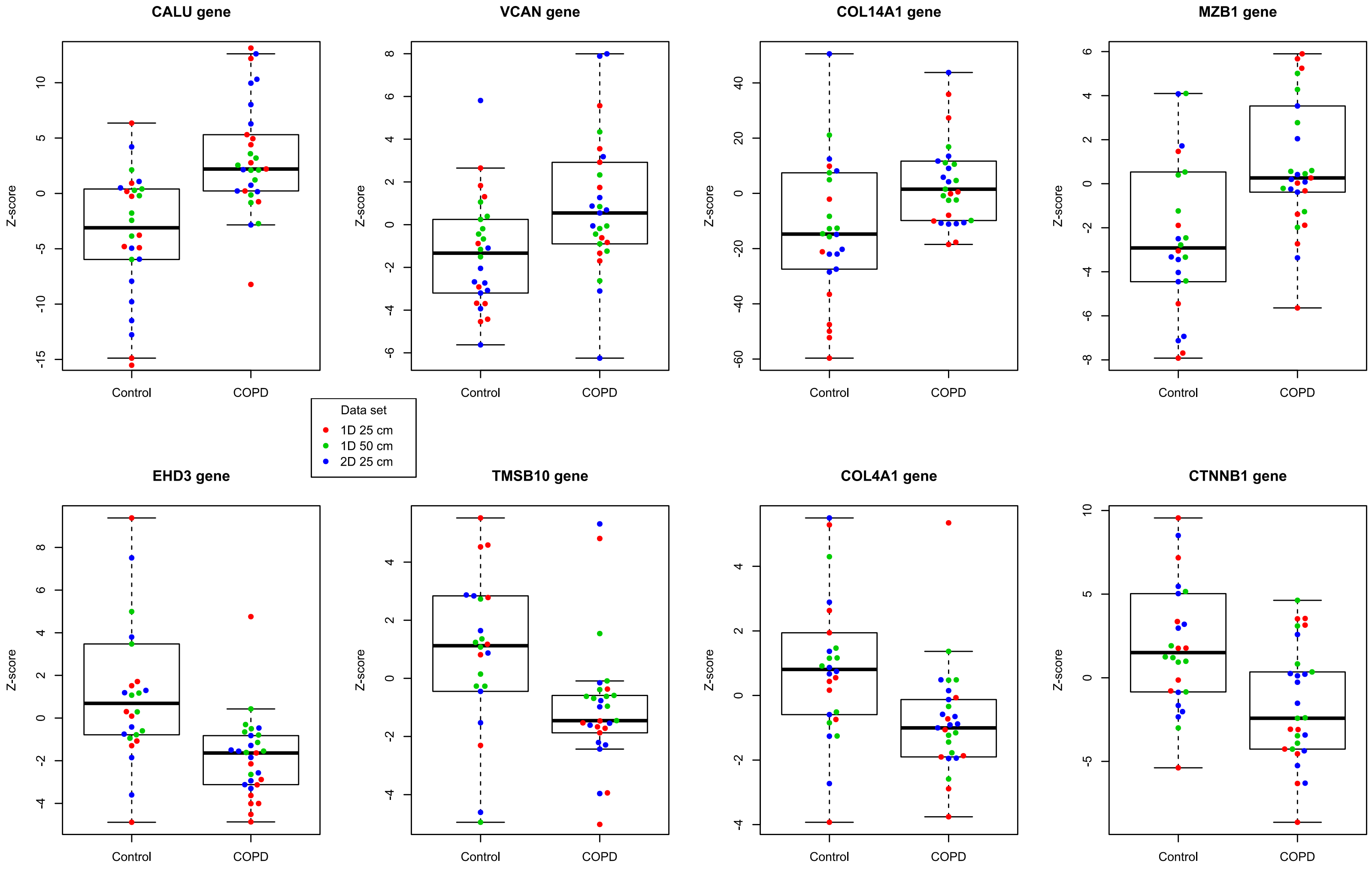

Figure S4. Bee swarm plots are shown for 4 proteins with higher protein levels in COPD (i.e. CALU, VCAN, COL14A1, MZB1) and 4 proteins with lower protein levels in COPD (i.e. EHD3, TMSB10, COL4A1, CTNNB1). Z-scores (residuals) are depicted for data generated in each proteomics dataset (1D 25cm in red, 1D 50 cm in green, 2D 25 cm in blue) after correction for confounders.

Brandsma C-A, *et al. Thorax* 2020; 75:180–183. doi: 10.1136/thoraxjnl-2019-213200

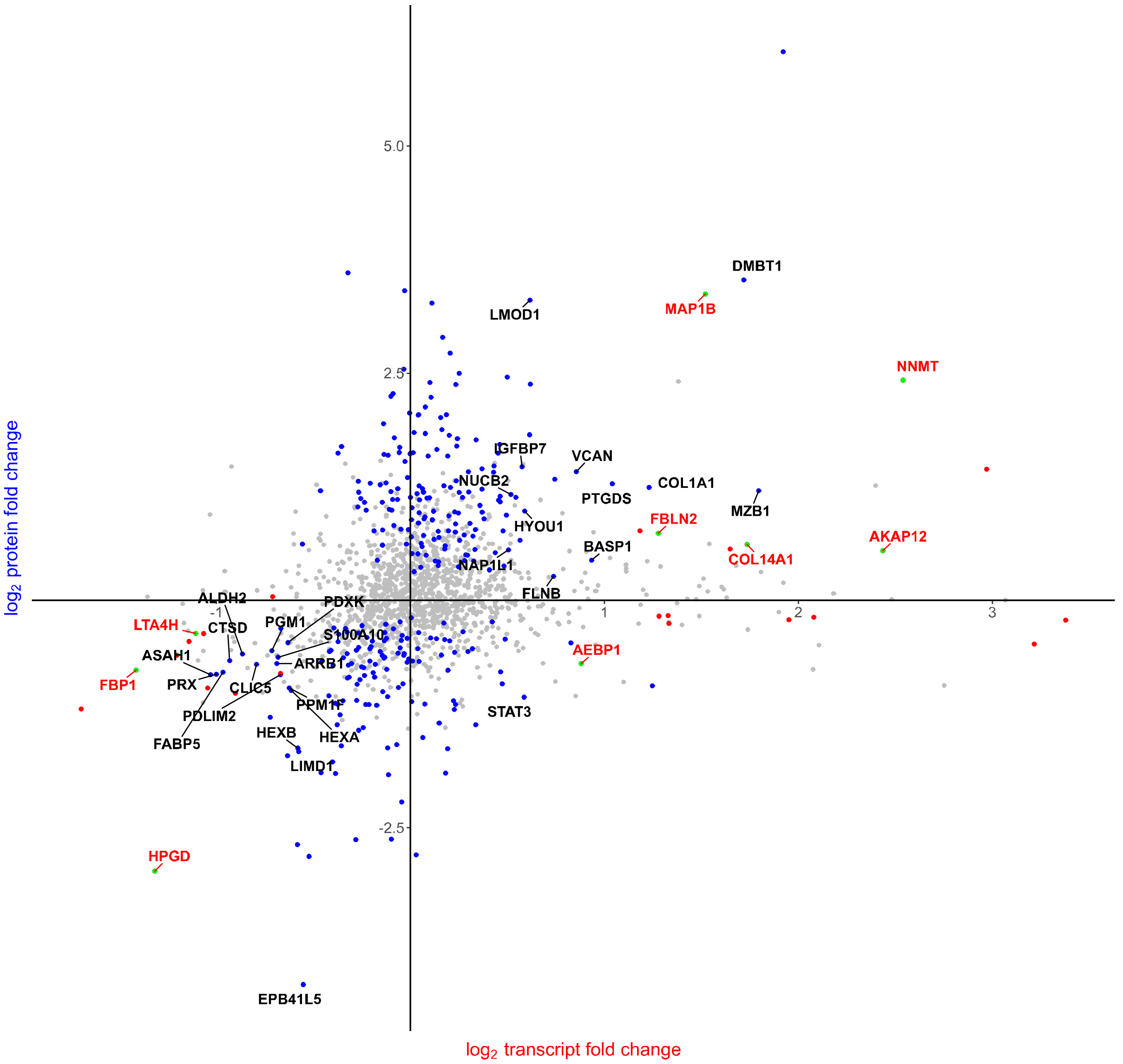

Figure S5. **Fold change scatter plot of transcript and protein expression**. Fold change scatter plot of transcript (horizontal axis) and protein expression (vertical axis) of protein-coding human genes identified in control and COPD human lung tissue. Differentially expressed proteins at an FDR <0.05 (blue), differentially expressed transcripts at an FDR <0.05 (red), differential-expression on both the protein and transcript levels at an FDR <0.05 (green) with gene names (red text). All proteins that are differentially-expressed at the transcript level with a nominal p<0.05 are named (black text).

Brandsma C-A, *et al. Thorax* 2020; 75:180–183. doi: 10.1136/thoraxjnl-2019-213200

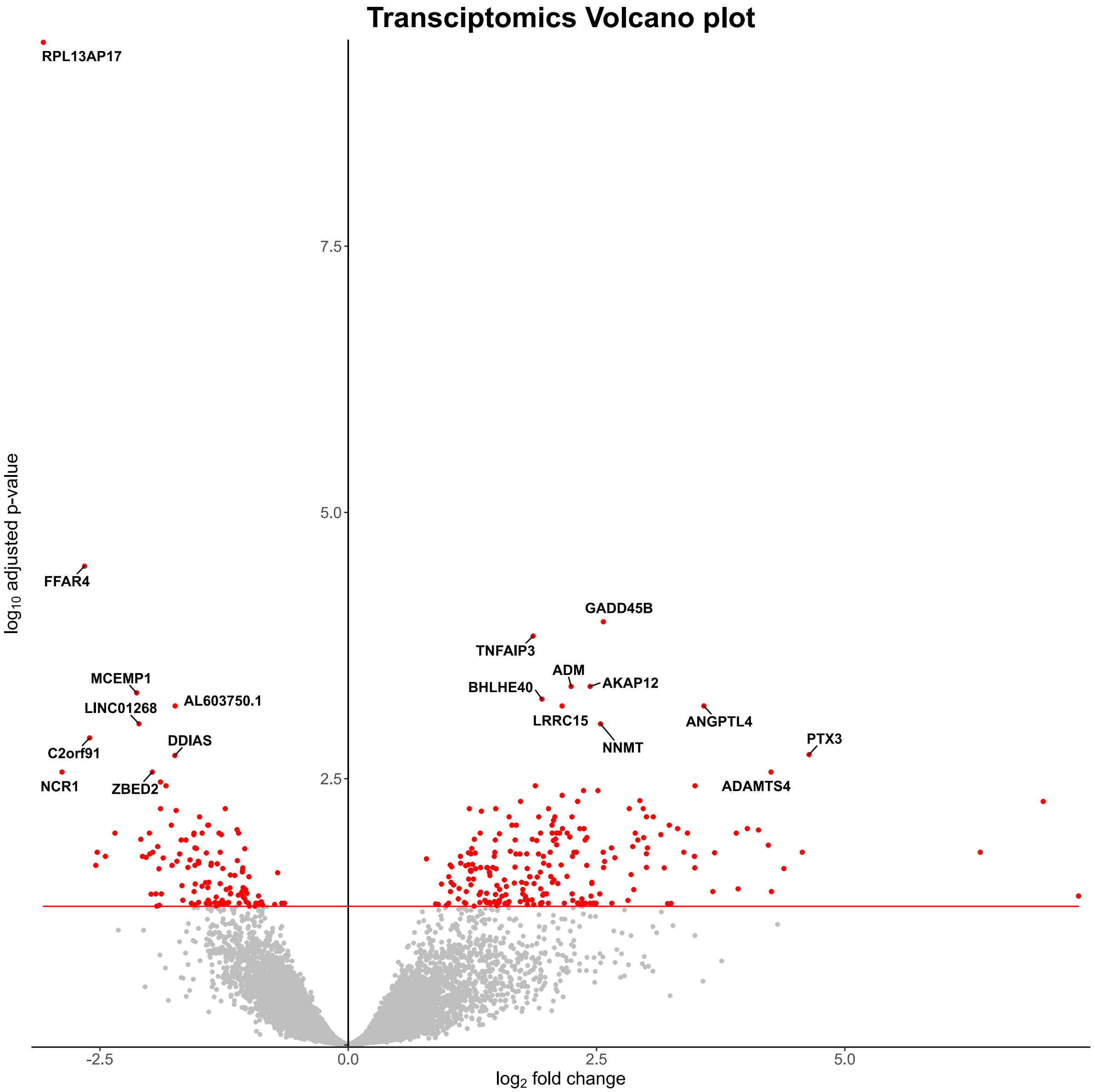

Figure S6. **Differential gene expression in severe COPD lung tissue.** Volcano plot of all genes consistently expressed in COPD and control lung tissue. Differentially expressed genes (FDR < 0.05) are shown in red. The most significantly differentially expressed genes are indicated with gene symbol.

Brandsma C-A, *et al. Thorax* 2020; 75:180–183. doi: 10.1136/thoraxjnl-2019-213200

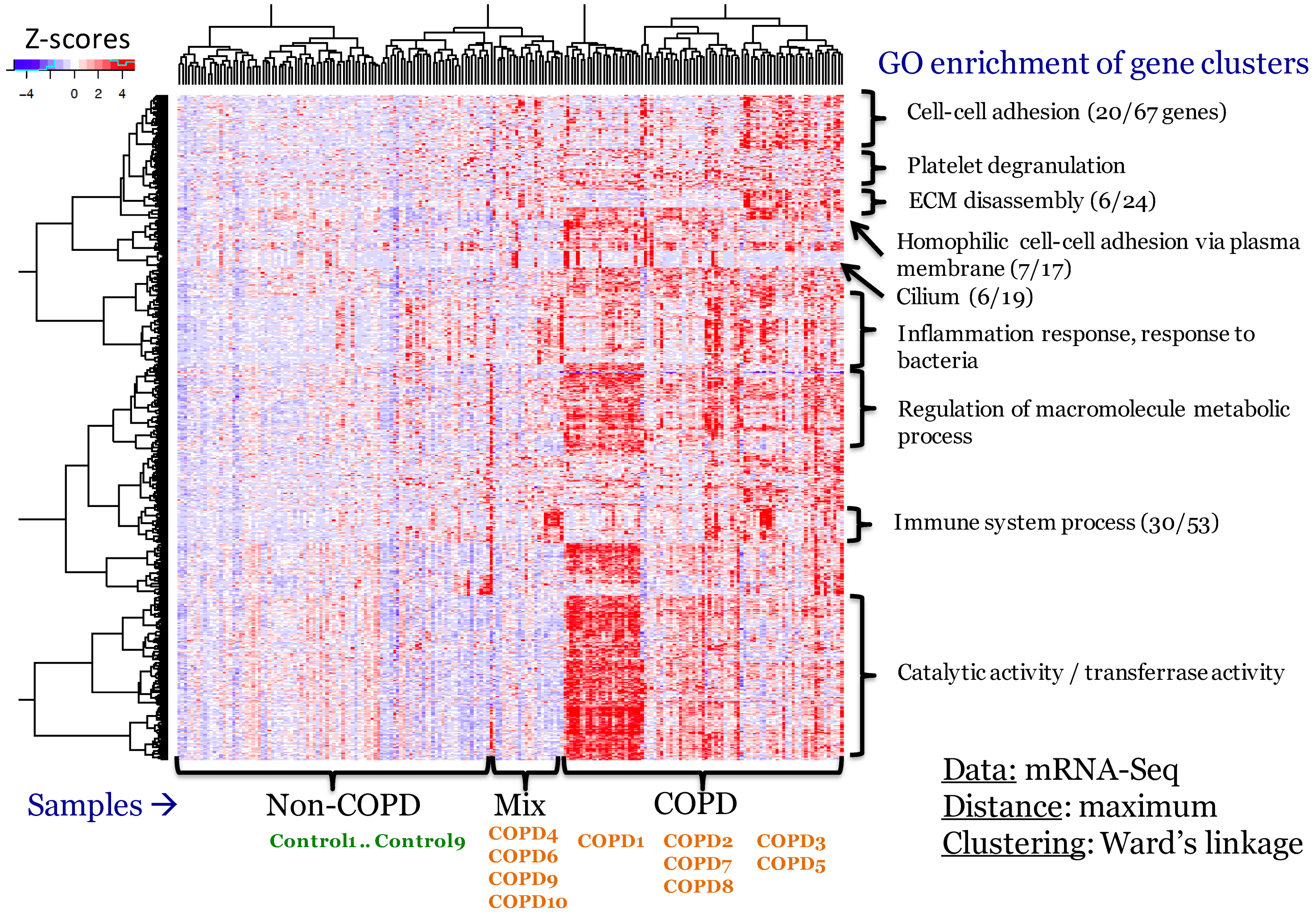

Figure S7. **Heat map of 350 differential transcripts** (FDR<0.05) in human lung tissue measured in this study (10 COPD and 8 controls) and in 189 samples from a South Korean study[19]. The heat map shows the Z-scores of the transcripts counts after upper-quartile normalization. Both samples and transcripts expression are submitted to hierarchical clustering using maximum Ward's linkage. The most significant gene ontology enrichment of clustered transcript expression is shown right. Samples are clustered in there groups as control, mixed control and COPD and COPD, and our samples are highlighted in the bottom.

Brandsma C-A, *et al. Thorax* 2020; 75:180–183. doi: 10.1136/thoraxjnl-2019-213200

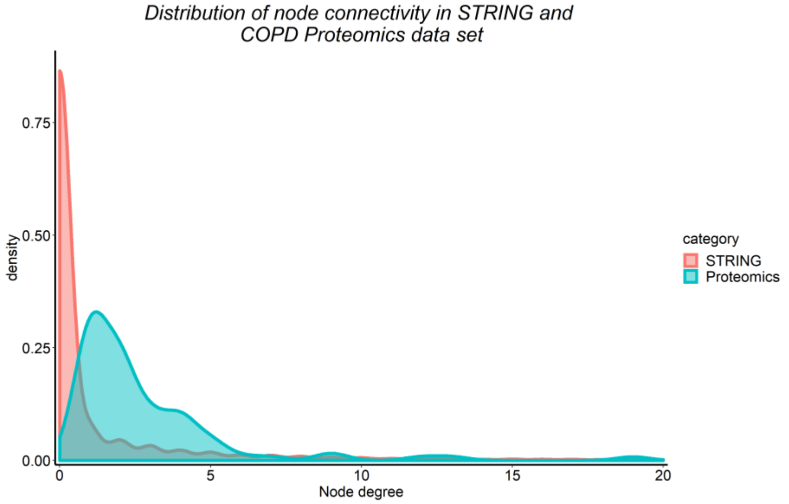

Figure S8. **Density plot** showing the connectivity (number of connections, degree) distribution in STRING and in differentially expressed proteins of combined COPD dataset (FDR 0.01) using "experiments" and "database" connections with scores higher than 399. STRING database contained 4 274 001 connections (any types) between 19 247 proteins, from which 16 837 were type defined above and which connections were between 4 685 proteins. The plot reflects the higher connections (degree distribution) of nodes between the differential proteins of COPD proteomics data set.

Brandsma C-A, *et al. Thorax* 2020; 75:180–183. doi: 10.1136/thoraxjnl-2019-213200

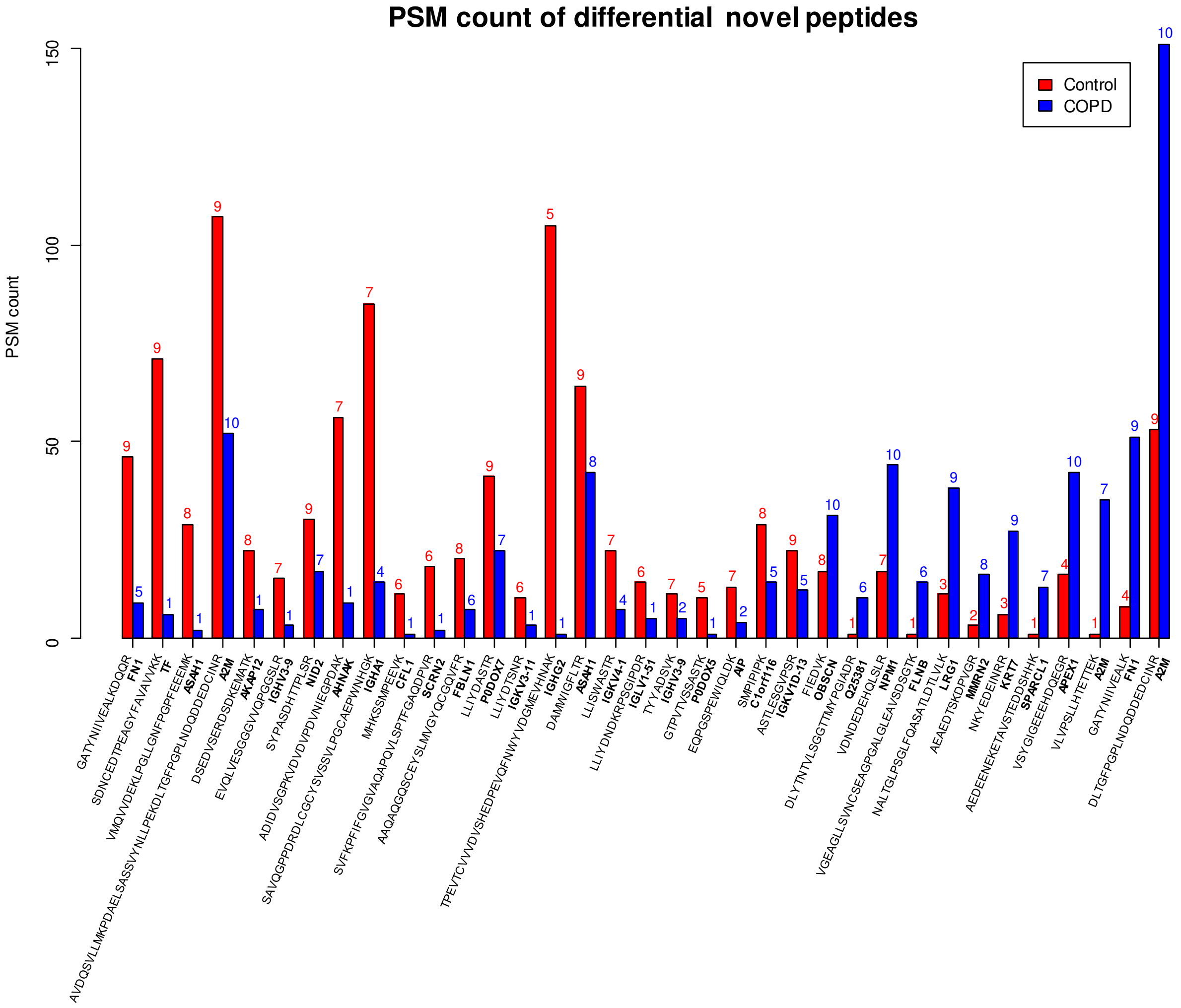

Figure S9. **Differential non-reference sample specific peptides present in severe COPD and control lung tissue.** Bar plot of the number of MS/MS spectra (PSMs) for non-reference sample specific peptides that were differentially-expressed based on Mann-Whitney U test (Figure 1D) but not exclusively present in one of the sample group. Only peptides with at least 5 PSMs and present in at least 4 COPD patients or controls were considered. The number of samples where the non-reference peptide was identified is indicated at the top of each bar.

Brandsma C-A, *et al. Thorax* 2020; 75:180–183. doi: 10.1136/thoraxjnl-2019-213200

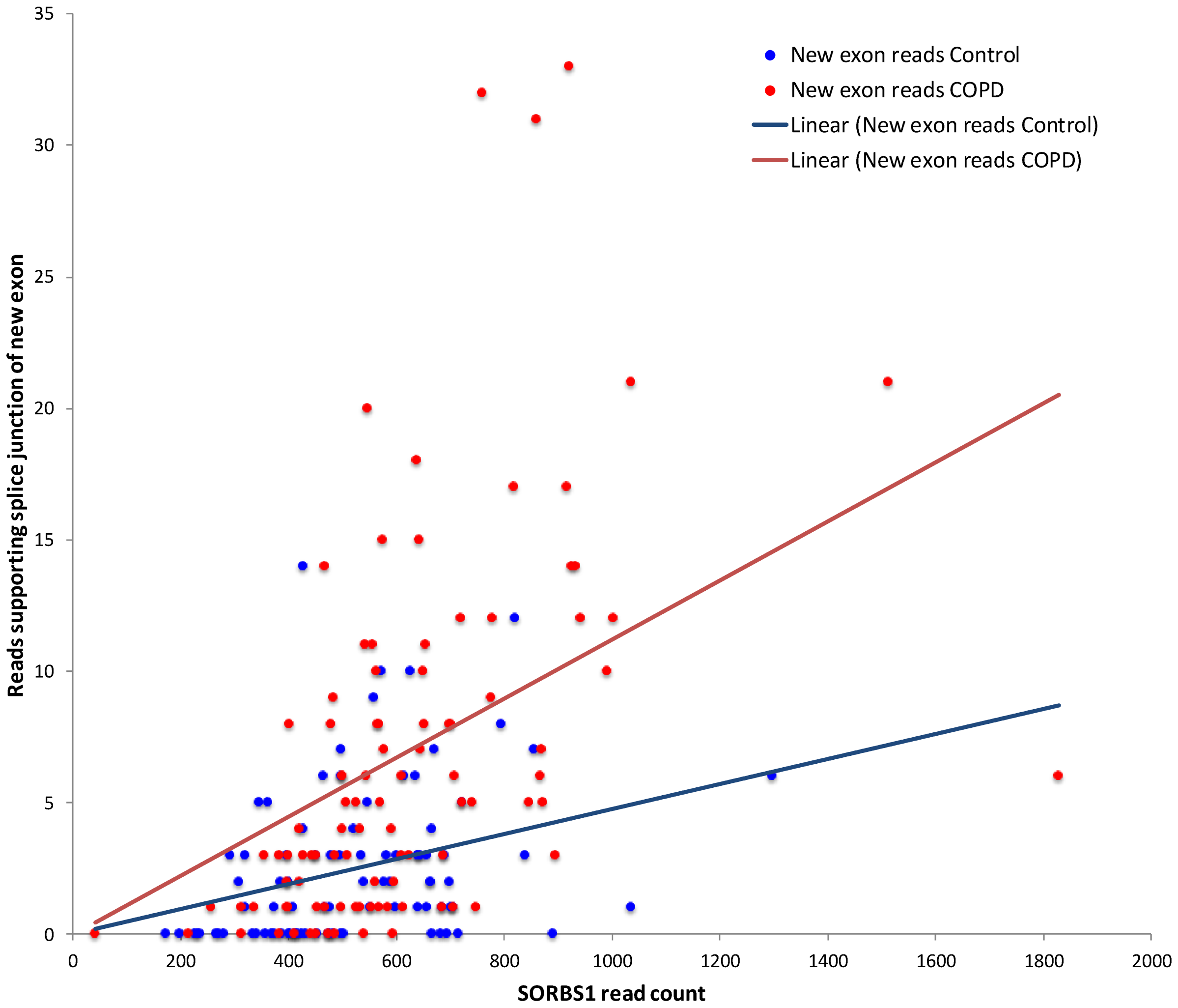

Figure S10. **Novel SORBS1 exon usage in independent lung tissue dataset.** The differential usage of the novel exon in SORBS1 in RNA sequencing data from an independent Korean dataset of COPD and control lung tissues is shown. The number of reads supporting the splice junction of the novel exon is plotted against the total read count for SORBS 1. The reds dots and line representing COPD samples and the blue dots and line representing control samples. A higher slope of the line indicates a higher proportion of new exon usage.

Brandsma C-A, *et al. Thorax* 2020; 75:180–183. doi: 10.1136/thoraxjnl-2019-213200

Peptide ion counts in dataset COPD 1D 25cm

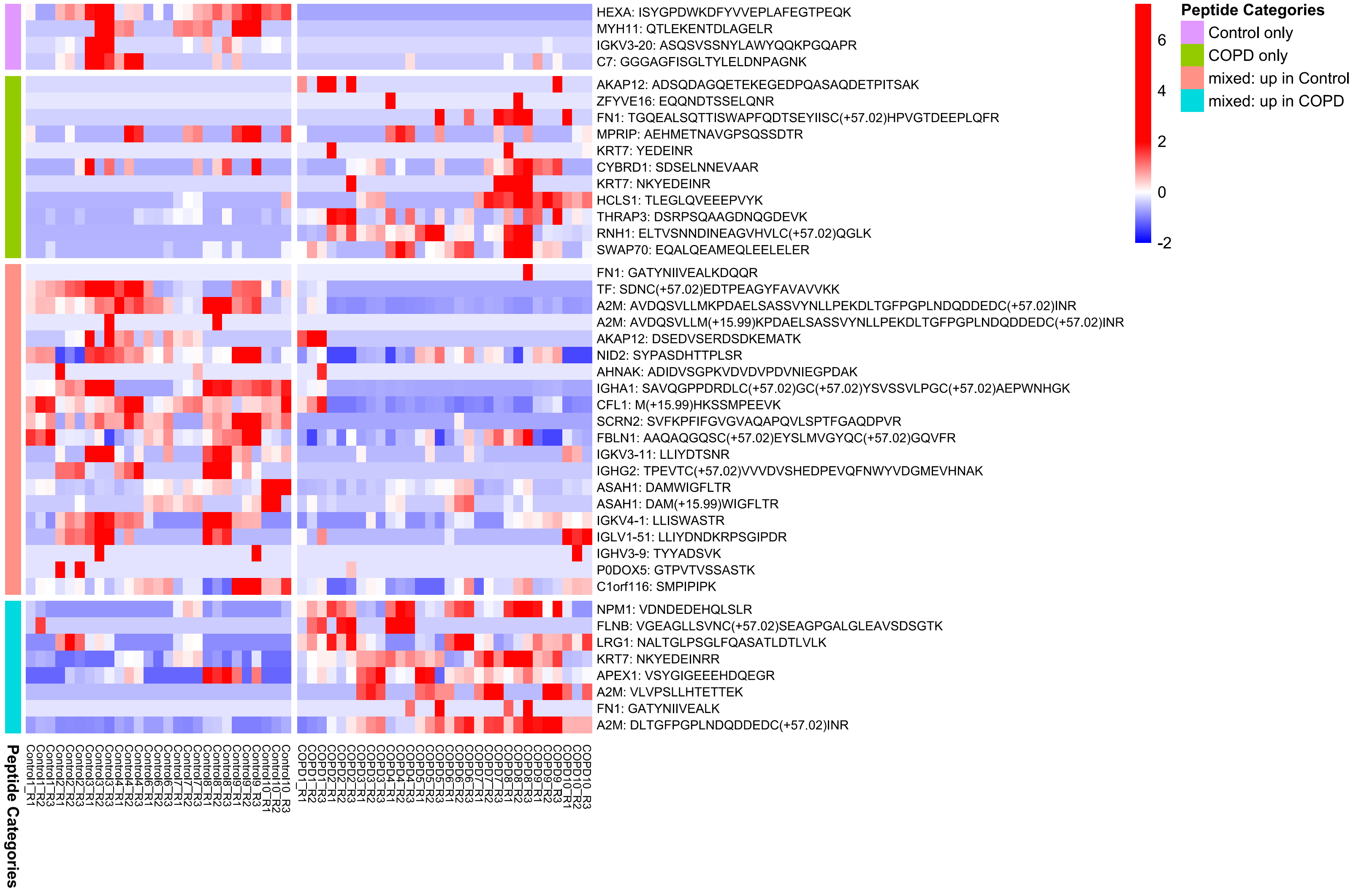

Figure S11. Ion count levels of the 44 non-reference peptides out of 61 shown differential with spectral count differential analysis in 1D25 dataset using PEAKS ion count quantification module. The heat map shows the Z-scores of the peptide MS1 intensity. The peptides are classified according PSM differential analysis as up or down identified in control or COPD lung tissue exclusively or partially.

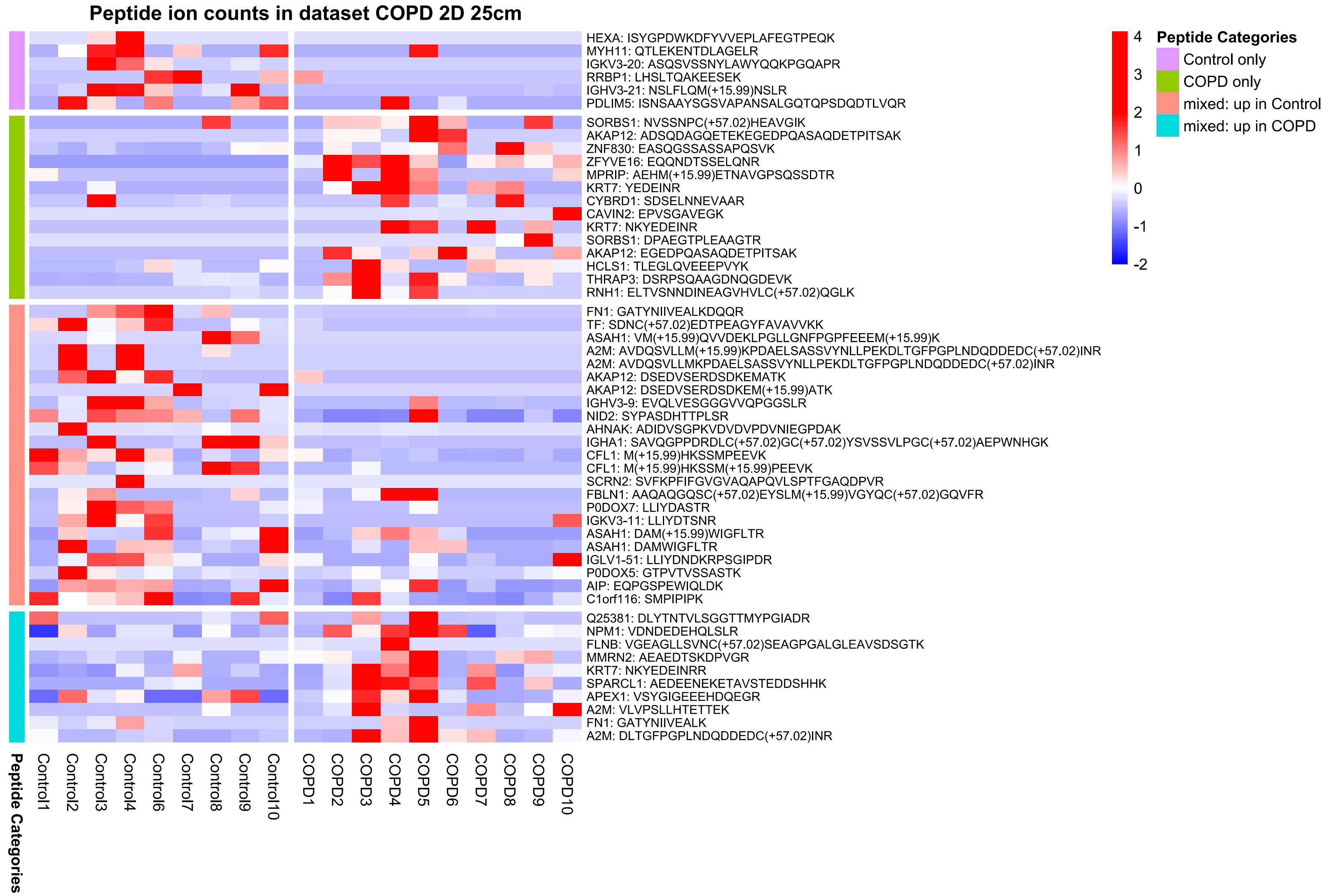

Figure S12. Ion count levels of the 49 non-reference peptides out of 61 shown differential with spectral count differential analysis in 2D25 dataset using PEAKS ion count quantification module. The heat map shows the Z-scores of the peptide MS1 intensity. The peptides are classified according PSM differential analysis as up or down identified in control or COPD lung tissue exclusively or partially.

3 Supporting information tables

Table S1. Complete list of differentially expressed transcripts and proteins with FDR < 0.05.
Separate Excel sheet (file name Protein_transcipt_differential_expression.xlsx).

Table S2. Top 15 enriched Gene Ontology terms based on differential protein expression with FDR p<0.01 in severe COPD.

	GO term id	GO term description	p-value	FDR p-value
1	GO:0030198	extracellular matrix organization	$8.61{\cdot}10^{-8}$	$1.05{\cdot}10^{-4}$
2	GO:0043062	extracellular structure organization	$8.95{\cdot}10^{-8}$	$1.05{\cdot}10^{-4}$
3	GO:1901575	organic substance catabolic process	$1.07{\cdot}10^{-7}$	$1.05{\cdot}10^{-4}$
4	GO:0016192	vesicle-mediated transport	$1.96{\cdot}10^{-7}$	$1.44{\cdot}10^{-4}$
5	GO:0007010	cytoskeleton organization	$2.16{\cdot}10^{-6}$	$1.27{\cdot}10^{-3}$
6	GO:0044248	cellular catabolic process	$4.26{\cdot}10^{-6}$	$1.98{\cdot}10^{-3}$
7	GO:0006897	endocytosis	$4.71{\cdot}10^{-6}$	$1.98{\cdot}10^{-3}$
8	GO:0010941	regulation of cell death	$6.44{\cdot}10^{-6}$	$2.13{\cdot}10^{-3}$
9	GO:0044712	single-organism catabolic process	$6.52{\cdot}10^{-6}$	$2.13{\cdot}10^{-3}$
10	GO:0060548	negative regulation of cell death	$1.02{\cdot}10^{-5}$	$2.58{\cdot}10^{-3}$
11	GO:1901564	organonitrogen compound metabolic process	$1.03{\cdot}10^{-5}$	$2.58{\cdot}10^{-3}$
12	GO:0006915	apoptotic process	$1.05{\cdot}10^{-5}$	$2.58{\cdot}10^{-3}$
13	GO:0012501	programmed cell death	$1.21{\cdot}10^{-5}$	$2.68{\cdot}10^{-3}$
14	GO:0043066	negative regulation of apoptotic process	$1.27{\cdot}10^{-5}$	$2.68{\cdot}10^{-3}$
15	GO:0043069	negative regulation of programmed cell death	$1.39{\cdot}10^{-5}$	$2.74{\cdot}10^{-3}$

GO: Gene Ontology, p-value represent the nominal p-value, FDR p-value was obtained after applying Benjamini-Hochberg procedure for multiple testing correction.

Table S3. **Properties of non-reference peptide sequences that were differentially expressed between COPD and Control.**

Peptides only present in COPD

Peptide sequence	Quality score	Gene	Effect	Ion support	Flanking ion support
ADSQDAGQETEKEGEDPQASAQDETPITSAK	134.83	AKAP12	E1600D amino acid substitution	✗	✗
DSRPSQAAGDNQGDEVK	132.86	THRAP3	A201V amino acid substitution	✗	✗
TGQEALSQTTISWAPFQDTSEYIISCHPVGTDEEPLQFR	131.44	FN1	Native	NA	NA
AEHMETNAVGPSQSSDTR	129.43	MPRIP	P327Q amino acid substitution	✗	✗
EGEDPQASAQDETPITSAK	129.34	AKAP12	E1600D amino acid substitution	✗	✗
DPAEGTPLEAAGTR	118.25	SORBS1	Splice variant, exon extension	NA	NA
ELTVSNNDINEAGVHVLCQGLK	115.80	RNH1	R188H amino acid substitution	✗	✗
TLEGLQVEEEPVYK	106.37	HCLS1	E361K amino acid substitution	✗	✗
EASQGSSASSAPQSVK	105.60	ZNF830	H99Q amino acid substitution	✗	✗
SDSELNNEVAAR	103.81	CYBRD1	S266N amino acid substitution	✗	✗
EQALQEAMEQLEELELER	100.83	SWAP70	Q505E amino acid substitution	✗	✗
EQQNDTSSELQNR	91.55	ZFYVE16	I192T amino acid substitution	✗	✗
YGFQFLR	81.46	PCYOX1	S149F amino acid substitution	✗	✗
NKYEDEINR	70.58	KRT7	H186R amino acid substitution	✗	✗
EPVSGAVEGK	62.17	CAVIN2	Q174E amino acid substitution	✗	✗
NVSSNPCHEAVGIK	56.53	SORBS1	Splice variant, exon extension	NA	NA
YEDEINR	49.80	KRT7	H186R amino acid substitution	✗	✗

Peptides only present in Control

Peptide sequence	Quality score	Gene	Effect	Ion support	Flanking ion support
ISYGPDWKDFYVVEPLAFEGTPEQK	120.51	HEXA	I447V amino acid substitution	✗	✗
ISNSAAYSGSVAPANSALGQTQPSDQDTLVQR	110.84	PDLIM5	T410A amino acid substitution	✗	✗
LHSLTQAKEESEK	110.58	RRBP1	L1043H amino acid substitution	✗	-
GGGAGFISGLTYLELDNPAGNK	108.34	C7	S389T amino acid substitution	✗	✗
ASQSVSSNYLAWYQQKPGQAPR	105.88	IGKV3-20	S52N amino acid substitution	✗	✗
QTLEKENTDLAGELR	77.52	MYH11	A1241T amino acid substitution	✗	✗
NSLFLQMNSLR	76.68	IGHV3-43	Y99F amino acid substitution	✗	✗
LLIYWASAR	68.41	IGKV4-1	T79A amino acid substitution	✗	✗
LLEDLR	33.62	OTOA/PDE4DIP	Native	NA	NA

Peptide sequence, identification score, mapping gene symbol and sequence variant type is shown. The quality scores (-10 log10(p-value of PSM)) reflect the quality of the peptide-to-spectrum-match, i.e., peptide identification by mass spectrometry. The effect 'native' indicates that the peptide sequence is now included in the most recent version of Ensembl, but was absent when proteogenomic data integration was performed. ✗ indicates the presence, while – indicates the absence of Ion support or Flaking ion support provided by SpectrumAI assessment. The presence of Ion support indicates the presence of the altered amino acids, while the presence of Flanking ions providing evidence on the correct location of the altered amino acid in SAAV variants. NA indicates not applicable (variant other than SAAV). The altered amino acids in SAAV variants are highlighted in red.

Table S4. Properties of non-reference peptide sequences that were differentially expressed between COPD and Control.

Peptides different between COPD and Control

Peptide sequence	Quality score	Gene	Effect	Ion support	Flanking ion support
AVDQSVLLMKPDAELSASSVYNLLPEKDLTGFPGPLNDQDDEDCINR	200.00	A2M	N639D amino acid substitution	×	×
SAVQGPPDRDLCGCYSVSSVLPGCAEPWNHGK	200.00	IGHA1	E176D amino acid substitution	×	×
TPEVTCVVVDVSHEDPEVQFNWYVDGMEVHNAK	147.77	IGHG2	V161M amino acid substitution	×	-
SDNCEDTPEAGYFAVAVVKK	145.51	TF	I448V amino acid substitution	×	×
AAQAQGQSCEYSLMVGYQCGQVFR	140.99	FBLN1	Q179R amino acid substitution	×	-
ADIDVSGPKVDVDVPDVNIEGPDAK	138.93	AHNAK	Native	NA	NA
DLTGFPGPLNDQDDEDCINR	138.23	A2M	N639D amino acid substitution	×	×
VMQVVDEKLPGLLGNFPGPFEEEMK	134.27	ASAH1	I109V amino acid substitution	×	-
VGEAGLLSVNCSEAGPGALGLEAVSDSGTK	130.08	FLNB	D1157N amino acid substitution	×	×
SVFKPFIFGVGVAQAPQVLSPTFGAQDPVR	124.78	SCRN2	M331V amino acid substitution	×	×
NALTGLPSGLFQASATLDTLVLK	116.16	LRG1	P133S amino acid substitution	×	×
VSYGIGEEEHDQEGR	115.91	APEX1	D148E amino acid substitution	×	×
EVQLVESGGGVVQPGGSLR	111.36	IGHV3-13	L30V amino acid substitution	×	×
GATYNIIVEALKDQQR	109.33	FN1	V2230I amino acid substitution	×	×
DSEDVSERDSDKEMATK	107.08	AKAP12	K117E amino acid substitution	×	-
LLIYDNDKRPSGIPDR	105.39	IGLV1-51	N72D amino acid substitution	×	×
AEDEENEKETAVSTEDDSHHK	104.77	SPARCL1	A49D amino acid substitution	×	×
SYPASDHTTPLSR	98.27	NID2	G453D amino acid substitution	×	×
DAMWIGFLTR	95.37	ASAH1	V262A amino acid substitution	×	-
GTPVTVSSASTK	94.79	IGHG1	T140P amino acid substitution	×	×
EQPGSPEWIQLDK	93.79	AIP	Q228K amino acid substitution	×	×
VDNDEDEHQLSLR	89.48	NPM1	Paralog	NA	NA
AEAEDTSKDPVGR	86.10	MMRN2	G49S amino acid substitution	×	×
VLVPSLLHTETTEK	84.08	A2M	Native	NA	NA
NKYEDEINRR	82.99	KRT7	H186R amino acid substitution	×	×
DLYTNTVLSGGTTMYPGIADR	78.17	ACTB	Paralog	NA	NA
LLIYDASTR	77.22	IGKV3D-20	S74T amino acid substitution	×	×
ASTLESGVPSR	75.55	IGKV1-5	S75T amino acid substitution	×	×
GATYNIIVEALK	73.04	FN1	V2261I amino acid substitution	×	×
LLIYDTSNR	72.05	IGKV3-11	A71T amino acid substitution	×	×
LLISWASTR	63.08	IGKV4-1	Y75S amino acid substitution	×	×
TYYADSVK	55.49	IGHV3-23	Native	NA	NA
SMPIPIPK	53.15	C1ORF116	S444P amino acid substitution	×	×
FIEDVK	42.76	OBSCN	Native	NA	NA
MHKSSMPEEVK	36.09	CFL1	Paralog	NA	NA

Peptide sequence, identification score, mapping gene symbol and sequence variant type is shown. The quality scores (-10 log10(p-value of PSM)) reflect the quality of the peptide-to-spectrum-match, i.e., peptide identification by mass spectrometry. The effect 'native' indicates that the peptide sequence is now included in the most recent version of Ensembl, but was absent when proteogenomic data integration was performed. ×indicates the presence, while – indicates the absence of Ion support or Flaking ion support provided by SpectrumAI assessment. The presence of Ion support indicates the presence of the altered amino acids, while the presence of Flanking ions providing evidence on the correct location of the altered amino acid in SAAV variants. NA indicates not applicable (variant other than SAAV). The altered amino acids in SAAV variants are highlighted in red.

Brandsma C-A, *et al. Thorax* 2020; 75:180–183. doi: 10.1136/thoraxjnl-2019-213200

Table S5. Non-reference peptide sequences mapping to immunoglobulin proteins

Peptide	Gene	Effect	one representative entry	domain	subdomain	# of mRNA	tool	presence
**NSLFLQMNSLR**	**IGHV3-43**	Y->F	ENST00000434710_Y99F	VH	FR3 position 22	3	ANARCI, IPBlast	4 controls, 5 PSM
**ASQSVSSNYLAWYQQKPGQAPR**	**IGKV3-20**	S->N	ENST00000492167_S52N	VK	CDR1 position 6	1	ANARCI, IPBlast	4 controls, 11 PSM
**LLIYWASAR**	**IGKV4-1**	T->A	ENST00000390243_T79A	VK	FR3 position 1	1	ANARCI, IPBlast	4 controls, 8 PSM
**EVQLVESGGGVVQPGGSLR**	**IGHV3-13**	V->G, L->V	ENST00000390602_L30V	VH	FR1 positions 10, 11	3	ANARCI, IPBlast	7 control, 1 COPD, 15 and 3 PSM
**LLIYDASTR**	**IGKV3D-20**	G->D, N(S)->T	ENST00000390270_S74T	VK	CDR1 position 1, FR3 position 1	5	ANARCI, IPBlast	9 control, 7 COPD, 41 and 22 PSM
**TPEVTCVVVDVSHEDPEVQFNWYVDGMEVHNAK**	**IGHG2**	V->M, new gene models	ENST00000390545_V161M	Ig heavy gamma 2	CH3 domain (constant IgC_CH3 domain)	3	pBlast	5 control, 1 COPD, 105 and 1 PSM
**LLIYDNDKRPSGIPDR**	**IGLV1-51**	N->D	ENST00000390290_N72D	VL	CDR2 position 3	1	ANARCI, IPBlast	6 control, 1 COPD, 14 and 5 PSM
**ASTLESGVPSR**	**IGKV1-5**	S->T	ENST00000496168_S75T	VK	FR3 position 1	2	ANARCI, IPBlast	9 control, 5 COPD, 22 and 12 PSM
**LLIYDTSNR**	**IGKV3-11**	A->T	ENST00000483158_A71T	VK	CDR2 position 2	2	ANARCI, IPBlast	6 control, 1 COPD, 10 and 3 PSM
**LLISWASTR**	**IGKV4-1**	Y->S	ENST00000390243_Y75S	VK	FR2 postion 17	1	ANARCI, IPBlast	7 control, 4 COPD, 22 and 7 PSM
**TYYADSVK**	**IGHV3-23**	Native	ENST00000390609	VH	peptide mapping last position of CDR2 and beginning of FR3	138	ANARCI, IPBlast	7 control, 2 COPD, 11 and 5 PSM
**SAVQGPPDRDLCGCYSVSSVLPGCAEPWNHGK**	**IGHA1**	E->D	ENST00000390547_E176D	Ig heavy alpha 1	peptide mapping to CH2 domain (second constant Ig domain of the heavy chain) in immunoglobulin; IgC_CH2	1	pBlast	7 control, 4 COPD, 85 and 14 PSM
**GTPVTVSSASTK**	**IGHG1**	T->P	ENST00000612473_T140P	Ig heavy gamma 1	peptide mapping the end of IgV-H domain (heterodimer interface)	2	pBlast	5 control, 1 COPD, 10 and 1 PSM

Peptide sequence indicating single amino acid variants in red, immune gene symbol, the genetic variant effect, one representative entry of Ensembl transcript, human immunoglobulins structural domain and subdomains, number of matching mRNA entries, used mapping tools (ANARCI, IgBlast) and presence of the peptide in controls and COPD samples with corresponding PSMs.

4 References

1 UMCG Researchcode. 2018; Available at: https://www.umcg.nl/SiteCollectionDocuments/English/Researchcode/umcg-research-code-2018-en.pdf.

2 Bolger AM, Lohse M, Usadel B. Trimmomatic: a flexible trimmer for Illumina sequence data. *Bioinformatics.* 2014;**30** (suppl 15):2114-2120.

3 Dobin A, Davis CA, Schlesinger F*, et al*. STAR: ultrafast universal RNA-seq aligner. *Bioinformatics.* 2013;**29** (suppl 1):15-21.

4 Kim D, Pertea G, Trapnell C*, et al*. TopHat2: accurate alignment of transcriptomes in the presence of insertions, deletions and gene fusions. *Genome Biol.* 2013;**14** (suppl 4):R36-2013-14-4-r36.

5 Anders S, Pyl PT, Huber W. HTSeq--a Python framework to work with high-throughput sequencing data. *Bioinformatics.* 2015;**31** (suppl 2):166-169.

6 Pertea M, Pertea GM, Antonescu CM*, et al*. StringTie enables improved reconstruction of a transcriptome from RNA-seq reads. *Nat Biotechnol.* 2015;**33** (suppl 3):290-295.

7 Grabherr MG, Haas BJ, Yassour M*, et al*. Full-length transcriptome assembly from RNA-Seq data without a reference genome. *Nat Biotechnol.* 2011;**29** (suppl 7):644-652.

8 Riter LS, Hodge BD, Gooding KM*, et al*. Use of lysozyme as a standard for evaluating the effectiveness of a proteomics process. *J Proteome Res.* 2005;**4** (suppl 1):153-160.

9 Zhang J, Xin L, Shan B*, et al*. PEAKS DB: de novo sequencing assisted database search for sensitive and accurate peptide identification. *Mol Cell Proteomics.* 2012;**11** (suppl 4):M111.010587.

10 Risso D, Ngai J, Speed TP*, et al*. Normalization of RNA-seq data using factor analysis of control genes or samples. *Nat Biotechnol.* 2014;**32** (suppl 9):896-902.

11 McCarthy DJ, Chen Y, Smyth GK. Differential expression analysis of multifactor RNA-Seq experiments with respect to biological variation. *Nucleic Acids Res.* 2012;**40** (suppl 10):4288-4297.

12 Robinson MD, McCarthy DJ, Smyth GK. edgeR: a Bioconductor package for differential expression analysis of digital gene expression data. *Bioinformatics.* 2010;**26** (suppl 1):139-140.

13 Johnson M, Zaretskaya I, Raytselis Y*, et al*. NCBI BLAST: a better web interface. *Nucleic Acids Res.* 2008;**36** (suppl Web Server issue):W5-9.

14 Dunbar J, Deane CM. ANARCI: antigen receptor numbering and receptor classification. *Bioinformatics.* 2016;**32** (suppl 2):298-300.

Brandsma C-A, *et al. Thorax* 2020; 75:180–183. doi: 10.1136/thoraxjnl-2019-213200

15 Ye J, Ma N, Madden TL, *et al*. IgBLAST: an immunoglobulin variable domain sequence analysis tool. *Nucleic Acids Res.* 2013;**41** (suppl Web Server issue):W34-40.

16 Reimand J, Arak T, Adler P, *et al*. g:Profiler-a web server for functional interpretation of gene lists (2016 update). *Nucleic Acids Res.* 2016;**44** (suppl W1):W83-9.

17 Csardi G, Nepusz T. The igraph software package for complex network research. *InterJournal Complex Systems.* 2006;**1695**.

18 Szklarczyk D, Morris JH, Cook H, *et al*. The STRING database in 2017: quality-controlled protein-protein association networks, made broadly accessible. *Nucleic Acids Res.* 2017;**45** (suppl D1):D362-D368.

19 Kim WJ, Lim JH, Lee JS, *et al*. Comprehensive Analysis of Transcriptome Sequencing Data in the Lung Tissues of COPD Subjects. *Int J Genomics.* 2015;**2015**:206937.

Brandsma C-A, *et al. Thorax* 2020; 75:180–183. doi: 10.1136/thoraxjnl-2019-213200

5 MS/MS spectra and identification parameters of 61 non-reference peptides

Peptide Sequence	Gene	Dataset	Sample	Score	ScanID
ISYGPDWKDFYVVEPLAFEGTPEQK	HEXA	1D25	Control3	120.51	26144

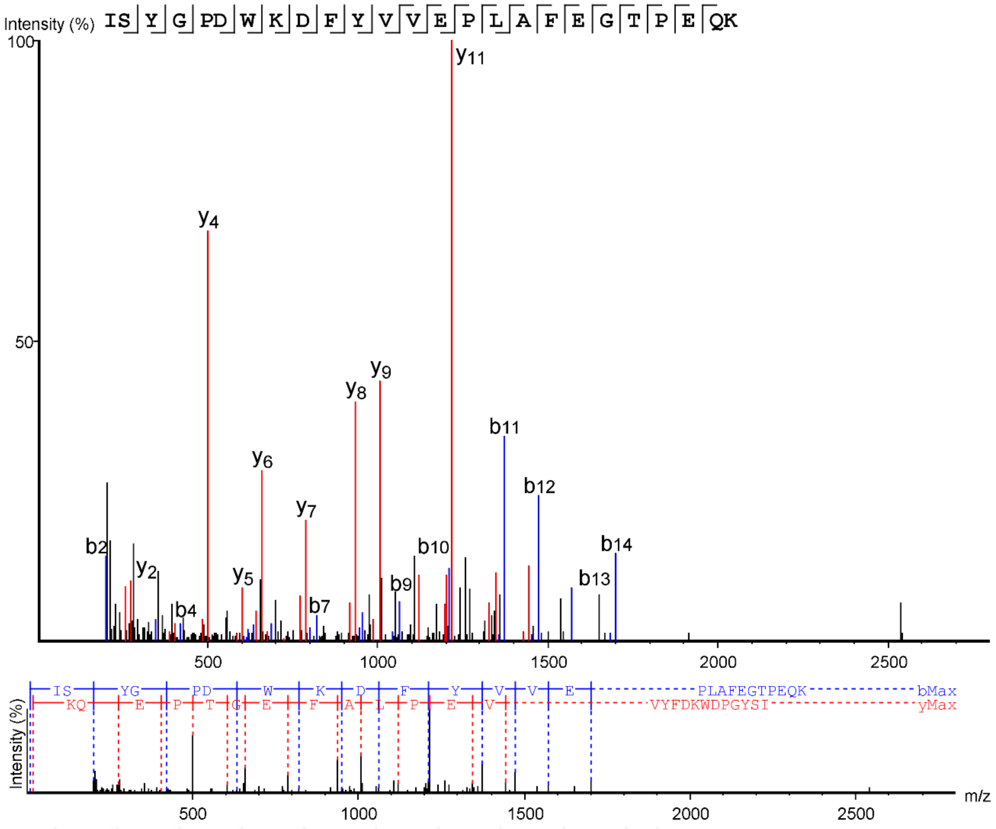

#	b	b-H2O	b-NH3	b (2+)	Seq	y	y-H2O	y-NH3	y (2+)	#
1	114.09	96.08	97.06	57.55	I					25
2	201.12	183.11	184.10	101.06	S	2802.33	2784.32	2785.30	1401.66	24
3	364.19	346.18	347.16	182.59	Y	2715.30	2697.29	2698.27	1358.15	23
4	421.21	403.20	404.18	211.10	G	2552.23	2534.22	2535.21	1276.62	22
5	518.26	500.25	501.23	259.63	P	2495.21	2477.20	2478.19	1248.11	21
6	633.29	615.27	616.28	317.14	D	2398.16	2380.15	2381.13	1199.58	20
7	819.37	801.36	802.34	410.18	W	2283.13	2265.12	2266.11	1142.07	19
8	947.46	929.45	930.44	474.23	K	2097.05	2079.04	2080.03	1049.03	18
9	1062.49	1044.47	1045.46	531.74	D	1968.96	1950.95	1951.93	984.98	17
10	1209.56	1191.55	1192.53	605.28	F	1853.93	1835.92	1836.90	927.47	16
11	1372.62	1354.61	1355.61	686.81	Y	1706.86	1688.85	1689.84	853.93	15
12	1471.69	1453.68	1454.66	736.34	V	1543.80	1525.79	1526.77	772.40	14
13	1570.76	1552.75	1553.73	785.88	V	1444.73	1426.72	1427.71	722.85	13
14	1699.80	1681.79	1682.77	850.40	E	1345.66	1327.65	1328.64	673.32	12
15	1796.85	1778.84	1779.83	898.93	P	1216.62	1198.62	1199.61	608.81	11
16	1909.94	1891.93	1892.91	955.46	L	1119.57	1101.56	1102.54	560.28	10
17	1980.97	1962.96	1963.95	990.99	A	1006.48	988.47	989.46	503.74	9
18	2128.04	2110.03	2111.02	1064.52	F	935.45	917.44	918.42	468.22	8
19	2257.09	2239.08	2240.06	1129.04	E	788.38	770.37	771.35	394.69	7
20	2314.11	2296.10	2297.08	1157.55	G	659.34	641.33	642.31	330.17	6
21	2415.15	2397.14	2398.13	1208.08	T	602.32	584.30	585.27	301.66	5
22	2512.21	2494.20	2495.18	1256.60	P	501.27	483.25	484.24	251.13	4
23	2641.25	2623.24	2624.22	1321.13	E	404.21	386.20	387.19	202.61	3
24	2769.31	2751.30	2752.28	1385.15	Q	275.17	257.16	258.14	138.09	2
25					K	147.11	129.10	130.09	74.06	1

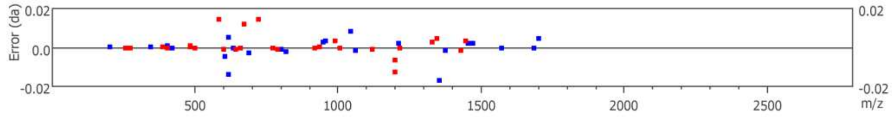

Brandsma C-A, *et al. Thorax* 2020; 75:180–183. doi: 10.1136/thoraxjnl-2019-213200

Peptide Sequence	Gene	Dataset	Sample	Score	ScanID
QTLEKENTDLAGELR	MYH11	1D50	Control9	77.52	12943

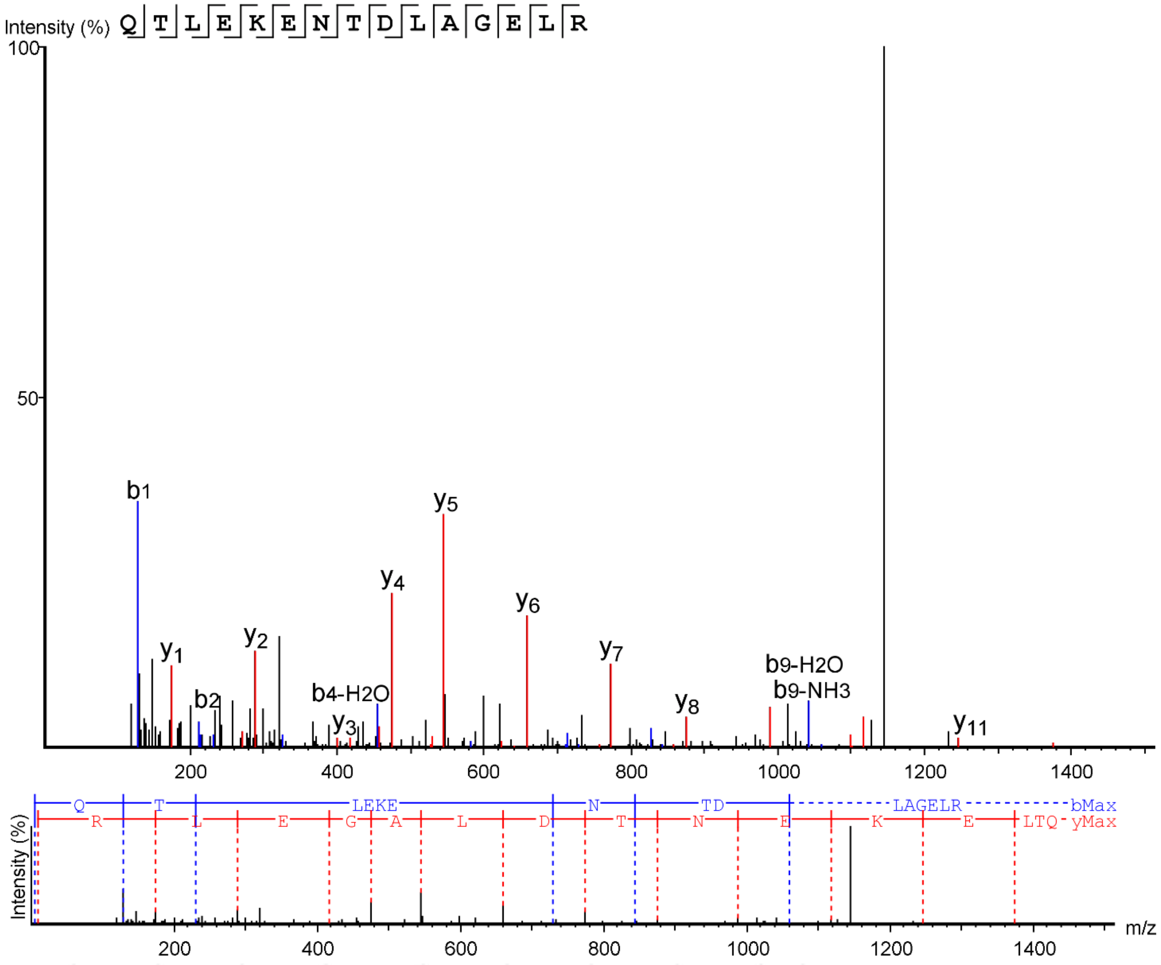

#	b	b-H2O	b-NH3	b (2+)	Seq	y	y-H2O	y-NH3	y (2+)	#
1	129.07	111.06	112.04	65.03	Q					15
2	230.11	212.10	213.09	115.56	T	1588.82	1570.81	1571.79	794.91	14
3	343.20	325.19	326.17	172.10	L	1487.77	1469.76	1470.74	744.38	13
4	472.24	454.23	455.21	236.62	E	1374.69	1356.68	1357.66	687.84	12
5	600.34	582.32	583.31	300.67	K	1245.64	1227.63	1228.62	623.32	11
6	729.38	711.37	712.35	365.19	E	1117.55	1099.54	1100.53	559.27	10
7	843.42	825.41	826.39	422.21	N	988.51	970.50	971.48	494.75	9
8	944.47	926.46	927.44	472.73	T	874.46	856.45	857.44	437.73	8
9	1059.50	1041.48	1042.47	530.25	D	773.41	755.40	756.39	387.21	7
10	1172.58	1154.57	1155.55	586.79	L	658.39	640.38	641.36	329.69	6
11	1243.62	1225.61	1226.59	622.31	A	545.30	527.30	528.28	273.15	5
12	1300.64	1282.63	1283.61	650.82	G	474.27	456.26	457.24	237.63	4
13	1429.68	1411.67	1412.65	715.34	E	417.25	399.23	400.22	209.12	3
14	1542.77	1524.75	1525.74	771.88	L	288.20	270.19	271.18	144.60	2
15					R	175.12	157.11	158.09	88.06	1

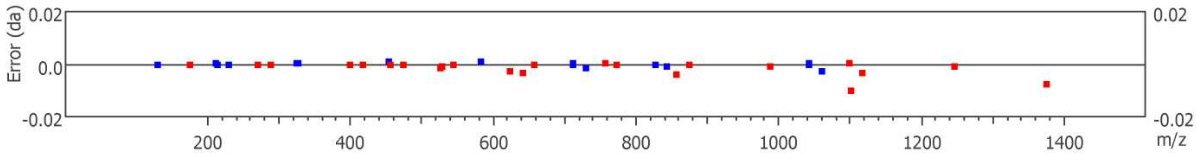

Peptide Sequence	Gene	Dataset	Sample	Score	ScanID
ASQSVSSNYLAWYQQKPGQAPR	IGKV3-20	2D25	Control3	105.88	12086

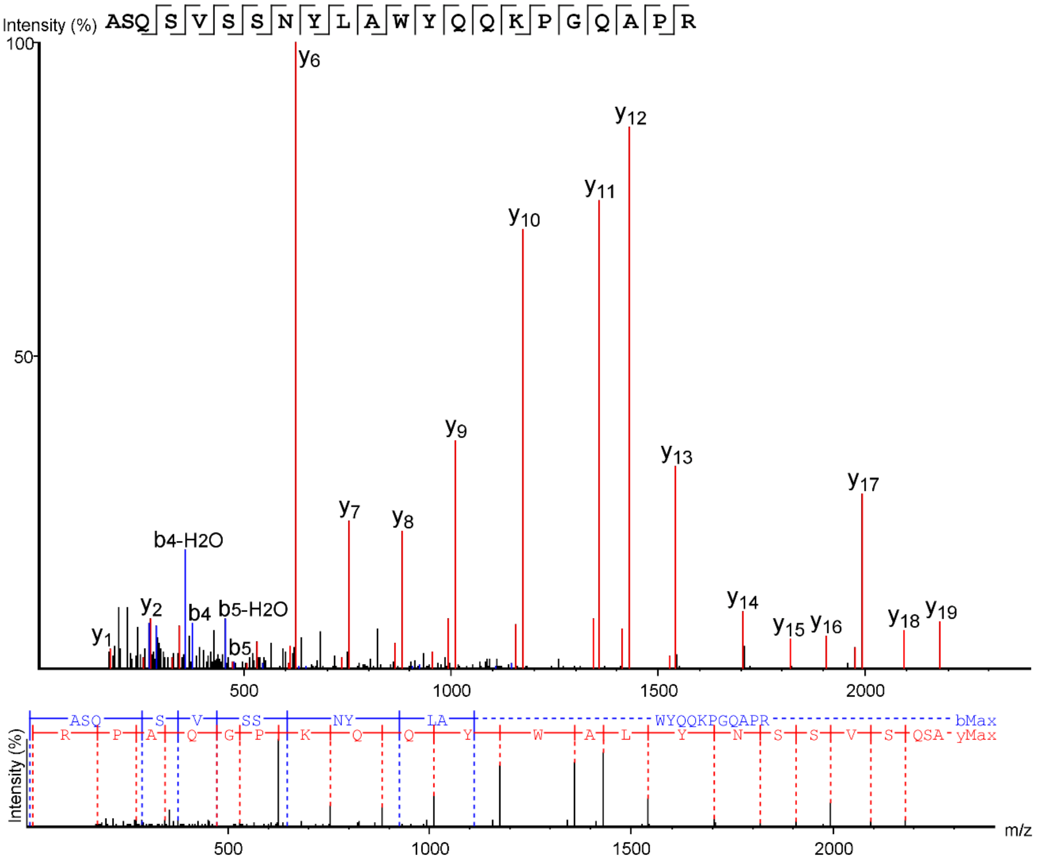

#	b	b-H2O	b-NH3	b (2+)	Seq	y	y-H2O	y-NH3	y (2+)	#
1	72.04	54.03	55.02	36.52	A					22
2	159.08	141.07	142.05	80.04	S	2395.18	2377.17	2378.15	1198.09	21
3	287.13	269.12	270.11	144.07	Q	2308.15	2290.14	2291.12	1154.57	20
4	374.17	356.16	357.14	187.58	S	2180.09	2162.08	2163.06	1090.54	19
5	473.24	455.22	456.21	237.12	V	2093.06	2075.05	2076.03	1047.03	18
6	560.27	542.26	543.24	280.63	S	1994.00	1975.98	1976.97	997.49	17
7	647.30	629.29	630.27	324.15	S	1906.96	1888.95	1889.93	953.98	16
8	761.34	743.33	744.32	381.17	N	1819.92	1801.91	1802.90	910.46	15
9	924.40	906.40	907.38	462.70	Y	1705.89	1687.87	1688.85	853.44	14
10	1037.49	1019.48	1020.46	519.25	L	1542.82	1524.81	1525.79	771.91	13
11	1108.54	1090.52	1091.50	554.76	A	1429.73	1411.72	1412.71	715.37	12
12	1294.61	1276.60	1277.58	647.80	W	1358.70	1340.69	1341.67	679.85	11
13	1457.67	1439.66	1440.64	729.34	Y	1172.62	1154.61	1155.59	586.81	10
14	1585.73	1567.72	1568.70	793.36	Q	1009.55	991.54	992.53	505.27	9
15	1713.79	1695.78	1696.76	857.39	Q	881.49	863.49	864.47	441.25	8
16	1841.88	1823.87	1824.86	921.45	K	753.44	735.43	736.41	377.22	7
17	1938.93	1920.92	1921.91	969.97	P	625.34	607.33	608.31	313.17	6
18	1995.96	1977.95	1978.93	998.48	G	528.29	510.28	511.26	264.64	5
19	2124.01	2106.00	2106.99	1062.51	Q	471.26	453.26	454.24	236.13	4
20	2195.05	2177.04	2178.02	1098.03	A	343.21	325.20	326.18	172.10	3
21	2292.10	2274.09	2275.08	1146.56	P	272.17	254.16	255.15	136.59	2
22					R	175.12	157.11	158.09	88.06	1

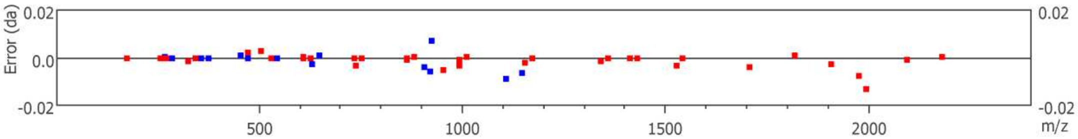

Peptide Sequence	Gene	Dataset	Sample	Score	ScanID
GGGAGFISGLTYLELDNPAGNK	C7	1D50	Control3	108.34	27083

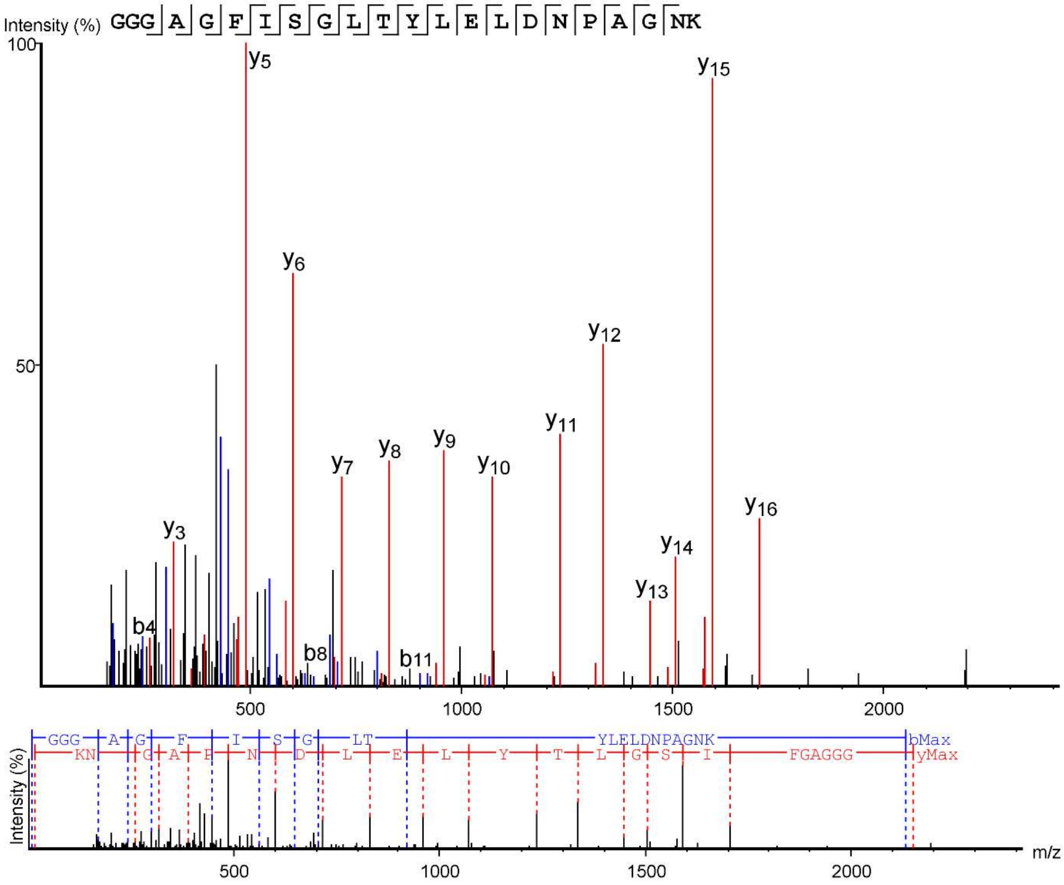

#	b	b-H2O	b-NH3	b (2+)	Seq	y	y-H2O	y-NH3	y (2+)	#
1	58.03	40.02	41.00	29.51	G					22
2	115.05	97.04	98.02	58.03	G	2094.05	2076.04	2077.02	1047.53	21
3	172.07	154.06	155.05	86.54	G	2037.03	2019.02	2020.00	1019.01	20
4	243.11	225.10	226.08	122.05	A	1980.01	1962.00	1962.98	990.50	19
5	300.13	282.12	283.10	150.57	G	1908.97	1890.96	1891.94	954.99	18
6	447.20	429.19	430.17	224.10	F	1851.95	1833.94	1834.92	926.47	17
7	560.28	542.27	543.26	280.64	I	1704.88	1686.87	1687.85	852.94	16
8	647.31	629.30	630.29	324.16	S	1591.80	1573.79	1574.78	796.40	15
9	704.34	686.33	687.31	352.67	G	1504.76	1486.75	1487.74	752.88	14
10	817.42	799.41	800.39	409.21	L	1447.74	1429.73	1430.72	724.37	13
11	918.47	900.46	901.46	459.73	T	1334.66	1316.64	1317.65	667.83	12
12	1081.53	1063.52	1064.50	541.27	Y	1233.61	1215.60	1216.59	617.31	11
13	1194.62	1176.61	1177.59	597.81	L	1070.55	1052.54	1053.53	535.77	10
14	1323.66	1305.65	1306.63	662.33	E	957.46	939.44	940.45	479.23	9
15	1436.74	1418.73	1419.72	718.87	L	828.42	810.40	811.38	414.71	8
16	1551.77	1533.76	1534.74	776.38	D	715.34	697.33	698.31	358.17	7
17	1665.81	1647.80	1648.79	833.41	N	600.31	582.30	583.28	300.65	6
18	1762.87	1744.85	1745.84	881.93	P	486.27	468.26	469.24	243.63	5
19	1833.90	1815.89	1816.88	917.45	A	389.21	371.20	372.19	195.11	4
20	1890.92	1872.91	1873.90	945.96	G	318.18	300.16	301.15	159.59	3
21	2004.97	1986.96	1987.94	1002.98	N	261.16	243.15	244.13	131.08	2
22					K	147.11	129.10	130.09	74.06	1

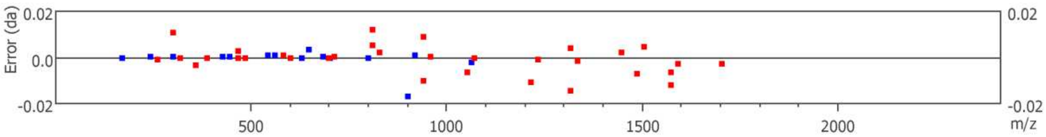

Brandsma C-A, *et al. Thorax* 2020; 75:180–183. doi: 10.1136/thoraxjnl-2019-213200

Peptide Sequence	Gene	Dataset	Sample	Score	ScanID
LLIYWASAR	IGKV4-1	1D25	Control2	68.41	18493

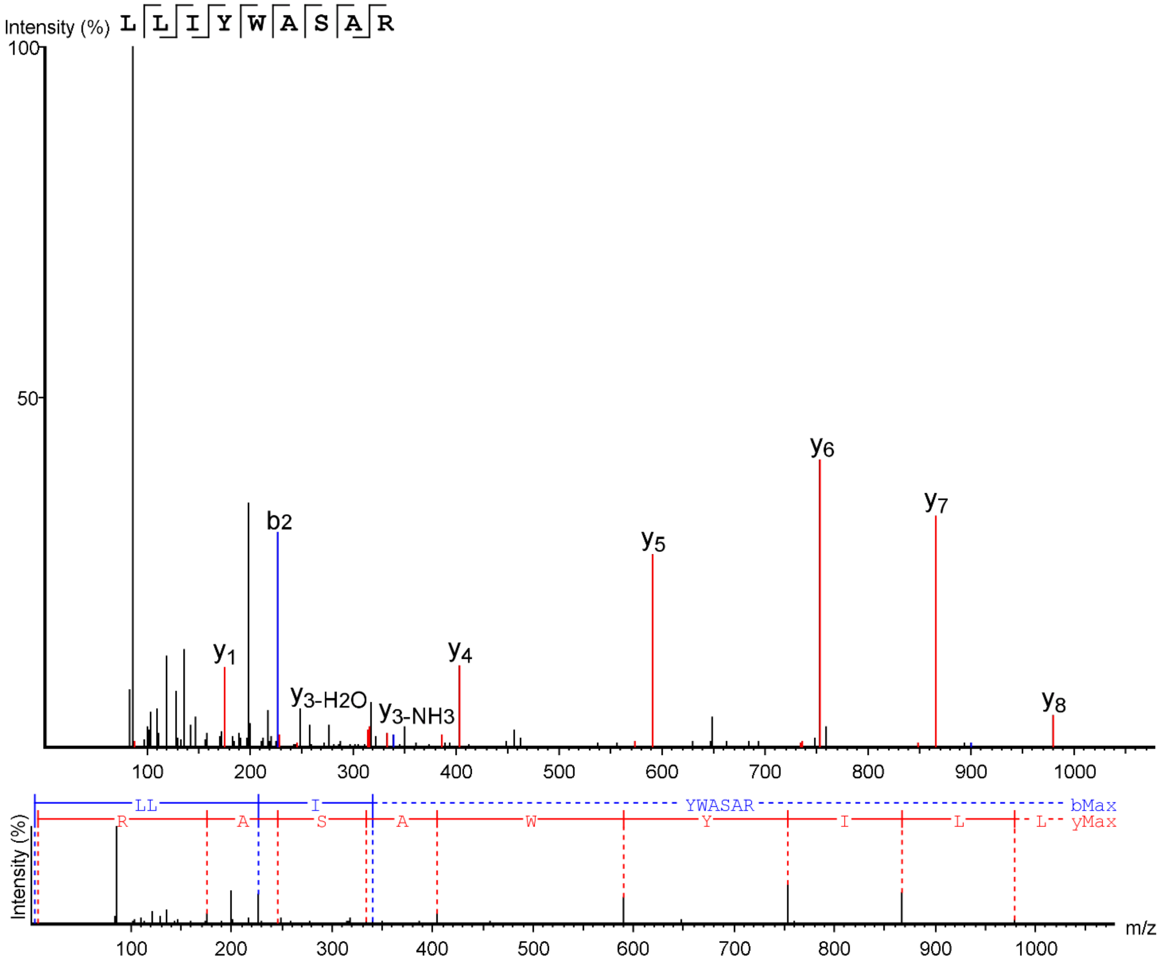

#	b	b-H2O	b-NH3	b (2+)	Seq	y	y-H2O	y-NH3	y (2+)	#
1	114.09	96.08	97.06	57.55	L					9
2	227.17	209.17	210.15	114.09	L	979.53	961.53	962.51	490.27	8
3	340.26	322.25	323.23	170.63	I	866.45	848.44	849.42	433.73	7
4	503.32	485.31	486.30	252.16	Y	753.37	735.36	736.34	377.18	6
5	689.40	671.39	672.38	345.20	W	590.30	572.29	573.28	295.65	5
6	760.44	742.43	743.41	380.72	A	404.22	386.22	387.20	202.61	4
7	847.47	829.46	830.44	424.24	S	333.19	315.18	316.16	167.09	3
8	918.51	900.50	901.48	459.75	A	246.16	228.15	229.12	123.58	2
9					R	175.12	157.11	158.09	88.04	1

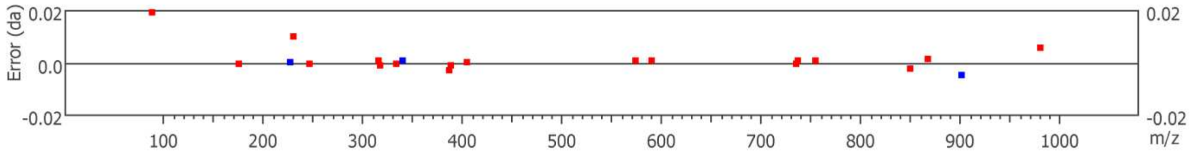

Brandsma C-A, *et al. Thorax* 2020; 75:180–183. doi: 10.1136/thoraxjnl-2019-213200

Peptide Sequence	Gene	Dataset	Sample	Score	ScanID
LHSLTQAKEESEK	RRBP1	2D25	Control10	110.58	2122

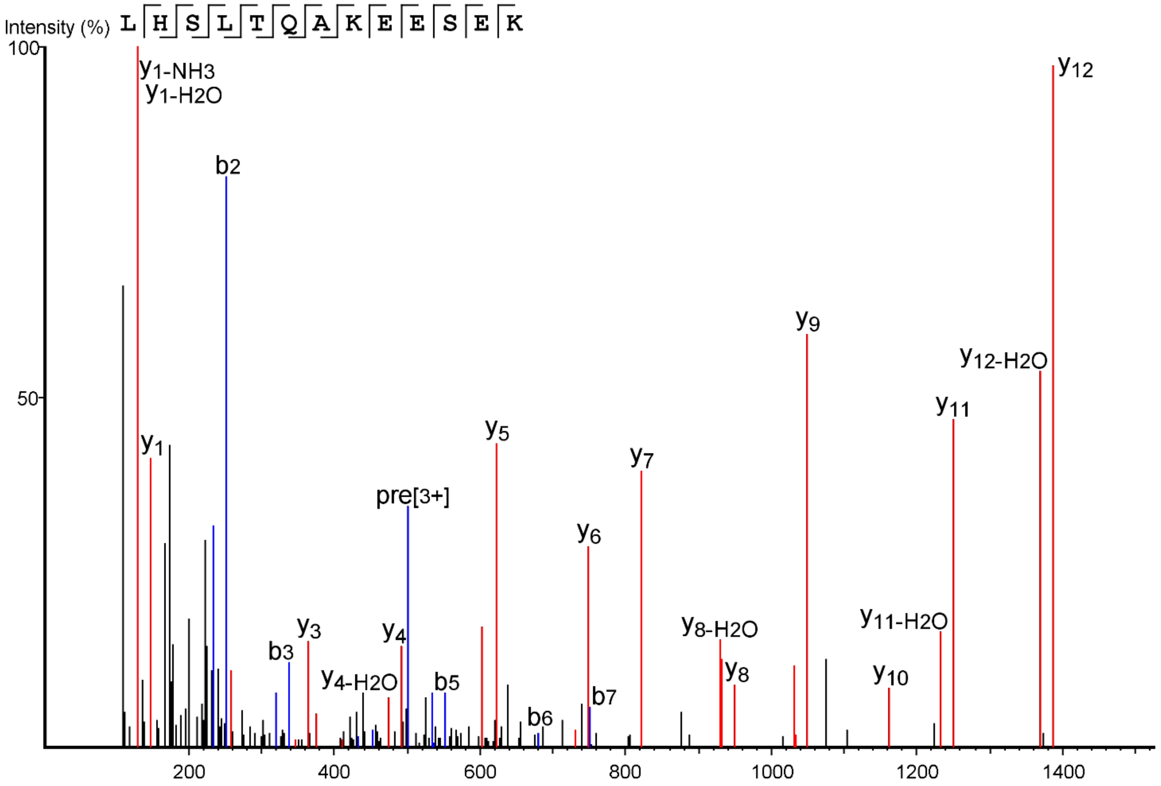

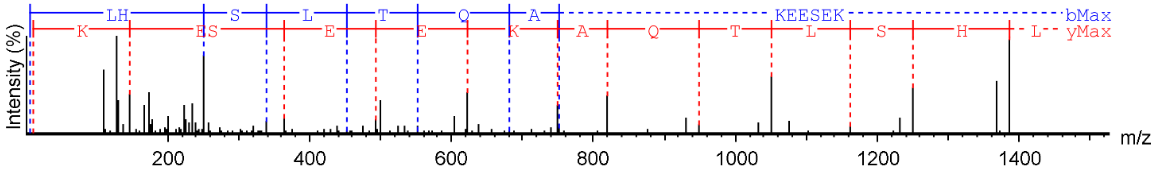

#	b	b-H2O	b-NH3	b (2+)	Seq	y	y-H2O	y-NH3	y (2+)	#
1	114.09	96.08	97.06	57.55	L					13
2	251.15	233.14	234.12	126.08	H	1386.68	1368.67	1369.66	693.84	12
3	338.18	320.17	321.16	169.59	S	1249.63	1231.62	1232.60	625.31	11
4	451.26	433.26	434.24	226.13	L	1162.59	1144.58	1145.57	581.80	10
5	552.31	534.30	535.31	276.66	T	1049.51	1031.50	1032.47	525.26	9
6	680.37	662.36	663.35	340.69	Q	948.46	930.45	931.44	474.73	8
7	751.41	733.40	734.38	376.21	A	820.40	802.39	803.38	410.71	7
8	879.51	861.49	862.48	440.25	K	749.37	731.35	732.34	375.19	6
9	1008.55	990.54	991.52	504.77	E	621.27	603.26	604.25	311.14	5
10	1137.59	1119.58	1120.56	569.30	E	492.23	474.22	475.20	246.61	4
11	1224.62	1206.61	1207.60	612.81	S	363.19	345.18	346.16	182.09	3
12	1353.67	1335.65	1336.64	677.33	E	276.16	258.14	259.13	138.58	2
13					K	147.11	129.10	130.09	74.06	1

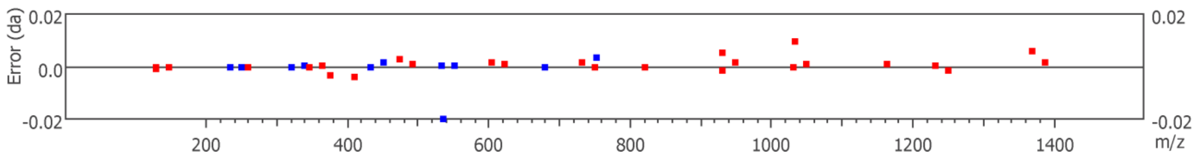

Brandsma C-A, *et al. Thorax* 2020; 75:180–183. doi: 10.1136/thoraxjnl-2019-213200

Peptide Sequence	Gene	Dataset	Sample	Score	ScanID
LLEDLR	PDE4DIP	1D25	Control8	33.62	7532

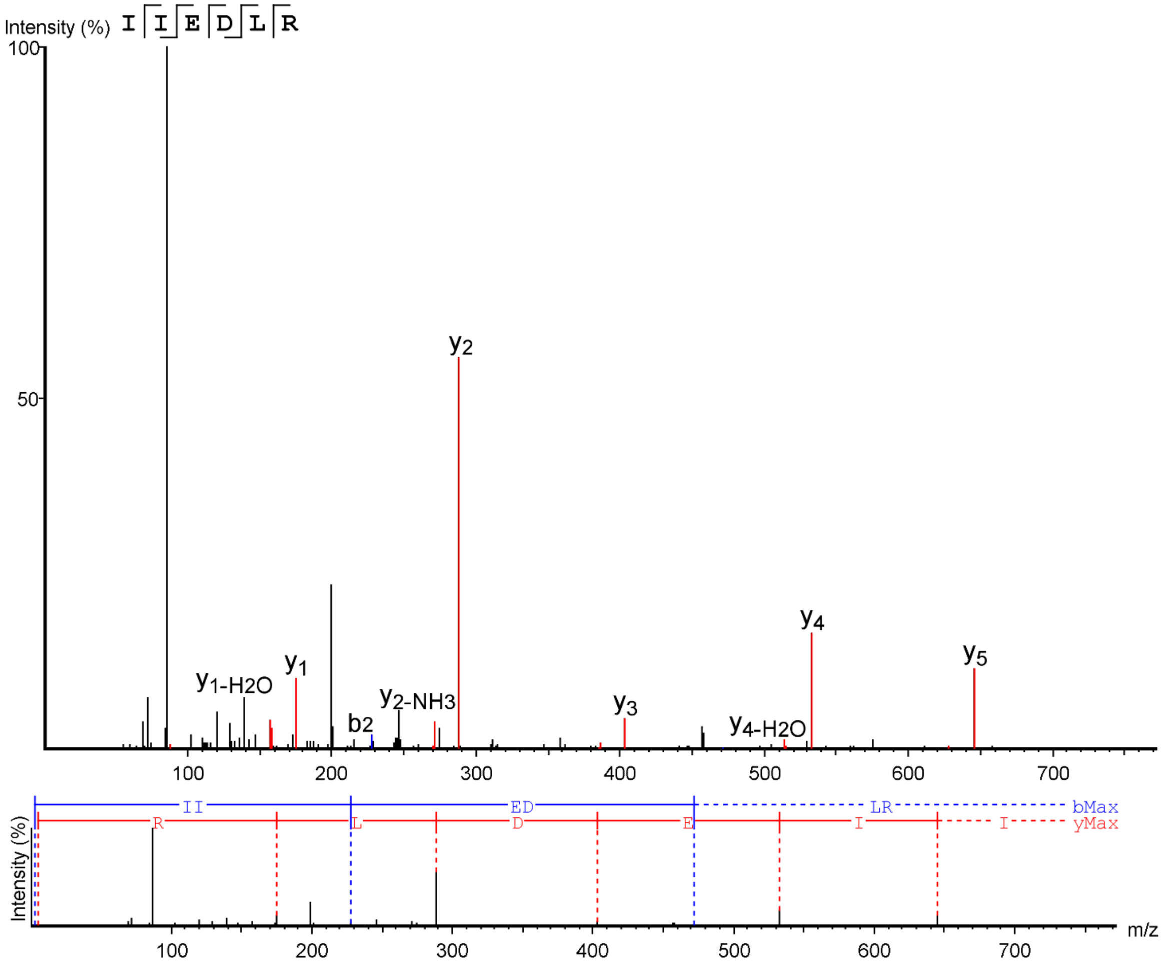

#	b	b-H2O	b-NH3	b (2+)	Seq	y	y-H2O	y-NH3	y (2+)	#
1	114.09	96.08	97.06	57.55	I					6
2	227.18	209.17	210.15	114.09	I	645.36	627.35	628.33	323.18	5
3	356.22	338.21	339.19	178.61	E	532.27	514.26	515.25	266.64	4
4	471.24	453.23	454.22	236.12	D	403.23	385.22	386.20	202.11	3
5	584.33	566.32	567.30	292.66	L	288.20	270.19	271.18	144.60	2
6					R	175.12	157.11	158.09	88.04	1

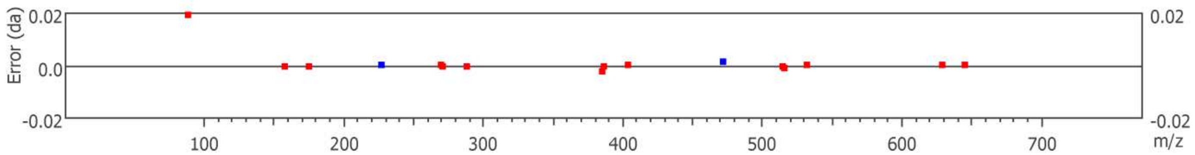

Brandsma C-A, *et al. Thorax* 2020; 75:180–183. doi: 10.1136/thoraxjnl-2019-213200

Peptide Sequence	Gene	Dataset	Sample	Score	ScanID
NSLFLQMNSLR	IGHV3-21	1D50	Control3	76.68	22081

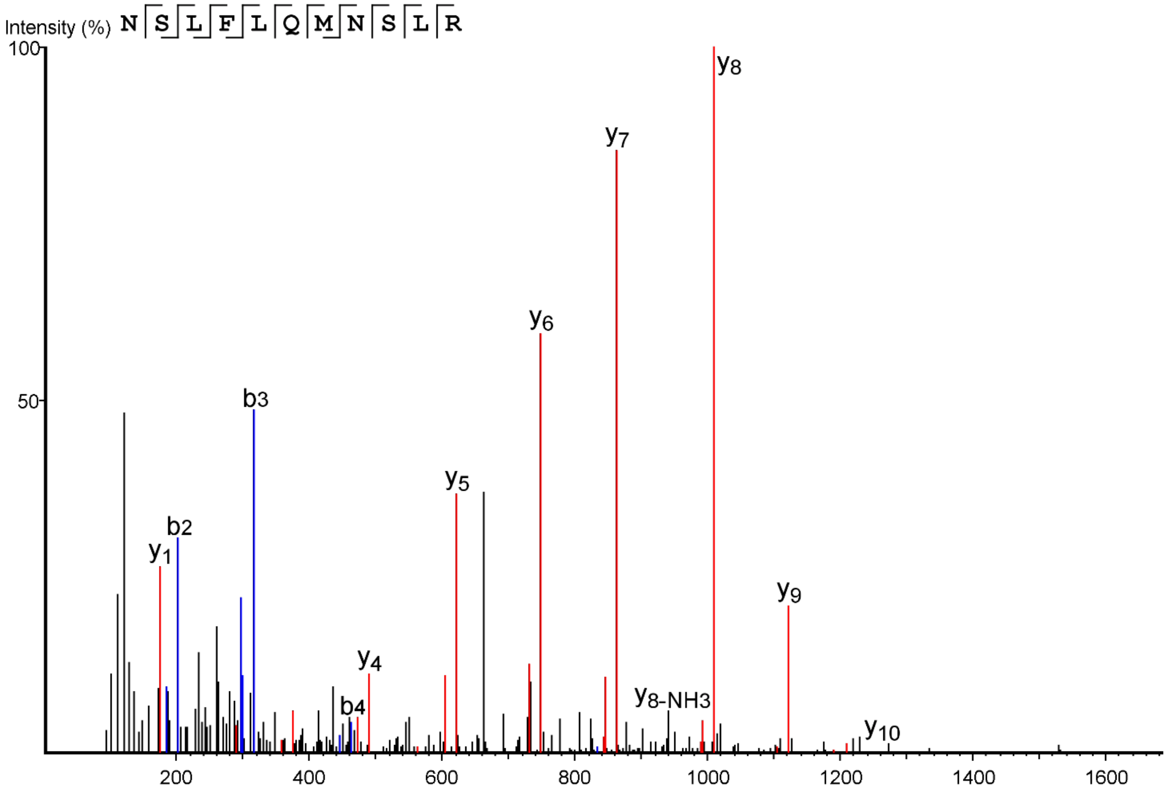

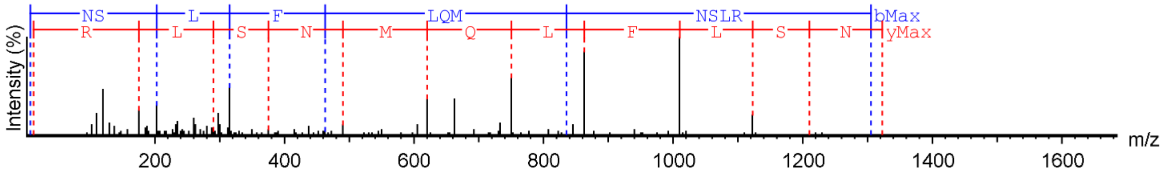

#	b	b-H2O	b-NH3	b (2+)	Seq	y	y-H2O	y-NH3	y (2+)	#
1	115.05	97.04	98.02	58.03	N					11
2	202.08	184.07	185.06	101.54	S	1208.64	1190.63	1191.62	604.82	10
3	315.17	297.16	298.14	158.08	L	1121.61	1103.60	1104.58	561.30	9
4	462.23	444.22	445.21	231.62	F	1008.53	990.52	991.50	504.76	8
5	575.32	557.31	558.29	288.16	L	861.46	843.46	844.43	431.23	7
6	703.38	685.37	686.35	352.19	Q	748.38	730.36	731.35	374.69	6
7	834.40	816.41	817.39	417.71	M	620.32	602.31	603.29	310.66	5
8	948.46	930.45	931.43	474.73	N	489.28	471.27	472.25	245.14	4
9	1035.49	1017.48	1018.47	518.25	S	375.23	357.22	358.21	188.12	3
10	1148.58	1130.57	1131.55	574.79	L	288.20	270.19	271.18	144.60	2
11					R	175.12	157.11	158.09	88.06	1

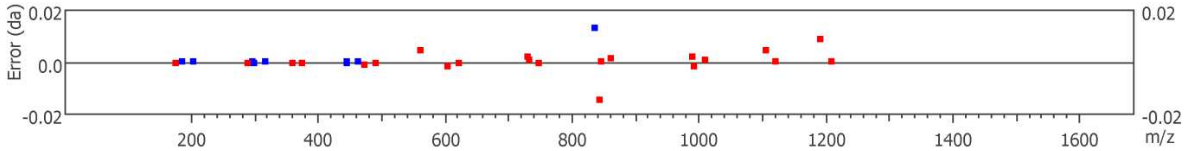

Brandsma C-A, *et al. Thorax* 2020; 75:180–183. doi: 10.1136/thoraxjnl-2019-213200

Peptide Sequence	Gene	Dataset	Sample	Score	ScanID
ISNSAAYSGSVAPANSALGQTQPSDQDTLVQR	PDLIM5	2D25	Control6	110.84	12448

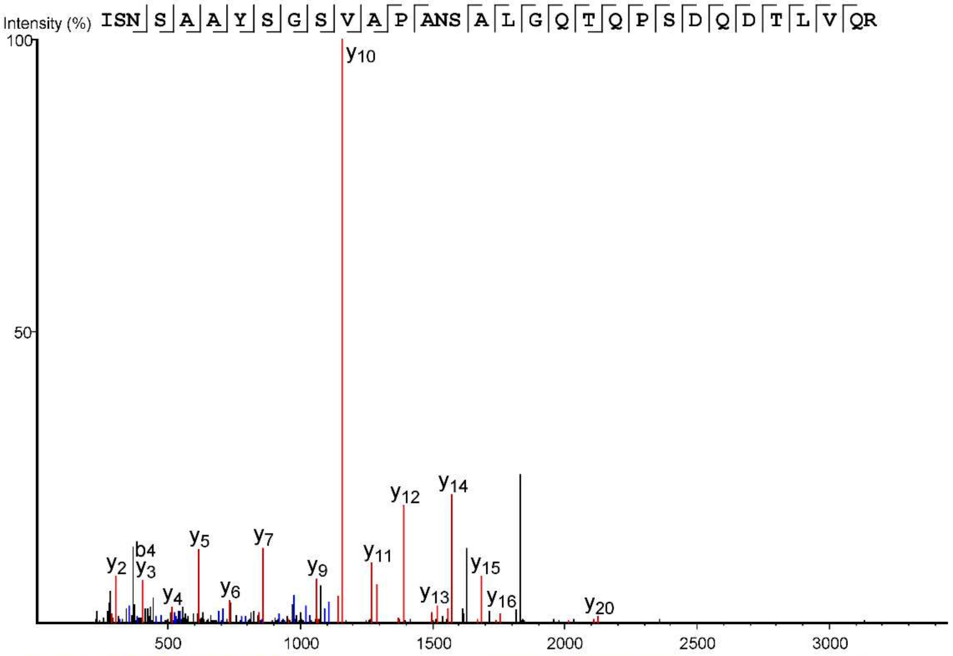

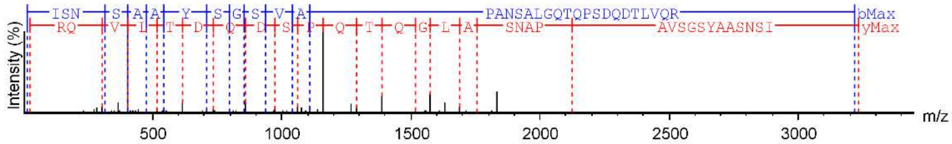

#	b	b-H2O	b-NH3	b (2+)	Seq	y	y-H2O	y-NH3	y (2+)	#
1	114.09	96.08	97.06	57.55	I					32
2	201.12	183.11	184.10	101.06	S	3120.48	3102.47	3103.46	1560.74	31
3	315.17	297.16	298.14	158.08	N	3033.45	3015.44	3016.42	1517.23	30
4	402.20	384.19	385.17	201.60	S	2919.41	2901.40	2902.38	1460.20	29
5	473.24	455.22	456.21	237.12	A	2832.38	2814.37	2815.35	1416.69	28
6	544.27	526.26	527.25	272.64	A	2761.34	2743.33	2744.31	1381.17	27
7	707.33	689.33	690.31	354.18	Y	2690.30	2672.29	2673.27	1345.65	26
8	794.37	776.36	777.34	397.68	S	2527.24	2509.23	2510.21	1264.12	25
9	851.38	833.38	834.36	426.19	G	2440.21	2422.20	2423.18	1220.60	24
10	938.42	920.41	921.39	469.71	S	2383.18	2365.17	2366.16	1192.09	23
11	1037.49	1019.48	1020.46	519.26	V	2296.15	2278.14	2279.13	1148.58	22
12	1108.53	1090.52	1091.51	554.76	A	2197.08	2179.07	2180.06	1099.04	21
13	1205.58	1187.57	1188.55	603.29	P	2126.04	2108.03	2109.02	1063.52	20
14	1276.62	1258.61	1259.59	638.81	A	2028.99	2010.98	2011.95	1015.00	19
15	1390.66	1372.65	1373.63	695.83	N	1957.96	1939.95	1940.93	979.48	18
16	1477.69	1459.68	1460.67	739.35	S	1843.91	1825.90	1826.89	922.46	17
17	1548.73	1530.72	1531.70	774.86	A	1756.88	1738.87	1739.85	878.94	16
18	1661.81	1643.80	1644.79	831.41	L	1685.84	1667.83	1668.83	843.43	15
19	1718.83	1700.82	1701.81	859.92	G	1572.76	1554.75	1555.75	786.88	14
20	1846.89	1828.88	1829.87	923.95	Q	1515.74	1497.72	1498.72	758.37	13
21	1947.94	1929.93	1930.91	974.49	T	1387.68	1369.66	1370.67	694.34	12
22	2076.00	2057.99	2058.97	1038.50	Q	1286.63	1268.63	1269.61	643.82	11
23	2173.05	2155.04	2156.03	1087.03	P	1158.57	1140.56	1141.56	579.79	10
24	2260.08	2242.07	2243.06	1130.54	S	1061.52	1043.51	1044.50	531.26	9
25	2375.11	2357.10	2358.08	1188.06	D	974.49	956.49	957.46	487.75	8
26	2503.17	2485.16	2486.14	1252.08	Q	859.46	841.44	842.44	430.23	7
27	2618.20	2600.19	2601.17	1309.60	D	731.41	713.39	714.38	366.20	6
28	2719.24	2701.23	2702.22	1360.12	T	616.38	598.37	599.35	308.69	5
29	2832.33	2814.32	2815.30	1416.66	L	515.33	497.32	498.30	258.17	4
30	2931.40	2913.39	2914.37	1466.20	V	402.25	384.24	385.22	201.62	3
31	3059.46	3041.45	3042.43	1530.23	Q	303.18	285.17	286.15	152.09	2
32					R	175.12	157.11	158.09	88.06	1

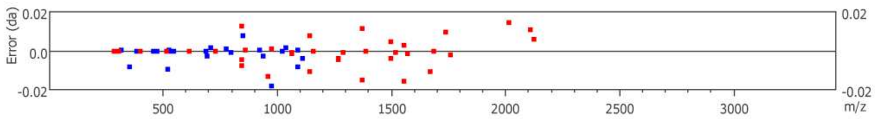

Peptide Sequence	Gene	Dataset	Sample	Score	ScanID
NVSSNPCHEAVGIK	SORBS1	2D25	COPD2	56.53	4714

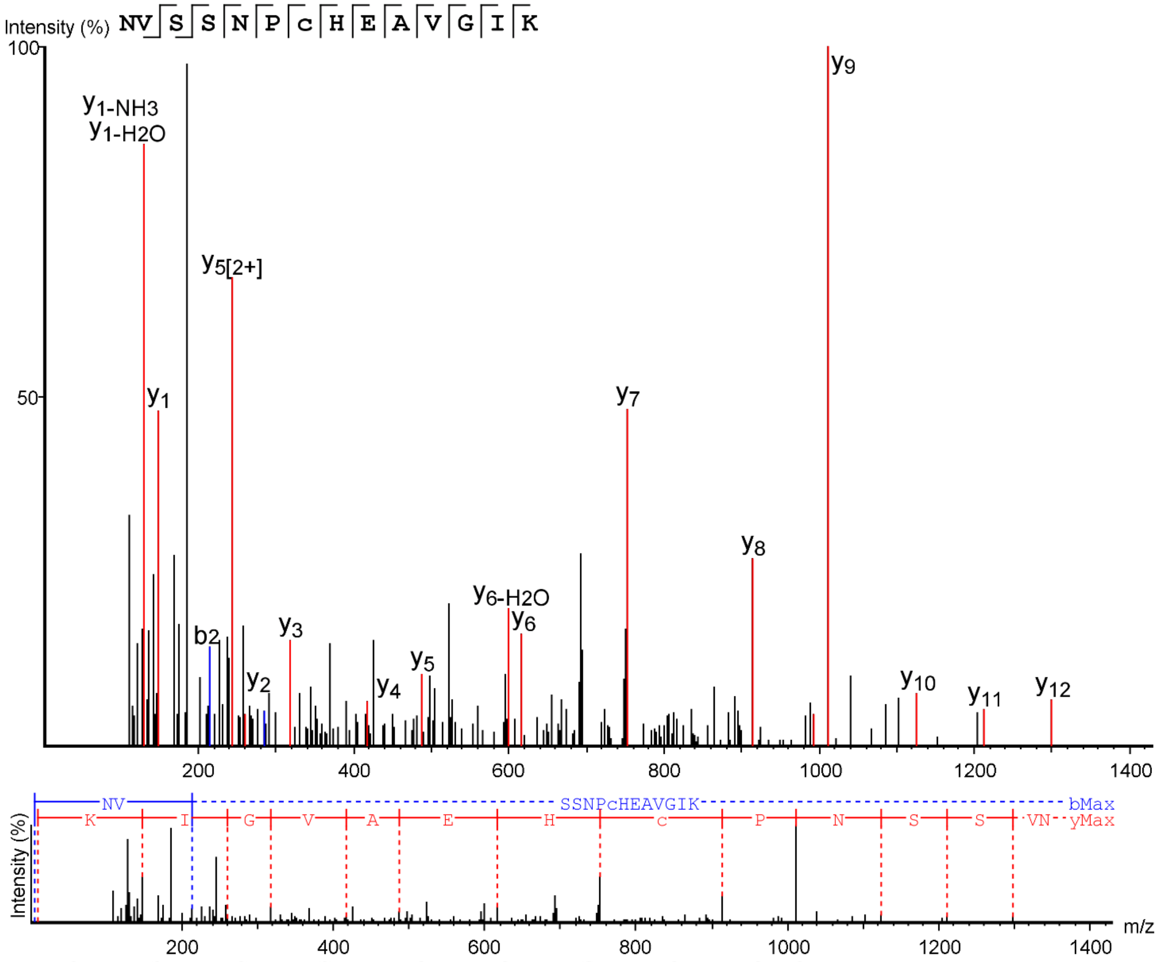

#	b	b-H2O	b-NH3	b (2+)	Seq	y	y-H2O	y-NH3	y (2+)	#
1	115.05	97.04	98.02	58.03	N					14
2	214.12	196.11	197.09	107.56	V	1397.68	1379.67	1380.66	699.34	13
3	301.15	283.14	284.12	151.08	S	1298.61	1280.61	1281.59	649.81	12
4	388.18	370.17	371.16	194.59	S	1211.58	1193.57	1194.56	606.29	11
5	502.23	484.22	485.20	251.61	N	1124.54	1106.54	1107.52	562.78	10
6	599.28	581.27	582.25	300.14	P	1010.51	992.49	993.48	505.75	9
7	759.31	741.30	742.28	380.15	C(+57.02)	913.45	895.45	896.43	457.23	8
8	896.37	878.36	879.34	448.68	H	753.42	735.41	736.40	377.21	7
9	1025.41	1007.40	1008.38	513.21	E	616.37	598.35	599.34	308.68	6
10	1096.45	1078.44	1079.42	548.72	A	487.32	469.31	470.30	244.17	5
11	1195.52	1177.51	1178.49	598.26	V	416.29	398.28	399.26	208.64	4
12	1252.54	1234.53	1235.51	626.77	G	317.22	299.21	300.19	159.11	3
13	1365.62	1347.61	1348.60	683.31	I	260.20	242.19	243.17	130.60	2
14					K	147.11	129.10	130.09	74.06	1

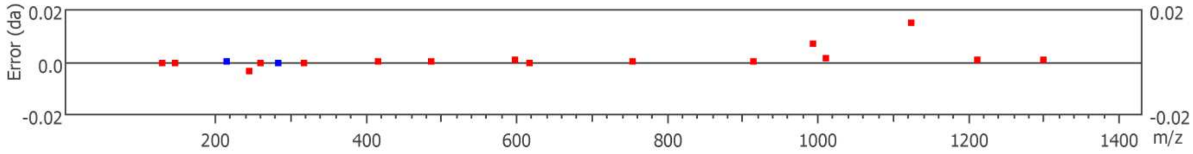

Brandsma C-A, *et al. Thorax* 2020; 75:180–183. doi: 10.1136/thoraxjnl-2019-213200

Peptide Sequence	Gene	Dataset	Sample	Score	ScanID
ADSQDAGQETEKEGEDPQASAQDETPITSAK	AKAP12	2D25	COPD5	134.83	7488

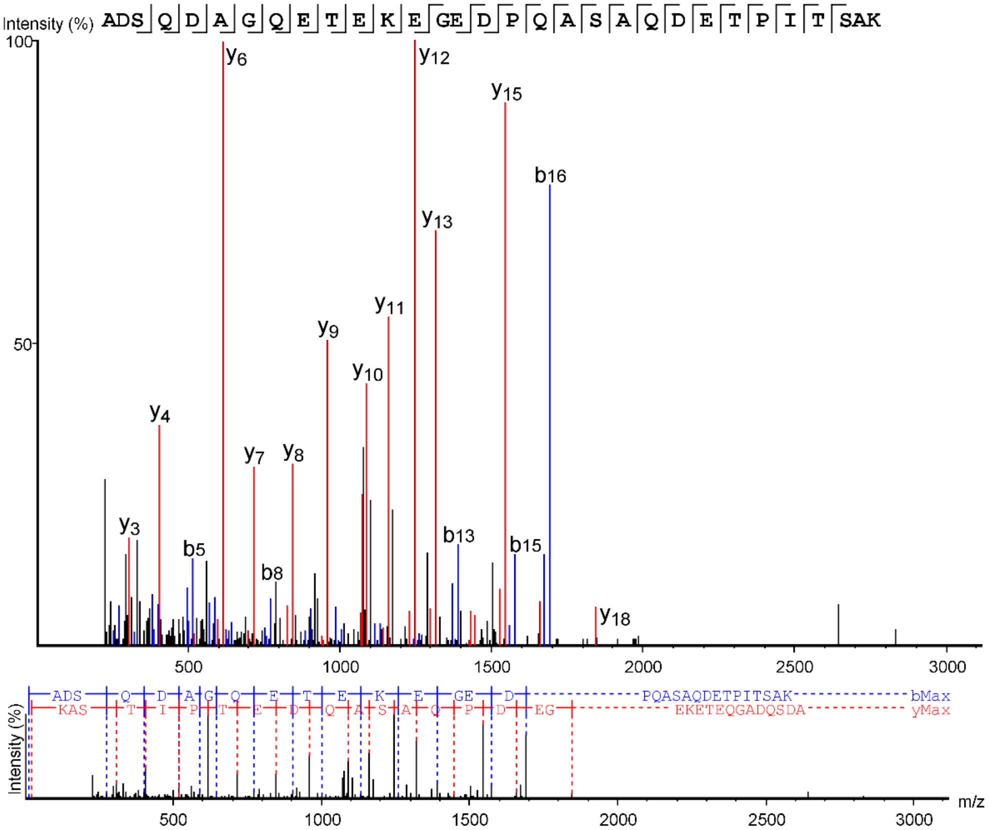

#	b	b-H2O	b-NH3	b (2+)	Seq	y	y-H2O	y-NH3	y (2+)	#
1	72.04	54.03	55.02	36.52	A					31
2	187.07	169.06	170.04	94.04	D	3162.38	3144.37	3145.36	1581.69	30
3	274.10	256.09	257.08	137.55	S	3047.36	3029.35	3030.33	1524.18	29
4	402.16	384.15	385.13	201.58	Q	2960.32	2942.31	2943.30	1480.66	28
5	517.19	499.18	500.16	259.09	D	2832.27	2814.25	2815.24	1416.63	27
6	588.22	570.21	571.20	294.61	A	2717.24	2699.23	2700.21	1359.12	26
7	645.25	627.24	628.22	323.13	G	2646.20	2628.19	2629.17	1323.60	25
8	773.30	755.29	756.29	387.15	Q	2589.18	2571.17	2572.15	1295.09	24
9	902.35	884.34	885.32	451.67	E	2461.12	2443.11	2444.09	1231.06	23
10	1003.40	985.38	986.37	502.20	T	2332.08	2314.07	2315.05	1166.54	22
11	1132.44	1114.43	1115.42	566.72	E	2231.03	2213.02	2214.00	1116.02	21
12	1260.53	1242.52	1243.52	630.77	K	2101.99	2083.98	2084.96	1051.49	20
13	1389.58	1371.57	1372.55	695.29	E	1973.89	1955.88	1956.87	987.45	19
14	1446.60	1428.59	1429.57	723.80	G	1844.84	1826.84	1827.82	922.93	18
15	1575.64	1557.65	1558.63	788.32	E	1787.83	1769.82	1770.80	894.41	17
16	1690.67	1672.66	1673.64	845.83	D	1658.79	1640.78	1641.76	829.89	16
17	1787.72	1769.71	1770.69	894.36	P	1543.76	1525.75	1526.74	772.38	15
18	1915.78	1897.77	1898.75	958.39	Q	1446.71	1428.70	1429.69	723.85	14
19	1986.82	1968.81	1969.79	993.91	A	1318.65	1300.63	1301.62	659.82	13
20	2073.85	2055.84	2056.82	1037.42	S	1247.61	1229.60	1230.59	624.31	12
21	2144.89	2126.88	2127.86	1072.94	A	1160.58	1142.56	1143.55	580.79	11
22	2272.94	2254.93	2255.92	1136.97	Q	1089.54	1071.54	1072.52	545.27	10
23	2387.97	2369.96	2370.94	1194.49	D	961.48	943.47	944.46	481.24	9
24	2517.01	2499.00	2499.99	1259.01	E	846.46	828.44	829.43	423.73	8
25	2618.06	2600.05	2601.03	1309.53	T	717.41	699.40	700.39	359.21	7
26	2715.11	2697.10	2698.09	1358.06	P	616.37	598.35	599.34	308.68	6
27	2828.20	2810.19	2811.17	1414.60	I	519.31	501.30	502.29	260.16	5
28	2929.25	2911.24	2912.22	1465.12	T	406.23	388.22	389.20	203.61	4
29	3016.28	2998.27	2999.25	1508.64	S	305.18	287.17	288.15	153.09	3
30	3087.31	3069.30	3070.29	1544.16	A	218.15	200.14	201.12	109.57	2
31					K	147.11	129.10	130.09	74.06	1

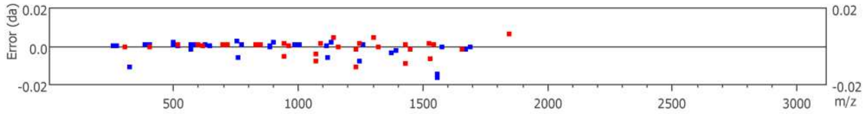

Brandsma C-A, *et al. Thorax* 2020; 75:180–183. doi: 10.1136/thoraxjnl-2019-213200

Peptide Sequence	Gene	Dataset	Sample	Score	ScanID
EASQGSSASSAPQSVK	ZNF830	2D25	COPD8	105.6	2846

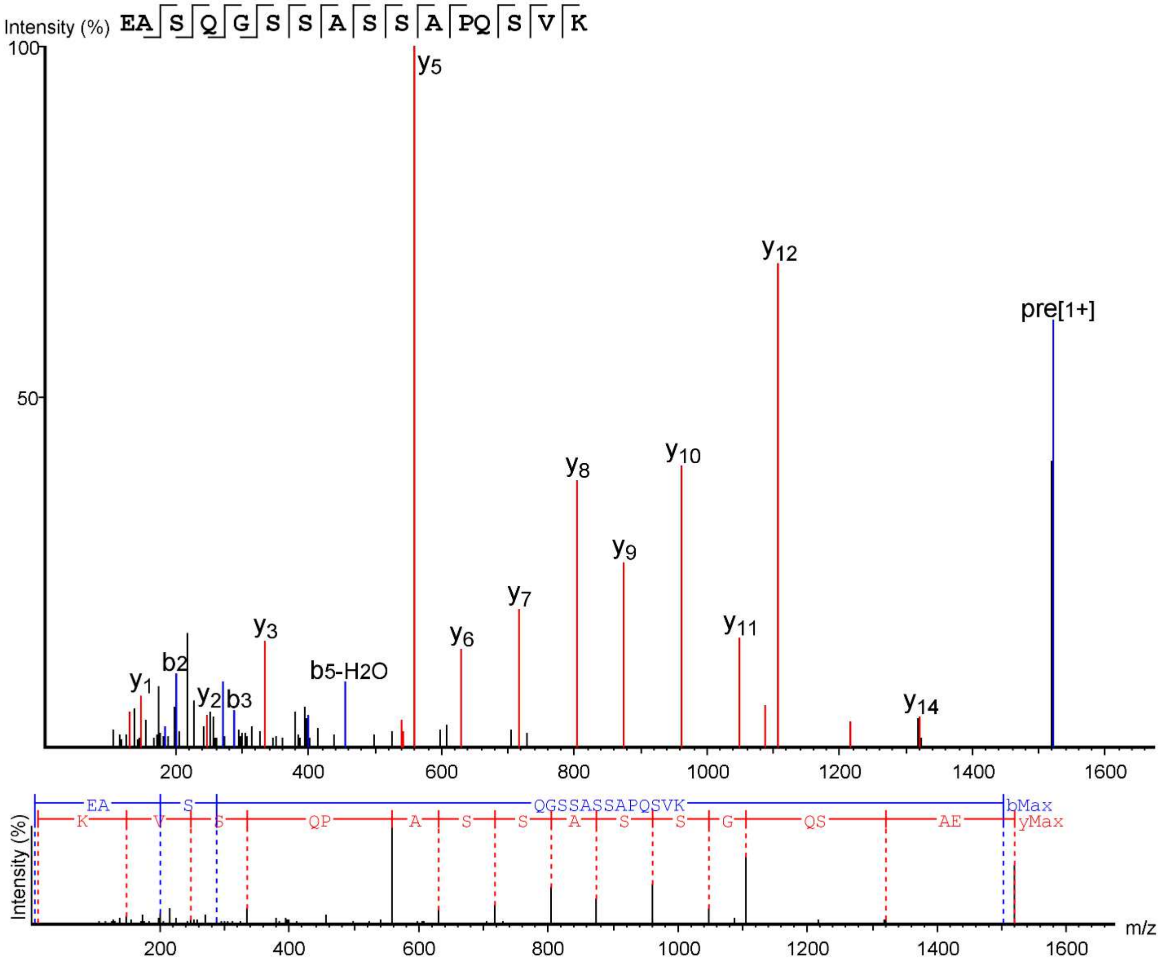

#	b	b-H2O	b-NH3	b (2+)	Seq	y	y-H2O	y-NH3	y (2+)	#
1	130.05	112.04	113.02	65.53	E					16
2	201.09	183.08	184.06	101.04	A	1391.68	1373.67	1374.65	696.34	15
3	288.12	270.11	271.09	144.56	S	1320.62	1302.63	1303.61	660.82	14
4	416.18	398.17	399.15	208.59	Q	1233.61	1215.60	1216.57	617.30	13
5	473.20	455.19	456.17	237.10	G	1105.55	1087.53	1088.52	553.27	12
6	560.23	542.22	543.20	280.62	S	1048.52	1030.52	1031.50	524.76	11
7	647.26	629.25	630.24	324.13	S	961.49	943.48	944.47	481.25	10
8	718.30	700.29	701.27	359.65	A	874.46	856.45	857.44	437.73	9
9	805.33	787.32	788.31	403.17	S	803.43	785.42	786.40	402.21	8
10	892.36	874.35	875.34	446.68	S	716.39	698.38	699.37	358.70	7
11	963.40	945.39	946.37	482.20	A	629.36	611.35	612.33	315.18	6
12	1060.45	1042.44	1043.43	530.73	P	558.32	540.31	541.29	279.66	5
13	1188.51	1170.50	1171.49	594.76	Q	461.27	443.26	444.24	231.14	4
14	1275.55	1257.53	1258.52	638.27	S	333.21	315.20	316.19	167.11	3
15	1374.61	1356.60	1357.59	687.81	V	246.18	228.17	229.15	123.59	2
16					K	147.11	129.10	130.09	74.06	1

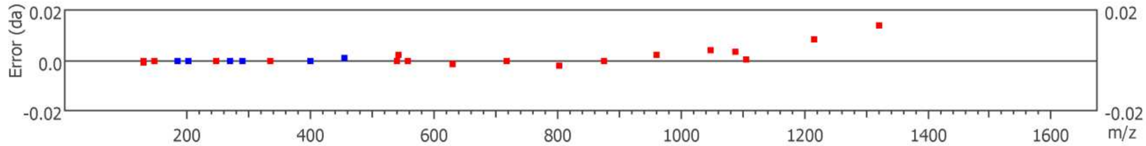

Brandsma C-A, *et al. Thorax* 2020; 75:180–183. doi: 10.1136/thoraxjnl-2019-213200

Peptide Sequence	Gene	Dataset	Sample	Score	ScanID
EQQNDTSSELQNR	ZFYVE16	2D25	COPD1	91.55	3387

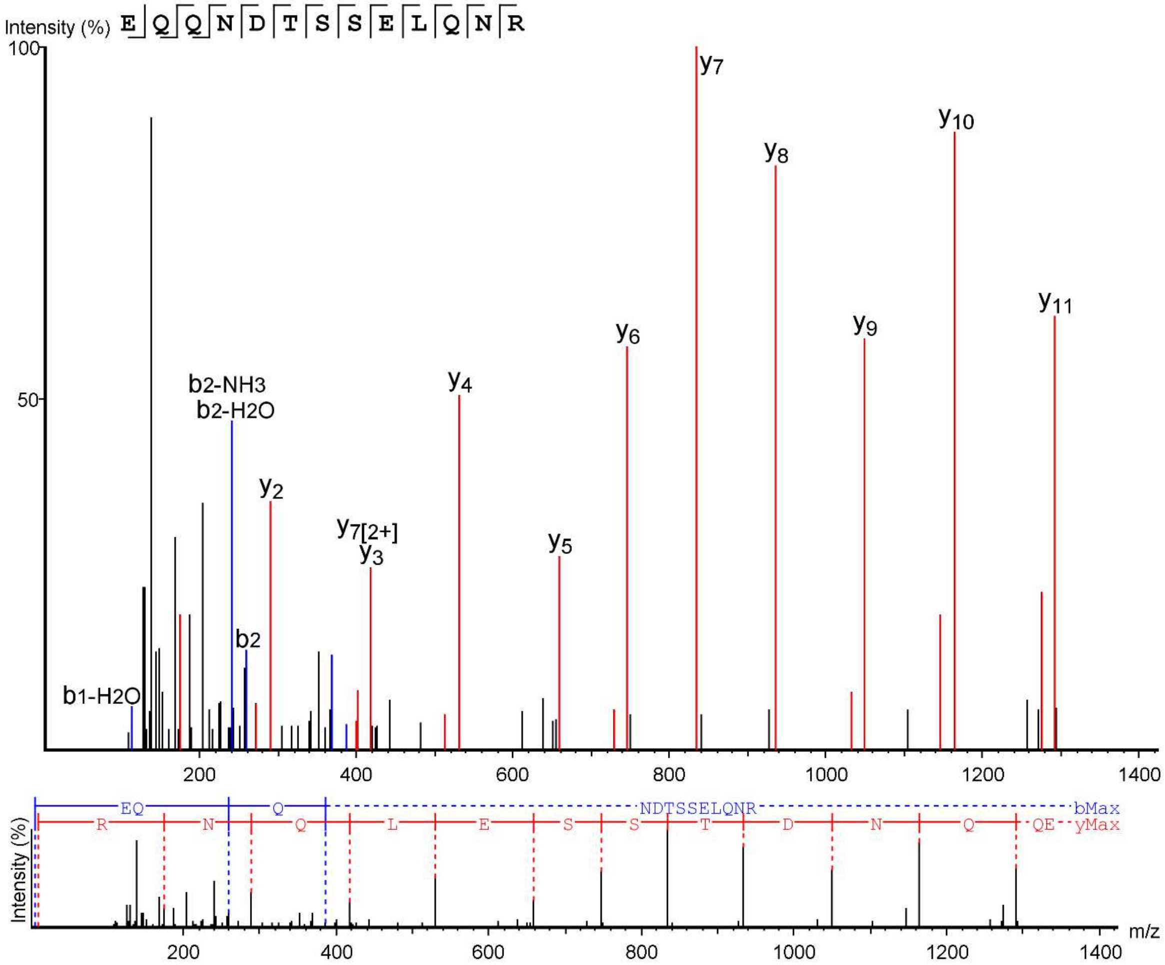

#	b	b-H2O	b-NH3	b (2+)	Seq	y	y-H2O	y-NH3	y (2+)	#
1	130.05	112.05	113.02	65.53	E					13
2	258.11	240.10	241.08	129.55	Q	1419.65	1401.64	1402.62	710.32	12
3	386.17	368.16	369.14	193.58	Q	1291.58	1273.58	1274.56	646.29	11
4	500.21	482.20	483.18	250.61	N	1163.53	1145.52	1146.51	582.26	10
5	615.24	597.23	598.21	308.12	D	1049.48	1031.47	1032.46	525.24	9
6	716.29	698.27	699.26	358.64	T	934.46	916.45	917.43	467.73	8
7	803.32	785.31	786.29	402.16	S	833.41	815.40	816.38	417.22	7
8	890.35	872.34	873.32	445.67	S	746.38	728.37	729.36	373.69	6
9	1019.39	1001.38	1002.36	510.20	E	659.35	641.34	642.32	330.17	5
10	1132.48	1114.47	1115.45	566.74	L	530.30	512.29	513.28	265.65	4
11	1260.53	1242.52	1243.51	630.77	Q	417.22	399.21	400.19	209.11	3
12	1374.58	1356.57	1357.55	687.79	N	289.16	271.15	272.14	145.08	2
13					R	175.12	157.11	158.09	88.06	1

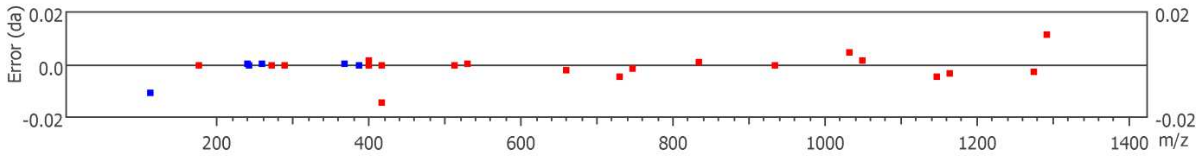

Brandsma C-A, *et al. Thorax* 2020; 75:180–183. doi: 10.1136/thoraxjnl-2019-213200

Peptide Sequence	Gene	Dataset	Sample	Score	ScanID
YGFQFLR	PCYOX1	2D25	COPD10	81.46	15911

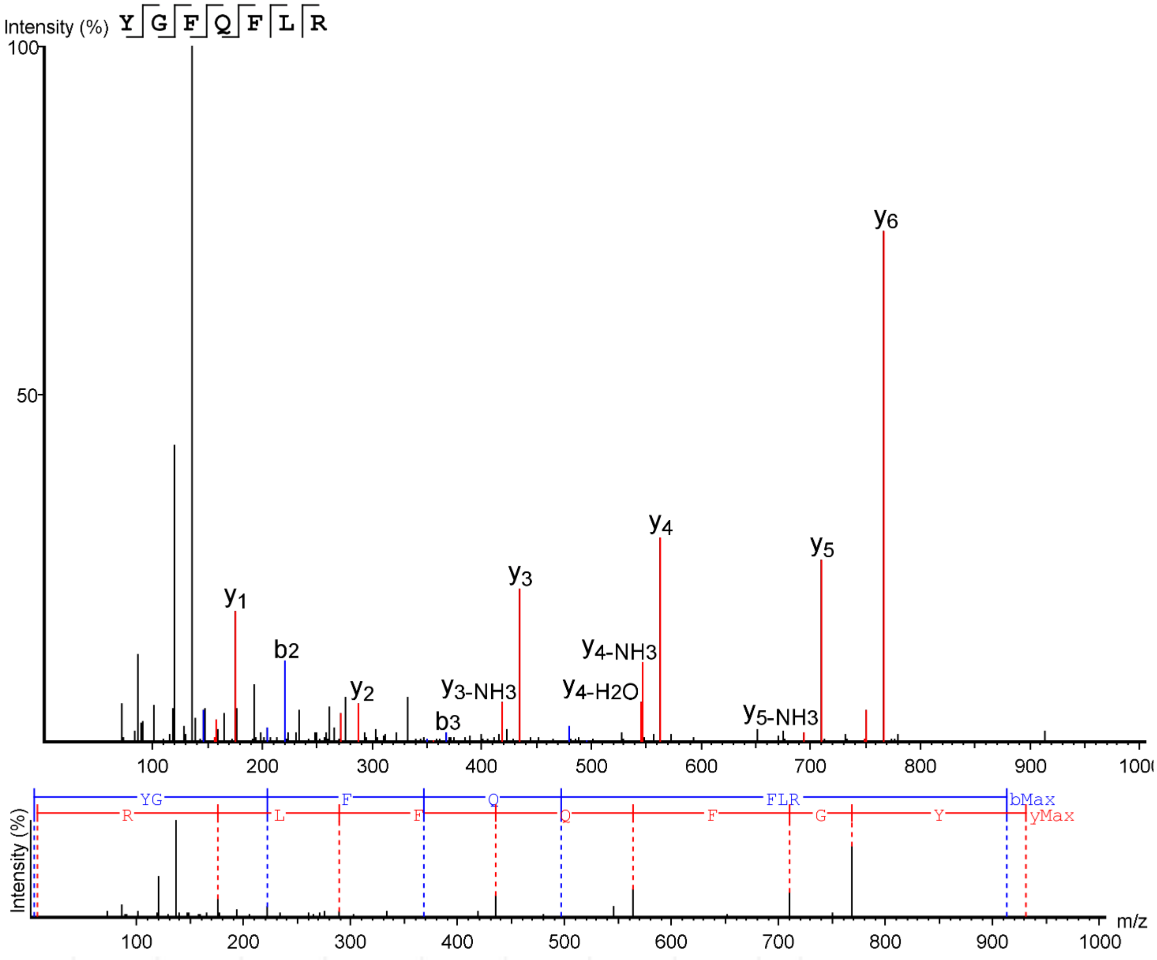

#	b	b-H2O	b-NH3	b (2+)	Seq	y	y-H2O	y-NH3	y (2+)	#
1	164.07	146.06	147.04	82.54	Y					7
2	221.09	203.08	204.07	111.05	G	767.42	749.41	750.39	384.21	6
3	368.16	350.15	351.13	184.58	F	710.40	692.39	693.37	355.70	5
4	496.22	478.21	479.19	248.61	Q	563.33	545.32	546.30	282.16	4
5	643.29	625.28	626.26	322.14	F	435.27	417.26	418.24	218.14	3
6	756.37	738.36	739.35	378.69	L	288.20	270.19	271.18	144.60	2
7					R	175.12	157.11	158.09	88.06	1

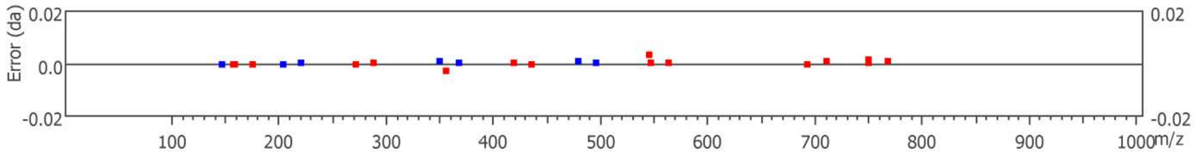

Brandsma C-A, *et al. Thorax* 2020; 75:180–183. doi: 10.1136/thoraxjnl-2019-213200

Peptide Sequence	Gene	Dataset	Sample	Score	ScanID
TGQEALSQTTISWAPFQDTSEYIISCHPVGTDEEPLQFR	FN1	1D50	COPD3	131.44	29106

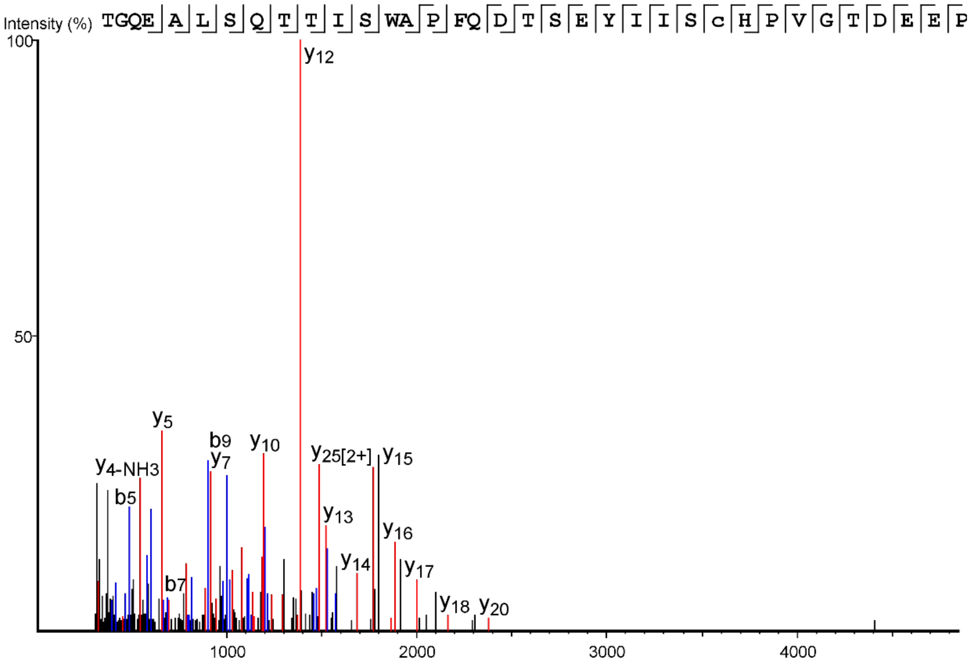

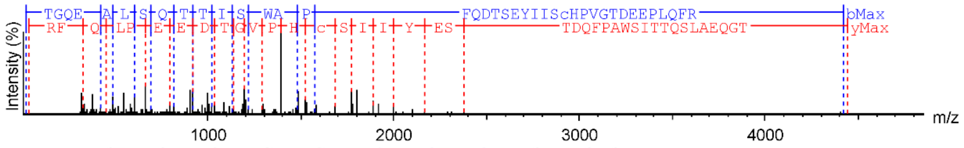

#	b	b-H2O	b-NH3	b (2+)	Seq	y	y-H2O	y-NH3	y (2+)	#
1	102.06	84.04	85.03	51.53	T					39
2	159.08	141.07	142.05	80.04	G	4338.03	4320.02	4321.00	2169.51	38
3	287.14	269.12	270.11	144.07	Q	4281.01	4263.00	4263.98	2141.00	37
4	416.18	398.17	399.15	208.59	E	4152.95	4134.94	4135.92	2076.97	36
5	487.21	469.20	470.21	244.11	A	4023.91	4005.90	4006.88	2012.45	35
6	600.30	582.29	583.27	300.65	L	3952.87	3934.86	3935.84	1976.93	34
7	687.33	669.32	670.30	344.17	S	3839.79	3821.78	3822.76	1920.39	33
8	815.38	797.38	798.36	408.19	Q	3752.75	3734.74	3735.73	1876.88	32
9	916.44	898.43	899.40	458.72	T	3624.70	3606.68	3607.67	1812.85	31
10	1017.49	999.46	1000.46	509.24	T	3523.65	3505.64	3506.62	1762.32	30
11	1130.58	1112.56	1113.56	565.78	I	3422.60	3404.59	3405.57	1711.80	29
12	1217.59	1199.58	1200.59	609.30	S	3309.52	3291.51	3292.49	1655.26	28
13	1403.68	1385.67	1386.65	702.34	W	3222.48	3204.47	3205.46	1611.74	27
14	1474.71	1456.70	1457.70	737.86	A	3036.40	3018.39	3019.38	1518.70	26
15	1571.77	1553.76	1554.74	786.37	P	2965.37	2947.36	2948.34	1483.18	25
16	1718.84	1700.83	1701.81	859.92	F	2868.31	2850.30	2851.29	1434.66	24
17	1846.90	1828.89	1829.87	923.95	Q	2721.25	2703.24	2704.22	1361.12	23
18	1961.92	1943.91	1944.90	981.46	D	2593.19	2575.18	2576.16	1297.10	22
19	2062.97	2044.96	2045.95	1031.99	T	2478.16	2460.15	2461.13	1239.59	21
20	2150.00	2131.99	2132.98	1075.50	S	2377.10	2359.10	2360.09	1189.05	20
21	2279.05	2261.04	2262.02	1140.02	E	2290.08	2272.07	2273.05	1145.54	19
22	2442.11	2424.10	2425.08	1221.56	Y	2161.05	2143.03	2144.01	1081.04	18
23	2555.19	2537.18	2538.17	1278.10	I	1997.97	1979.96	1980.95	999.48	17
24	2668.28	2650.27	2651.25	1334.64	I	1884.90	1866.86	1867.86	942.96	16
25	2755.31	2737.30	2738.28	1378.16	S	1771.81	1753.80	1754.78	886.40	15
26	2915.34	2897.33	2898.31	1458.17	C(+57.02)	1684.79	1666.76	1667.75	842.89	14
27	3052.40	3034.39	3035.37	1526.70	H	1524.75	1506.73	1507.72	762.87	13
28	3149.45	3131.44	3132.43	1575.23	P	1387.67	1369.66	1370.67	694.34	12
29	3248.52	3230.51	3231.49	1624.76	V	1290.64	1272.62	1273.61	645.82	11
30	3305.54	3287.53	3288.52	1653.27	G	1191.57	1173.55	1174.54	596.28	10
31	3406.59	3388.58	3389.56	1703.80	T	1134.54	1116.53	1117.52	567.77	9
32	3521.62	3503.61	3504.59	1761.31	D	1033.50	1015.48	1016.47	517.25	8
33	3650.66	3632.65	3633.63	1825.83	E	918.47	900.45	901.43	459.73	7
34	3779.70	3761.69	3762.68	1890.35	E	789.42	771.41	772.40	395.21	6
35	3876.75	3858.74	3859.73	1938.88	P	660.38	642.37	643.36	330.69	5
36	3989.84	3971.83	3972.81	1995.42	L	563.33	545.32	546.28	282.16	4
37	4117.90	4099.89	4100.87	2059.45	Q	450.25	432.24	433.22	225.62	3
38	4264.97	4246.96	4247.94	2132.98	F	322.19	304.18	305.16	161.59	2
39					R	175.12	157.11	158.09	88.06	1

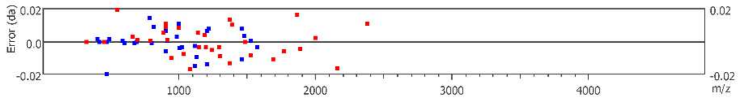

Peptide Sequence	Gene	Dataset	Sample	Score	ScanID
AEHMETNAVGPSQSSDTR	MPRIP	2D25	COPD4	129.43	2347

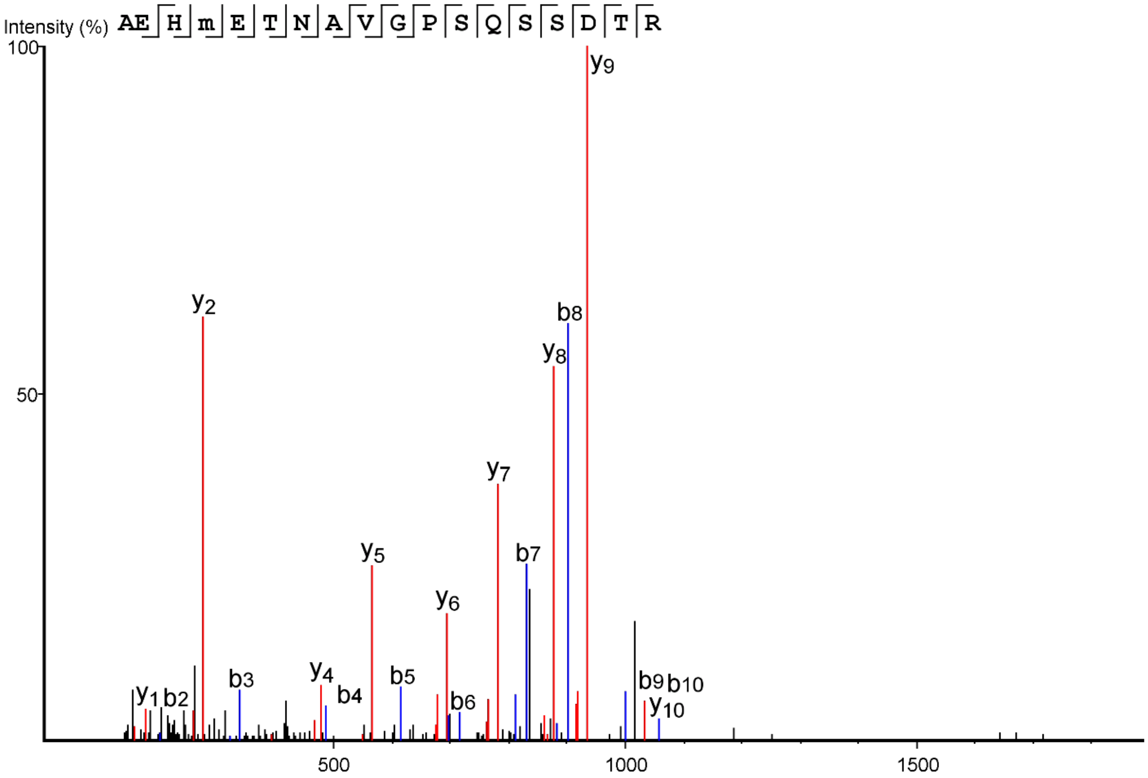

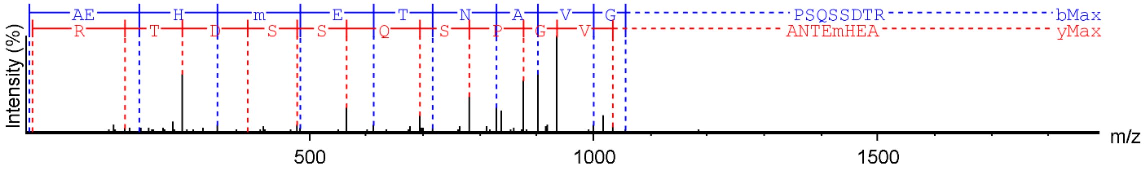

#	b	b-H2O	b-NH3	b (2+)	Seq	y	y-H2O	y-NH3	y (2+)	#
1	72.04	54.03	55.02	36.52	A					18
2	201.09	183.08	184.06	101.04	E	1861.80	1843.79	1844.77	931.40	17
3	338.15	320.13	321.12	169.57	H	1732.76	1714.74	1715.73	866.86	16
4	485.18	467.17	468.15	243.09	M(+15.99)	1595.70	1577.69	1578.67	798.35	15
5	614.22	596.21	597.20	307.61	E	1448.66	1430.65	1431.63	724.83	14
6	715.27	697.26	698.25	358.14	T	1319.62	1301.61	1302.59	660.31	13
7	829.31	811.30	812.29	415.16	N	1218.57	1200.56	1201.54	609.79	12
8	900.35	882.34	883.33	450.68	A	1104.53	1086.52	1087.50	552.76	11
9	999.42	981.41	982.39	500.21	V	1033.49	1015.48	1016.46	517.25	10
10	1056.44	1038.43	1039.42	528.72	G	934.42	916.41	917.40	467.71	9
11	1153.49	1135.48	1136.47	577.25	P	877.40	859.39	860.39	439.20	8
12	1240.53	1222.52	1223.50	620.76	S	780.35	762.34	763.33	390.67	7
13	1368.59	1350.57	1351.56	684.79	Q	693.32	675.30	676.29	347.16	6
14	1455.62	1437.61	1438.59	728.31	S	565.26	547.25	548.23	283.13	5
15	1542.65	1524.64	1525.62	771.82	S	478.23	460.22	461.20	239.61	4
16	1657.68	1639.67	1640.65	829.34	D	391.19	373.18	374.17	196.10	3
17	1758.72	1740.71	1741.70	879.86	T	276.17	258.16	259.14	138.58	2
18					R	175.12	157.11	158.09	88.06	1

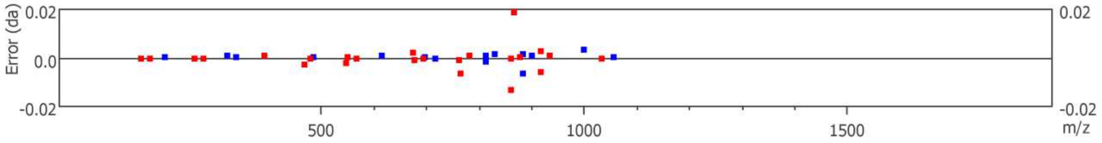

Peptide Sequence	Gene	Dataset	Sample	Score	ScanID
YEDEINR	KRT7	2D25	COPD5	49.8	3828

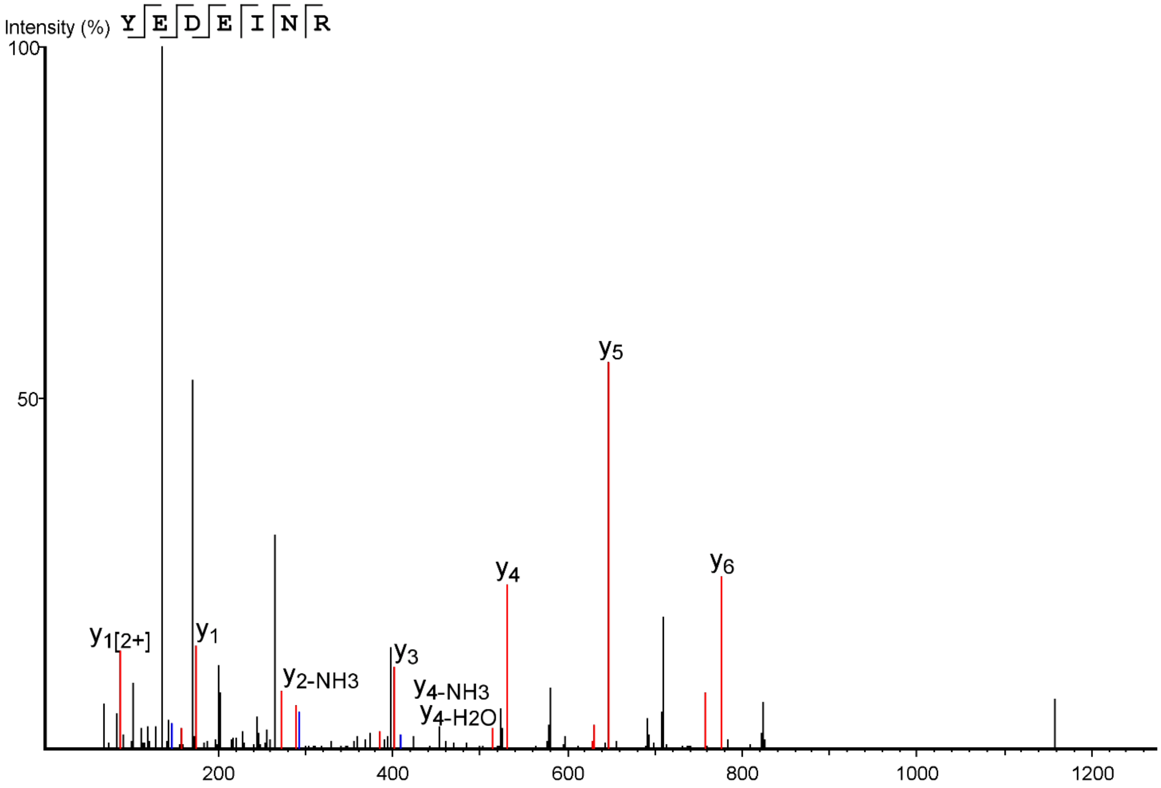

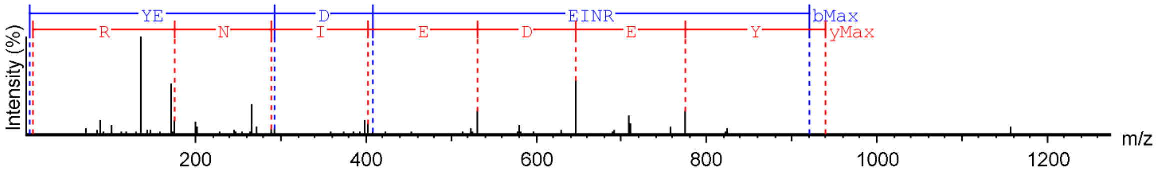

#	b	b-H2O	b-NH3	b (2+)	Seq	y	y-H2O	y-NH3	y (2+)	#
1	164.07	146.06	147.04	82.54	Y					7
2	293.11	275.10	276.09	147.04	E	775.36	757.35	758.33	388.18	6
3	408.14	390.13	391.11	204.57	D	646.31	628.31	629.29	323.66	5
4	537.18	519.17	520.16	269.09	E	531.29	513.28	514.26	266.14	4
5	650.27	632.26	633.24	325.63	I	402.25	384.24	385.22	201.62	3
6	764.31	746.30	747.28	382.66	N	289.16	271.15	272.13	145.08	2
7					R	175.12	157.11	158.09	88.04	1

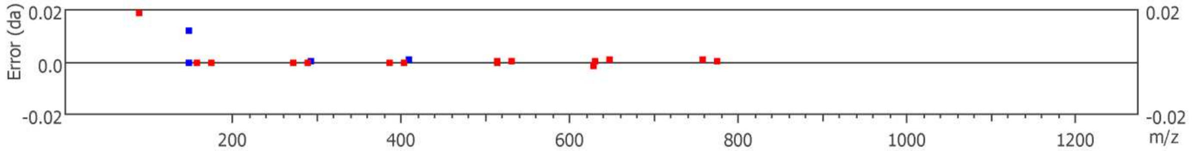

Brandsma C-A, *et al. Thorax* 2020; 75:180–183. doi: 10.1136/thoraxjnl-2019-213200

Peptide Sequence	Gene	Dataset	Sample	Score	ScanID
SDSELNNEVAAR	CYBRD1	2D25	COPD6	103.81	6014

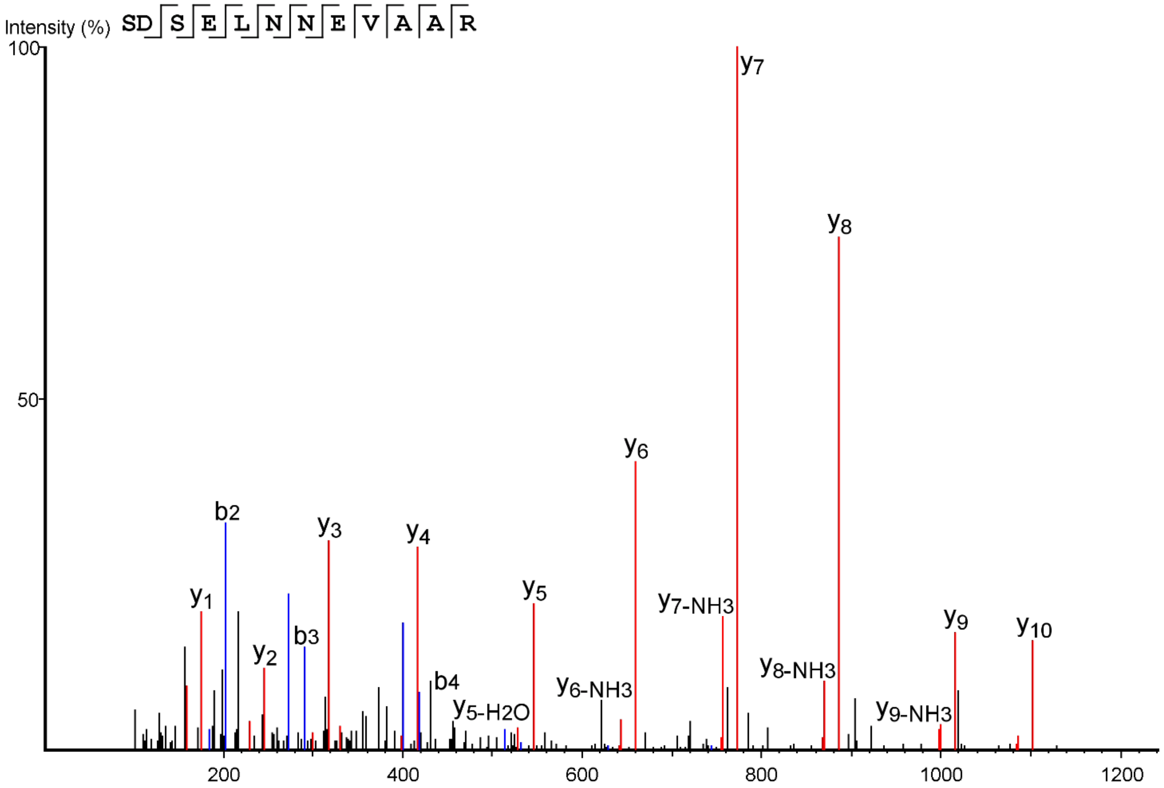

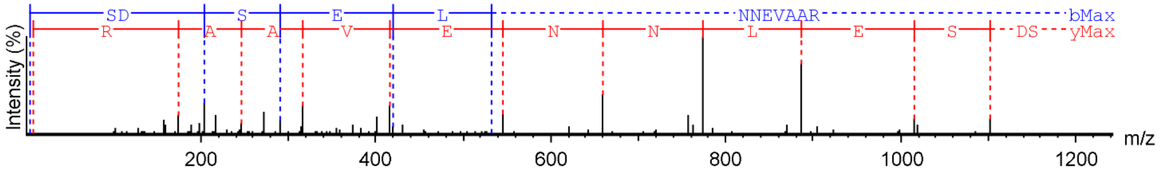

#	b	b-H2O	b-NH3	b (2+)	Seq	y	y-H2O	y-NH3	y (2+)	#
1	88.04	70.03	71.01	44.52	S					12
2	203.07	185.06	186.04	102.03	D	1217.58	1199.57	1200.55	609.29	11
3	290.10	272.09	273.07	145.55	S	1102.55	1084.54	1085.52	551.77	10
4	419.14	401.13	402.11	210.07	E	1015.52	997.51	998.49	508.26	9
5	532.22	514.21	515.20	266.61	L	886.47	868.46	869.45	443.74	8
6	646.27	628.26	629.25	323.63	N	773.39	755.38	756.36	387.20	7
7	760.31	742.30	743.29	380.66	N	659.35	641.33	642.32	330.16	6
8	889.35	871.34	872.33	445.18	E	545.30	527.29	528.28	273.15	5
9	988.42	970.41	971.40	494.71	V	416.26	398.25	399.24	208.63	4
10	1059.46	1041.45	1042.43	530.23	A	317.19	299.18	300.17	159.11	3
11	1130.50	1112.49	1113.47	565.75	A	246.16	228.15	229.13	123.58	2
12					R	175.12	157.11	158.09	88.06	1

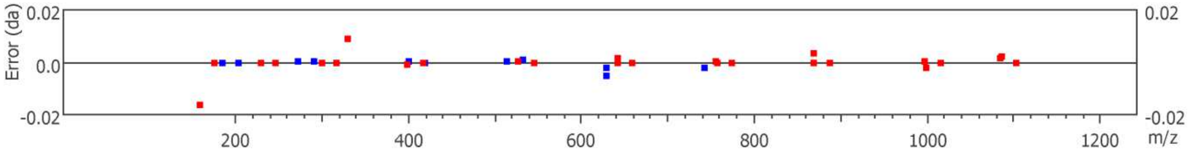

Peptide Sequence	Gene	Dataset	Sample	Score	ScanID
EPVSGAVEGK	CAVIN2	2D25	COPD10	62.17	3769

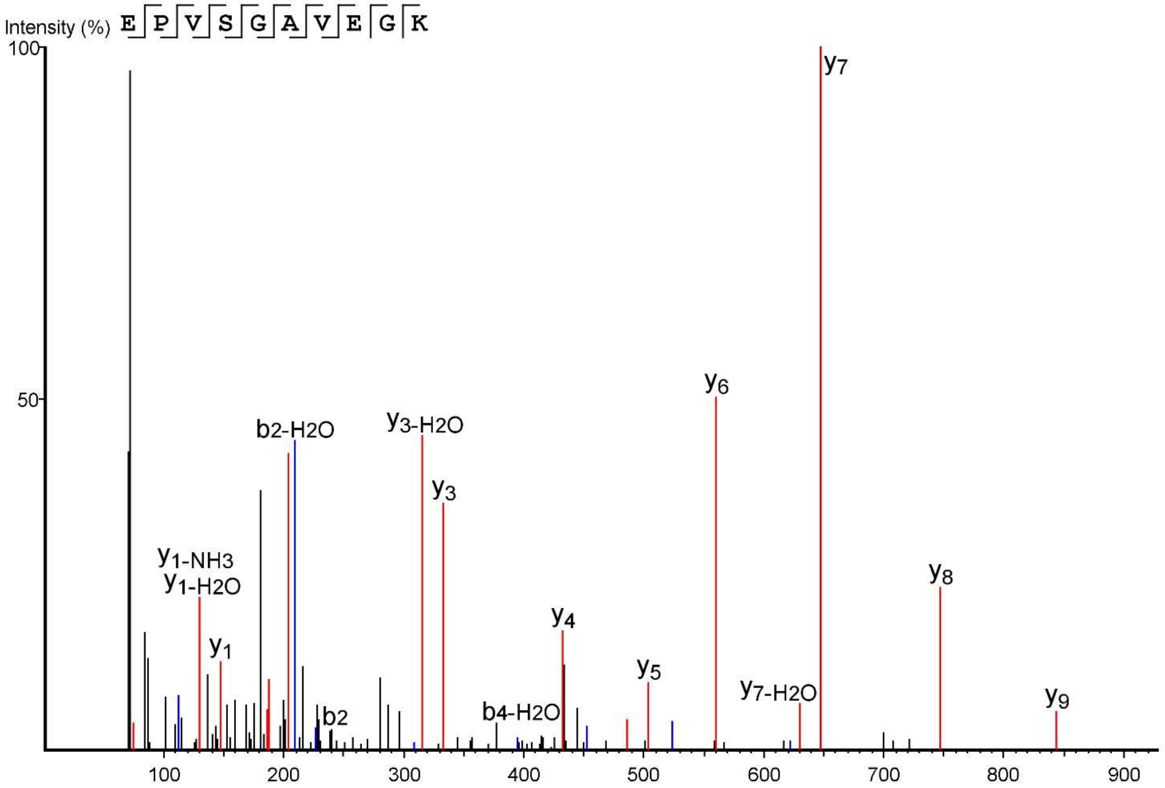

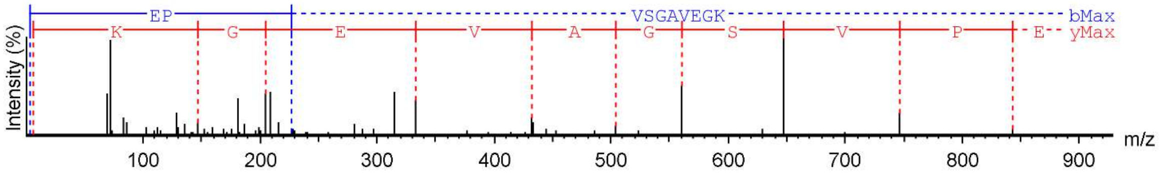

#	b	b-H2O	b-NH3	b (2+)	Seq	y	y-H2O	y-NH3	y (2+)	#
1	130.05	112.05	113.02	65.53	E					10
2	227.10	209.09	210.08	114.05	P	843.46	825.45	826.43	422.23	9
3	326.17	308.16	309.14	163.59	V	746.40	728.39	729.38	373.70	8
4	413.20	395.19	396.18	207.10	S	647.34	629.32	630.31	324.17	7
5	470.23	452.21	453.20	235.61	G	560.30	542.29	543.28	280.65	6
6	541.26	523.25	524.24	271.13	A	503.28	485.27	486.26	252.14	5
7	640.33	622.32	623.30	320.67	V	432.24	414.23	415.22	216.62	4
8	769.37	751.36	752.35	385.19	E	333.18	315.17	316.15	167.09	3
9	826.39	808.38	809.37	413.70	G	204.13	186.12	187.11	102.57	2
10					K	147.11	129.10	130.09	74.06	1

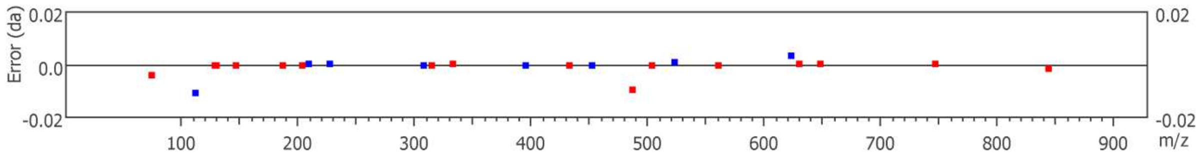

Brandsma C-A, *et al. Thorax* 2020; 75:180–183. doi: 10.1136/thoraxjnl-2019-213200

Peptide Sequence	Gene	Dataset	Sample	Score	ScanID
NKYEDEINR	KRT7	2D25	COPD4	70.58	4085

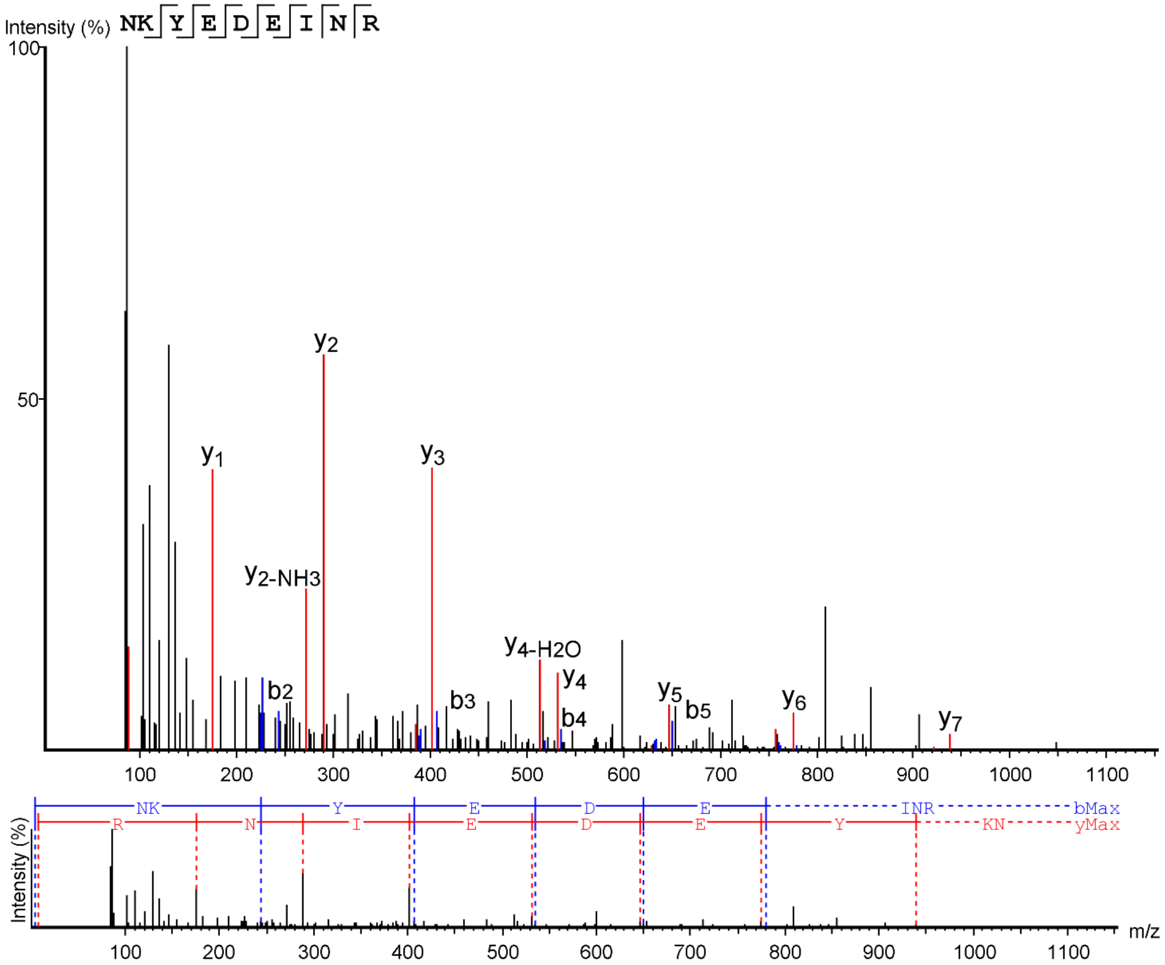

#	b	b-H2O	b-NH3	b (2+)	Seq	y	y-H2O	y-NH3	y (2+)	#
1	115.05	97.04	98.02	58.03	N					9
2	243.14	225.13	226.12	122.07	K	1066.52	1048.51	1049.49	533.76	8
3	406.21	388.20	389.18	203.60	Y	938.42	920.41	921.39	469.71	7
4	535.25	517.24	518.23	268.13	E	775.36	757.34	758.33	388.20	6
5	650.28	632.27	633.25	325.64	D	646.32	628.30	629.29	323.66	5
6	779.32	761.31	762.29	390.16	E	531.29	513.28	514.26	266.14	4
7	892.41	874.39	875.38	446.70	I	402.25	384.24	385.22	201.62	3
8	1006.45	988.44	989.42	503.72	N	289.16	271.15	272.13	145.08	2
9					R	175.12	157.11	158.09	88.04	1

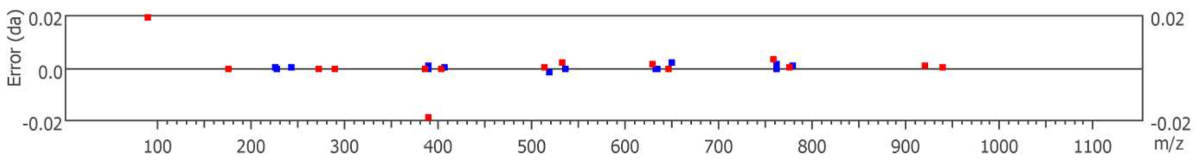

Brandsma C-A, *et al. Thorax* 2020; 75:180–183. doi: 10.1136/thoraxjnl-2019-213200

Peptide Sequence	Gene	Dataset	Sample	Score	ScanID
DPAEGTPLEAAGTR	SORBS1	2D25	COPD8	118.25	10066

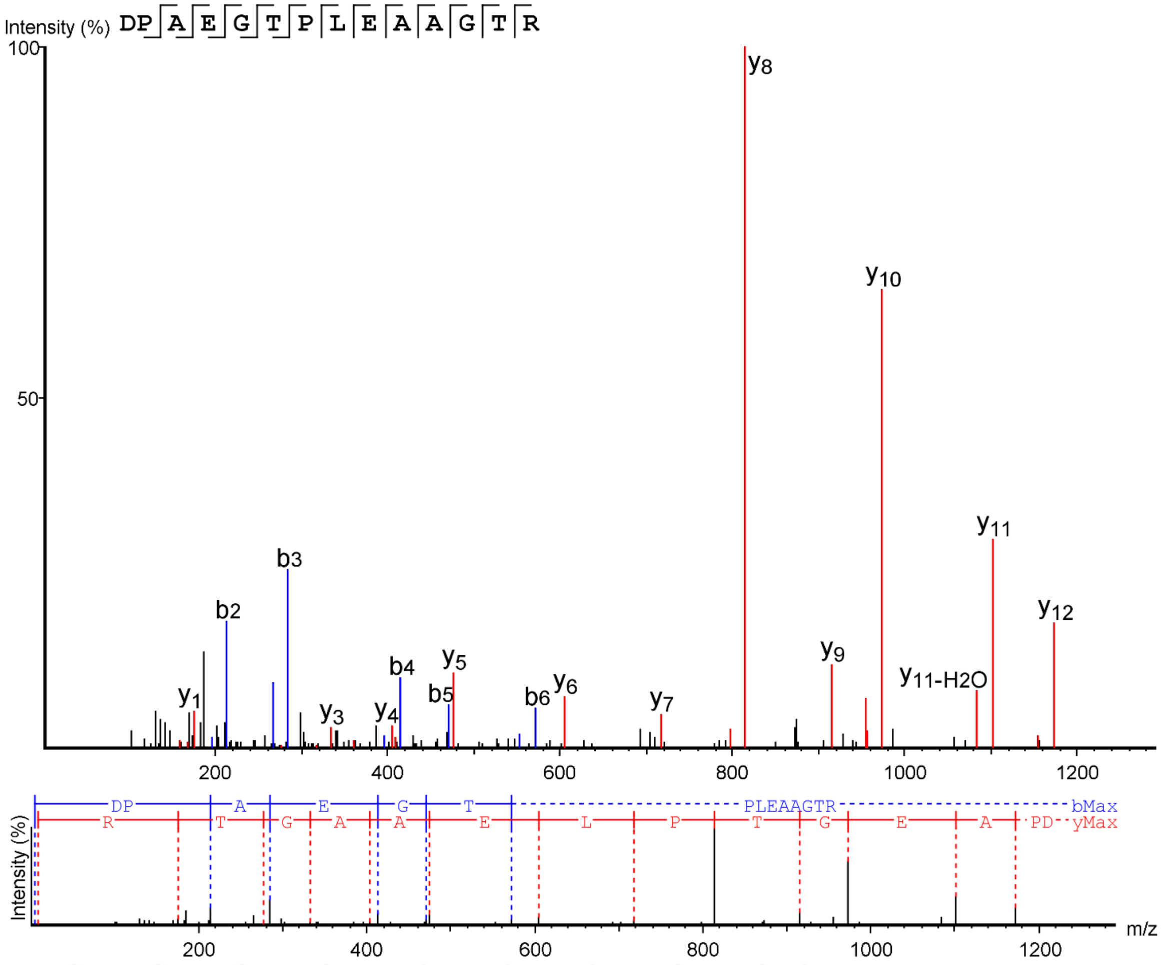

#	b	b-H2O	b-NH3	b (2+)	Seq	y	y-H2O	y-NH3	y (2+)	#
1	116.03	98.02	99.01	58.52	D					14
2	213.09	195.08	196.06	107.04	P	1269.64	1251.63	1252.62	635.32	13
3	284.12	266.11	267.10	142.56	A	1172.59	1154.58	1155.56	586.80	12
4	413.17	395.16	396.14	207.08	E	1101.55	1083.54	1084.53	551.28	11
5	470.19	452.18	453.16	235.59	G	972.51	954.50	955.49	486.76	10
6	571.23	553.23	554.21	286.12	T	915.49	897.48	898.46	458.24	9
7	668.29	650.28	651.26	334.64	P	814.44	796.43	797.42	407.72	8
8	781.37	763.36	764.35	391.19	L	717.39	699.38	700.36	359.19	7
9	910.42	892.41	893.39	455.71	E	604.31	586.29	587.28	302.65	6
10	981.45	963.44	964.43	491.23	A	475.26	457.25	458.24	238.13	5
11	1052.49	1034.48	1035.46	526.74	A	404.23	386.21	387.20	202.61	4
12	1109.51	1091.50	1092.48	555.26	G	333.19	315.18	316.16	167.08	3
13	1210.56	1192.55	1193.53	605.78	T	276.17	258.16	259.14	138.58	2
14					R	175.12	157.11	158.09	88.06	1

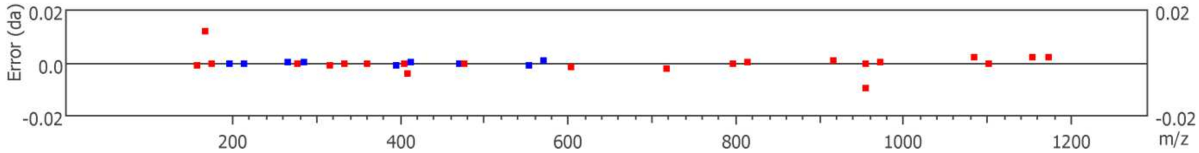

Brandsma C-A, *et al. Thorax* 2020; 75:180–183. doi: 10.1136/thoraxjnl-2019-213200

Peptide Sequence	Gene	Dataset	Sample	Score	ScanID
EGEDPQASAQDETPITSAK	AKAP12	2D25	COPD5	129.34	9167

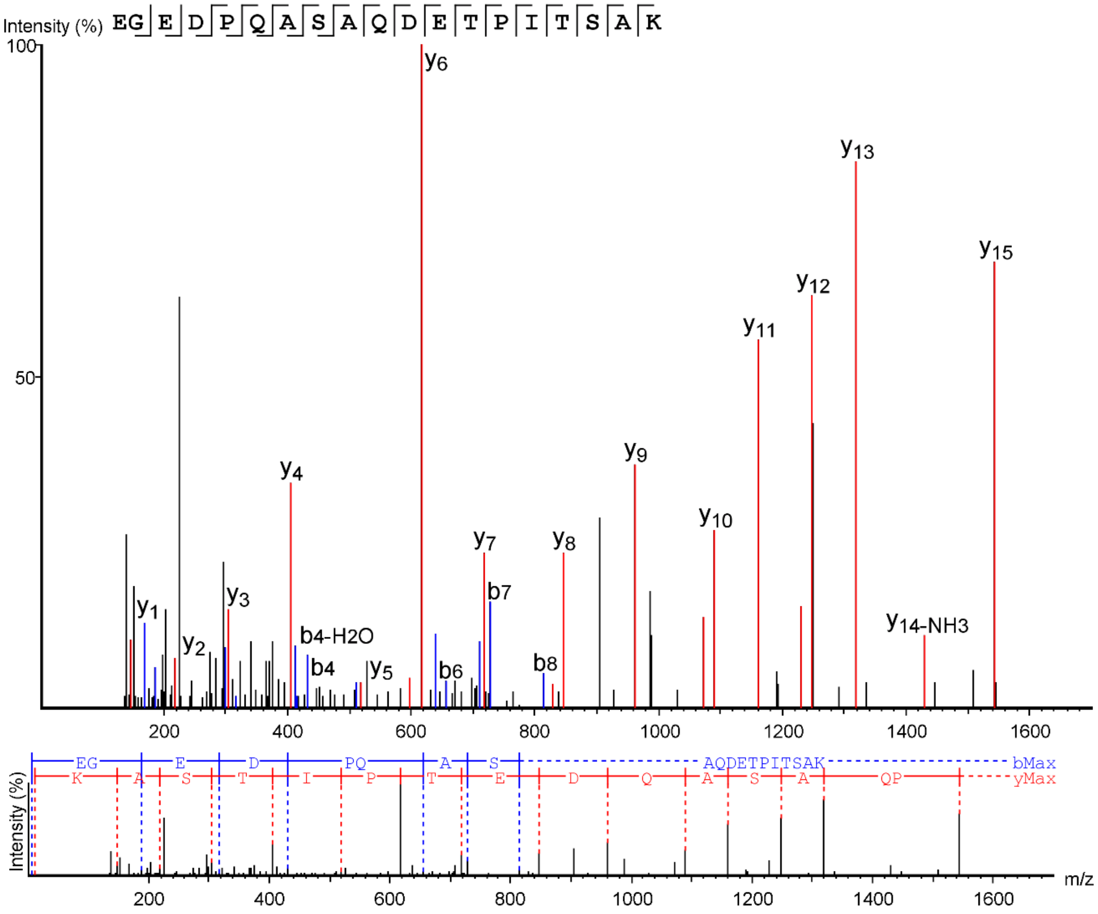

#	b	b-H2O	b-NH3	b (2+)	Seq	y	y-H2O	y-NH3	y (2+)	#
1	130.05	112.04	113.02	65.53	E					19
2	187.07	169.06	170.04	94.04	G	1844.85	1826.84	1827.82	922.93	18
3	316.11	298.10	299.09	158.56	E	1787.83	1769.82	1770.80	894.41	17
4	431.14	413.13	414.10	216.07	D	1658.79	1640.78	1641.76	829.89	16
5	528.19	510.18	511.17	264.60	P	1543.76	1525.75	1526.73	772.38	15
6	656.25	638.24	639.23	328.63	Q	1446.71	1428.70	1429.67	723.85	14
7	727.29	709.28	710.26	364.14	A	1318.65	1300.64	1301.62	659.82	13
8	814.32	796.31	797.29	407.66	S	1247.61	1229.60	1230.58	624.31	12
9	885.36	867.35	868.33	443.18	A	1160.58	1142.57	1143.55	580.79	11
10	1013.42	995.41	996.39	507.21	Q	1089.54	1071.54	1072.51	545.27	10
11	1128.44	1110.43	1111.42	564.72	D	961.49	943.47	944.46	481.24	9
12	1257.49	1239.48	1240.46	629.24	E	846.46	828.45	829.43	423.73	8
13	1358.53	1340.52	1341.51	679.77	T	717.41	699.40	700.39	359.21	7
14	1455.59	1437.58	1438.56	728.29	P	616.37	598.35	599.34	308.68	6
15	1568.67	1550.66	1551.64	784.84	I	519.31	501.30	502.29	260.16	5
16	1669.72	1651.71	1652.69	835.36	T	406.23	388.22	389.20	203.61	4
17	1756.75	1738.74	1739.72	878.88	S	305.18	287.17	288.15	153.09	3
18	1827.79	1809.78	1810.76	914.39	A	218.15	200.14	201.12	109.57	2
19					K	147.11	129.10	130.09	74.06	1

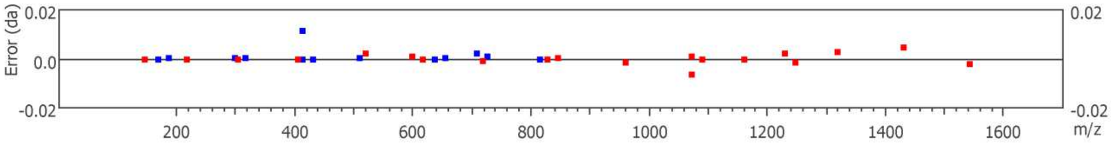

Brandsma C-A, *et al. Thorax* 2020; 75:180–183. doi: 10.1136/thoraxjnl-2019-213200

Peptide Sequence	Gene	Dataset	Sample	Score	ScanID
TLEGLQVEEEPVYK	HCLS1	2D25	COPD7	106.37	13173

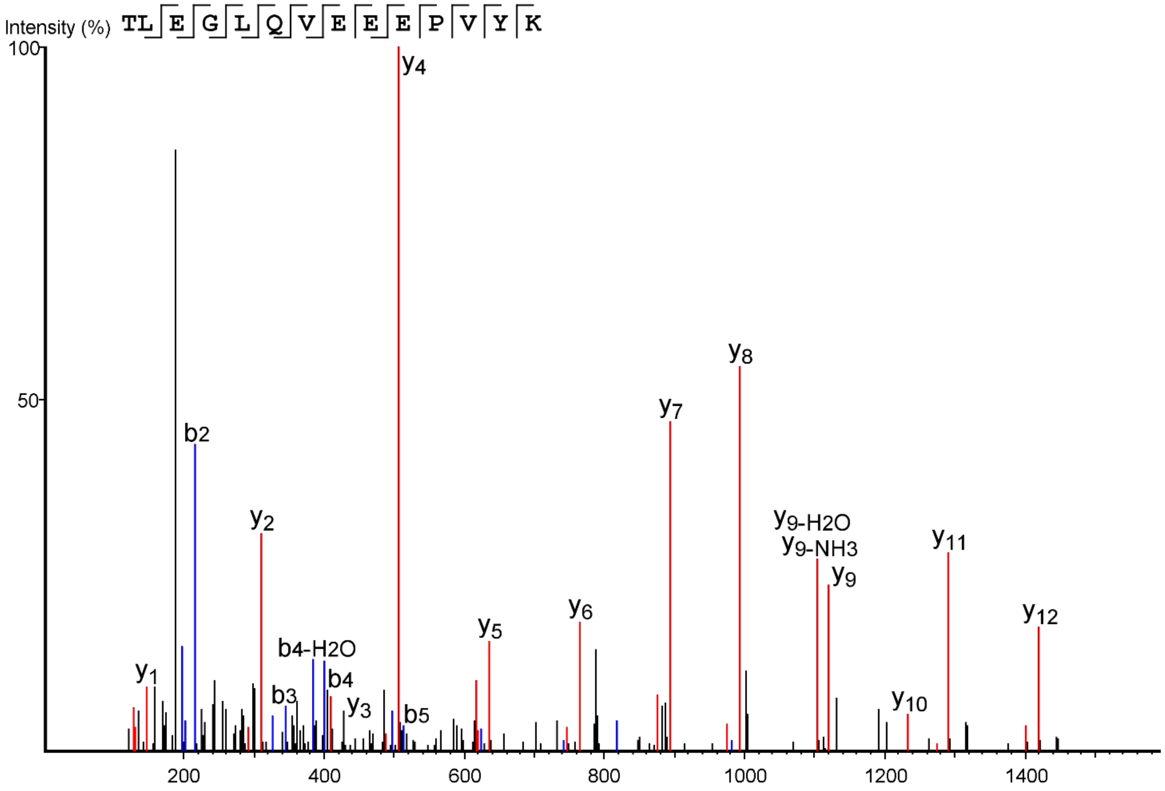

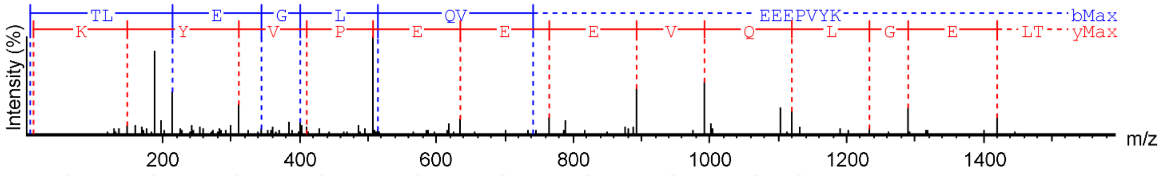

#	b	b-H2O	b-NH3	b (2+)	Seq	y	y-H2O	y-NH3	y (2+)	#
1	102.06	84.04	85.03	51.53	T					14
2	215.14	197.13	198.11	108.07	L	1532.78	1514.77	1515.76	766.89	13
3	344.18	326.17	327.16	172.59	E	1419.70	1401.68	1402.67	710.35	12
4	401.20	383.19	384.18	201.09	G	1290.66	1272.65	1273.65	645.83	11
5	514.29	496.28	497.26	257.64	L	1233.63	1215.63	1216.61	617.33	10
6	642.35	624.33	625.32	321.67	Q	1120.55	1102.54	1103.53	560.78	9
7	741.42	723.40	724.39	371.21	V	992.49	974.47	975.48	496.75	8
8	870.46	852.45	853.43	435.73	E	893.43	875.41	876.40	447.21	7
9	999.50	981.49	982.47	500.25	E	764.38	746.37	747.36	382.69	6
10	1128.54	1110.53	1111.52	564.77	E	635.34	617.33	618.33	318.17	5
11	1225.60	1207.58	1208.57	613.30	P	506.30	488.29	489.27	253.65	4
12	1324.66	1306.65	1307.64	662.83	V	409.24	391.23	392.22	205.12	3
13	1487.73	1469.72	1470.70	744.36	Y	310.18	292.17	293.15	155.59	2
14					K	147.11	129.10	130.09	74.06	1

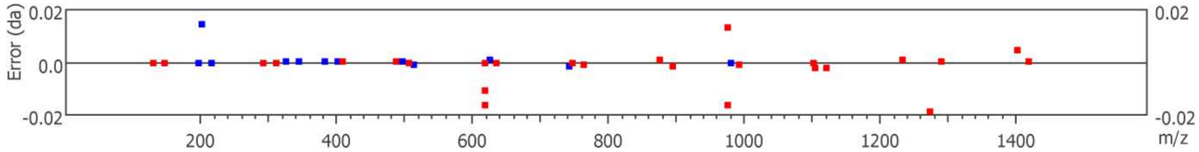

Brandsma C-A, *et al. Thorax* 2020; 75:180–183. doi: 10.1136/thoraxjnl-2019-213200

Peptide Sequence	Gene	Dataset	Sample	Score	ScanID
DSRPSQAAGDNQGDEVK	THRAP3	1D25	COPD9	132.86	2397

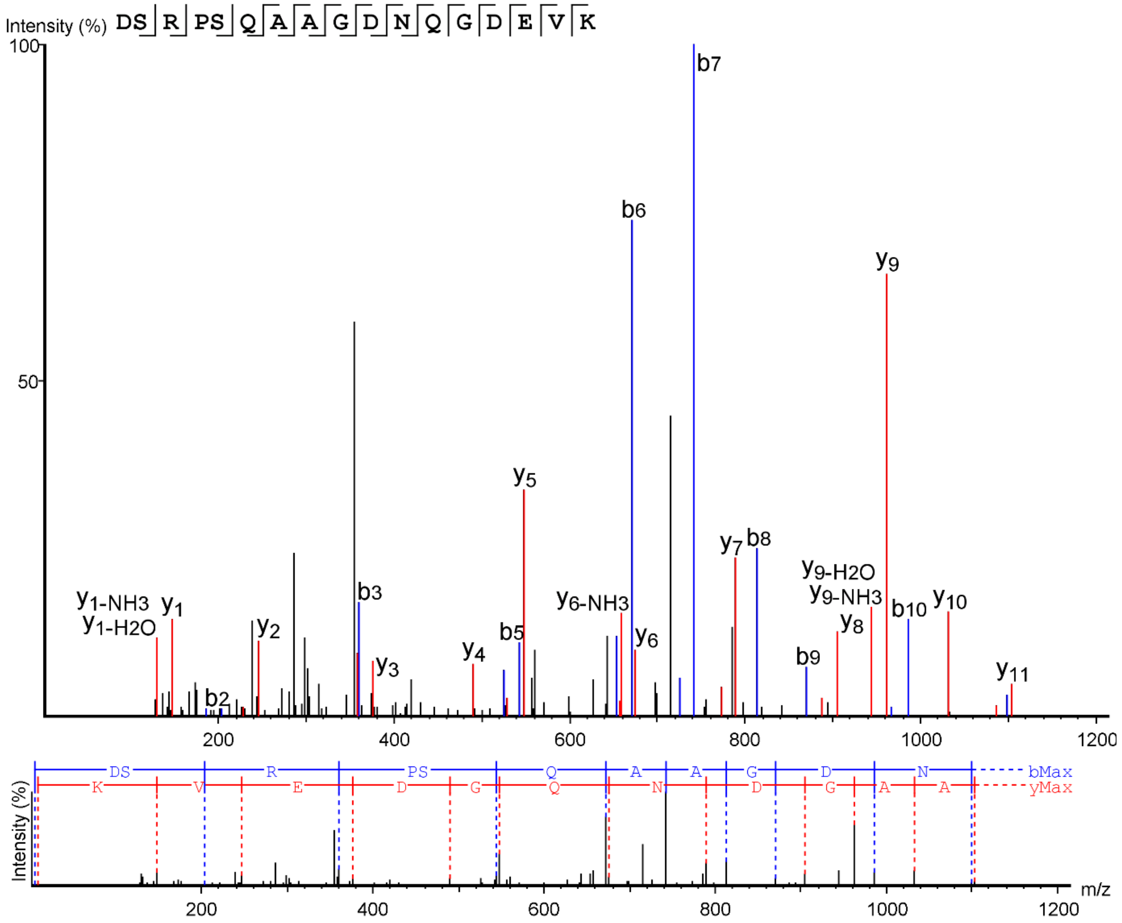

#	b	b-H2O	b-NH3	b (2+)	Seq	y	y-H2O	y-NH3	y (2+)	#
1	116.03	98.02	99.01	58.52	D					17
2	203.07	185.06	186.04	102.03	S	1658.77	1640.76	1641.75	829.89	16
3	359.17	341.16	342.14	180.08	R	1571.74	1553.73	1554.71	786.37	15
4	456.22	438.21	439.19	228.61	P	1415.64	1397.63	1398.61	708.32	14
5	543.25	525.24	526.23	272.13	S	1318.59	1300.58	1301.56	659.79	13
6	671.31	653.30	654.28	336.16	Q	1231.55	1213.54	1214.53	616.28	12
7	742.35	724.34	725.32	371.67	A	1103.50	1085.49	1086.47	552.25	11
8	813.39	795.37	796.36	407.19	A	1032.46	1014.45	1015.43	516.73	10
9	870.41	852.40	853.38	435.70	G	961.42	943.41	944.40	481.21	9
10	985.43	967.42	968.41	493.22	D	904.40	886.39	887.37	452.70	8
11	1099.48	1081.47	1082.45	550.24	N	789.37	771.36	772.35	395.19	7
12	1227.54	1209.52	1210.51	614.27	Q	675.33	657.32	658.30	338.17	6
13	1284.56	1266.55	1267.53	642.78	G	547.27	529.26	530.25	274.14	5
14	1399.58	1381.57	1382.56	700.29	D	490.25	472.24	473.22	245.63	4
15	1528.63	1510.62	1511.60	764.81	E	375.22	357.21	358.20	188.11	3
16	1627.69	1609.68	1610.67	814.35	V	246.18	228.17	229.15	123.59	2
17					K	147.11	129.10	130.09	74.06	1

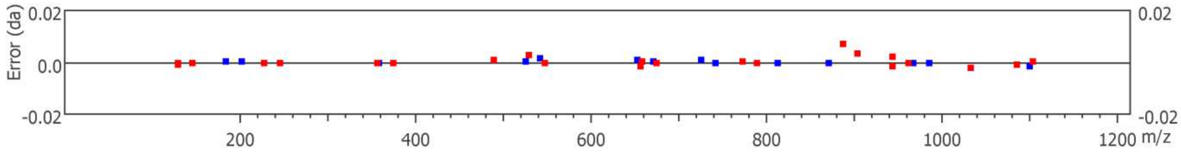

Brandsma C-A, *et al. Thorax* 2020; 75:180–183. doi: 10.1136/thoraxjnl-2019-213200

Peptide Sequence	Gene	Dataset	Sample	Score	ScanID
ELTVSNNDINEAGVHVLCQGLK	RNH1	1D25	COPD5	115.8	17573

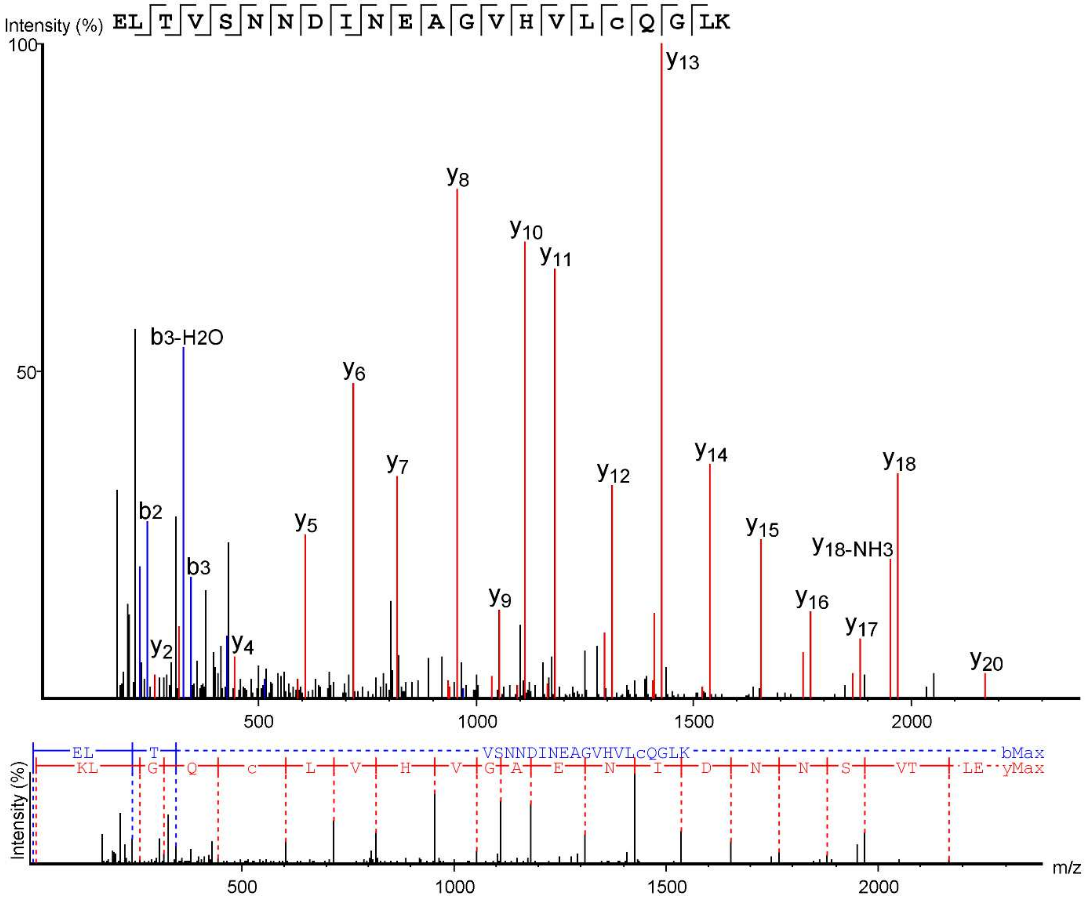

#	b	b-H2O	b-NH3	b (2+)	Seq	y	y-H2O	y-NH3	y (2+)	#
1	130.05	112.04	113.02	65.53	E					22
2	243.13	225.12	226.11	122.07	L	2281.16	2263.15	2264.13	1141.08	21
3	344.18	326.17	327.16	172.59	T	2168.08	2150.07	2151.05	1084.54	20
4	443.25	425.24	426.22	222.13	V	2067.03	2049.02	2050.00	1034.03	19
5	530.28	512.27	513.26	265.64	S	1967.97	1949.95	1950.93	984.48	18
6	644.33	626.31	627.30	322.66	N	1880.93	1862.92	1863.91	940.96	17
7	758.37	740.36	741.34	379.68	N	1766.90	1748.87	1749.87	883.94	16
8	873.40	855.38	856.37	437.20	D	1652.85	1634.83	1635.82	826.92	15
9	986.48	968.47	969.45	493.74	I	1537.82	1519.79	1520.79	769.41	14
10	1100.52	1082.51	1083.50	550.76	N	1424.73	1406.72	1407.71	712.87	13
11	1229.56	1211.55	1212.54	615.28	E	1310.69	1292.68	1293.68	655.84	12
12	1300.60	1282.59	1283.58	650.80	A	1181.64	1163.64	1164.61	591.32	11
13	1357.62	1339.61	1340.60	679.31	G	1110.61	1092.60	1093.59	555.80	10
14	1456.69	1438.68	1439.66	728.85	V	1053.59	1035.58	1036.55	527.29	9
15	1593.75	1575.74	1576.72	797.38	H	954.52	936.51	937.51	477.76	8
16	1692.82	1674.81	1675.79	846.91	V	817.46	799.45	800.43	409.23	7
17	1805.90	1787.89	1788.88	903.45	L	718.39	700.38	701.36	359.70	6
18	1965.93	1947.92	1948.91	983.47	C(+57.02)	605.31	587.30	588.28	303.15	5
19	2093.99	2075.98	2076.97	1047.50	Q	445.28	427.27	428.25	223.14	4
20	2151.01	2133.00	2133.99	1076.01	G	317.22	299.21	300.19	159.11	3
21	2264.10	2246.09	2247.07	1132.55	L	260.20	242.19	243.17	130.60	2
22					K	147.11	129.10	130.09	74.06	1

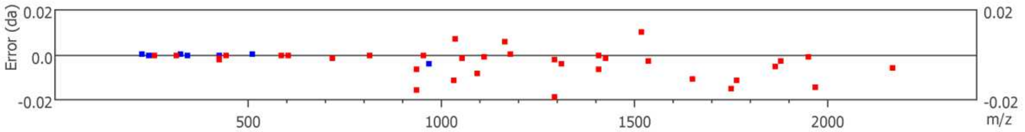

Brandsma C-A, *et al. Thorax* 2020; 75:180–183. doi: 10.1136/thoraxjnl-2019-213200

Peptide Sequence	Gene	Dataset	Sample	Score	ScanID
EQALQEAMEQLEELELER	SWAP70	1D25	COPD9	100.83	29732

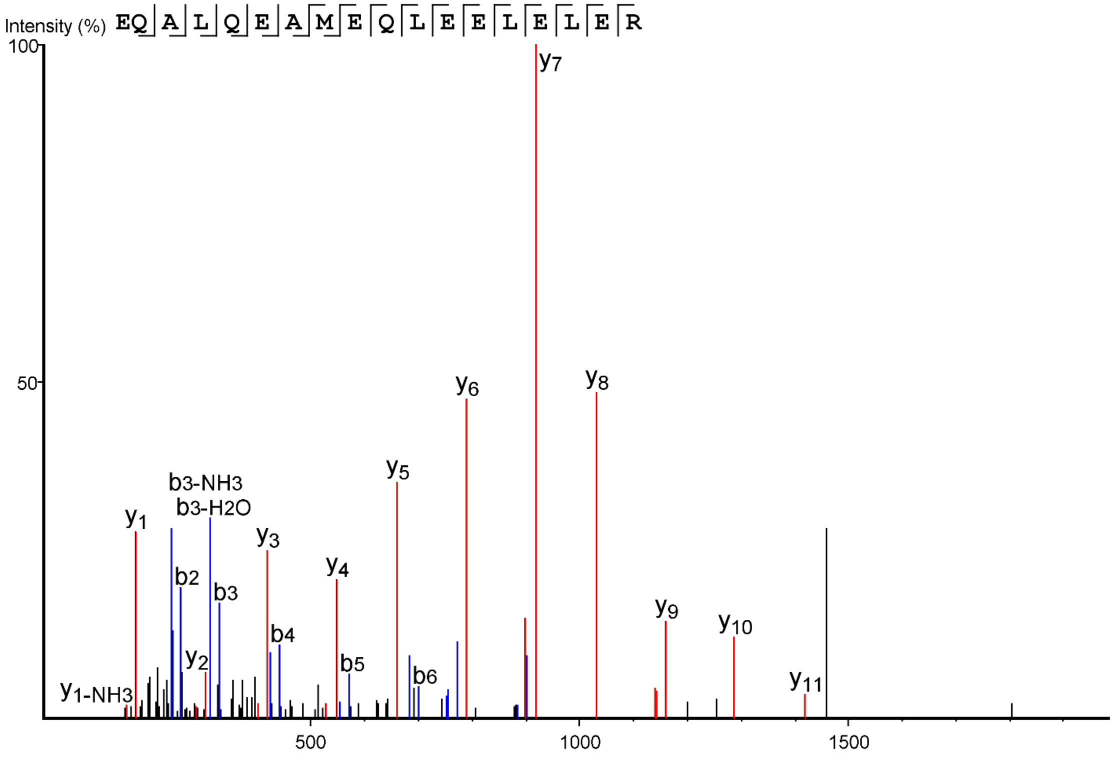

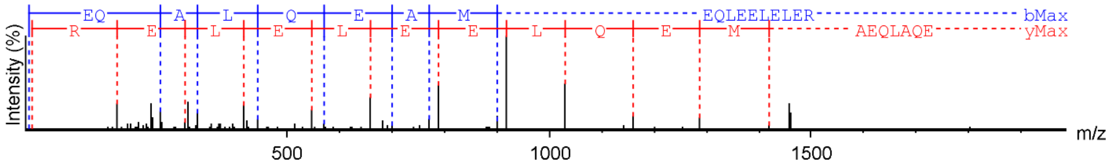

#	b	b-H2O	b-NH3	b (2+)	Seq	y	y-H2O	y-NH3	y (2+)	#
1	130.05	112.04	113.02	65.53	E					18
2	258.11	240.10	241.08	129.55	Q	2059.00	2040.99	2041.97	1030.00	17
3	329.15	311.13	312.12	165.07	A	1930.94	1912.93	1913.92	965.97	16
4	442.23	424.22	425.20	221.62	L	1859.91	1841.90	1842.88	930.45	15
5	570.29	552.28	553.26	285.64	Q	1746.82	1728.81	1729.79	873.91	14
6	699.33	681.32	682.30	350.17	E	1618.76	1600.75	1601.74	809.88	13
7	770.36	752.36	753.35	385.68	A	1489.72	1471.71	1472.69	745.36	12
8	901.41	883.40	884.39	451.20	M	1418.68	1400.67	1401.66	709.84	11
9	1030.45	1012.44	1013.42	515.73	E	1287.64	1269.63	1270.62	644.32	10
10	1158.51	1140.50	1141.48	579.76	Q	1158.60	1140.57	1141.58	579.80	9
11	1271.59	1253.58	1254.57	636.30	L	1030.54	1012.53	1013.51	515.77	8
12	1400.64	1382.63	1383.61	700.82	E	917.46	899.45	900.43	459.23	7
13	1529.68	1511.67	1512.65	765.34	E	788.42	770.40	771.37	394.71	6
14	1642.76	1624.75	1625.74	821.88	L	659.37	641.36	642.35	330.19	5
15	1771.81	1753.80	1754.78	886.40	E	546.29	528.28	529.26	273.64	4
16	1884.89	1866.88	1867.86	942.95	L	417.25	399.24	400.22	209.12	3
17	2013.93	1995.92	1996.91	1007.47	E	304.16	286.15	287.13	152.58	2
18					R	175.12	157.11	158.09	88.06	1

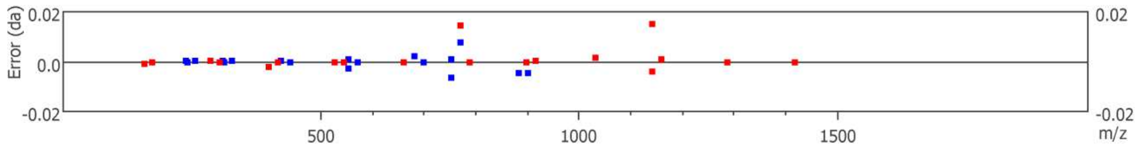

Brandsma C-A, *et al. Thorax* 2020; 75:180–183. doi: 10.1136/thoraxjnl-2019-213200

Peptide Sequence	Gene	Dataset	Sample	Score	ScanID
GATYNIIVEALKDQQR	FN1	2D25	Control6	109.33	15436

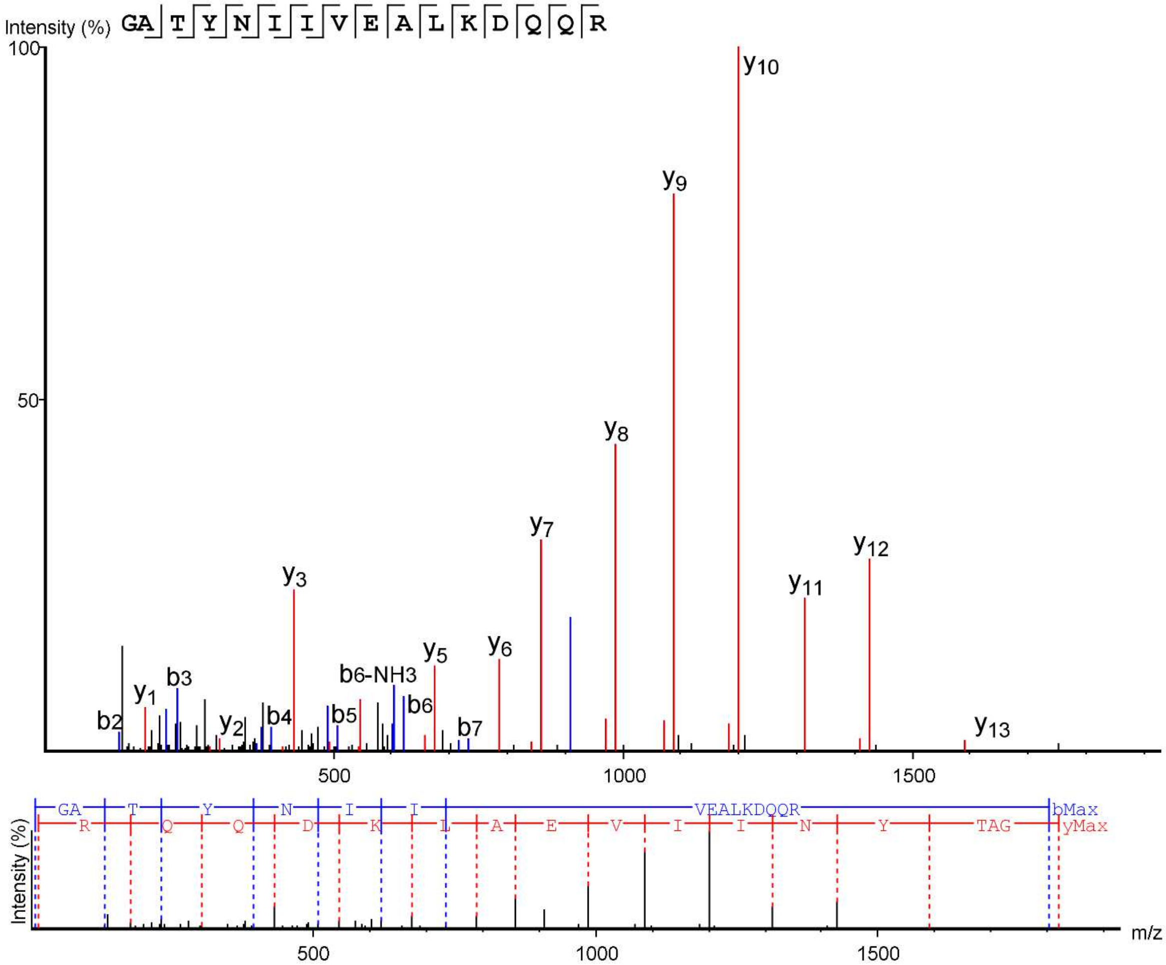

#	b	b-H2O	b-NH3	b (2+)	Seq	y	y-H2O	y-NH3	y (2+)	#
1	58.03	40.02	41.00	29.51	G					16
2	129.07	111.06	112.04	65.03	A	1761.95	1743.94	1744.92	881.47	15
3	230.11	212.10	213.09	115.56	T	1690.91	1672.90	1673.89	845.96	14
4	393.18	375.17	376.15	197.09	Y	1589.86	1571.85	1572.84	795.43	13
5	507.22	489.21	490.19	254.11	N	1426.80	1408.79	1409.79	713.90	12
6	620.30	602.29	603.28	310.65	I	1312.76	1294.75	1295.73	656.88	11
7	733.39	715.38	716.37	367.21	I	1199.67	1181.66	1182.65	600.34	10
8	832.46	814.45	815.43	416.73	V	1086.59	1068.58	1069.56	543.80	9
9	961.50	943.49	944.47	481.25	E	987.52	969.51	970.50	494.27	8
10	1032.54	1014.53	1015.51	516.77	A	858.48	840.47	841.45	429.74	7
11	1145.62	1127.61	1128.59	573.31	L	787.44	769.43	770.42	394.22	6
12	1273.72	1255.71	1256.69	637.36	K	674.36	656.35	657.33	337.68	5
13	1388.74	1370.73	1371.72	694.87	D	546.26	528.25	529.24	273.63	4
14	1516.80	1498.79	1499.77	758.90	Q	431.24	413.22	414.21	216.12	3
15	1644.86	1626.85	1627.83	822.93	Q	303.18	285.17	286.15	152.09	2
16					R	175.12	157.11	158.09	88.06	1

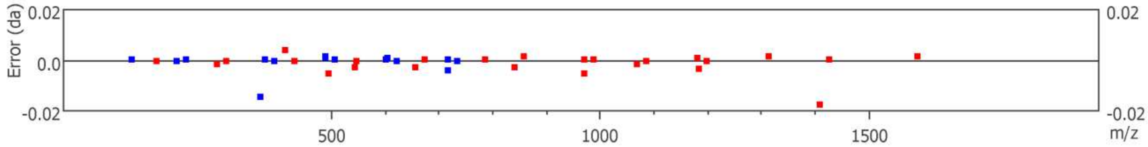

Brandsma C-A, *et al. Thorax* 2020; 75:180–183. doi: 10.1136/thoraxjnl-2019-213200

Peptide Sequence	Gene	Dataset	Sample	Score	ScanID
SDNCEDTPEAGYFAVAVVKK	TF	2D25	Control3	145.51	12809

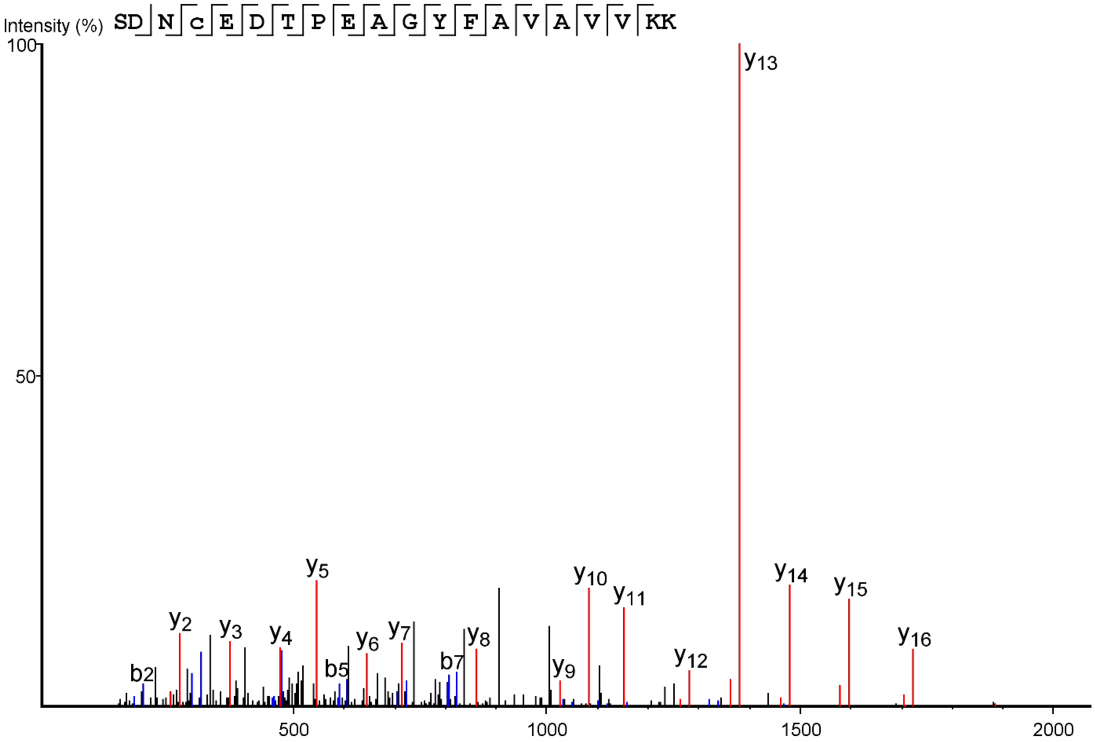

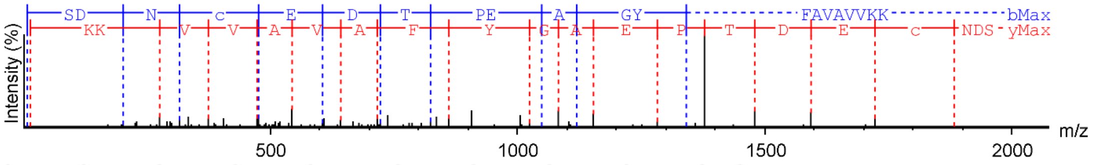

#	b	b-H2O	b-NH3	b (2+)	Seq	y	y-H2O	y-NH3	y (2+)	#
1	88.04	70.03	71.01	44.52	S					20
2	203.07	185.06	186.04	102.03	D	2112.99	2094.98	2095.96	1057.00	19
3	317.11	299.10	300.08	159.05	N	1997.96	1979.95	1980.94	999.48	18
4	477.14	459.13	460.11	239.07	C(+57.02)	1883.91	1865.91	1866.89	942.46	17
5	606.18	588.17	589.16	303.59	E	1723.89	1705.88	1706.88	862.45	16
6	721.21	703.20	704.18	361.10	D	1594.85	1576.84	1577.84	797.92	15
7	822.26	804.25	805.23	411.63	T	1479.82	1461.81	1462.79	740.41	14
8	919.31	901.30	902.28	460.16	P	1378.77	1360.76	1361.75	689.89	13
9	1048.36	1030.34	1031.33	524.68	E	1281.72	1263.71	1264.69	641.36	12
10	1119.39	1101.38	1102.36	560.20	A	1152.68	1134.67	1135.65	576.84	11
11	1176.41	1158.40	1159.38	588.71	G	1081.64	1063.63	1064.61	541.32	10
12	1339.48	1321.47	1322.45	670.24	Y	1024.62	1006.61	1007.59	512.81	9
13	1486.54	1468.55	1469.52	743.77	F	861.55	843.55	844.53	431.28	8
14	1557.58	1539.57	1540.55	779.29	A	714.49	696.48	697.46	357.74	7
15	1656.65	1638.64	1639.62	828.82	V	643.45	625.44	626.42	322.23	6
16	1727.69	1709.68	1710.66	864.34	A	544.38	526.37	527.35	272.69	5
17	1826.75	1808.74	1809.73	913.88	V	473.34	455.33	456.32	237.17	4
18	1925.82	1907.81	1908.80	963.41	V	374.28	356.27	357.25	187.64	3
19	2053.92	2035.91	2036.89	1027.46	K	275.21	257.20	258.18	138.10	2
20					K	147.11	129.10	130.09	74.06	1

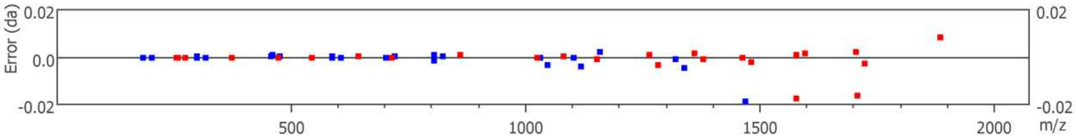

Peptide Sequence	Gene	Dataset	Sample	Score	ScanID
VMQVVDEKLPGLLGNFPGPFEEEMK	ASAH1	1D25	Control10	134.27	26054

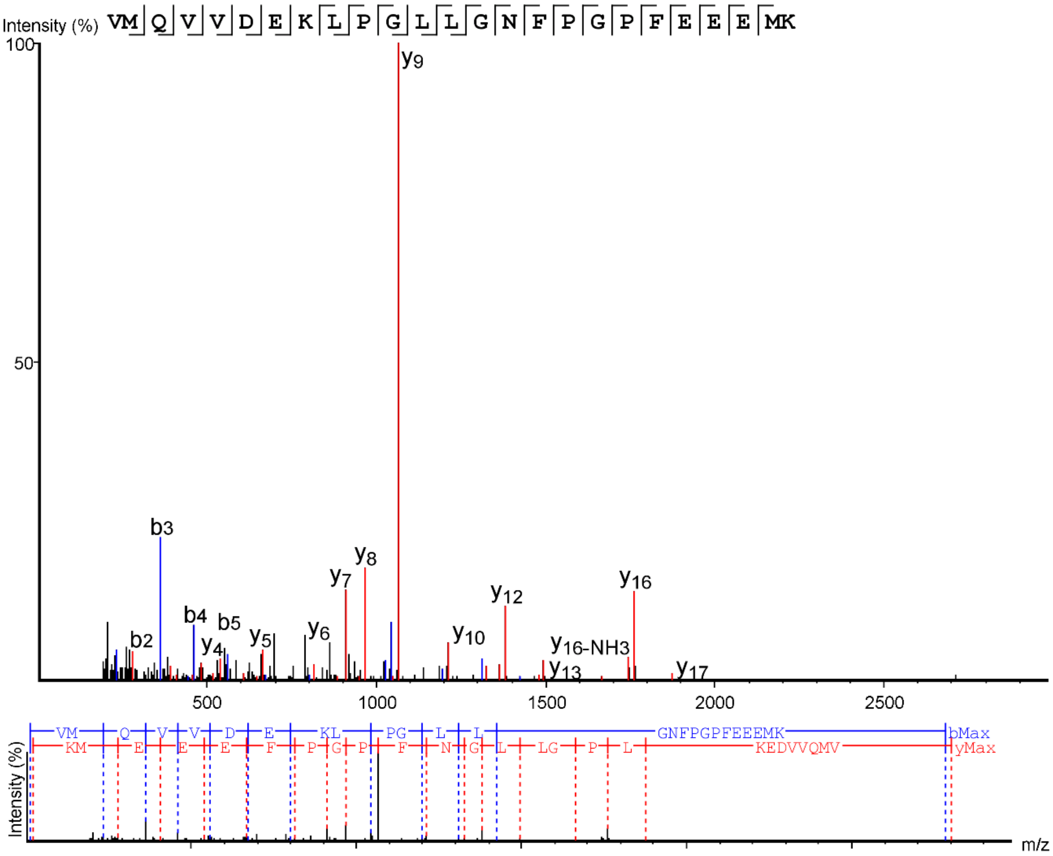

#	b	b-H2O	b-NH3	b (2+)	Seq	y	y-H2O	y-NH3	y (2+)	#
1	100.08	82.07	83.05	50.54	V					25
2	231.12	213.11	214.09	116.06	M	2704.34	2686.33	2687.31	1352.67	24
3	359.18	341.16	342.15	180.09	Q	2573.30	2555.28	2556.27	1287.15	23
4	458.24	440.23	441.22	229.62	V	2445.24	2427.23	2428.21	1223.12	22
5	557.31	539.30	540.29	279.16	V	2346.17	2328.16	2329.14	1173.58	21
6	672.33	654.33	655.31	336.67	D	2247.10	2229.09	2230.07	1124.05	20
7	801.38	783.37	784.35	401.19	E	2132.07	2114.06	2115.05	1066.54	19
8	929.48	911.47	912.45	465.24	K	2003.03	1985.02	1986.00	1002.02	18
9	1042.56	1024.55	1025.53	521.78	L	1874.92	1856.93	1857.91	937.97	17
10	1139.61	1121.60	1122.59	570.31	P	1761.85	1743.84	1744.83	881.43	16
11	1196.63	1178.62	1179.61	598.82	G	1664.80	1646.79	1647.77	832.90	15
12	1309.72	1291.71	1292.69	655.36	L	1607.78	1589.77	1590.75	804.39	14
13	1422.80	1404.79	1405.78	711.90	L	1494.69	1476.68	1477.66	747.85	13
14	1479.82	1461.81	1462.80	740.41	G	1381.61	1363.60	1364.59	691.30	12
15	1593.87	1575.86	1576.84	797.43	N	1324.59	1306.58	1307.56	662.79	11
16	1740.94	1722.93	1723.91	870.97	F	1210.54	1192.52	1193.52	605.77	10
17	1837.99	1819.98	1820.96	919.49	P	1063.48	1045.47	1046.47	532.24	9
18	1895.01	1877.00	1877.98	948.01	G	966.42	948.42	949.40	483.71	8
19	1992.06	1974.05	1975.04	996.53	P	909.40	891.39	892.38	455.20	7
20	2139.13	2121.12	2122.10	1070.07	F	812.35	794.34	795.32	406.67	6
21	2268.17	2250.16	2251.15	1134.59	E	665.28	647.27	648.25	333.14	5
22	2397.22	2379.21	2380.19	1199.11	E	536.24	518.23	519.21	268.62	4
23	2526.26	2508.25	2509.23	1263.63	E	407.20	389.18	390.17	204.10	3
24	2657.30	2639.29	2640.27	1329.15	M	278.15	260.14	261.13	139.58	2
25					K	147.11	129.10	130.09	74.06	1

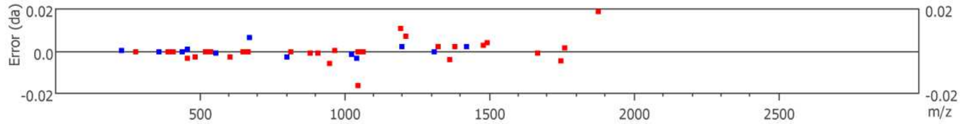

Peptide Sequence	Gene	Dataset	Sample	Score	ScanID
AVDQSVLLMKPDAELSASSVYNLLPEKDLTGFPGPLNDQDDEDCINR	A2M	1D25	Control3	200	28282

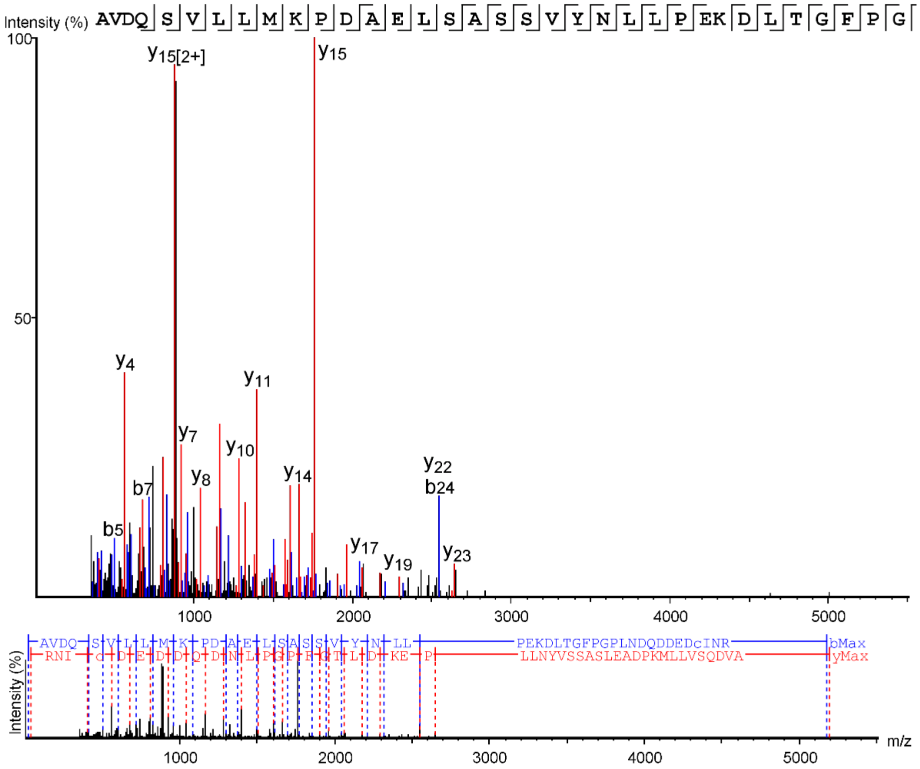

#	b	b-H2O	b-NH3	b (2+)	Seq	y	y-H2O	y-NH3	y (2+)	#
1	72.04	54.03	55.02	36.52	A					47
2	171.11	153.10	154.09	86.06	V	5118.46	5100.45	5101.43	2559.73	46
3	286.14	268.13	269.11	143.57	D	5019.39	5001.38	5002.36	2510.19	45
4	414.20	396.19	397.17	207.60	Q	4904.36	4886.35	4887.33	2452.68	44
5	501.23	483.22	484.20	251.12	S	4776.30	4758.29	4759.27	2388.65	43
6	600.30	582.29	583.29	300.65	V	4689.27	4671.26	4672.24	2345.14	42
7	713.38	695.38	696.35	357.21	L	4590.20	4572.19	4573.17	2295.60	41
8	826.46	808.45	809.46	413.73	L	4477.12	4459.11	4460.09	2239.06	40
9	957.50	939.49	940.49	479.26	M	4364.03	4346.02	4347.01	2182.52	39
10	1085.59	1067.58	1068.58	543.29	K	4232.99	4214.98	4215.97	2117.00	38
11	1182.66	1164.65	1165.63	591.83	P	4104.90	4086.89	4087.87	2052.95	37
12	1297.69	1279.67	1280.66	649.34	D	4007.84	3989.83	3990.82	2004.42	36
13	1368.73	1350.71	1351.69	684.86	A	3892.82	3874.81	3875.79	1946.93	35
14	1497.76	1479.75	1480.74	749.38	E	3821.78	3803.77	3804.75	1911.39	34
15	1610.85	1592.84	1593.82	805.93	L	3692.74	3674.73	3675.71	1846.87	33
16	1697.89	1679.86	1680.85	849.44	S	3579.65	3561.64	3562.63	1790.33	32
17	1768.92	1750.89	1751.89	884.96	A	3492.62	3474.61	3475.60	1746.80	31
18	1855.94	1837.95	1838.92	928.48	S	3421.59	3403.57	3404.56	1711.31	30
19	1942.98	1924.95	1925.96	971.99	S	3334.55	3316.54	3317.53	1667.78	29
20	2042.03	2024.02	2025.02	1021.52	V	3247.52	3229.51	3230.49	1624.26	28
21	2205.12	2187.10	2188.08	1103.06	Y	3148.45	3130.44	3131.43	1574.73	27
22	2319.16	2301.14	2302.13	1160.08	N	2985.39	2967.38	2968.36	1493.21	26
23	2432.24	2414.23	2415.21	1216.63	L	2871.35	2853.34	2854.32	1436.17	25
24	2545.31	2527.31	2528.30	1273.16	L	2758.26	2740.25	2741.24	1379.64	24
25	2642.38	2624.36	2625.35	1321.69	P	2645.16	2627.17	2628.14	1323.10	23
26	2771.42	2753.41	2754.39	1386.21	E	2548.13	2530.11	2531.10	1274.56	22
27	2899.51	2881.50	2882.49	1450.26	K	2419.08	2401.07	2402.06	1210.04	21
28	3014.54	2996.53	2997.51	1507.77	D	2290.98	2272.98	2273.96	1145.99	20
29	3127.62	3109.61	3110.60	1564.30	L	2175.95	2157.95	2158.93	1088.48	19
30	3228.67	3210.66	3211.64	1614.84	T	2062.87	2044.87	2045.85	1031.94	18
31	3285.69	3267.68	3268.67	1643.34	G	1961.85	1943.82	1944.80	981.41	17
32	3432.76	3414.75	3415.73	1716.89	F	1904.80	1886.80	1887.78	952.91	16
33	3529.81	3511.80	3512.79	1765.41	P	1757.76	1739.72	1740.72	879.37	15
34	3586.84	3568.82	3569.81	1793.92	G	1660.69	1642.68	1643.66	830.85	14
35	3683.89	3665.88	3666.86	1842.44	P	1603.67	1585.64	1586.63	802.34	13
36	3796.97	3778.96	3779.95	1898.99	L	1506.62	1488.60	1489.59	753.81	12
37	3911.02	3893.00	3893.99	1956.01	N	1393.53	1375.52	1376.50	697.27	11
38	4026.04	4008.03	4009.01	2013.52	D	1279.48	1261.47	1262.46	640.24	10
39	4154.10	4136.09	4137.07	2077.55	Q	1164.46	1146.44	1147.44	582.73	9
40	4269.13	4251.12	4252.10	2135.06	D	1036.40	1018.38	1019.37	518.70	8
41	4384.15	4366.14	4367.13	2192.58	D	921.37	903.36	904.35	461.19	7
42	4513.20	4495.19	4496.17	2257.10	E	806.34	788.33	789.32	403.67	6
43	4628.22	4610.21	4611.20	2314.61	D	677.30	659.29	660.28	339.15	5
44	4788.25	4770.24	4771.23	2394.63	C(+57.02)	562.28	544.27	545.25	281.64	4
45	4901.34	4883.33	4884.31	2451.17	I	402.25	384.24	385.22	201.62	3
46	5015.38	4997.37	4998.35	2508.19	N	289.16	271.15	272.13	145.08	2
47					R	175.12	157.11	158.09	88.06	1

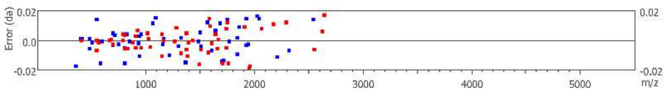

Peptide Sequence	Gene	Dataset	Sample	Score	ScanID
DSEDVSERDSDKEMATK	AKAP12	2D25	Control3	107.08	3190

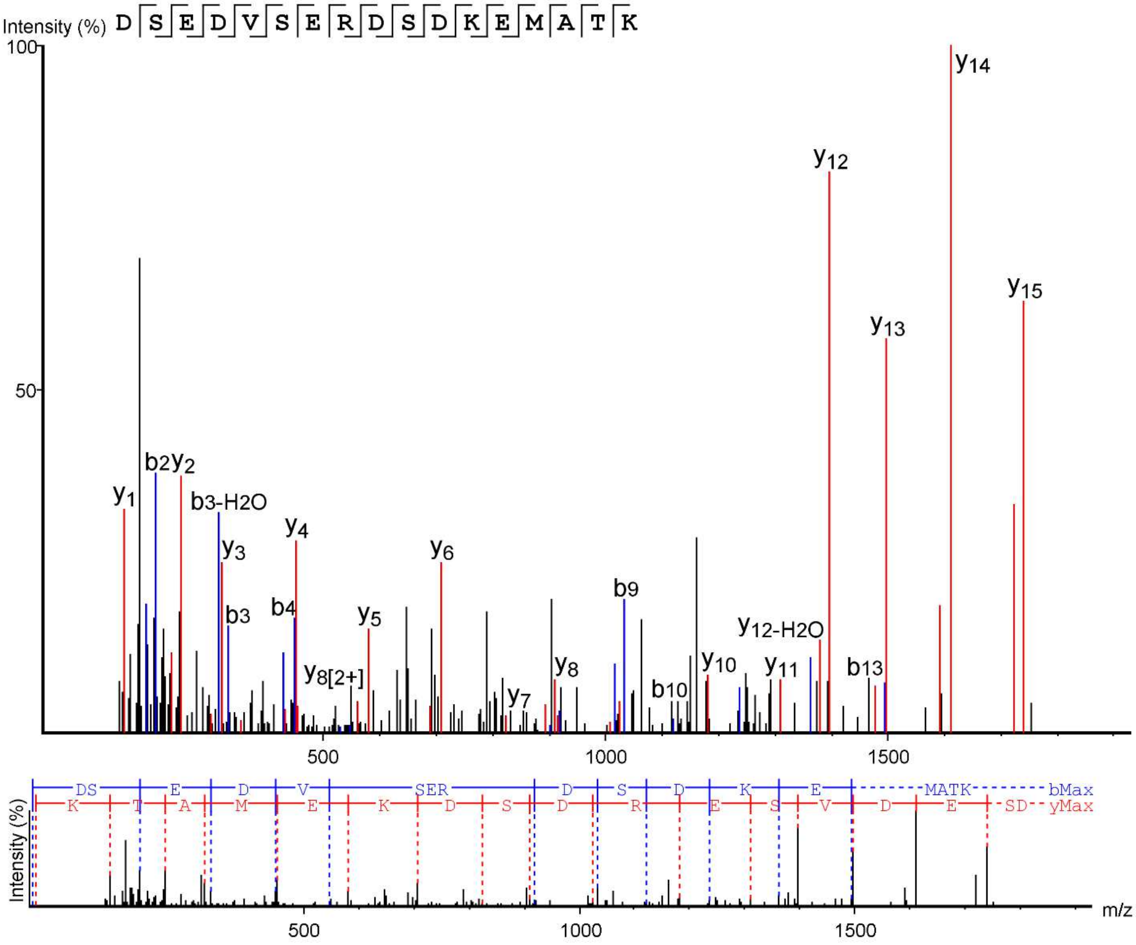

#	b	b-H2O	b-NH3	b (2+)	Seq	y	y-H2O	y-NH3	y (2+)	#
1	116.03	98.02	99.01	58.52	D					17
2	203.07	185.06	186.04	102.03	S	1826.81	1808.80	1809.78	913.91	16
3	332.11	314.10	315.08	166.55	E	1739.77	1721.76	1722.75	870.39	15
4	447.14	429.13	430.11	224.07	D	1610.73	1592.72	1593.71	805.87	14
5	546.20	528.19	529.18	273.60	V	1495.71	1477.69	1478.68	748.35	13
6	633.24	615.23	616.21	317.12	S	1396.63	1378.62	1379.61	698.82	12
7	762.28	744.27	745.25	381.64	E	1309.61	1291.59	1292.58	655.30	11
8	918.37	900.37	901.35	459.69	R	1180.56	1162.55	1163.54	590.78	10
9	1033.40	1015.40	1016.38	517.20	D	1024.46	1006.45	1007.43	512.73	9
10	1120.44	1102.43	1103.41	560.72	S	909.43	891.42	892.42	455.22	8
11	1235.47	1217.46	1218.44	618.23	D	822.40	804.39	805.38	411.70	7
12	1363.58	1345.55	1346.53	682.28	K	707.38	689.36	690.35	354.19	6
13	1492.62	1474.59	1475.58	746.80	E	579.28	561.27	562.25	290.14	5
14	1623.64	1605.63	1606.62	812.32	M	450.24	432.23	433.21	225.62	4
15	1694.68	1676.67	1677.65	847.84	A	319.20	301.19	302.17	160.10	3
16	1795.73	1777.72	1778.70	898.36	T	248.16	230.15	231.13	124.58	2
17					K	147.11	129.10	130.09	74.06	1

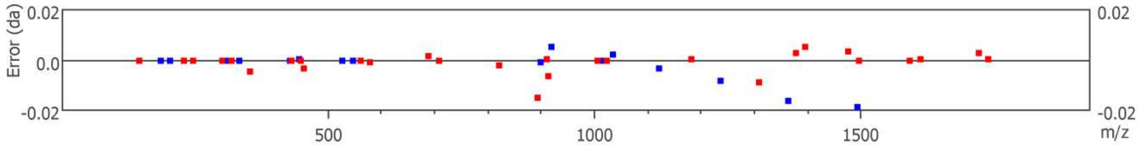

Brandsma C-A, *et al. Thorax* 2020; 75:180–183. doi: 10.1136/thoraxjnl-2019-213200

Peptide Sequence	Gene	Dataset	Sample	Score	ScanID
EVQLVESGGGVVQPGGSLR	IGHV3-9	2D25	Control4	111.36	12235

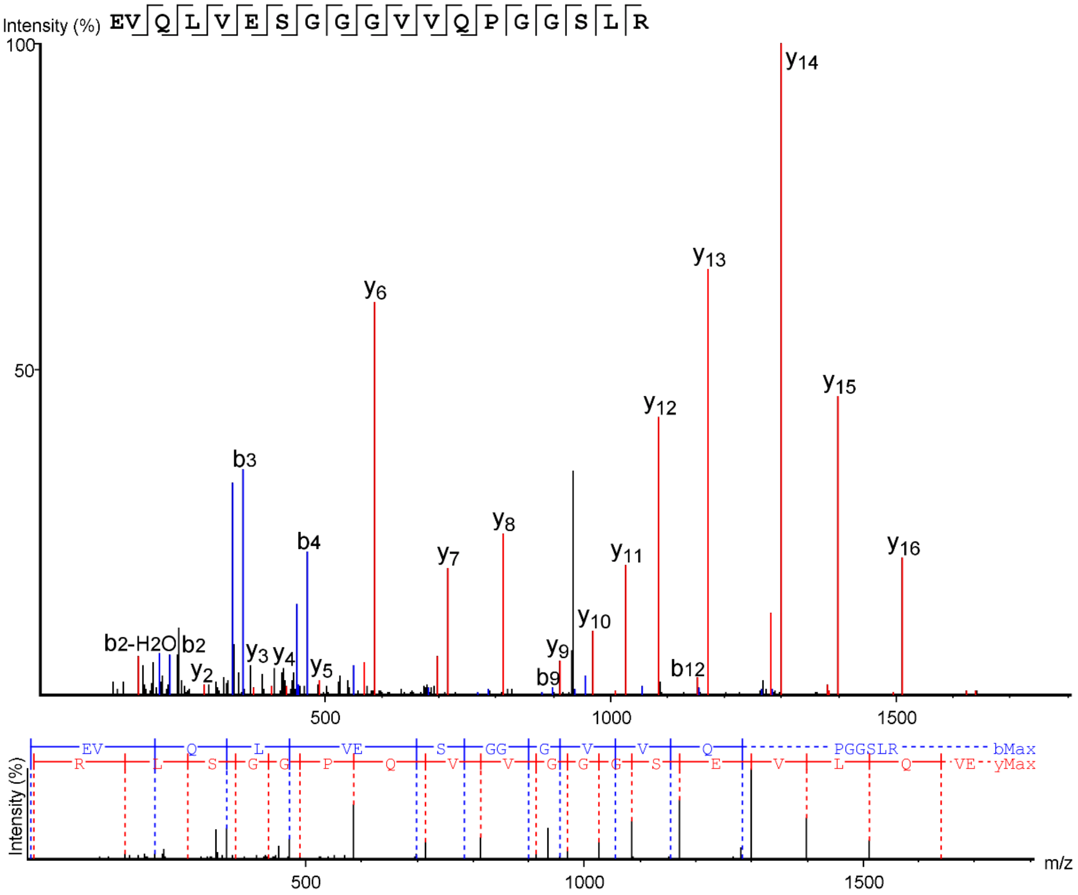

#	b	b-H2O	b-NH3	b (2+)	Seq	y	y-H2O	y-NH3	y (2+)	#
1	130.05	112.04	113.02	65.53	E					19
2	229.12	211.11	212.09	115.06	V	1738.94	1720.93	1721.92	869.97	18
3	357.18	339.17	340.15	179.09	Q	1639.89	1621.87	1622.86	820.44	17
4	470.26	452.25	453.23	235.63	L	1511.82	1493.81	1494.79	756.41	16
5	569.33	551.32	552.30	285.16	V	1398.73	1380.73	1381.71	699.87	15
6	698.37	680.36	681.35	349.69	E	1299.66	1281.65	1282.66	650.33	14
7	785.40	767.39	768.38	393.20	S	1170.62	1152.61	1153.61	585.81	13
8	842.43	824.42	825.40	421.71	G	1083.59	1065.58	1066.56	542.30	12
9	899.45	881.43	882.42	450.22	G	1026.57	1008.56	1009.54	513.78	11
10	956.47	938.45	939.44	478.73	G	969.55	951.54	952.52	485.27	10
11	1055.53	1037.53	1038.51	528.27	V	912.53	894.52	895.50	456.76	9
12	1154.60	1136.60	1137.58	577.80	V	813.46	795.45	796.43	407.23	8
13	1282.66	1264.64	1265.64	641.83	Q	714.39	696.38	697.36	357.69	7
14	1379.72	1361.71	1362.69	690.36	P	586.33	568.32	569.30	293.67	6
15	1436.74	1418.73	1419.71	718.87	G	489.28	471.27	472.25	245.14	5
16	1493.76	1475.75	1476.73	747.38	G	432.26	414.25	415.23	216.63	4
17	1580.79	1562.78	1563.77	790.90	S	375.23	357.22	358.21	188.12	3
18	1693.88	1675.87	1676.85	847.44	L	288.20	270.19	271.18	144.60	2
19					R	175.12	157.11	158.09	88.06	1

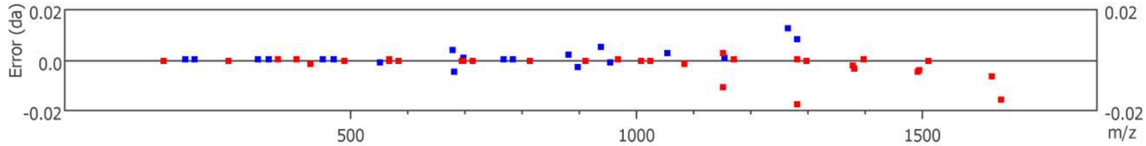

Brandsma C-A, *et al. Thorax* 2020; 75:180–183. doi: 10.1136/thoraxjnl-2019-213200

Peptide Sequence	Gene	Dataset	Sample	Score	ScanID
SYPASDHTTPLSR	NID2	2D25	Control2	98.27	5632

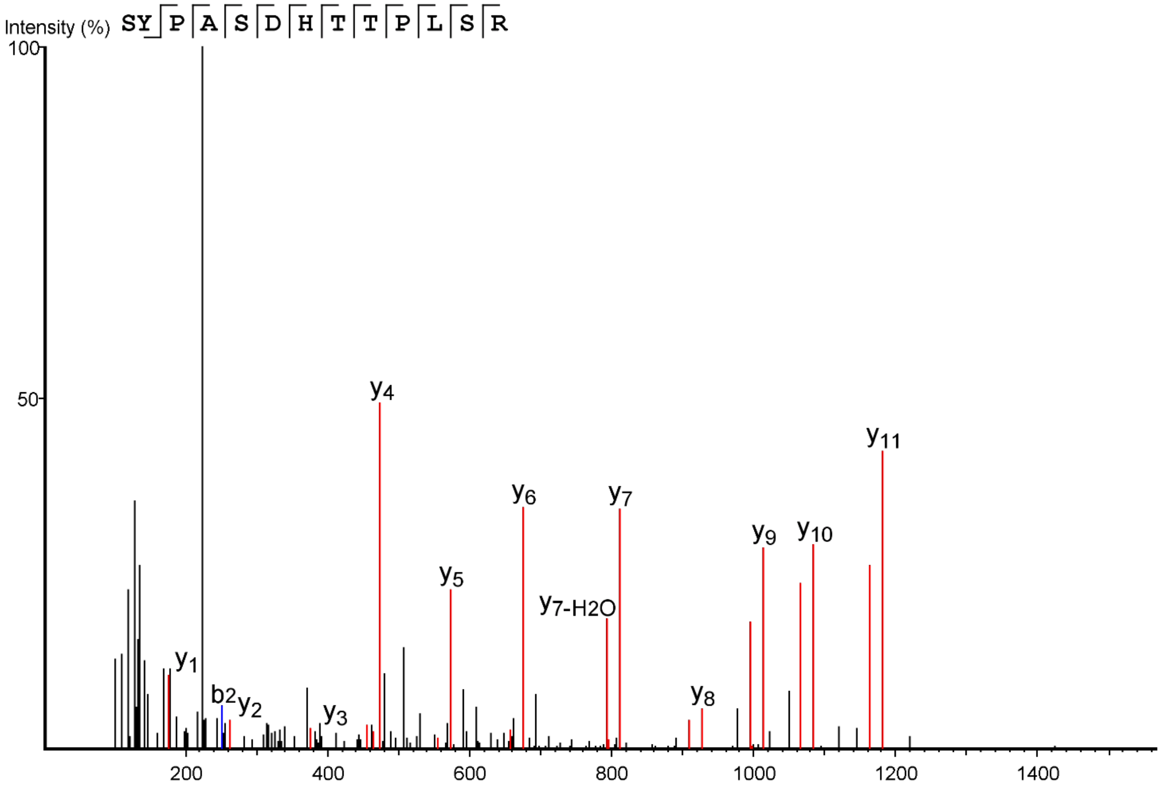

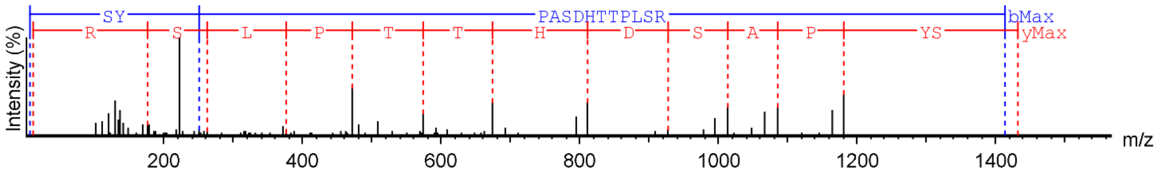

#	b	b-H2O	b-NH3	b (2+)	Seq	y	y-H2O	y-NH3	y (2+)	#
1	88.04	70.03	71.01	44.52	S					13
2	251.10	233.09	234.08	126.05	Y	1344.65	1326.64	1327.63	672.83	12
3	348.16	330.15	331.13	174.58	P	1181.59	1163.58	1164.56	591.30	11
4	419.19	401.18	402.17	210.10	A	1084.54	1066.52	1067.51	542.77	10
5	506.23	488.21	489.20	253.61	S	1013.50	995.49	996.49	507.25	9
6	621.25	603.24	604.23	311.13	D	926.47	908.45	909.44	463.74	8
7	758.31	740.30	741.28	379.66	H	811.44	793.44	794.43	406.22	7
8	859.36	841.35	842.33	430.18	T	674.38	656.37	657.36	337.69	6
9	960.41	942.40	943.38	480.70	T	573.34	555.33	556.31	287.17	5
10	1057.46	1039.45	1040.43	529.23	P	472.29	454.28	455.26	236.64	4
11	1170.54	1152.53	1153.52	585.77	L	375.23	357.22	358.21	188.12	3
12	1257.58	1239.56	1240.55	629.29	S	262.15	244.14	245.12	131.58	2
13					R	175.12	157.11	158.09	88.06	1

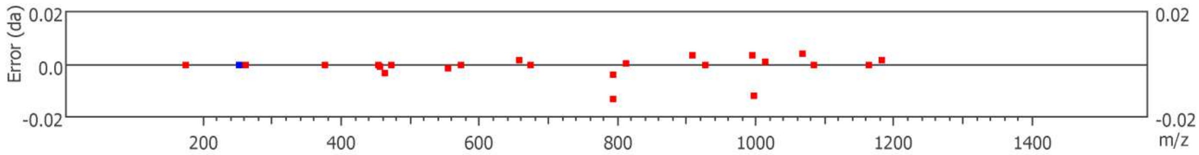

Brandsma C-A, *et al. Thorax* 2020; 75:180–183. doi: 10.1136/thoraxjnl-2019-213200

Peptide Sequence	Gene	Dataset	Sample	Score	ScanID
ADIDVSGPKVDVDVPDVNIEGPDAK	AHNAK	1D50	Control2	138.93	21200

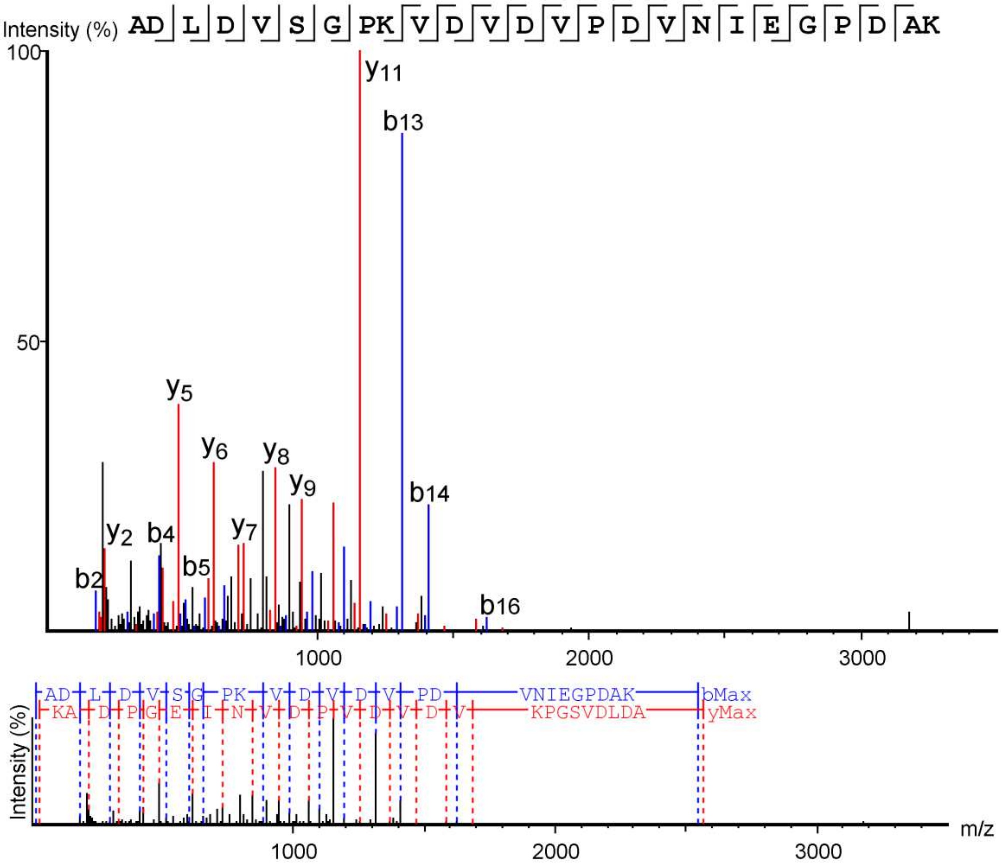

#	b	b-H2O	b-NH3	b (2+)	Seq	y	y-H2O	y-NH3	y (2+)	#
1	72.04	54.03	55.02	36.52	A					25
2	187.07	169.06	170.04	94.04	D	2493.24	2475.22	2476.21	1247.12	24
3	300.16	282.15	283.13	150.58	L	2378.21	2360.20	2361.18	1189.60	23
4	415.18	397.17	398.16	208.09	D	2265.12	2247.11	2248.10	1133.06	22
5	514.25	496.24	497.22	257.63	V	2150.10	2132.09	2133.07	1075.55	21
6	601.28	583.27	584.26	301.14	S	2051.03	2033.02	2034.00	1026.01	20
7	658.30	640.29	641.28	329.65	G	1964.00	1945.99	1946.97	982.50	19
8	755.36	737.35	738.33	378.18	P	1906.98	1888.97	1889.95	953.99	18
9	883.45	865.44	866.43	442.23	K	1809.92	1791.91	1792.90	905.46	17
10	982.52	964.51	965.49	491.76	V	1681.82	1663.82	1664.80	841.41	16
11	1097.55	1079.53	1080.54	549.28	D	1582.76	1564.75	1565.73	791.88	15
12	1196.62	1178.61	1179.59	598.81	V	1467.73	1449.72	1450.71	734.37	14
13	1311.64	1293.63	1294.62	656.32	D	1368.67	1350.65	1351.64	684.83	13
14	1410.71	1392.70	1393.68	705.86	V	1253.64	1235.63	1236.61	627.32	12
15	1507.76	1489.75	1490.74	754.38	P	1154.57	1136.56	1137.55	577.78	11
16	1622.80	1604.79	1605.76	811.90	D	1057.52	1039.50	1040.49	529.26	10
17	1721.86	1703.85	1704.83	861.44	V	942.49	924.48	925.46	471.74	9
18	1835.90	1817.89	1818.88	918.45	N	843.42	825.41	826.40	422.21	8
19	1948.99	1930.98	1931.96	974.99	I	729.38	711.37	712.35	365.19	7
20	2078.03	2060.02	2061.00	1039.50	E	616.29	598.28	599.27	308.65	6
21	2135.05	2117.04	2118.02	1068.03	G	487.25	469.23	470.22	244.13	5
22	2232.10	2214.09	2215.08	1116.55	P	430.23	412.22	413.20	215.61	4
23	2347.13	2329.12	2330.10	1174.07	D	333.18	315.17	316.15	167.09	3
24	2418.17	2400.16	2401.14	1209.58	A	218.15	200.14	201.12	109.57	2
25					K	147.11	129.10	130.09	74.06	1

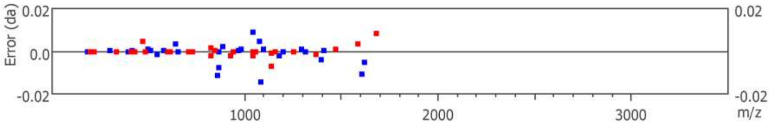

Brandsma C-A, *et al. Thorax* 2020; 75:180–183. doi: 10.1136/thoraxjnl-2019-213200

Peptide Sequence	Gene	Dataset	Sample	Score	ScanID
SAVQGPPDRDLCGCYSVSSVLPGCAEPWNHGK	IGHA1	2D25	Control8	200	14520

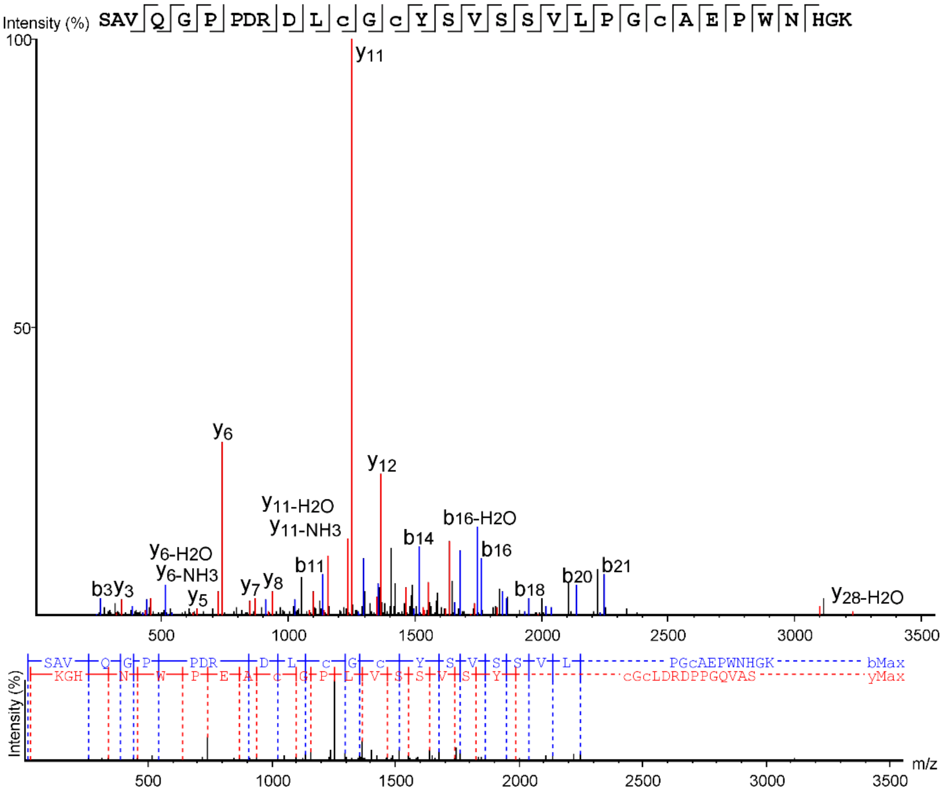

#	b	b-H2O	b-NH3	b (2+)	Seq	y	y-H2O	y-NH3	y (2+)	#
1	88.04	70.03	71.01	44.52	S					32
2	159.08	141.07	142.05	80.04	A	3413.55	3395.54	3396.52	1707.27	31
3	258.14	240.13	241.12	129.57	V	3342.51	3324.50	3325.48	1671.75	30
4	386.20	368.19	369.18	193.60	Q	3243.44	3225.43	3226.42	1622.22	29
5	443.22	425.21	426.20	222.11	G	3115.38	3097.38	3098.35	1558.19	28
6	540.28	522.27	523.25	270.64	P	3058.36	3040.35	3041.33	1529.69	27
7	637.33	619.32	620.30	319.17	P	2961.31	2943.30	2944.28	1481.15	26
8	752.36	734.35	735.33	376.68	D	2864.25	2846.24	2847.23	1432.63	25
9	908.46	890.45	891.43	454.73	R	2749.23	2731.22	2732.20	1375.11	24
10	1023.49	1005.48	1006.46	512.25	D	2593.13	2575.12	2576.10	1297.06	23
11	1136.57	1118.56	1119.54	568.78	L	2478.10	2460.09	2461.07	1239.55	22
12	1296.60	1278.57	1279.58	648.80	C(+57.02)	2365.02	2347.01	2347.99	1183.01	21
13	1353.62	1335.61	1336.60	677.31	G	2204.99	2186.97	2187.96	1102.99	20
14	1513.65	1495.63	1496.64	757.33	C(+57.02)	2147.96	2129.95	2130.94	1074.48	19
15	1676.72	1658.70	1659.69	838.86	Y	1987.92	1969.92	1970.91	994.47	18
16	1763.74	1745.73	1746.72	882.37	S	1824.88	1806.86	1807.84	912.93	17
17	1862.83	1844.81	1845.79	931.91	V	1737.84	1719.83	1720.80	869.42	16
18	1949.86	1931.84	1932.82	975.42	S	1638.77	1620.76	1621.76	819.88	15
19	2036.88	2018.86	2019.85	1018.94	S	1551.74	1533.73	1534.71	776.37	14
20	2135.94	2117.94	2118.92	1068.47	V	1464.70	1446.69	1447.68	732.85	13
21	2249.03	2231.03	2232.01	1125.02	L	1365.64	1347.62	1348.62	683.32	12
22	2346.09	2328.08	2329.06	1173.54	P	1252.55	1234.54	1235.53	626.78	11
23	2403.11	2385.10	2386.08	1202.05	G	1155.50	1137.49	1138.48	578.25	10
24	2563.14	2545.13	2546.11	1282.07	C(+57.02)	1098.48	1080.46	1081.45	549.74	9
25	2634.18	2616.16	2617.15	1317.59	A	938.45	920.44	921.42	469.72	8
26	2763.22	2745.21	2746.19	1382.11	E	867.41	849.40	850.38	434.21	7
27	2860.27	2842.26	2843.24	1430.64	P	738.37	720.36	721.34	369.68	6
28	3046.35	3028.34	3029.32	1523.67	W	641.32	623.30	624.29	321.16	5
29	3160.39	3142.38	3143.37	1580.70	N	455.24	437.23	438.21	228.12	4
30	3297.45	3279.44	3280.42	1649.23	H	341.19	323.18	324.17	171.10	3
31	3354.47	3336.46	3337.45	1677.74	G	204.13	186.12	187.11	102.57	2
32					K	147.11	129.10	130.09	74.06	1

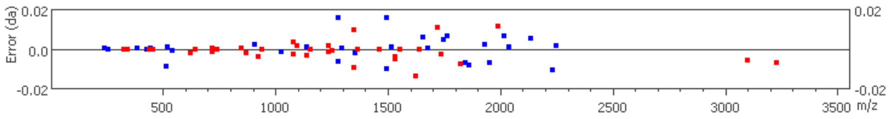

Brandsma C-A, *et al. Thorax* 2020; 75:180–183. doi: 10.1136/thoraxjnl-2019-213200

Peptide Sequence	Gene	Dataset	Sample	Score	ScanID
MHKSSMPEEVK	CFL1	1D25	Control10	36.09	11218

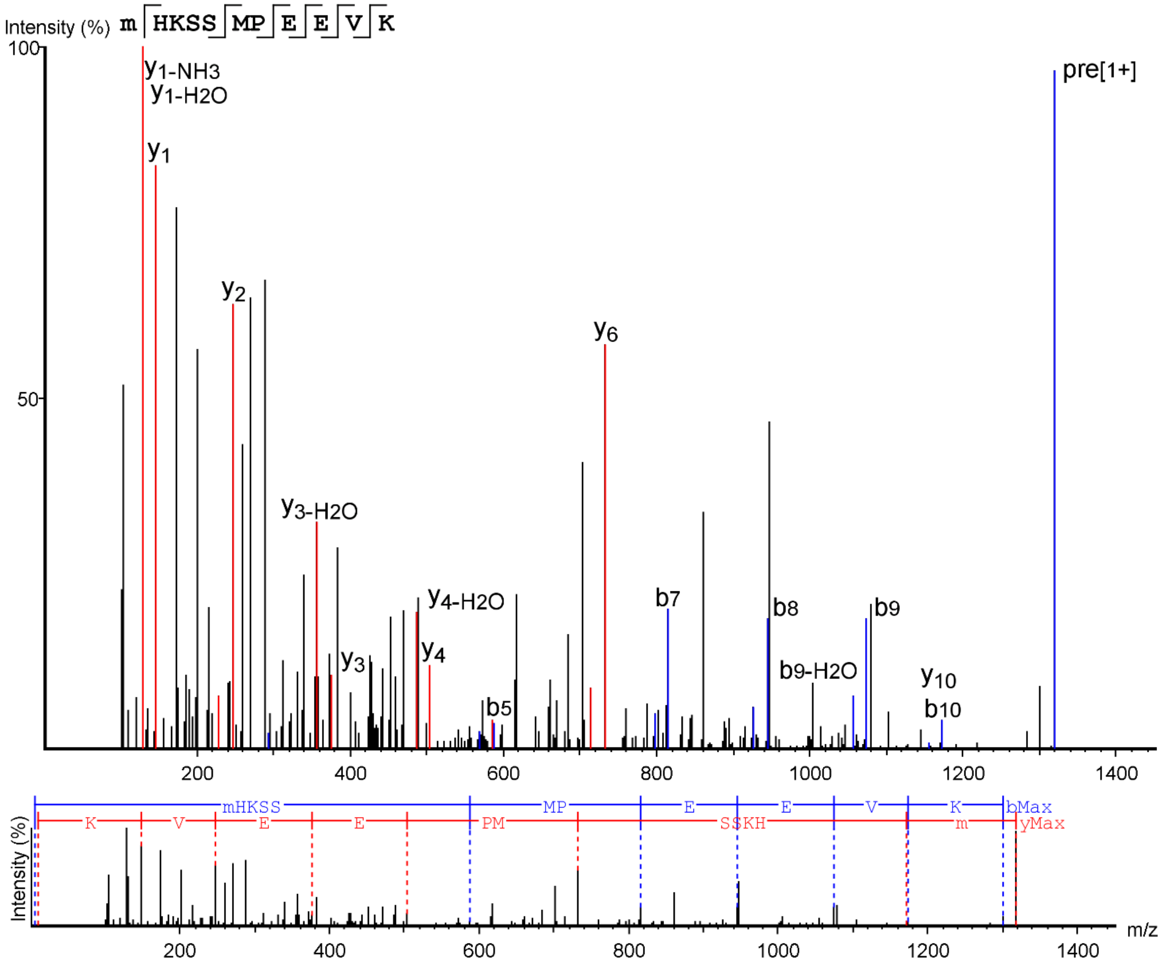

#	b	b-H2O	b-NH3	b (2+)	Seq	y	y-H2O	y-NH3	y (2+)	#
1	148.04	130.03	131.02	74.52	M(+15.99)					11
2	285.10	267.09	268.08	143.05	H	1171.59	1153.57	1154.55	586.27	10
3	413.20	395.19	396.17	207.10	K	1034.52	1016.51	1017.49	517.76	9
4	500.23	482.22	483.20	250.61	S	906.42	888.41	889.40	453.71	8
5	587.25	569.24	570.23	294.14	S	819.39	801.38	802.36	410.20	7
6	718.30	700.29	701.27	359.65	M	732.38	714.37	715.33	366.68	6
7	815.36	797.35	798.33	408.18	P	601.32	583.31	584.29	301.16	5
8	944.40	926.39	927.37	472.70	E	504.27	486.26	487.24	252.63	4
9	1073.44	1055.43	1056.41	537.22	E	375.22	357.21	358.20	188.11	3
10	1172.51	1154.50	1155.48	586.75	V	246.18	228.17	229.15	123.59	2
11					K	147.11	129.10	130.09	74.06	1

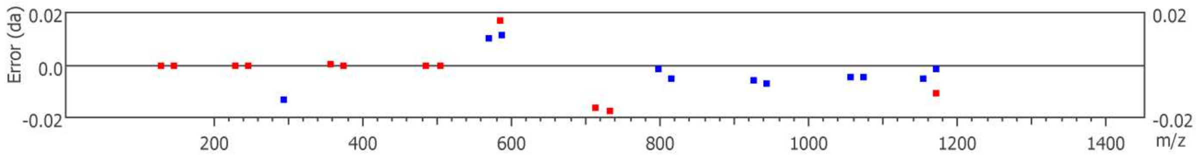

Brandsma C-A, *et al. Thorax* 2020; 75:180–183. doi: 10.1136/thoraxjnl-2019-213200

Peptide Sequence	Gene	Dataset	Sample	Score	ScanID
SVFKPFIFGVGVAQAPQVLSPTFGAQDPVR	SCRN2	1D25	Control9	124.78	26504

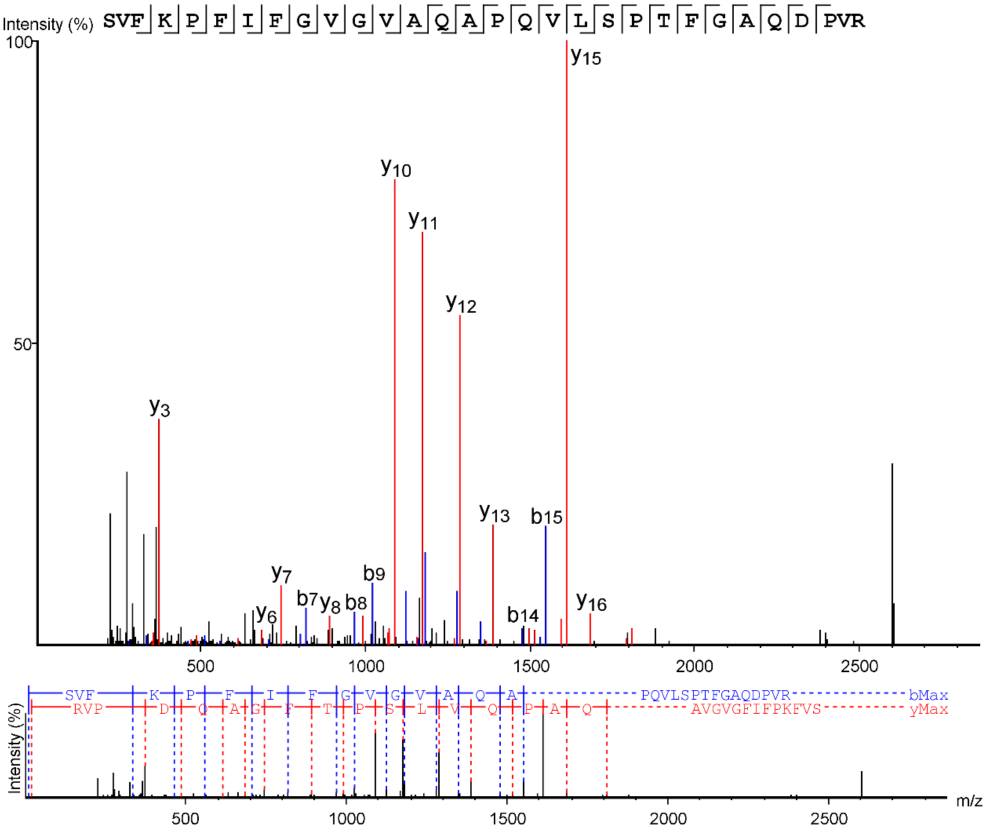

#	b	b-H2O	b-NH3	b (2+)	Seq	y	y-H2O	y-NH3	y (2+)	#
1	88.04	70.03	71.01	44.52	S					30
2	187.11	169.10	170.08	94.05	V	3072.67	3054.66	3055.64	1536.83	29
3	334.18	316.17	317.15	167.59	F	2973.60	2955.59	2956.57	1487.30	28
4	462.27	444.26	445.24	231.64	K	2826.53	2808.52	2809.50	1413.77	27
5	559.33	541.31	542.30	280.17	P	2698.44	2680.42	2681.41	1349.72	26
6	706.39	688.38	689.37	353.70	F	2601.38	2583.37	2584.36	1301.19	25
7	819.48	801.47	802.45	410.24	I	2454.31	2436.30	2437.29	1227.66	24
8	966.55	948.53	949.52	483.77	F	2341.23	2323.22	2324.20	1171.11	23
9	1023.57	1005.56	1006.54	512.29	G	2194.16	2176.15	2177.13	1097.58	22
10	1122.64	1104.62	1105.61	561.82	V	2137.14	2119.13	2120.11	1069.07	21
11	1179.66	1161.65	1162.63	590.33	G	2038.07	2020.06	2021.04	1019.54	20
12	1278.72	1260.71	1261.70	639.86	V	1981.05	1963.04	1964.02	991.03	19
13	1349.77	1331.75	1332.74	675.38	A	1881.98	1863.97	1864.95	941.49	18
14	1477.82	1459.81	1460.79	739.41	Q	1810.95	1792.93	1793.93	905.97	17
15	1548.86	1530.83	1531.84	774.93	A	1682.88	1664.88	1665.86	841.94	16
16	1645.91	1627.90	1628.88	823.46	P	1611.85	1593.84	1594.82	806.42	15
17	1773.97	1755.96	1756.94	887.48	Q	1514.80	1496.79	1497.77	757.90	14
18	1873.04	1855.03	1856.01	937.02	V	1386.74	1368.72	1369.71	693.87	13
19	1986.12	1968.11	1969.09	993.56	L	1287.67	1269.66	1270.66	644.33	12
20	2073.15	2055.14	2056.13	1037.08	S	1174.59	1156.57	1157.56	587.79	11
21	2170.21	2152.20	2153.18	1085.60	P	1087.55	1069.55	1070.54	544.28	10
22	2271.25	2253.24	2254.23	1136.13	T	990.50	972.49	973.47	495.75	9
23	2418.32	2400.31	2401.30	1209.66	F	889.45	871.44	872.43	445.23	8
24	2475.34	2457.33	2458.32	1238.17	G	742.39	724.37	725.36	371.69	7
25	2546.38	2528.37	2529.35	1273.69	A	685.36	667.35	668.34	343.18	6
26	2674.44	2656.43	2657.41	1337.72	Q	614.33	596.32	597.30	307.66	5
27	2789.47	2771.46	2772.44	1395.23	D	486.27	468.26	469.24	243.63	4
28	2886.52	2868.51	2869.49	1443.76	P	371.24	353.23	354.21	186.12	3
29	2985.59	2967.58	2968.56	1493.29	V	274.19	256.18	257.16	137.59	2
30					R	175.12	157.11	158.09	88.06	1

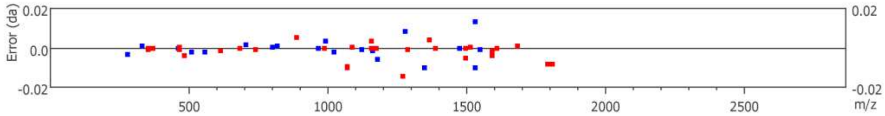

Brandsma C-A, *et al. Thorax* 2020; 75:180–183. doi: 10.1136/thoraxjnl-2019-213200

Peptide Sequence	Gene	Dataset	Sample	Score	ScanID
AAQAQGQSCEYSLMVGYQCGQVFR	FBLN1	1D50	COPD10	140.99	22140

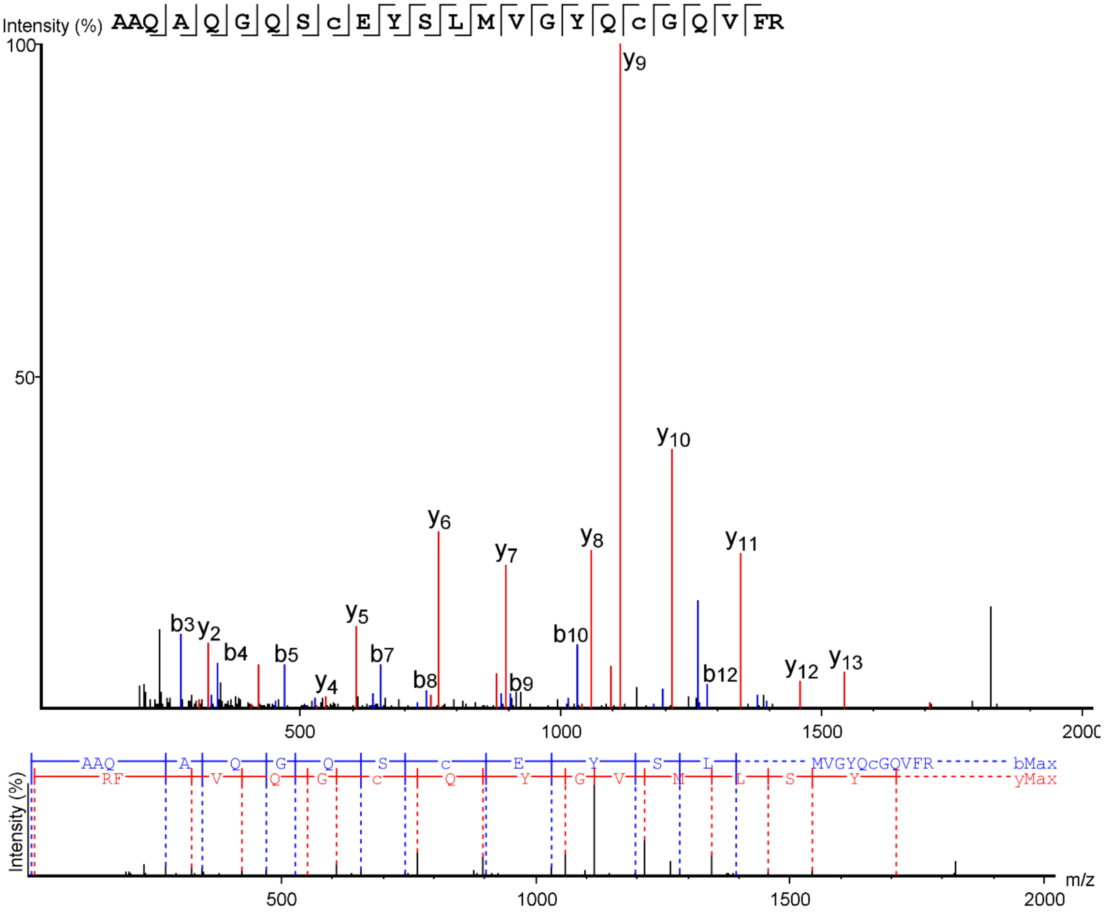

#	b	b-H2O	b-NH3	b (2+)	Seq	y	y-H2O	y-NH3	y (2+)	#
1	72.04	54.03	55.02	36.52	A					24
2	143.08	125.07	126.06	72.04	A	2667.17	2649.16	2650.15	1334.09	23
3	271.14	253.13	254.11	136.07	Q	2596.14	2578.13	2579.11	1298.57	22
4	342.18	324.17	325.15	171.59	A	2468.08	2450.07	2451.05	1234.54	21
5	470.24	452.23	453.21	235.62	Q	2397.04	2379.03	2380.01	1199.02	20
6	527.26	509.25	510.23	264.13	G	2268.98	2250.97	2251.96	1134.99	19
7	655.31	637.30	638.29	328.16	Q	2211.96	2193.95	2194.93	1106.48	18
8	742.35	724.34	725.33	371.67	S	2083.90	2065.89	2066.88	1042.45	17
9	902.37	884.38	885.35	451.69	C(+57.02)	1996.87	1978.86	1979.84	998.94	16
10	1031.41	1013.41	1014.39	516.21	E	1836.84	1818.83	1819.81	918.92	15
11	1194.48	1176.47	1177.45	597.74	Y	1707.80	1689.79	1690.77	854.40	14
12	1281.51	1263.51	1264.51	641.26	S	1544.74	1526.72	1527.71	772.87	13
13	1394.60	1376.59	1377.58	697.80	L	1457.70	1439.69	1440.68	729.35	12
14	1525.64	1507.63	1508.61	763.32	M	1344.62	1326.61	1327.59	672.81	11
15	1624.71	1606.70	1607.68	812.85	V	1213.58	1195.57	1196.55	607.29	10
16	1681.73	1663.72	1664.70	841.37	G	1114.51	1096.50	1097.48	557.75	9
17	1844.79	1826.78	1827.77	922.90	Y	1057.49	1039.48	1040.46	529.24	8
18	1972.85	1954.84	1955.83	986.93	Q	894.42	876.41	877.40	447.71	7
19	2132.88	2114.87	2115.86	1066.94	C(+57.02)	766.37	748.36	749.34	383.68	6
20	2189.91	2171.90	2172.88	1095.45	G	606.34	588.33	589.31	303.67	5
21	2317.96	2299.95	2300.94	1159.48	Q	549.32	531.30	532.29	275.16	4
22	2417.03	2399.02	2400.01	1209.02	V	421.26	403.25	404.23	211.13	3
23	2564.10	2546.09	2547.07	1282.55	F	322.19	304.18	305.16	161.59	2
24					R	175.12	157.11	158.09	88.06	1

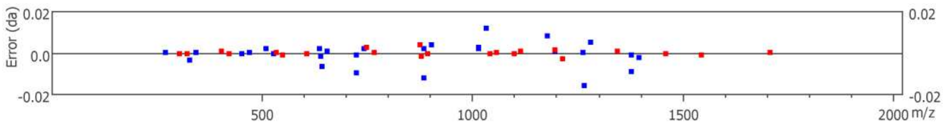

Peptide Sequence	Gene	Dataset	Sample	Score	ScanID
LLIYDASTR	P0DOX7	1D25	Control8	77.22	12317

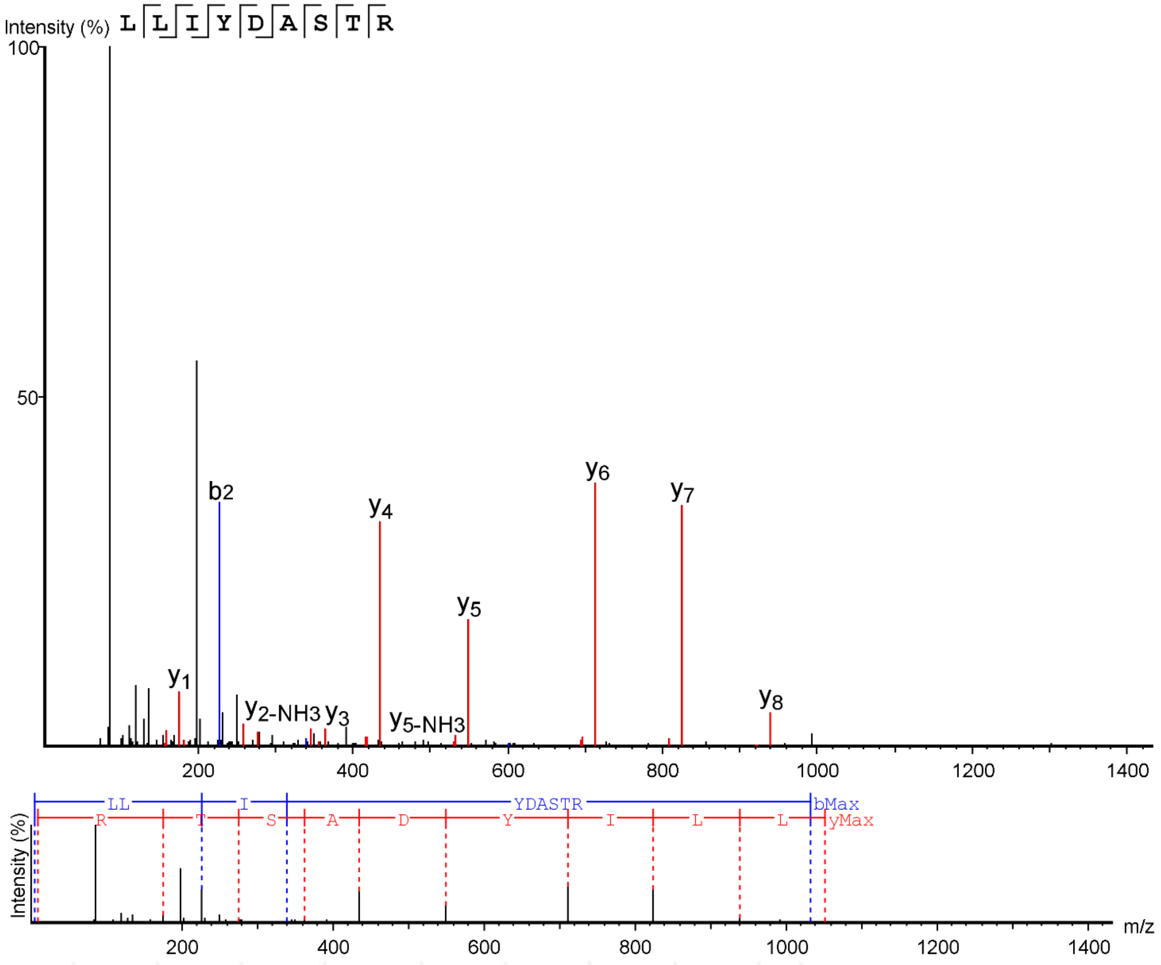

#	b	b-H2O	b-NH3	b (2+)	Seq	y	y-H2O	y-NH3	y (2+)	#
1	114.09	96.08	97.06	57.55	L					9
2	227.18	209.17	210.15	114.09	L	938.49	920.49	921.47	469.75	8
3	340.26	322.25	323.23	170.63	I	825.41	807.40	808.39	413.21	7
4	503.32	485.31	486.30	252.16	Y	712.33	694.31	695.30	356.67	6
5	618.35	600.34	601.33	309.68	D	549.26	531.25	532.24	275.13	5
6	689.39	671.38	672.36	345.19	A	434.24	416.23	417.21	217.62	4
7	776.42	758.41	759.39	388.71	S	363.20	345.19	346.17	182.08	3
8	877.47	859.46	860.44	439.23	T	276.17	258.16	259.14	138.58	2
9					R	175.12	157.11	158.09	88.06	1

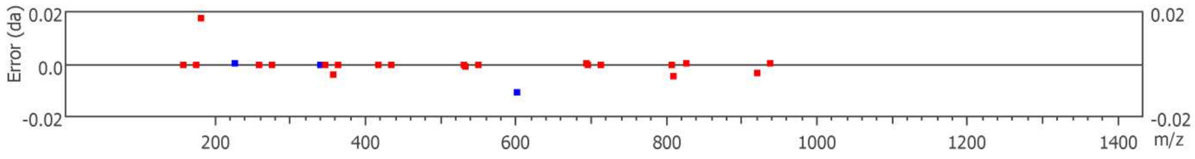

Peptide Sequence	Gene	Dataset	Sample	Score	ScanID
LLIYDTSNR	IGKV3-11	2D25	Control8	72.05	9811

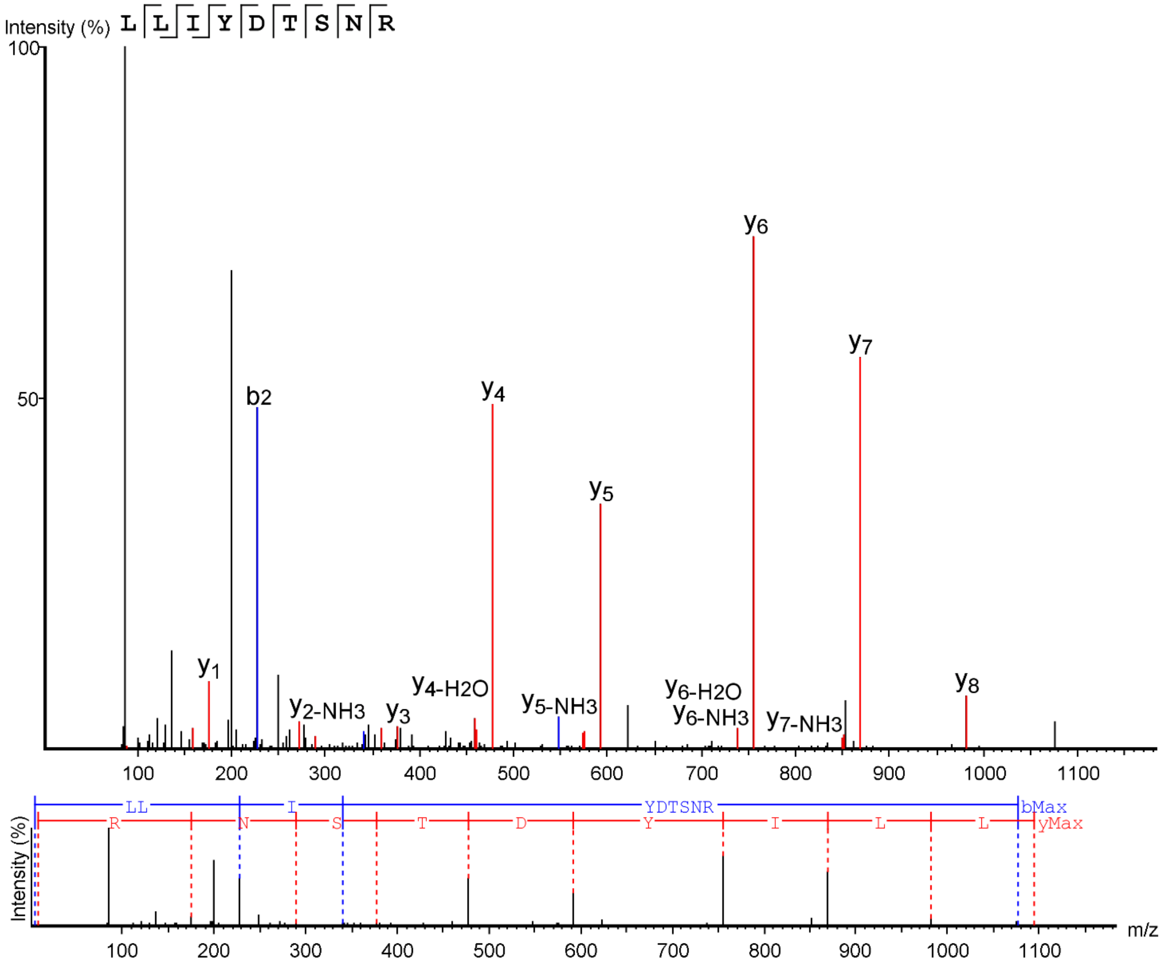

#	b	b-H2O	b-NH3	b (2+)	Seq	y	y-H2O	y-NH3	y (2+)	#
1	114.09	96.08	97.06	57.55	L					9
2	227.18	209.17	210.15	114.09	L	981.50	963.49	964.47	491.25	8
3	340.26	322.25	323.23	170.63	I	868.42	850.40	851.40	434.71	7
4	503.32	485.31	486.30	252.16	Y	755.33	737.32	738.30	378.17	6
5	618.35	600.34	601.32	309.68	D	592.27	574.26	575.24	296.63	5
6	719.40	701.39	702.37	360.20	T	477.24	459.23	460.21	239.12	4
7	806.43	788.42	789.40	403.71	S	376.19	358.18	359.17	188.60	3
8	920.47	902.46	903.45	460.74	N	289.16	271.15	272.14	145.08	2
9					R	175.12	157.11	158.09	88.04	1

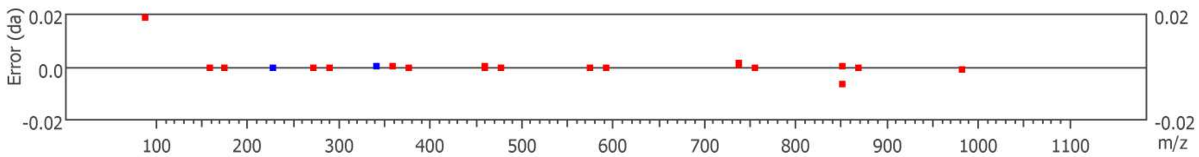

Brandsma C-A, *et al. Thorax* 2020; 75:180–183. doi: 10.1136/thoraxjnl-2019-213200

Peptide Sequence	Gene	Dataset	Sample	Score	ScanID
TPEVTCVVVDVSHEDPEVQFNWYVDGMEVHNAK	IGHG2	1D25	Control4	147.77	24113

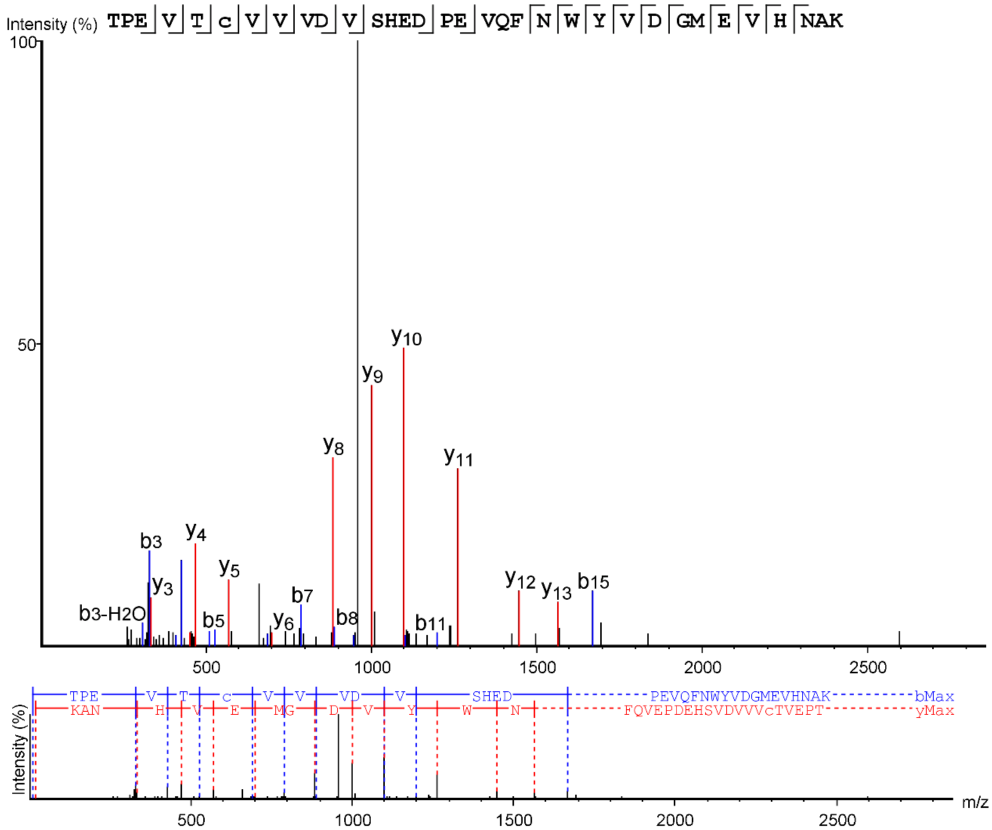

#	b	b-H2O	b-NH3	b (2+)	Seq	y	y-H2O	y-NH3	y (2+)	#
1	102.06	84.04	85.03	51.53	T					33
2	199.11	181.10	182.08	100.05	P	3728.70	3710.69	3711.67	1864.85	32
3	328.15	310.14	311.12	164.58	E	3631.65	3613.64	3614.62	1816.32	31
4	427.22	409.21	410.19	214.11	V	3502.60	3484.59	3485.58	1751.80	30
5	528.27	510.26	511.24	264.63	T	3403.54	3385.53	3386.51	1702.27	29
6	688.29	670.29	671.27	344.65	C(+57.02)	3302.49	3284.48	3285.46	1651.74	28
7	787.37	769.36	770.34	394.18	V	3142.46	3124.45	3125.43	1571.73	27
8	886.42	868.42	869.41	443.72	V	3043.39	3025.38	3026.36	1522.19	26
9	985.50	967.49	968.48	493.25	V	2944.32	2926.31	2927.29	1472.66	25
10	1100.53	1082.52	1083.50	550.76	D	2845.25	2827.24	2828.23	1423.13	24
11	1199.61	1181.59	1182.57	600.30	V	2730.23	2712.21	2713.20	1365.61	23
12	1286.63	1268.62	1269.60	643.82	S	2631.16	2613.15	2614.13	1316.08	22
13	1423.69	1405.68	1406.66	712.34	H	2544.12	2526.11	2527.10	1272.56	21
14	1552.73	1534.72	1535.70	776.87	E	2407.07	2389.06	2390.04	1204.03	20
15	1667.75	1649.75	1650.73	834.38	D	2278.02	2260.01	2261.00	1139.51	19
16	1764.81	1746.80	1747.78	882.91	P	2163.00	2144.99	2145.97	1082.00	18
17	1893.85	1875.84	1876.83	947.43	E	2065.94	2047.93	2048.92	1033.47	17
18	1992.92	1974.91	1975.90	996.96	V	1936.90	1918.89	1919.87	968.95	16
19	2120.98	2102.97	2103.95	1060.99	Q	1837.83	1819.82	1820.81	919.42	15
20	2268.05	2250.04	2251.02	1134.52	F	1709.77	1691.76	1692.75	855.39	14
21	2382.09	2364.08	2365.07	1191.55	N	1562.71	1544.70	1545.68	781.85	13
22	2568.17	2550.16	2551.14	1284.59	W	1448.66	1430.65	1431.64	724.83	12
23	2731.23	2713.22	2714.21	1366.12	Y	1262.58	1244.57	1245.56	631.79	11
24	2830.30	2812.29	2813.28	1415.65	V	1099.52	1081.51	1082.49	550.26	10
25	2945.33	2927.32	2928.30	1473.17	D	1000.45	982.44	983.42	500.73	9
26	3002.35	2984.34	2985.32	1501.68	G	885.42	867.41	868.40	443.21	8
27	3133.39	3115.38	3116.37	1567.20	M	828.40	810.39	811.38	414.70	7
28	3262.43	3244.42	3245.41	1631.72	E	697.36	679.35	680.34	349.18	6
29	3361.50	3343.49	3344.48	1681.25	V	568.32	550.31	551.29	284.66	5
30	3498.56	3480.55	3481.54	1749.78	H	469.25	451.24	452.22	235.13	4
31	3612.61	3594.59	3595.58	1806.80	N	332.19	314.18	315.17	166.60	3
32	3683.64	3665.63	3666.62	1842.32	A	218.15	200.14	201.12	109.57	2
33					K	147.11	129.10	130.09	74.06	1

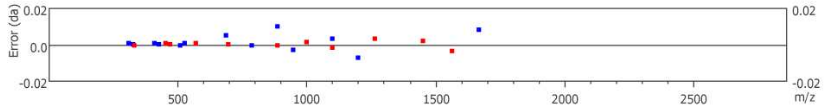

Peptide Sequence	Gene	Dataset	Sample	Score	ScanID
DAMWIGFLTR	ASAH1	1D25	Control10	95.37	26060

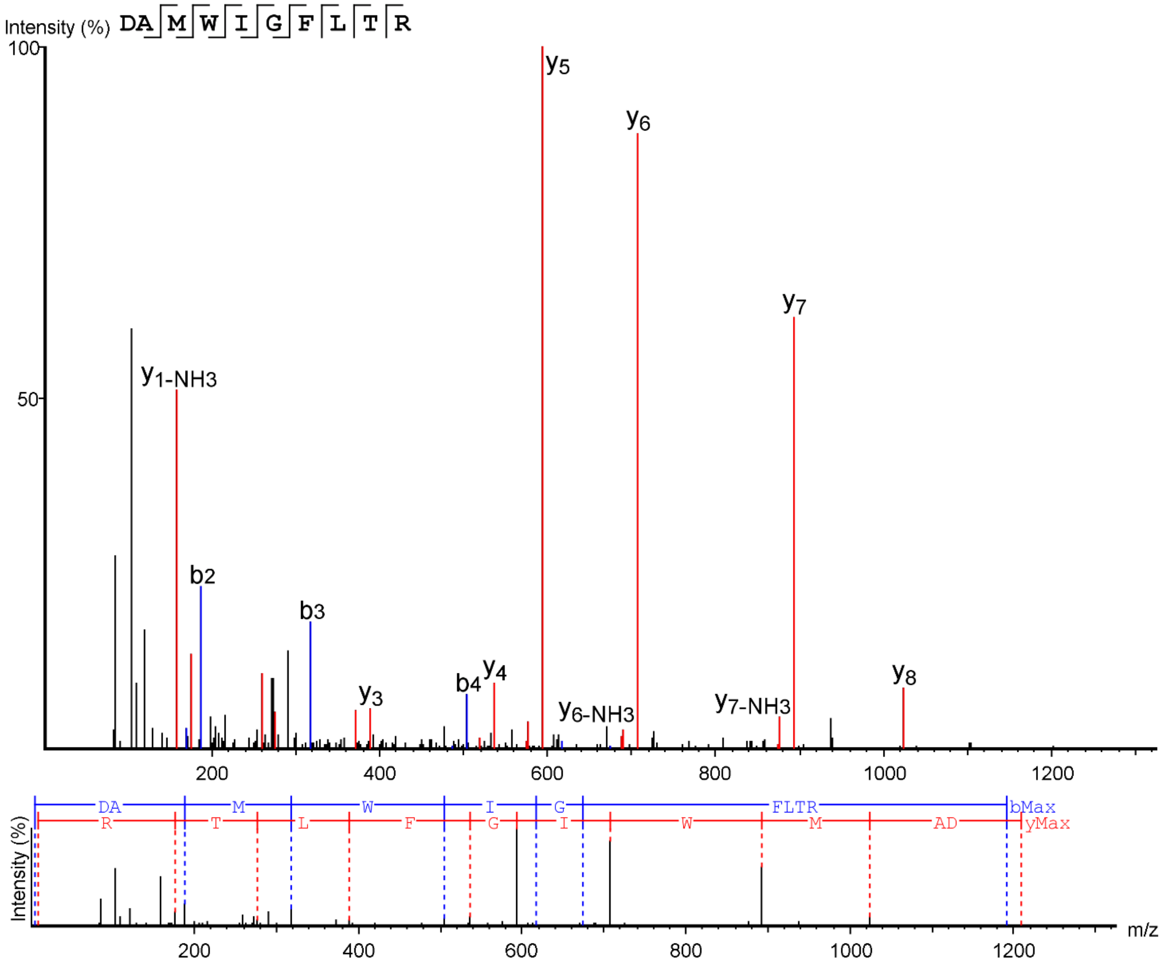

#	b	b-H2O	b-NH3	b (2+)	Seq	y	y-H2O	y-NH3	y (2+)	#
1	116.03	98.02	99.01	58.52	D					10
2	187.07	169.06	170.06	94.04	A	1094.58	1076.57	1077.55	547.79	9
3	318.11	300.10	301.09	159.56	M	1023.54	1005.53	1006.52	512.27	8
4	504.19	486.18	487.16	252.60	W	892.50	874.49	875.48	446.75	7
5	617.27	599.27	600.25	309.14	I	706.42	688.41	689.40	353.71	6
6	674.30	656.29	657.27	337.65	G	593.34	575.33	576.31	297.17	5
7	821.37	803.36	804.34	411.18	F	536.32	518.31	519.29	268.66	4
8	934.45	916.44	917.42	467.72	L	389.25	371.24	372.22	195.13	3
9	1035.50	1017.49	1018.47	518.25	T	276.17	258.16	259.14	138.58	2
10					R	175.12	157.11	158.09	88.06	1

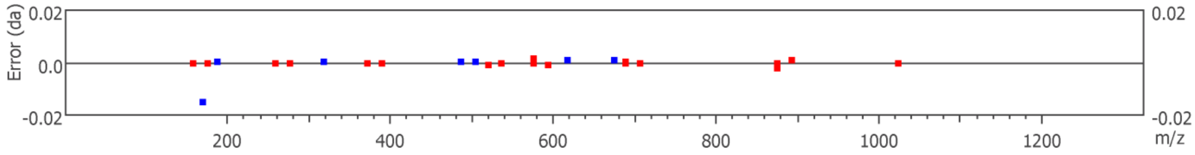

Brandsma C-A, *et al. Thorax* 2020; 75:180–183. doi: 10.1136/thoraxjnl-2019-213200

Peptide Sequence	Gene	Dataset	Sample	Score	ScanID
LLISWASTR	IGKV4-1	1D50	Control2	63.08	20195

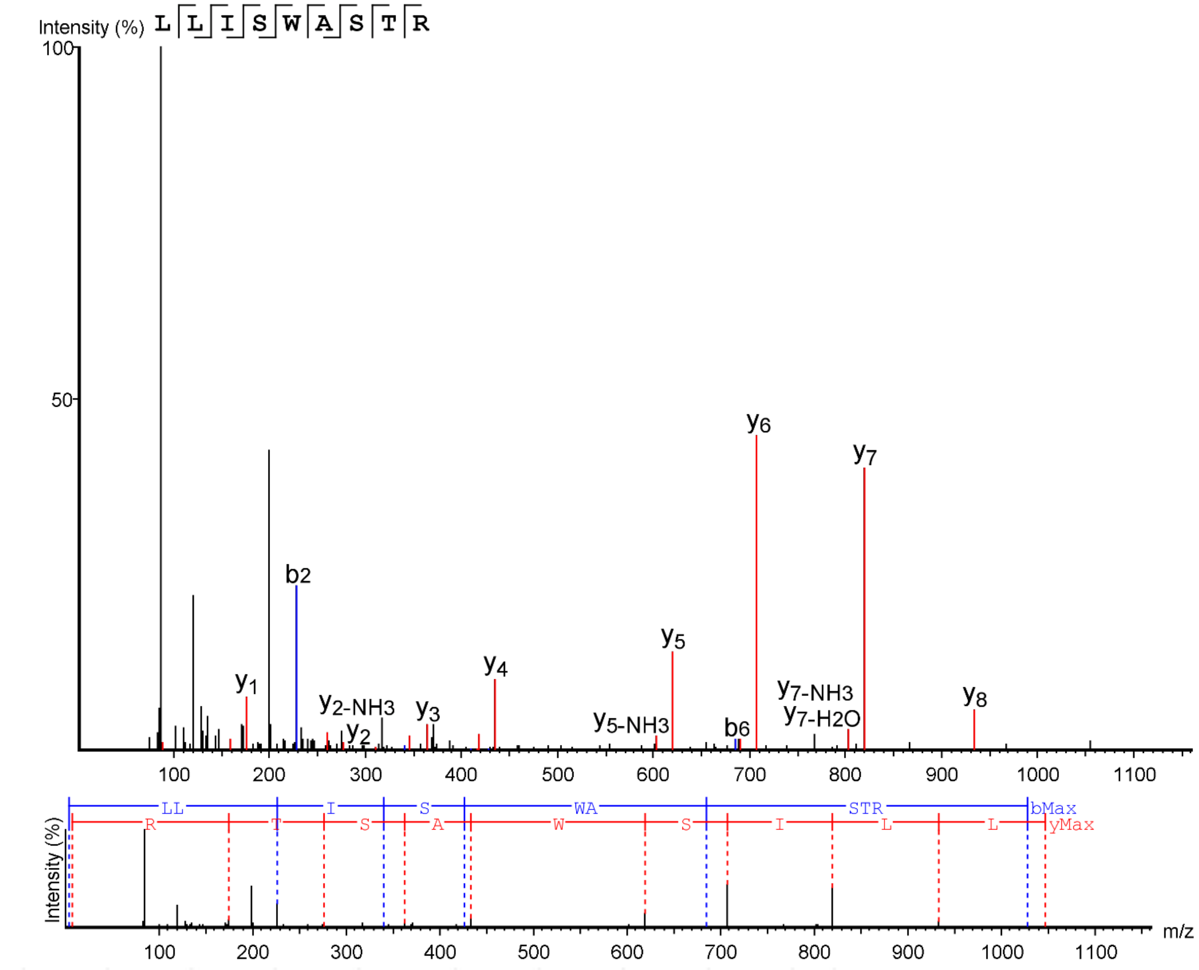

#	b	b-H2O	b-NH3	b (2+)	Seq	y	y-H2O	y-NH3	y (2+)	#
1	114.09	96.08	97.06	57.55	L					9
2	227.18	209.17	210.15	114.09	L	933.51	915.50	916.49	467.26	8
3	340.26	322.25	323.23	170.63	I	820.43	802.42	803.42	410.72	7
4	427.29	409.28	410.27	214.15	S	707.35	689.34	690.33	354.17	6
5	613.37	595.36	596.34	307.19	W	620.31	602.30	603.29	310.66	5
6	684.40	666.40	667.38	342.70	A	434.24	416.23	417.21	217.62	4
7	771.44	753.43	754.41	386.22	S	363.20	345.19	346.17	182.10	3
8	872.49	854.48	855.46	436.74	T	276.17	258.16	259.14	138.58	2
9					R	175.12	157.11	158.09	88.04	1

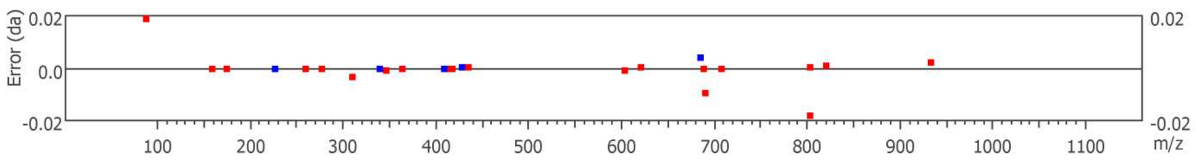

Peptide Sequence	Gene	Dataset	Sample	Score	ScanID
LLIYDNDKRPSGIPDR	IGLV1-51	2D25	Control10	105.39	9338

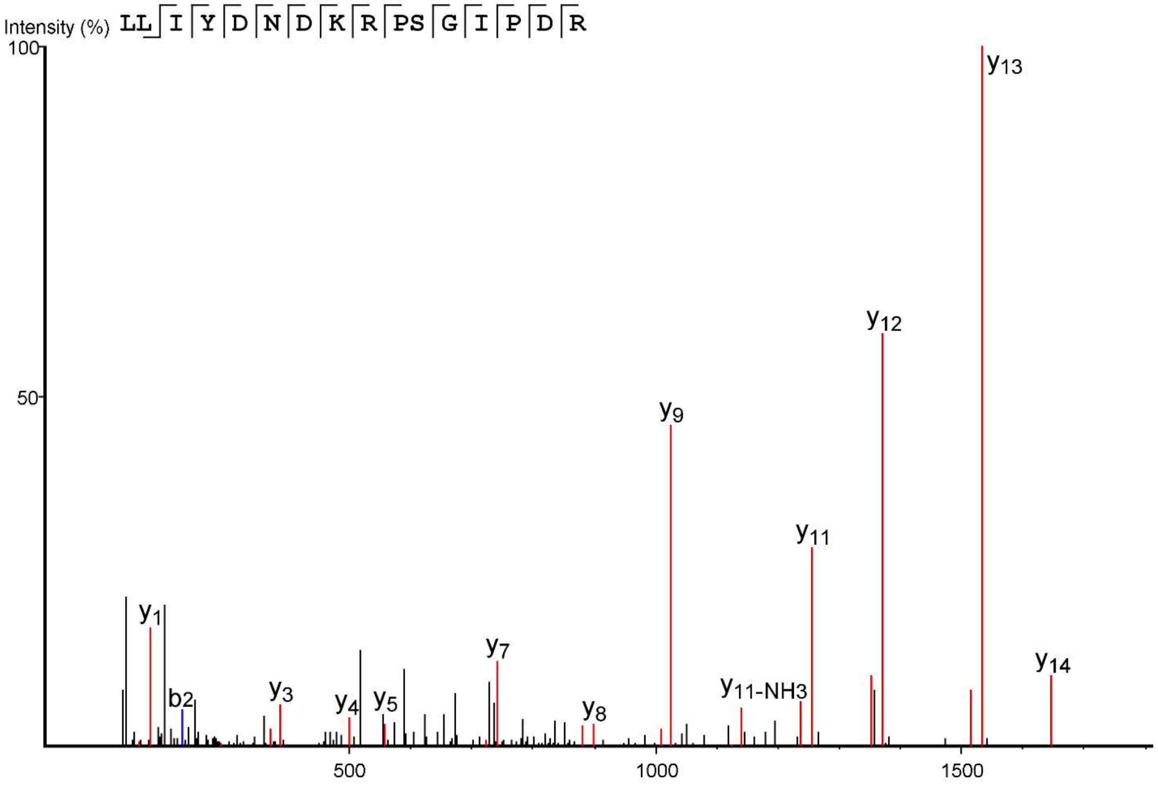

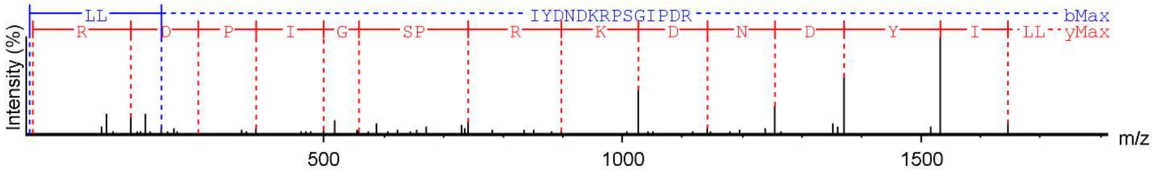

#	b	b-H2O	b-NH3	b (2+)	Seq	y	y-H2O	y-NH3	y (2+)	#
1	114.09	96.08	97.06	57.55	L					16
2	227.18	209.17	210.15	114.09	L	1758.91	1740.90	1741.89	879.96	15
3	340.26	322.25	323.23	170.63	I	1645.83	1627.82	1628.80	823.41	14
4	503.32	485.31	486.30	252.16	Y	1532.74	1514.72	1515.72	766.87	13
5	618.35	600.34	601.32	309.68	D	1369.68	1351.67	1352.66	685.34	12
6	732.39	714.38	715.37	366.70	N	1254.65	1236.64	1237.63	627.83	11
7	847.42	829.41	830.39	424.21	D	1140.61	1122.60	1123.59	570.81	10
8	975.52	957.50	958.49	488.26	K	1025.58	1007.56	1008.56	513.29	9
9	1131.62	1113.61	1114.59	566.31	R	897.49	879.48	880.47	449.25	8
10	1228.67	1210.66	1211.64	614.83	P	741.39	723.38	724.36	371.19	7
11	1315.70	1297.69	1298.67	658.35	S	644.34	626.33	627.31	322.67	6
12	1372.72	1354.71	1355.70	686.86	G	557.30	539.29	540.28	279.15	5
13	1485.81	1467.80	1468.78	743.40	I	500.28	482.27	483.26	250.64	4
14	1582.86	1564.85	1565.83	791.93	P	387.20	369.19	370.17	194.10	3
15	1697.89	1679.88	1680.86	849.44	D	290.15	272.14	273.12	145.57	2
16					R	175.12	157.11	158.09	88.06	1

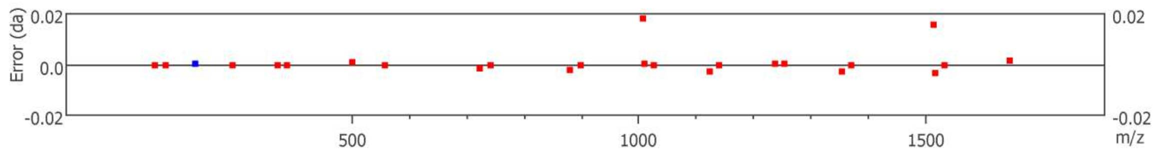

Brandsma C-A, *et al. Thorax* 2020; 75:180–183. doi: 10.1136/thoraxjnl-2019-213200

Peptide Sequence	Gene	Dataset	Sample	Score	ScanID
TYYADSVK	IGHV3-9	1D25	COPD10	55.49	4978

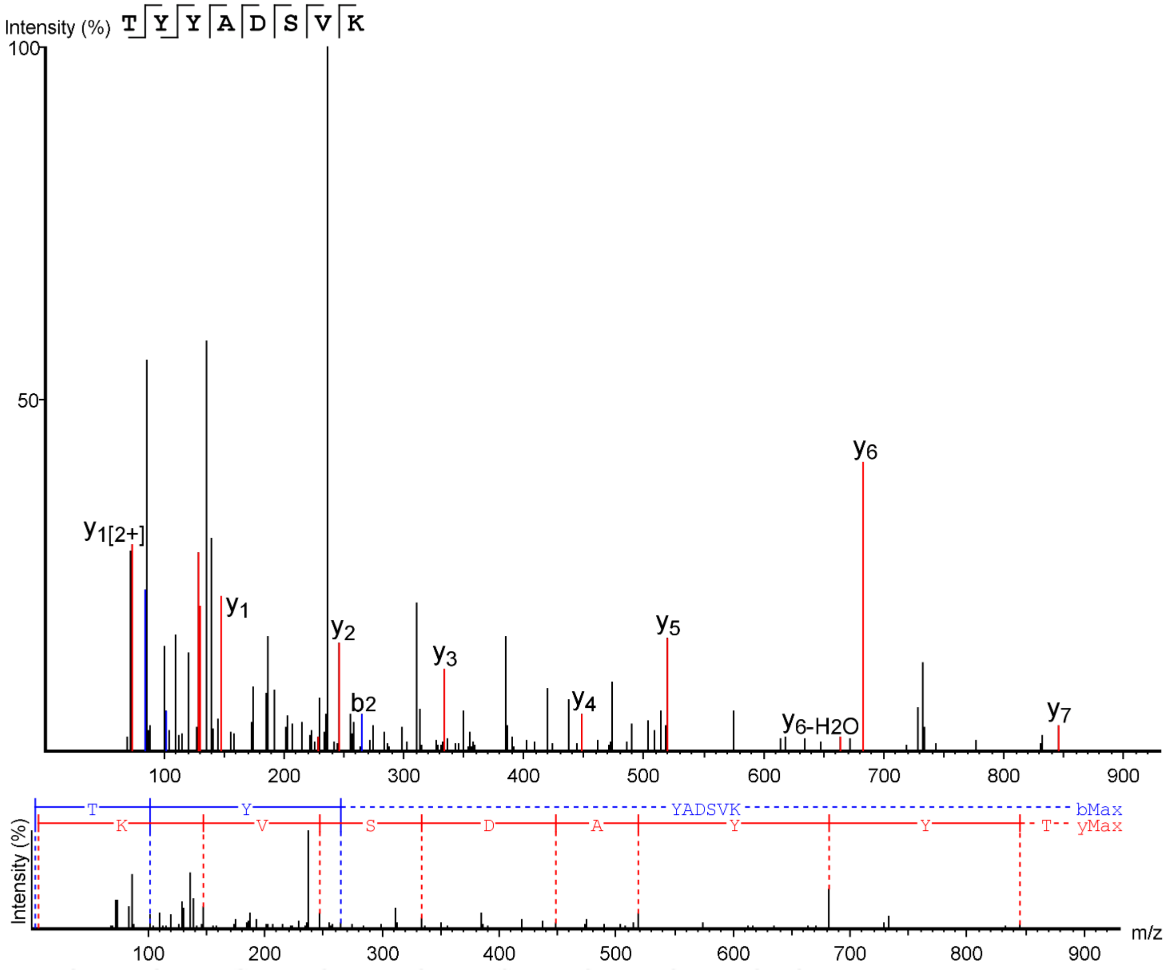

#	b	b-H2O	b-NH3	b (2+)	Seq	y	y-H2O	y-NH3	y (2+)	#
1	102.06	84.04	85.03	51.53	T					8
2	265.12	247.11	248.09	133.06	Y	845.40	827.39	828.38	423.20	7
3	428.18	410.17	411.16	214.59	Y	682.34	664.33	665.31	341.67	6
4	499.22	481.21	482.19	250.11	A	519.28	501.27	502.25	260.14	5
5	614.25	596.24	597.22	307.62	D	448.24	430.23	431.21	224.62	4
6	701.28	683.27	684.25	351.14	S	333.21	315.20	316.19	167.11	3
7	800.35	782.34	783.32	400.67	V	246.18	228.17	229.15	123.59	2
8					K	147.11	129.10	130.09	74.06	1

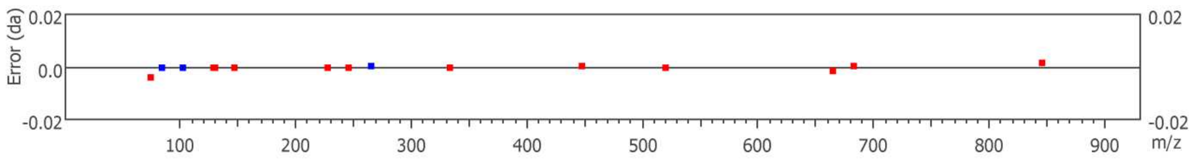

Brandsma C-A, *et al. Thorax* 2020; 75:180–183. doi: 10.1136/thoraxjnl-2019-213200

Peptide Sequence	Gene	Dataset	Sample	Score	ScanID
GTPVTVSSASTK	P0DOX5	2D25	Control2	94.79	4387

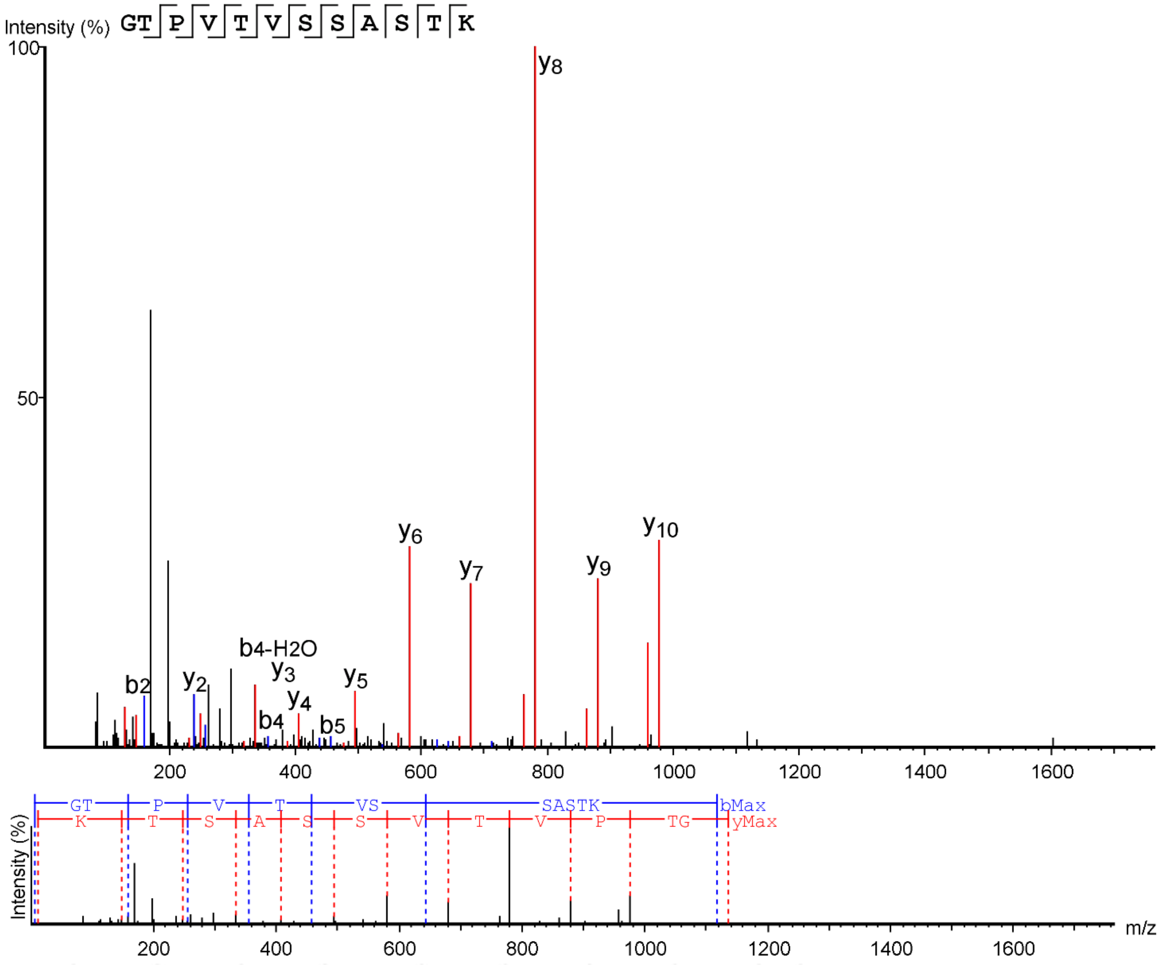

#	b	b-H2O	b-NH3	b (2+)	Seq	y	y-H2O	y-NH3	y (2+)	#
1	58.03	40.02	41.00	29.51	G					12
2	159.08	141.07	142.05	80.04	T	1077.58	1059.57	1060.55	539.29	11
3	256.13	238.12	239.10	128.56	P	976.53	958.52	959.50	488.77	10
4	355.20	337.19	338.17	178.10	V	879.48	861.47	862.45	440.24	9
5	456.24	438.23	439.22	228.62	T	780.41	762.40	763.38	390.70	8
6	555.31	537.30	538.29	278.16	V	679.36	661.35	662.34	340.18	7
7	642.35	624.33	625.32	321.67	S	580.29	562.28	563.27	290.65	6
8	729.38	711.37	712.35	365.19	S	493.26	475.25	476.23	247.13	5
9	800.42	782.40	783.39	400.71	A	406.23	388.22	389.20	203.61	4
10	887.45	869.44	870.42	444.22	S	335.19	317.18	318.17	168.10	3
11	988.50	970.48	971.47	494.75	T	248.16	230.15	231.13	124.58	2
12					K	147.11	129.10	130.09	74.06	1

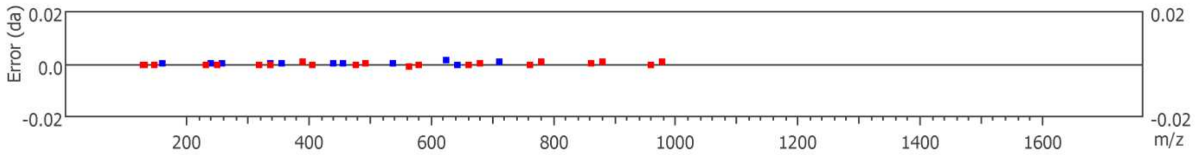

Peptide Sequence	Gene	Dataset	Sample	Score	ScanID
EQPGSPEWIQLDK	AIP	2D25	Control8	93.79	13674

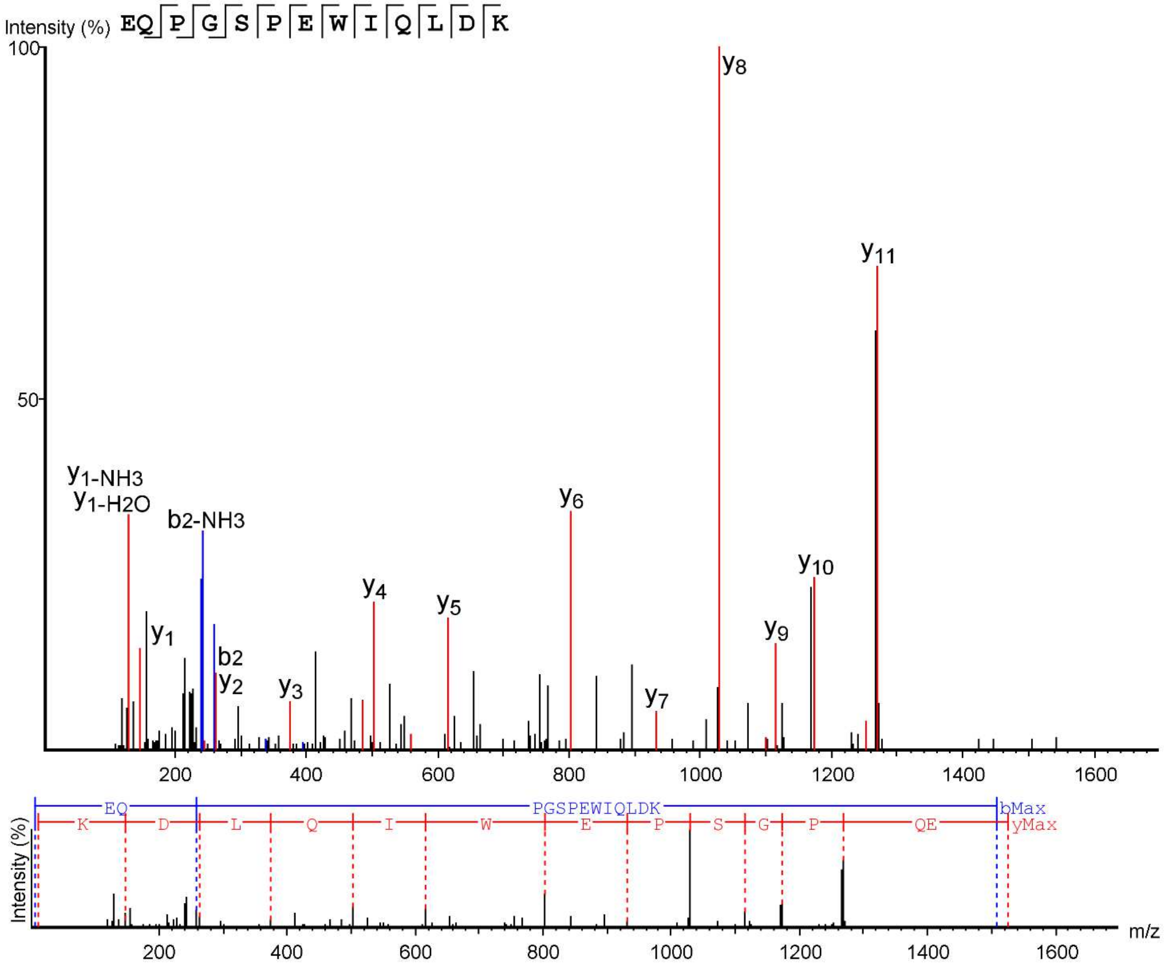

#	b	b-H2O	b-NH3	b (2+)	Seq	y	y-H2O	y-NH3	y (2+)	#
1	130.05	112.04	113.02	65.53	E					13
2	258.11	240.10	241.08	129.55	Q	1397.71	1379.70	1380.68	699.35	12
3	355.16	337.15	338.13	178.08	P	1269.65	1251.64	1252.62	635.32	11
4	412.18	394.17	395.16	206.59	G	1172.59	1154.58	1155.57	586.80	10
5	499.22	481.20	482.19	250.11	S	1115.57	1097.56	1098.56	558.29	9
6	596.27	578.26	579.24	298.63	P	1028.54	1010.53	1011.51	514.77	8
7	725.31	707.30	708.28	363.16	E	931.49	913.48	914.46	466.24	7
8	911.39	893.38	894.36	456.19	W	802.45	784.44	785.42	401.72	6
9	1024.47	1006.46	1007.45	512.74	I	616.37	598.36	599.34	308.68	5
10	1152.53	1134.52	1135.51	576.77	Q	503.28	485.27	486.25	252.14	4
11	1265.62	1247.61	1248.59	633.31	L	375.22	357.21	358.20	188.11	3
12	1380.64	1362.63	1363.62	690.82	D	262.14	244.13	245.11	131.57	2
13					K	147.11	129.10	130.09	74.06	1

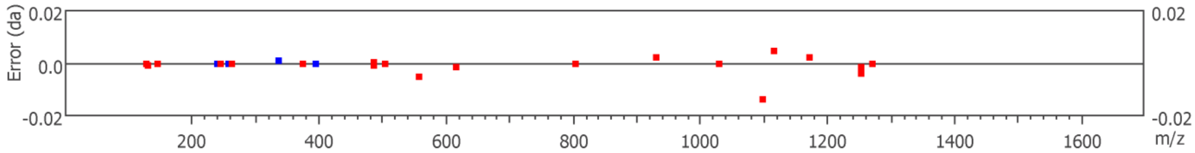

Peptide Sequence	Gene	Dataset	Sample	Score	ScanID
SMPIPIPK	C1orf116	2D25	Control6	53.15	10897

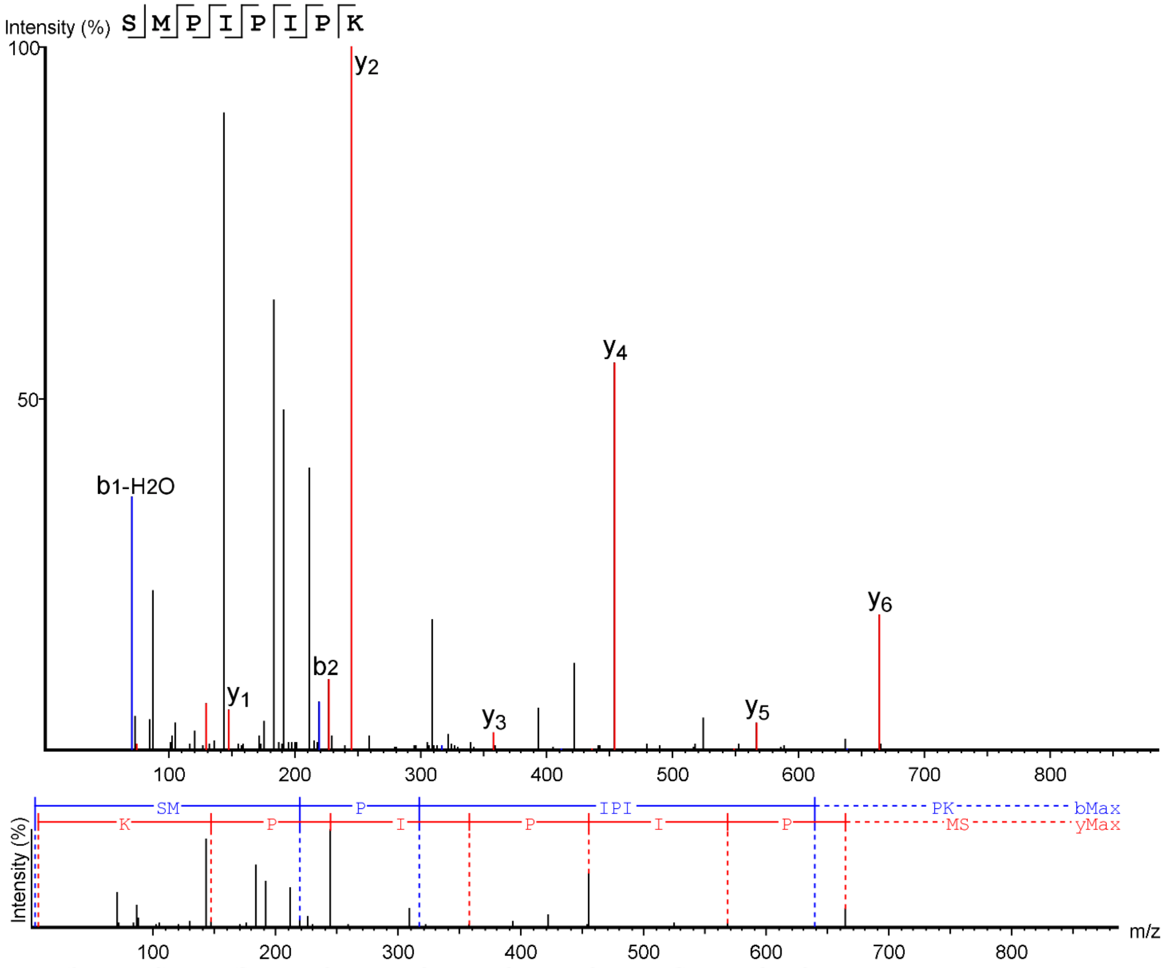

#	b	b-H2O	b-NH3	b (2+)	Seq	y	y-H2O	y-NH3	y (2+)	#
1	88.04	70.03	71.01	44.52	S					8
2	219.08	201.07	202.05	110.04	M	795.48	777.47	778.45	398.24	7
3	316.13	298.12	299.11	158.57	P	664.44	646.43	647.41	332.72	6
4	429.22	411.19	412.19	215.11	I	567.39	549.38	550.36	284.19	5
5	526.27	508.26	509.24	263.63	P	454.30	436.29	437.28	227.65	4
6	639.35	621.34	622.33	320.18	I	357.25	339.24	340.22	179.12	3
7	736.41	718.40	719.38	368.70	P	244.17	226.15	227.14	122.58	2
8					K	147.11	129.10	130.09	74.06	1

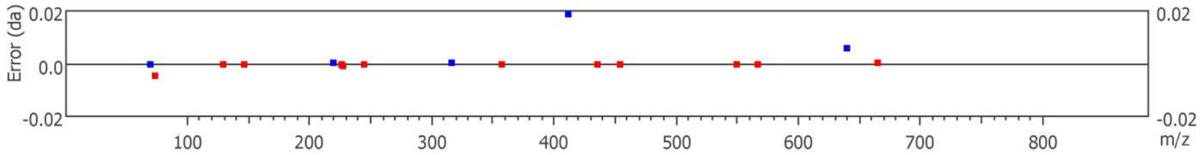

Peptide Sequence	Gene	Dataset	Sample	Score	ScanID
ASTLESGVPSR	IGKV1D-13	1D25	COPD9	75.55	6263

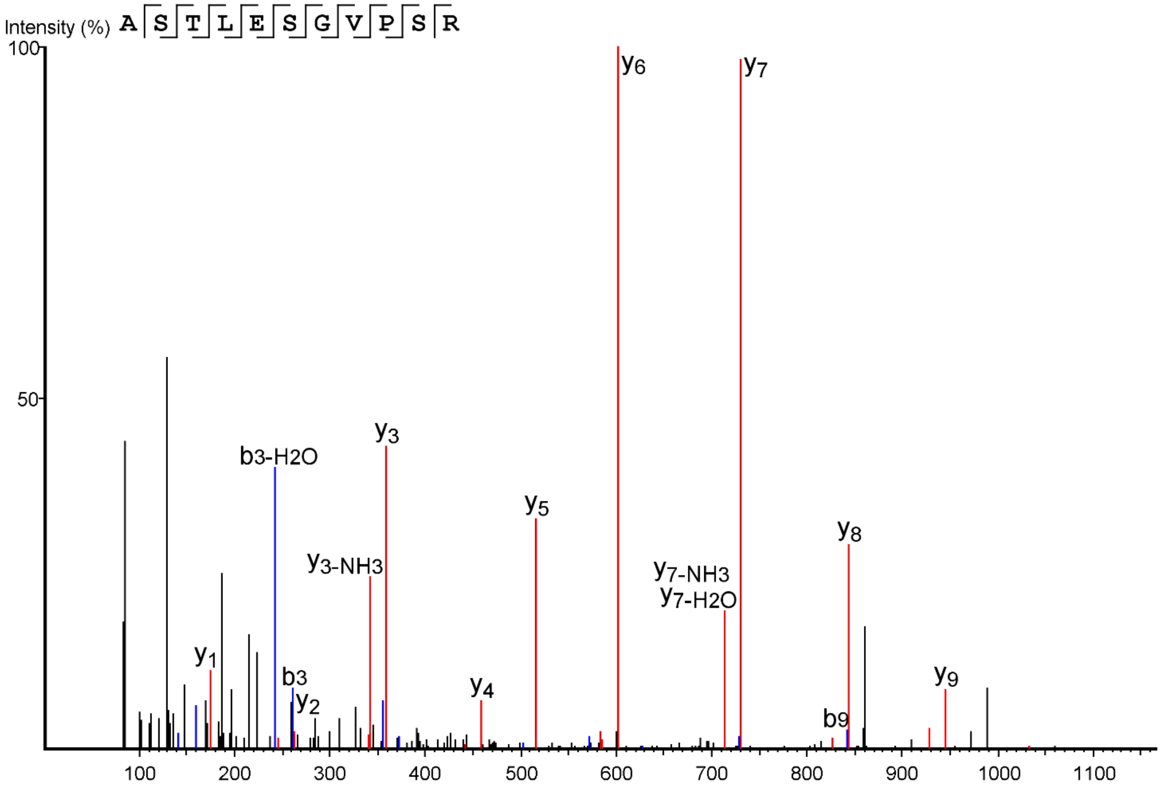

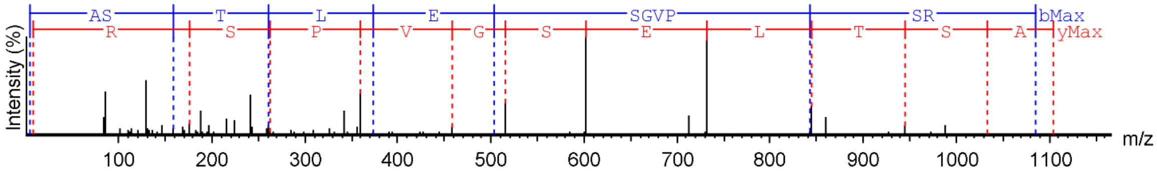

#	b	b-H2O	b-NH3	b (2+)	Seq	y	y-H2O	y-NH3	y (2+)	#
1	72.04	54.03	55.02	36.52	A					11
2	159.08	141.07	142.05	80.04	S	1032.52	1014.52	1015.51	516.77	10
3	260.12	242.11	243.10	130.56	T	945.50	927.48	928.47	473.25	9
4	373.21	355.20	356.18	187.10	L	844.45	826.44	827.43	422.73	8
5	502.25	484.24	485.22	251.63	E	731.37	713.36	714.36	366.18	7
6	589.28	571.27	572.26	295.14	S	602.33	584.31	585.30	301.66	6
7	646.30	628.29	629.28	323.65	G	515.29	497.28	498.27	258.15	5
8	745.37	727.36	728.36	373.17	V	458.27	440.26	441.24	229.64	4
9	842.44	824.42	825.40	421.71	P	359.20	341.19	342.18	180.10	3
10	929.46	911.45	912.43	465.23	S	262.15	244.14	245.12	131.58	2
11					R	175.12	157.11	158.09	88.06	1

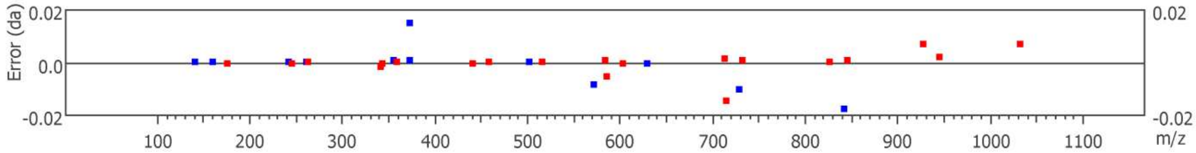

Peptide Sequence	Gene	Dataset	Sample	Score	ScanID
FIEDVK	OBSCN	1D50	COPD5	42.76	11427

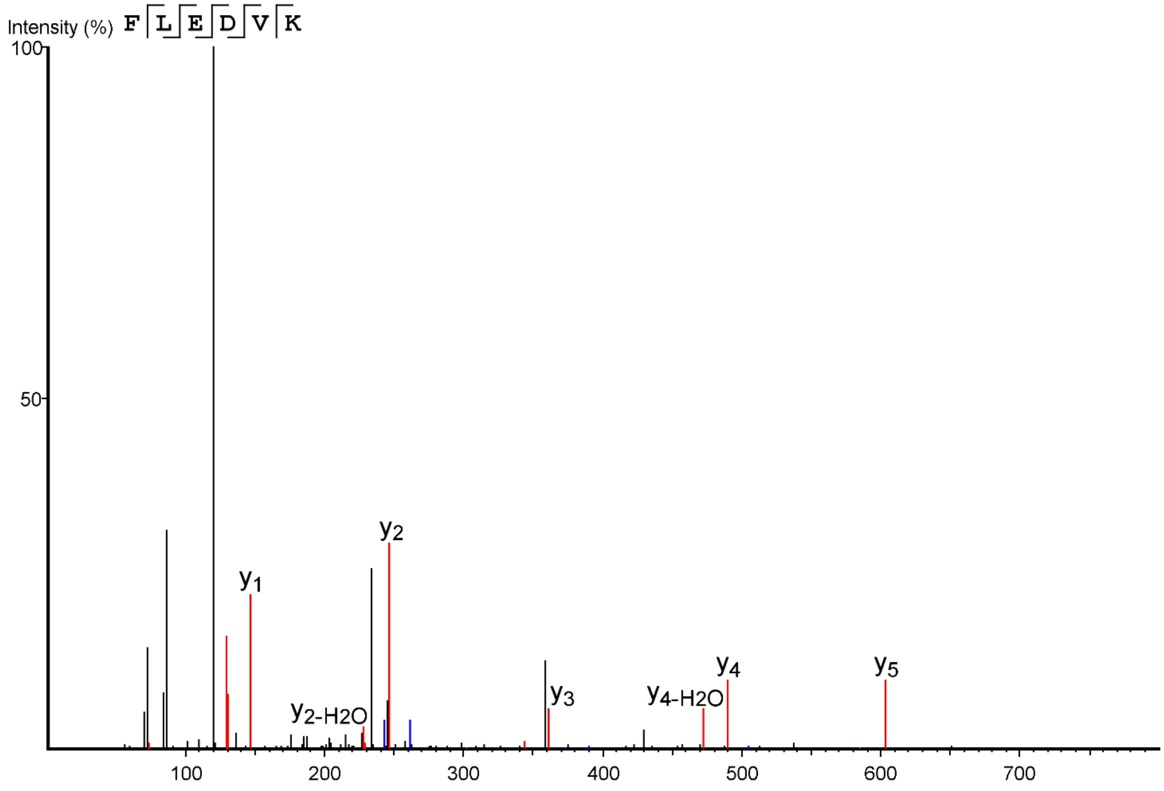

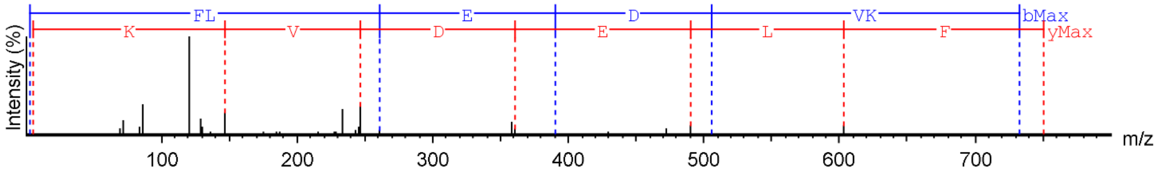

#	b	b-H2O	b-NH3	b (2+)	Seq	y	y-H2O	y-NH3	y (2+)	#
1	148.08	130.07	131.05	74.54	F					6
2	261.16	243.13	244.13	131.08	L	603.33	585.32	586.31	302.17	5
3	390.20	372.19	373.17	195.60	E	490.25	472.24	473.22	245.63	4
4	505.23	487.22	488.20	253.11	D	361.21	343.20	344.18	181.10	3
5	604.30	586.29	587.27	302.65	V	246.18	228.17	229.15	123.59	2
6					K	147.11	129.10	130.09	74.06	1

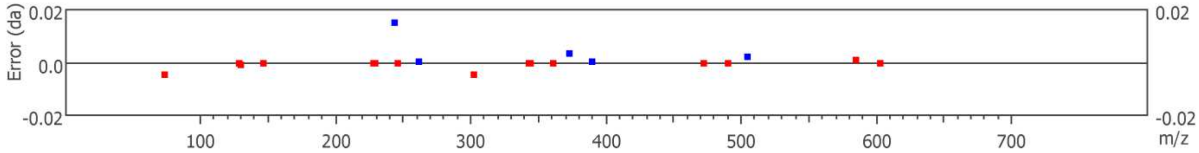

Brandsma C-A, *et al. Thorax* 2020; 75:180–183. doi: 10.1136/thoraxjnl-2019-213200

Peptide Sequence	Gene	Dataset	Sample	Score	ScanID
DLYTNTVLSGGTTMYPGIADR	Q25381	2D25	COPD3	78.17	17358

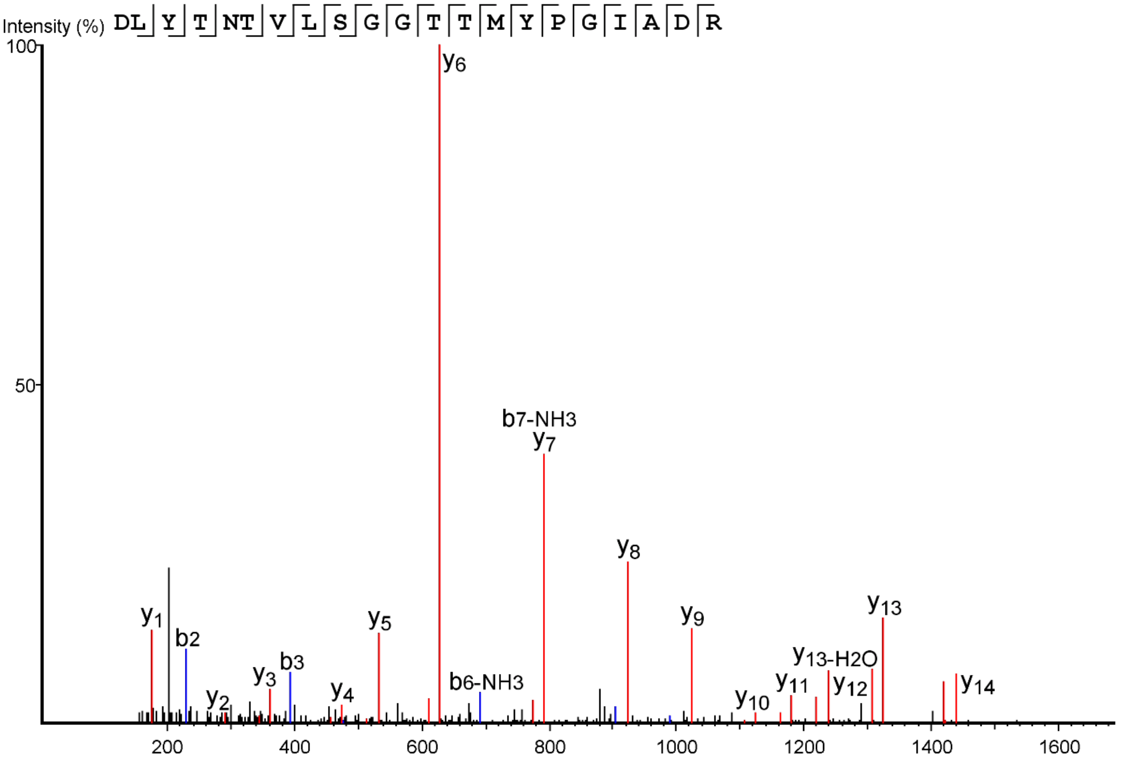

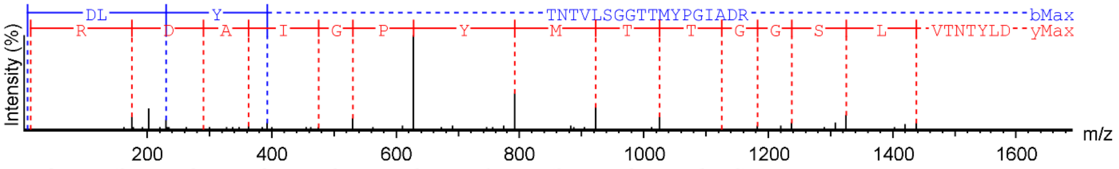

#	b	b-H2O	b-NH3	b (2+)	Seq	y	y-H2O	y-NH3	y (2+)	#
1	116.03	98.02	99.01	58.52	D					21
2	229.12	211.11	212.09	115.06	L	2130.05	2112.04	2113.03	1065.53	20
3	392.18	374.17	375.16	196.59	Y	2016.97	1998.96	1999.94	1008.98	19
4	493.23	475.22	476.22	247.11	T	1853.91	1835.90	1836.88	927.45	18
5	607.27	589.26	590.25	304.14	N	1752.86	1734.85	1735.83	876.93	17
6	708.32	690.31	691.30	354.66	T	1638.82	1620.81	1621.79	819.91	16
7	807.39	789.38	790.37	404.19	V	1537.77	1519.76	1520.74	769.38	15
8	920.47	902.46	903.46	460.74	L	1438.70	1420.69	1421.69	719.85	14
9	1007.50	989.49	990.49	504.25	S	1325.62	1307.61	1308.59	663.31	13
10	1064.53	1046.52	1047.50	532.76	G	1238.58	1220.57	1221.56	619.79	12
11	1121.55	1103.54	1104.52	561.27	G	1181.56	1163.55	1164.53	591.28	11
12	1222.60	1204.59	1205.57	611.80	T	1124.54	1106.54	1107.51	562.77	10
13	1323.64	1305.63	1306.62	662.32	T	1023.49	1005.48	1006.47	512.25	9
14	1454.68	1436.67	1437.66	727.84	M	922.44	904.43	905.42	461.72	8
15	1617.75	1599.74	1600.72	809.37	Y	791.41	773.40	774.39	396.20	7
16	1714.80	1696.79	1697.77	857.90	P	628.34	610.33	611.31	314.67	6
17	1771.82	1753.81	1754.79	886.41	G	531.29	513.29	514.26	266.14	5
18	1884.91	1866.89	1867.88	942.95	I	474.27	456.25	457.24	237.63	4
19	1955.94	1937.93	1938.92	978.47	A	361.18	343.19	344.16	181.09	3
20	2070.97	2052.96	2053.94	1035.98	D	290.15	272.14	273.12	145.57	2
21					R	175.12	157.11	158.09	88.06	1

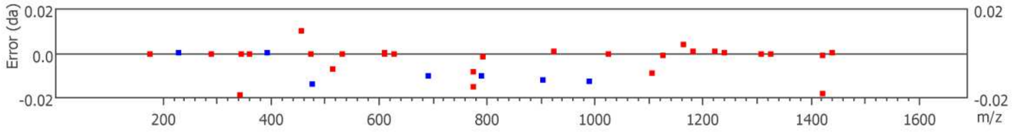

Peptide Sequence	Gene	Dataset	Sample	Score	ScanID
VDNDEDEHQLSLR	NPM1	2D25	COPD6	89.48	6619

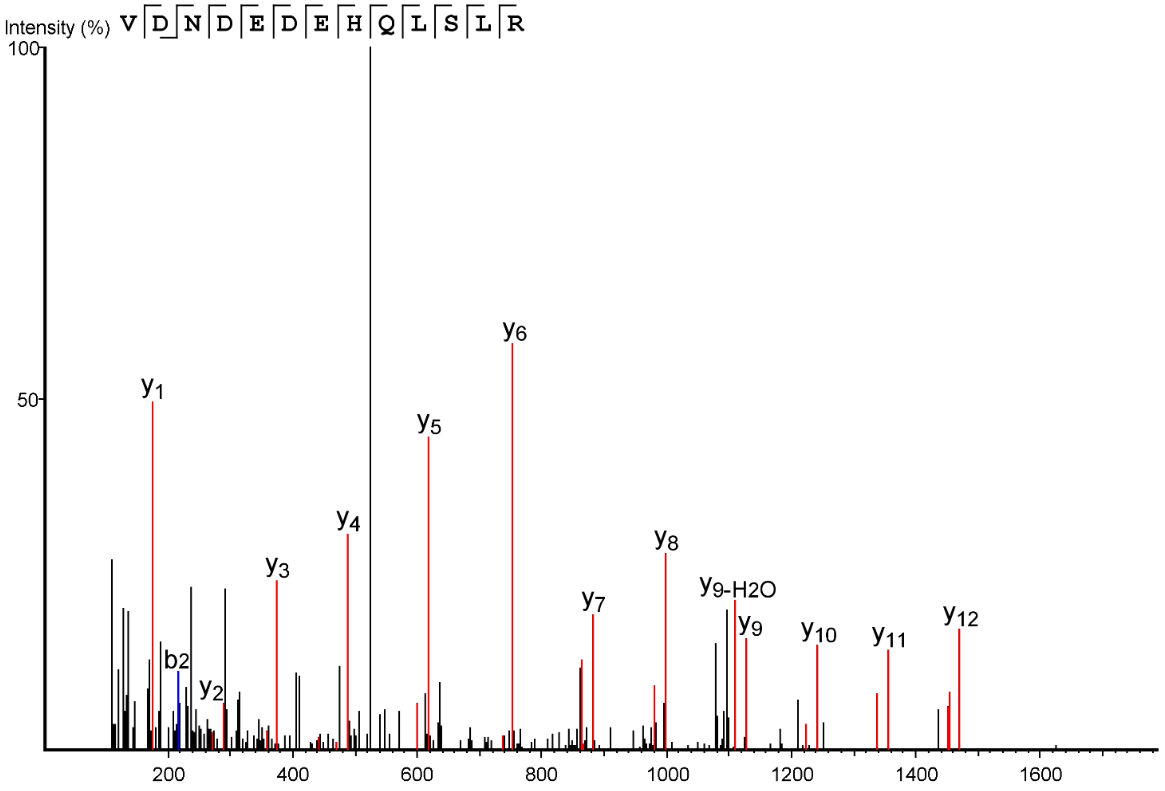

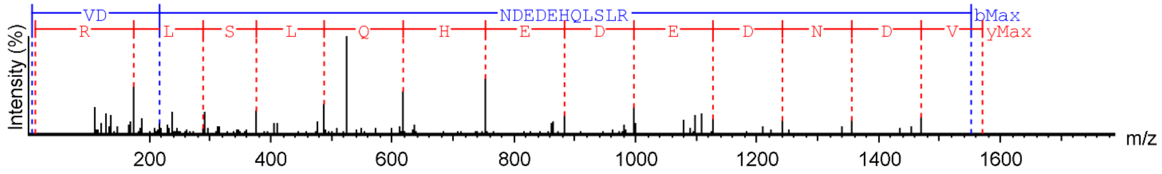

#	b	b-H2O	b-NH3	b (2+)	Seq	y	y-H2O	y-NH3	y (2+)	#
1	100.08	82.07	83.05	50.54	V					13
2	215.10	197.09	198.08	108.05	D	1470.65	1452.62	1453.63	735.82	12
3	329.15	311.14	312.12	165.07	N	1355.62	1337.61	1338.60	678.31	11
4	444.17	426.16	427.15	222.59	D	1241.58	1223.57	1224.55	621.29	10
5	573.22	555.21	556.19	287.11	E	1126.55	1108.54	1109.52	563.77	9
6	688.24	670.23	671.22	344.62	D	997.51	979.49	980.49	499.25	8
7	817.29	799.27	800.26	409.14	E	882.48	864.47	865.46	441.74	7
8	954.34	936.33	937.32	477.67	H	753.44	735.43	736.41	377.22	6
9	1082.40	1064.39	1065.38	541.70	Q	616.38	598.37	599.35	308.69	5
10	1195.49	1177.48	1178.46	598.24	L	488.32	470.31	471.29	244.66	4
11	1282.52	1264.51	1265.49	641.76	S	375.24	357.22	358.21	188.12	3
12	1395.60	1377.59	1378.58	698.30	L	288.20	270.19	271.18	144.60	2
13					R	175.12	157.11	158.09	88.06	1

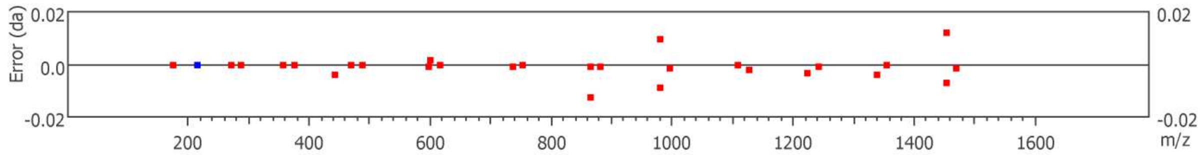

Peptide Sequence	Gene	Dataset	Sample	Score	ScanID
VGEAGLLSVNCSEAGPGALGLEAVSDSGTK	FLNB	1D25	COPD2	130.08	22577

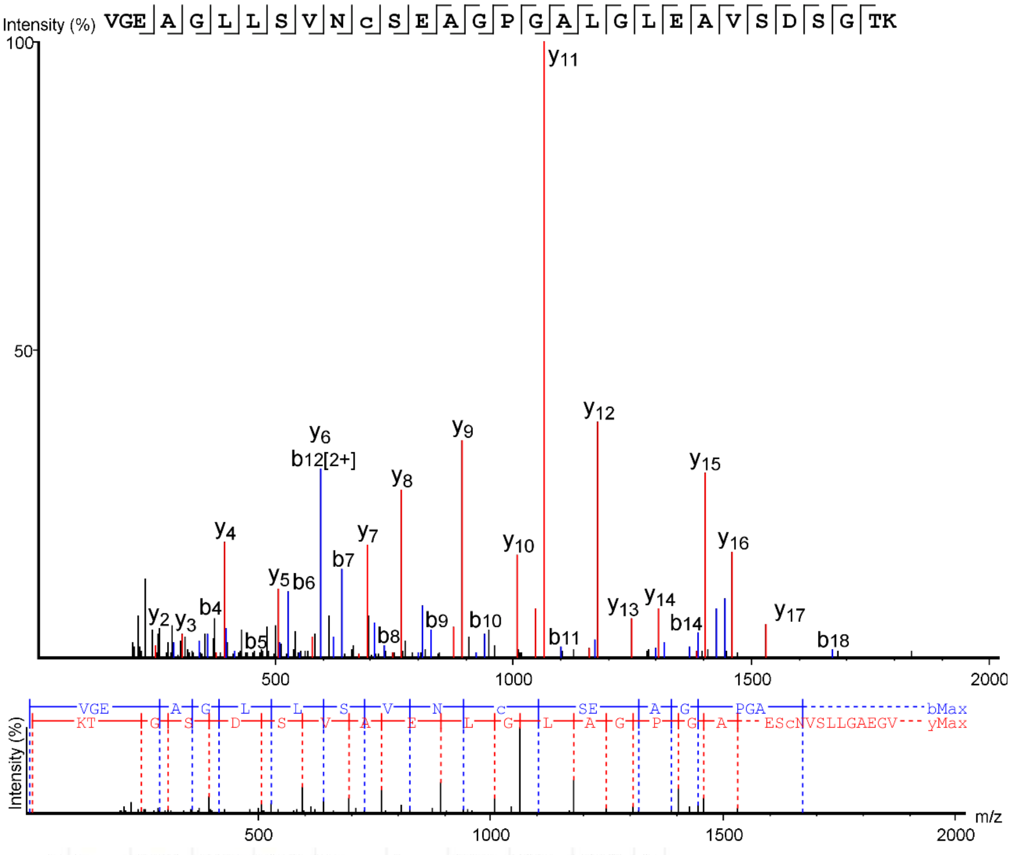

#	b	b-H2O	b-NH3	b (2+)	Seq	y	y-H2O	y-NH3	y (2+)	#
1	100.08	82.07	83.05	50.54	V					30
2	157.10	139.09	140.07	79.05	G	2746.32	2728.31	2729.29	1373.66	29
3	286.14	268.13	269.11	143.57	E	2689.30	2671.29	2672.27	1345.15	28
4	357.18	339.17	340.15	179.09	A	2560.26	2542.25	2543.23	1280.63	27
5	414.20	396.19	397.17	207.60	G	2489.22	2471.21	2472.19	1245.11	26
6	527.28	509.27	510.26	264.14	L	2432.20	2414.19	2415.17	1216.60	25
7	640.37	622.36	623.34	320.68	L	2319.11	2301.10	2302.09	1160.06	24
8	727.40	709.39	710.37	364.20	S	2206.03	2188.02	2189.00	1103.51	23
9	826.47	808.46	809.45	413.73	V	2119.00	2100.99	2101.97	1060.00	22
10	940.51	922.49	923.48	470.76	N	2019.93	2001.92	2002.90	1010.46	21
11	1100.54	1082.53	1083.51	550.77	C(+57.02)	1905.89	1887.88	1888.86	953.44	20
12	1187.57	1169.56	1170.56	594.27	S	1745.86	1727.84	1728.83	873.43	19
13	1316.62	1298.60	1299.59	658.81	E	1658.82	1640.81	1641.80	829.91	18
14	1387.66	1369.65	1370.63	694.33	A	1529.78	1511.77	1512.75	765.39	17
15	1444.67	1426.67	1427.65	722.84	G	1458.75	1440.73	1441.72	729.87	16
16	1541.73	1523.72	1524.70	771.36	P	1401.72	1383.71	1384.69	701.36	15
17	1598.75	1580.74	1581.72	799.88	G	1304.66	1286.66	1287.64	652.83	14
18	1669.79	1651.78	1652.76	835.39	A	1247.65	1229.64	1230.62	624.32	13
19	1782.87	1764.86	1765.84	891.93	L	1176.61	1158.60	1159.58	588.81	12
20	1839.89	1821.88	1822.86	920.45	G	1063.53	1045.52	1046.50	532.26	11
21	1952.98	1934.96	1935.95	976.99	L	1006.50	988.49	989.48	503.75	10
22	2082.02	2064.01	2064.99	1041.51	E	893.42	875.41	876.39	447.21	9
23	2153.05	2135.04	2136.03	1077.03	A	764.38	746.37	747.35	382.69	8
24	2252.12	2234.11	2235.10	1126.56	V	693.34	675.33	676.31	347.17	7
25	2339.16	2321.14	2322.13	1170.08	S	594.27	576.26	577.25	297.64	6
26	2454.18	2436.17	2437.16	1227.59	D	507.24	489.23	490.21	254.12	5
27	2541.21	2523.20	2524.19	1271.11	S	392.21	374.20	375.19	196.61	4
28	2598.24	2580.23	2581.21	1299.62	G	305.18	287.17	288.15	153.09	3
29	2699.28	2681.27	2682.26	1350.14	T	248.16	230.15	231.13	124.58	2
30					K	147.11	129.10	130.09	74.06	1

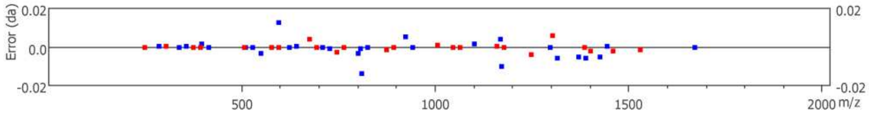

Peptide Sequence	Gene	Dataset	Sample	Score	ScanID
NALTGLPSGLFQASATLDTLVLK	LRG1	1D50	COPD10	116.16	33137

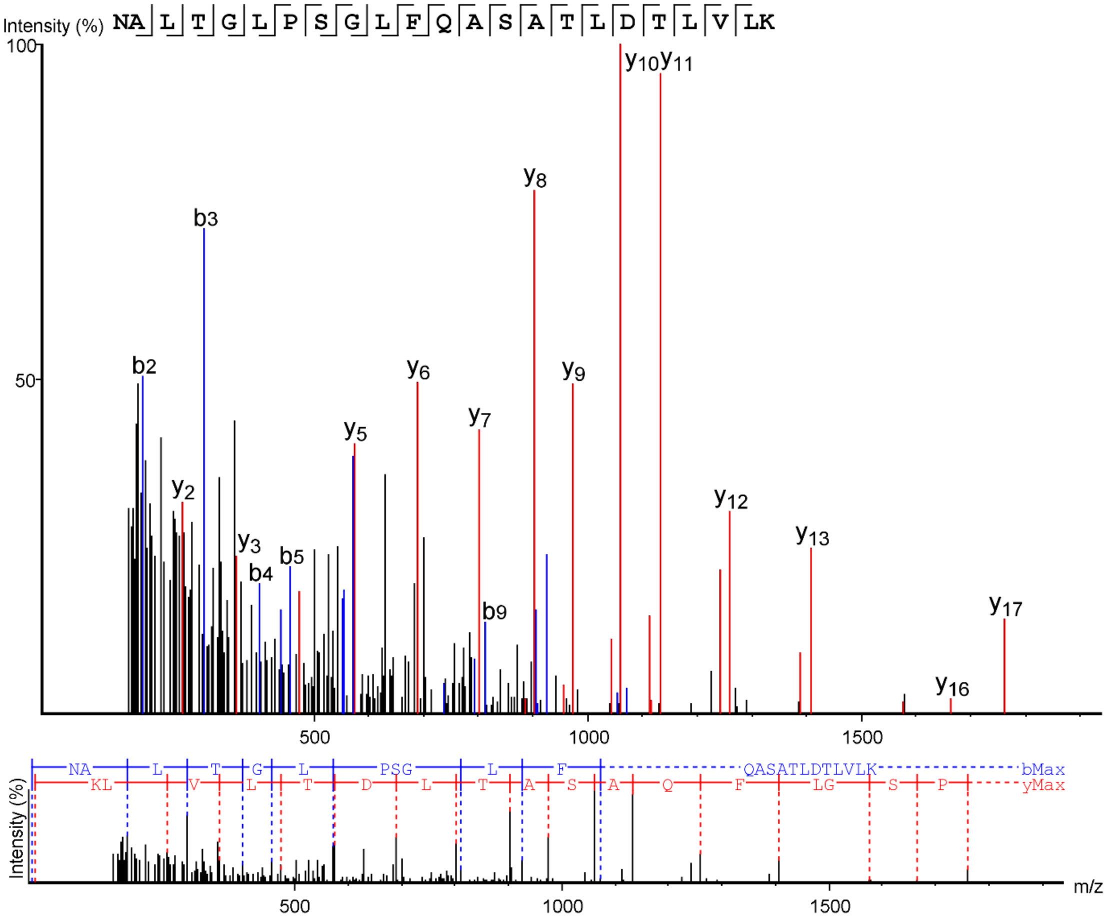

#	b	b-H2O	b-NH3	b (2+)	Seq	y	y-H2O	y-NH3	y (2+)	#
1	115.05	97.04	98.02	58.03	N					23
2	186.09	168.08	169.06	93.54	A	2216.25	2198.24	2199.23	1108.63	22
3	299.17	281.16	282.14	150.09	L	2145.22	2127.21	2128.19	1073.11	21
4	400.22	382.21	383.19	200.61	T	2032.13	2014.12	2015.11	1016.57	20
5	457.24	439.23	440.21	229.12	G	1931.08	1913.07	1914.06	966.04	19
6	570.32	552.31	553.30	285.66	L	1874.06	1856.05	1857.04	937.53	18
7	667.38	649.37	650.35	334.19	P	1760.99	1742.97	1743.95	880.99	17
8	754.41	736.40	737.38	377.70	S	1663.94	1645.92	1646.90	832.46	16
9	811.43	793.42	794.41	406.22	G	1576.90	1558.88	1559.87	788.95	15
10	924.52	906.50	907.50	462.76	L	1519.87	1501.86	1502.85	760.44	14
11	1071.58	1053.57	1054.56	536.29	F	1406.79	1388.77	1389.76	703.89	13
12	1199.64	1181.63	1182.62	600.32	Q	1259.72	1241.71	1242.70	630.36	12
13	1270.68	1252.67	1253.65	635.84	A	1131.66	1113.66	1114.65	566.33	11
14	1357.71	1339.70	1340.68	679.36	S	1060.62	1042.61	1043.61	530.81	10
15	1428.75	1410.74	1411.72	714.87	A	973.59	955.58	956.57	487.30	9
16	1529.80	1511.79	1512.77	765.40	T	902.55	884.54	885.53	451.78	8
17	1642.88	1624.87	1625.85	821.94	L	801.51	783.50	784.48	401.25	7
18	1757.91	1739.90	1740.88	879.45	D	688.42	670.41	671.40	344.71	6
19	1858.96	1840.94	1841.93	929.98	T	573.40	555.39	556.37	287.20	5
20	1972.04	1954.03	1955.01	986.52	L	472.35	454.34	455.32	236.67	4
21	2071.11	2053.10	2054.08	1036.05	V	359.27	341.25	342.24	180.13	3
22	2184.19	2166.18	2167.16	1092.60	L	260.20	242.19	243.17	130.60	2
23					K	147.11	129.10	130.09	74.06	1

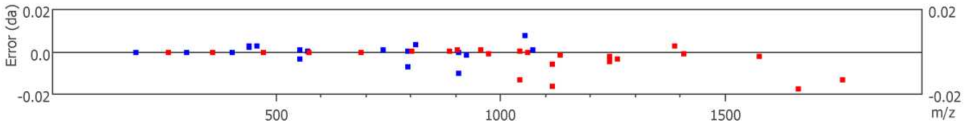

Brandsma C-A, *et al. Thorax* 2020; 75:180–183. doi: 10.1136/thoraxjnl-2019-213200

Peptide Sequence	Gene	Dataset	Sample	Score	ScanID
AEAEDTSKDPVGR	MMRN2	2D25	COPD7	86.1	3034

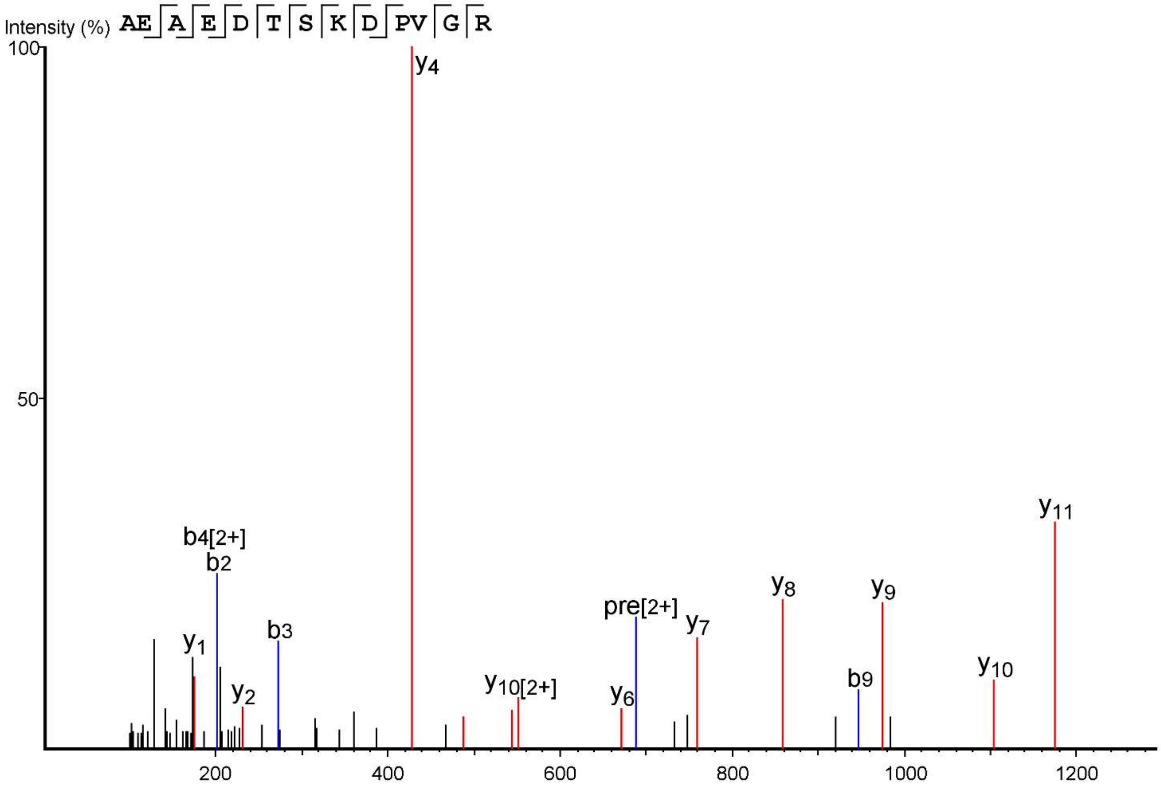

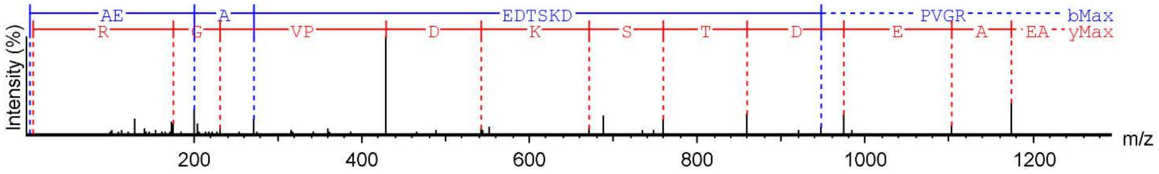

#	b	b-H2O	b-NH3	b (2+)	Seq	y	y-H2O	y-NH3	y (2+)	#
1	72.04	54.03	55.02	36.52	A					13
2	201.09	183.08	184.06	101.04	E	1303.61	1285.60	1286.59	652.31	12
3	272.12	254.11	255.10	136.56	A	1174.57	1156.56	1157.54	587.78	11
4	401.17	383.16	384.14	201.09	E	1103.53	1085.52	1086.51	552.27	10
5	516.19	498.18	499.17	258.60	D	974.48	956.48	957.46	487.75	9
6	617.24	599.23	600.21	309.12	T	859.46	841.45	842.44	430.23	8
7	704.27	686.26	687.25	352.64	S	758.41	740.40	741.39	379.71	7
8	832.37	814.36	815.34	416.68	K	671.38	653.37	654.36	336.19	6
9	947.39	929.39	930.37	474.20	D	543.29	525.28	526.26	272.14	5
10	1044.45	1026.44	1027.42	522.72	P	428.26	410.25	411.23	214.63	4
11	1143.52	1125.51	1126.49	572.26	V	331.21	313.20	314.18	166.10	3
12	1200.54	1182.53	1183.51	600.77	G	232.14	214.13	215.11	116.57	2
13					R	175.12	157.11	158.09	88.06	1

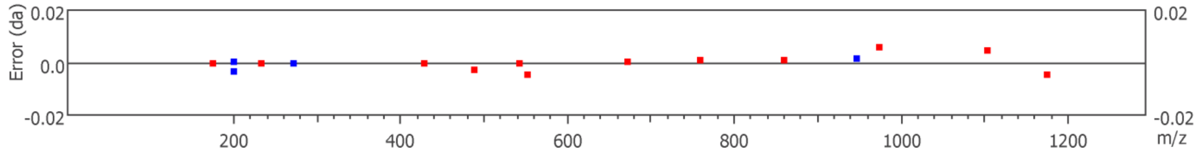

Brandsma C-A, *et al. Thorax* 2020; 75:180–183. doi: 10.1136/thoraxjnl-2019-213200

Peptide Sequence	Gene	Dataset	Sample	Score	ScanID
NKYEDEINRR	KRT7	2D25	COPD7	82.99	4279

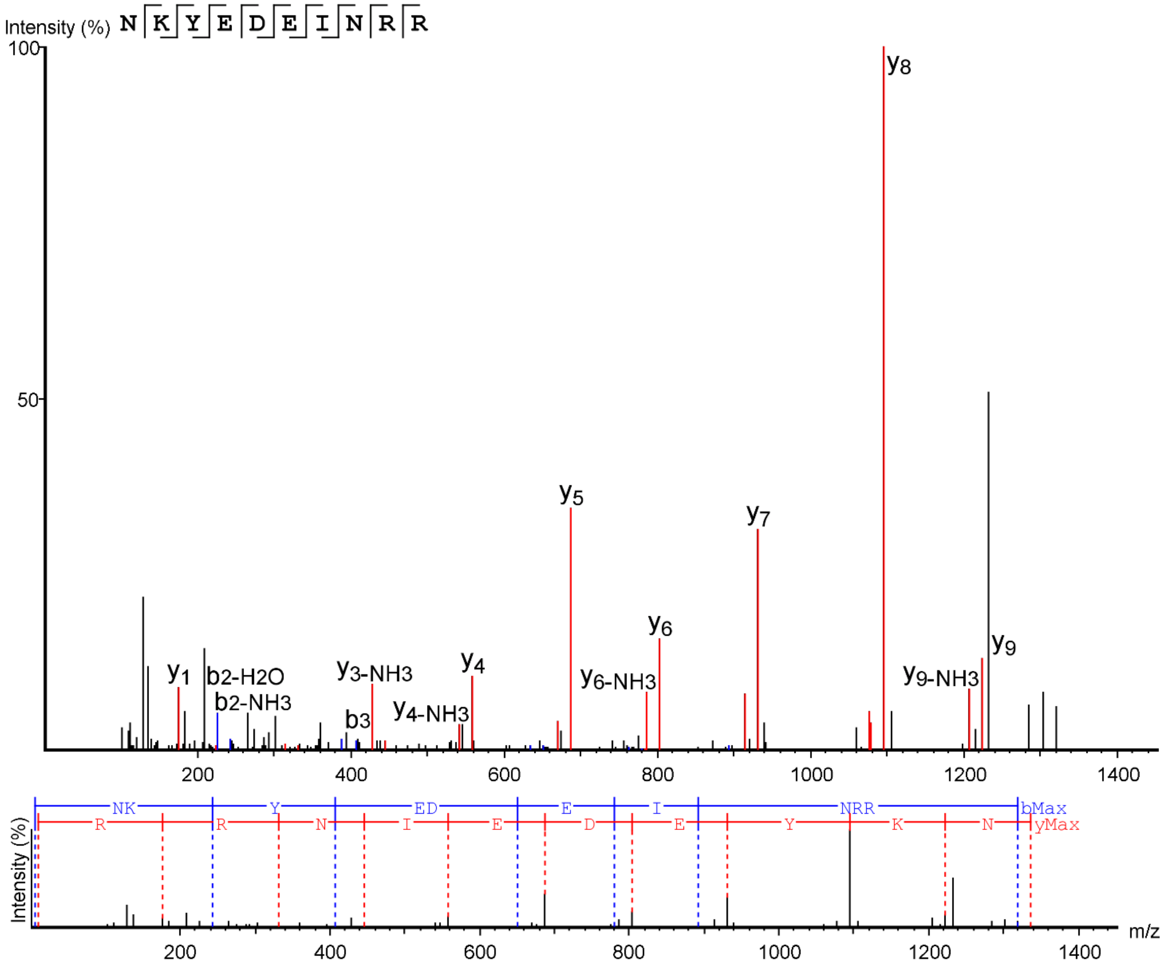

#	b	b-H2O	b-NH3	b (2+)	Seq	y	y-H2O	y-NH3	y (2+)	#
1	115.05	97.04	98.02	58.03	N					10
2	243.14	225.13	226.12	122.07	K	1222.62	1204.61	1205.59	611.81	9
3	406.21	388.20	389.18	203.60	Y	1094.52	1076.50	1077.50	547.76	8
4	535.25	517.24	518.22	268.13	E	931.46	913.45	914.43	466.23	7
5	650.27	632.27	633.25	325.64	D	802.42	784.40	785.39	401.71	6
6	779.32	761.31	762.29	390.16	E	687.39	669.38	670.36	344.19	5
7	892.41	874.39	875.38	446.70	I	558.35	540.34	541.32	279.67	4
8	1006.45	988.44	989.42	503.72	N	445.26	427.25	428.24	223.13	3
9	1162.55	1144.54	1145.52	581.77	R	331.22	313.21	314.19	166.11	2
10					R	175.12	157.11	158.09	88.06	1

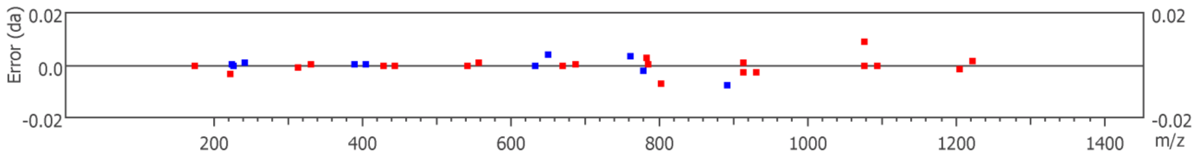

Brandsma C-A, *et al. Thorax* 2020; 75:180–183. doi: 10.1136/thoraxjnl-2019-213200

Peptide Sequence	Gene	Dataset	Sample	Score	ScanID
AEDEENEKETAVSTEDDSHHK	SPARCL1	2D25	COPD3	104.77	2117

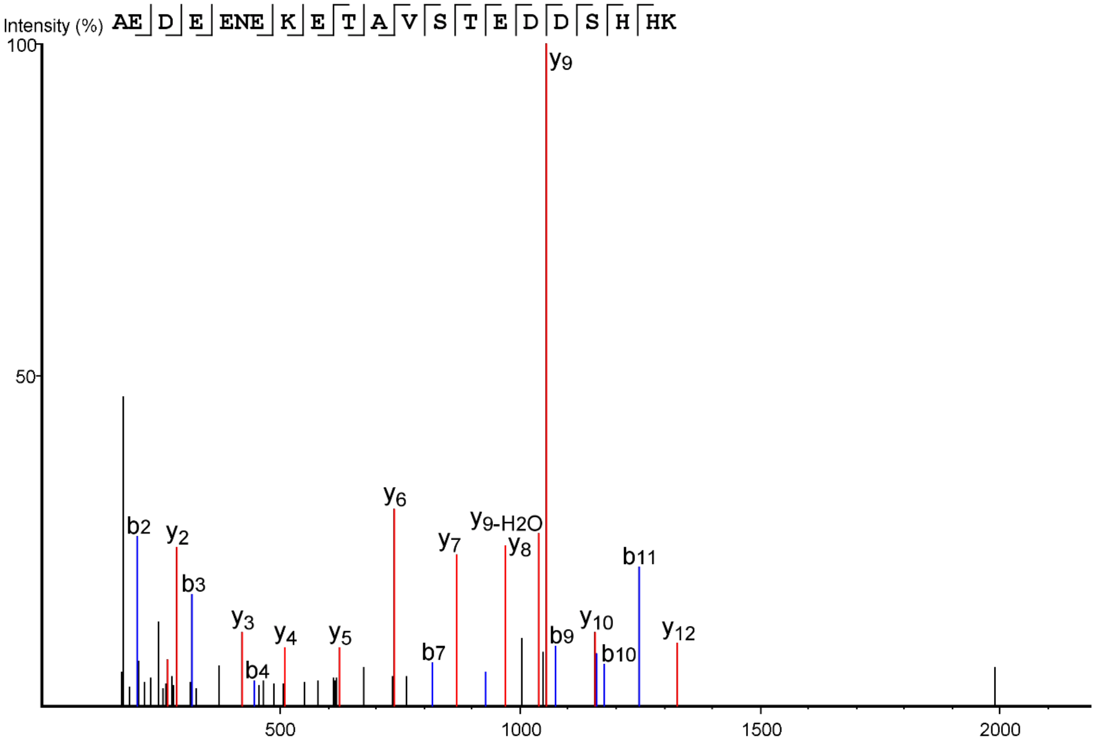

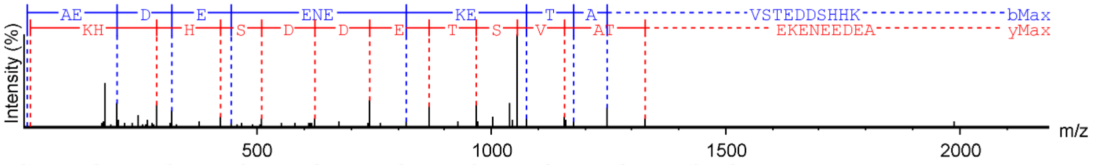

#	b	b-H2O	b-NH3	b (2+)	Seq	y	y-H2O	y-NH3	y (2+)	#
1	72.04	54.03	55.02	36.52	A					21
2	201.09	183.08	184.06	101.04	E	2328.97	2310.96	2311.94	1164.98	20
3	316.11	298.10	299.09	158.56	D	2199.93	2181.92	2182.90	1100.46	19
4	445.16	427.15	428.13	223.08	E	2084.90	2066.89	2067.87	1042.95	18
5	574.20	556.19	557.17	287.60	E	1955.86	1937.85	1938.83	978.43	17
6	688.24	670.23	671.22	344.62	N	1826.82	1808.80	1809.79	913.91	16
7	817.28	799.27	800.26	409.14	E	1712.77	1694.76	1695.75	856.89	15
8	945.38	927.37	928.35	473.19	K	1583.73	1565.72	1566.70	792.36	14
9	1074.42	1056.41	1057.40	537.71	E	1455.63	1437.62	1438.61	728.32	13
10	1175.48	1157.45	1158.44	588.24	T	1326.59	1308.58	1309.57	663.80	12
11	1246.51	1228.50	1229.48	623.75	A	1225.54	1207.53	1208.52	613.27	11
12	1345.58	1327.57	1328.55	673.29	V	1154.50	1136.50	1137.48	577.75	10
13	1432.61	1414.60	1415.58	716.80	S	1055.44	1037.43	1038.41	528.22	9
14	1533.66	1515.65	1516.63	767.33	T	968.41	950.40	951.38	484.70	8
15	1662.70	1644.69	1645.67	831.85	E	867.36	849.35	850.33	434.18	7
16	1777.73	1759.71	1760.70	889.36	D	738.32	720.31	721.29	369.66	6
17	1892.75	1874.74	1875.73	946.88	D	623.29	605.28	606.26	312.14	5
18	1979.78	1961.77	1962.76	990.39	S	508.26	490.25	491.24	254.63	4
19	2116.84	2098.83	2099.82	1058.92	H	421.23	403.22	404.20	211.12	3
20	2253.90	2235.89	2236.87	1127.45	H	284.17	266.16	267.14	142.59	2
21					K	147.11	129.10	130.09	74.06	1

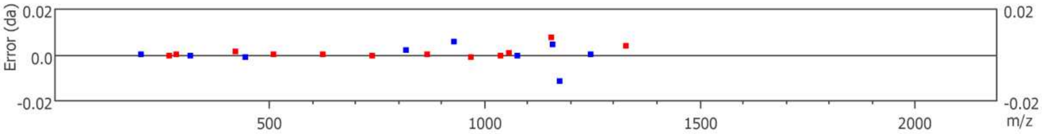

Brandsma C-A, *et al. Thorax* 2020; 75:180–183. doi: 10.1136/thoraxjnl-2019-213200

Peptide Sequence	Gene	Dataset	Sample	Score	ScanID
VSYGIGEEEHDQEGR	APEX1	2D25	Control8	115.91	6280

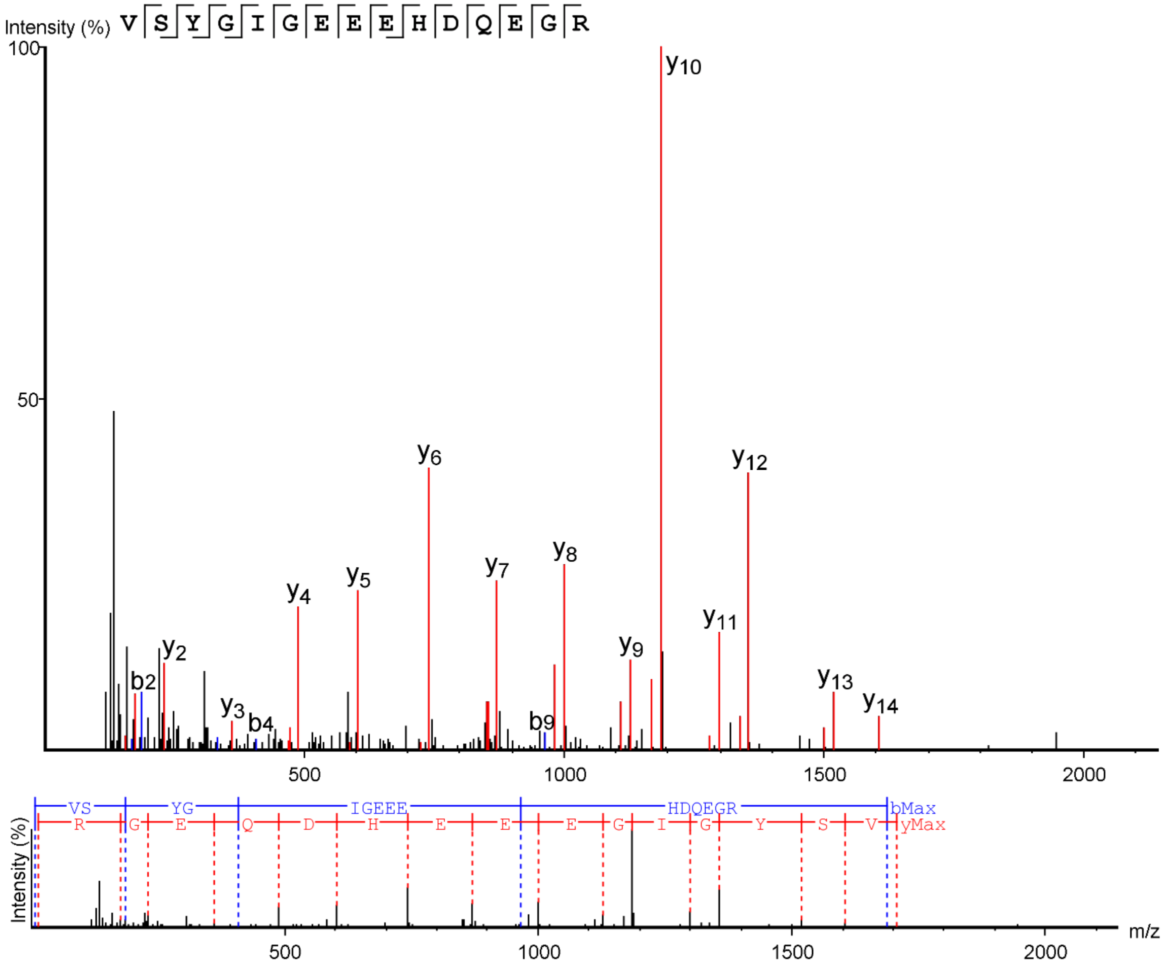

#	b	b-H2O	b-NH3	b (2+)	Seq	y	y-H2O	y-NH3	y (2+)	#
1	100.08	82.07	83.05	50.54	V					15
2	187.11	169.10	170.08	94.05	S	1605.68	1587.67	1588.65	803.34	14
3	350.17	332.16	333.14	175.59	Y	1518.65	1500.63	1501.62	759.82	13
4	407.19	389.18	390.17	204.10	G	1355.58	1337.57	1338.57	678.29	12
5	520.28	502.27	503.25	260.64	I	1298.56	1280.54	1281.53	649.78	11
6	577.30	559.29	560.27	289.15	G	1185.48	1167.47	1168.46	593.24	10
7	706.34	688.33	689.31	353.67	E	1128.46	1110.45	1111.43	564.73	9
8	835.38	817.37	818.36	418.19	E	999.41	981.40	982.39	500.21	8
9	964.43	946.42	947.40	482.71	E	870.37	852.36	853.35	435.68	7
10	1101.49	1083.47	1084.46	551.24	H	741.33	723.32	724.30	371.16	6
11	1216.51	1198.50	1199.49	608.76	D	604.27	586.26	587.24	302.63	5
12	1344.57	1326.56	1327.54	672.79	Q	489.24	471.23	472.21	245.12	4
13	1473.61	1455.60	1456.59	737.31	E	361.18	343.17	344.16	181.09	3
14	1530.63	1512.62	1513.61	765.82	G	232.14	214.13	215.11	116.57	2
15					R	175.12	157.11	158.09	88.06	1

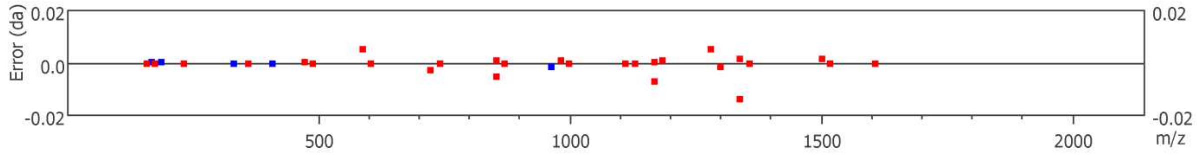

Brandsma C-A, *et al. Thorax* 2020; 75:180–183. doi: 10.1136/thoraxjnl-2019-213200

Peptide Sequence	Gene	Dataset	Sample	Score	ScanID
VLVPSLLHTETTEK	A2M	2D25	COPD6	84.08	13517

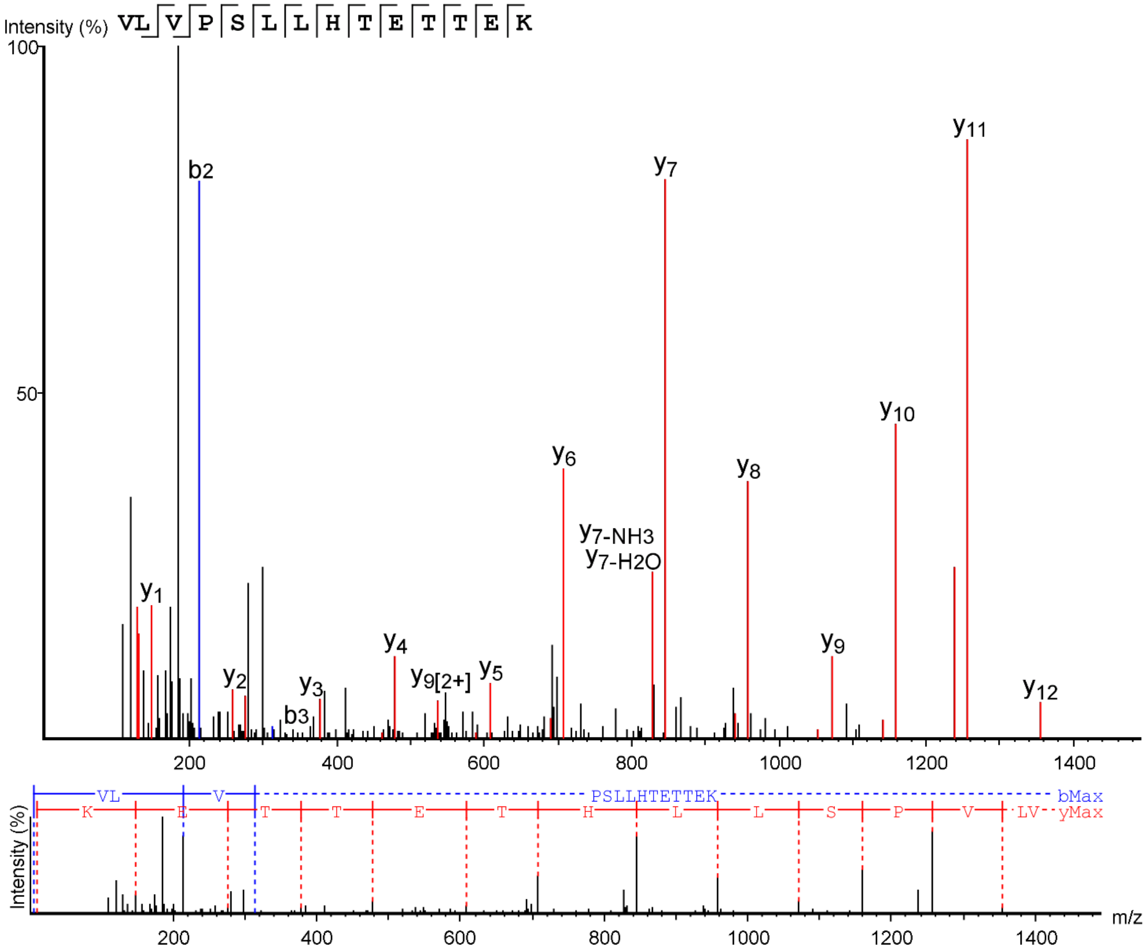

#	b	b-H2O	b-NH3	b (2+)	Seq	y	y-H2O	y-NH3	y (2+)	#
1	100.08	82.07	83.05	50.54	V					14
2	213.16	195.15	196.13	107.08	L	1467.81	1449.79	1450.78	734.40	13
3	312.23	294.22	295.20	156.61	V	1354.72	1336.71	1337.69	677.86	12
4	409.28	391.27	392.25	205.14	P	1255.65	1237.64	1238.63	628.33	11
5	496.31	478.30	479.29	248.66	S	1158.60	1140.60	1141.57	579.80	10
6	609.40	591.39	592.37	305.20	L	1071.57	1053.56	1054.54	536.29	9
7	722.48	704.47	705.45	361.74	L	958.48	940.47	941.46	479.74	8
8	859.54	841.53	842.51	430.27	H	845.40	827.39	828.39	423.20	7
9	960.59	942.58	943.56	480.79	T	708.34	690.33	691.31	354.67	6
10	1089.63	1071.62	1072.60	545.32	E	607.29	589.28	590.27	304.15	5
11	1190.68	1172.67	1173.65	595.84	T	478.25	460.24	461.22	239.63	4
12	1291.73	1273.72	1274.70	646.36	T	377.20	359.19	360.18	189.10	3
13	1420.77	1402.76	1403.74	710.88	E	276.16	258.14	259.13	138.58	2
14					K	147.11	129.10	130.09	74.06	1

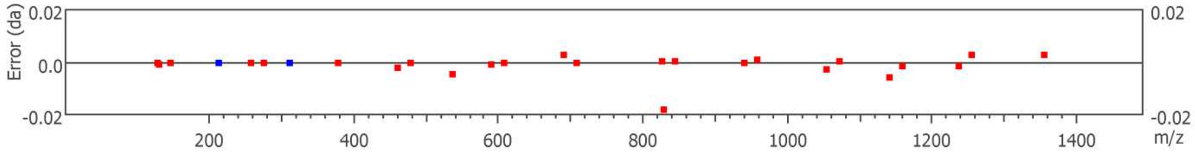

Brandsma C-A, *et al. Thorax* 2020; 75:180–183. doi: 10.1136/thoraxjnl-2019-213200

Peptide Sequence	Gene	Dataset	Sample	Score	ScanID
GATYNIIVEALK	FN1	1D50	COPD8	73.04	23536

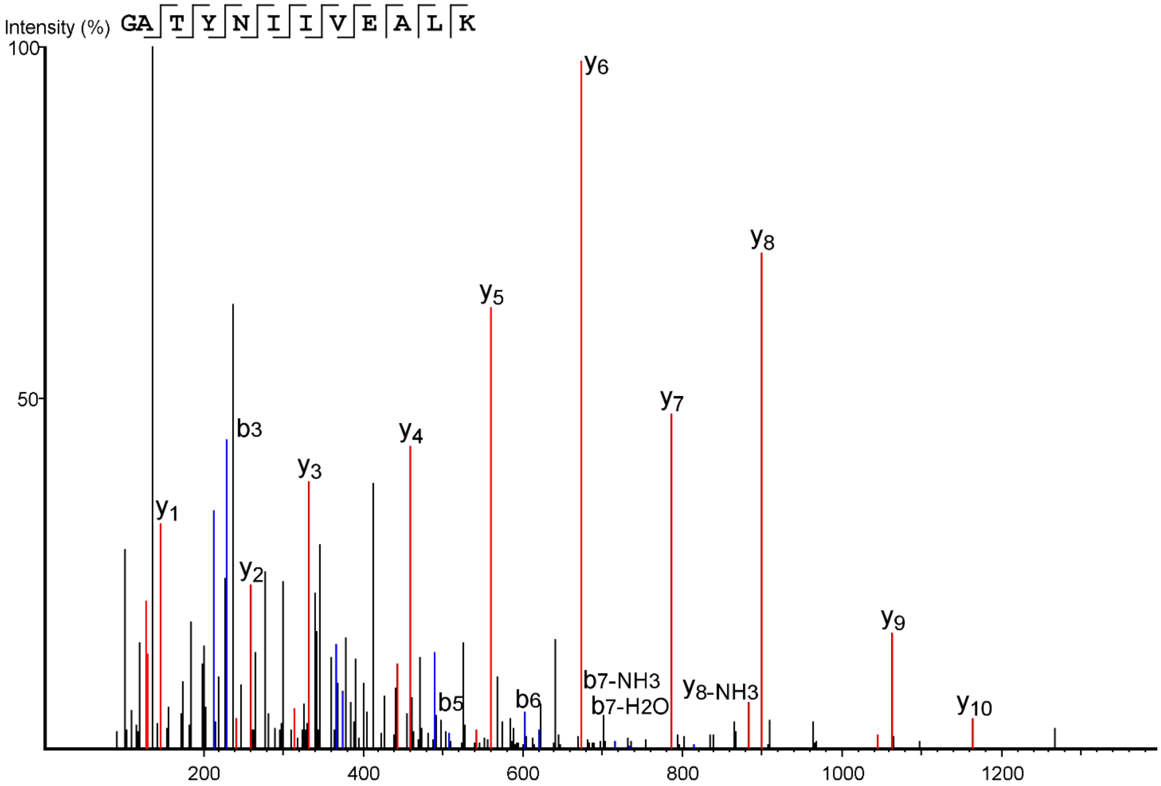

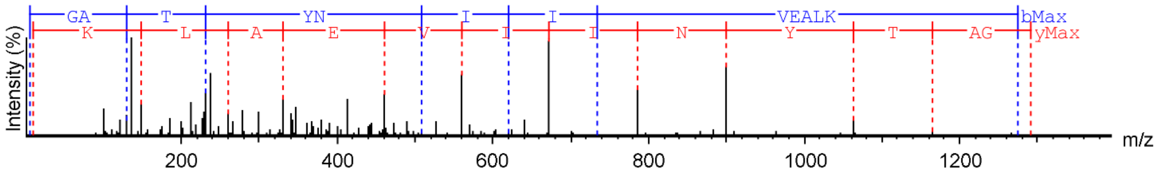

#	b	b-H2O	b-NH3	b (2+)	Seq	y	y-H2O	y-NH3	y (2+)	#
1	58.03	40.02	41.00	29.51	G					12
2	129.07	111.06	112.04	65.03	A	1234.70	1216.69	1217.68	617.85	11
3	230.11	212.10	213.09	115.56	T	1163.66	1145.66	1146.64	582.33	10
4	393.18	375.17	376.15	197.09	Y	1062.62	1044.61	1045.59	531.81	9
5	507.22	489.21	490.19	254.11	N	899.55	881.55	882.53	450.28	8
6	620.30	602.29	603.28	310.65	I	785.51	767.50	768.49	393.26	7
7	733.40	715.37	716.36	367.21	I	672.43	654.42	655.40	336.71	6
8	832.46	814.44	815.43	416.73	V	559.34	541.34	542.30	280.17	5
9	961.50	943.49	944.47	481.25	E	460.28	442.27	443.25	230.64	4
10	1032.54	1014.53	1015.51	516.77	A	331.23	313.22	314.21	166.12	3
11	1145.62	1127.61	1128.59	573.31	L	260.20	242.19	243.17	130.60	2
12					K	147.11	129.10	130.09	74.06	1

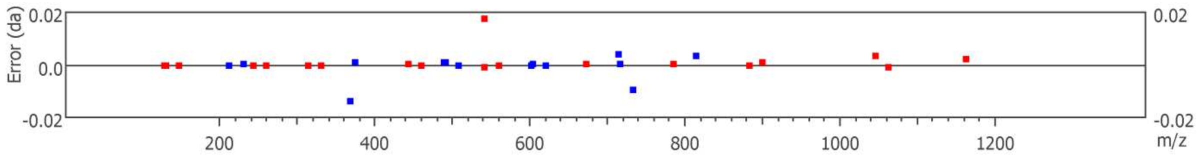

Brandsma C-A, *et al. Thorax* 2020; 75:180–183. doi: 10.1136/thoraxjnl-2019-213200

Peptide Sequence	Gene	Dataset	Sample	Score	ScanID
DLTGFPGPLNDQDDEDCINR	A2M	1D25	COPD9	138.23	20611

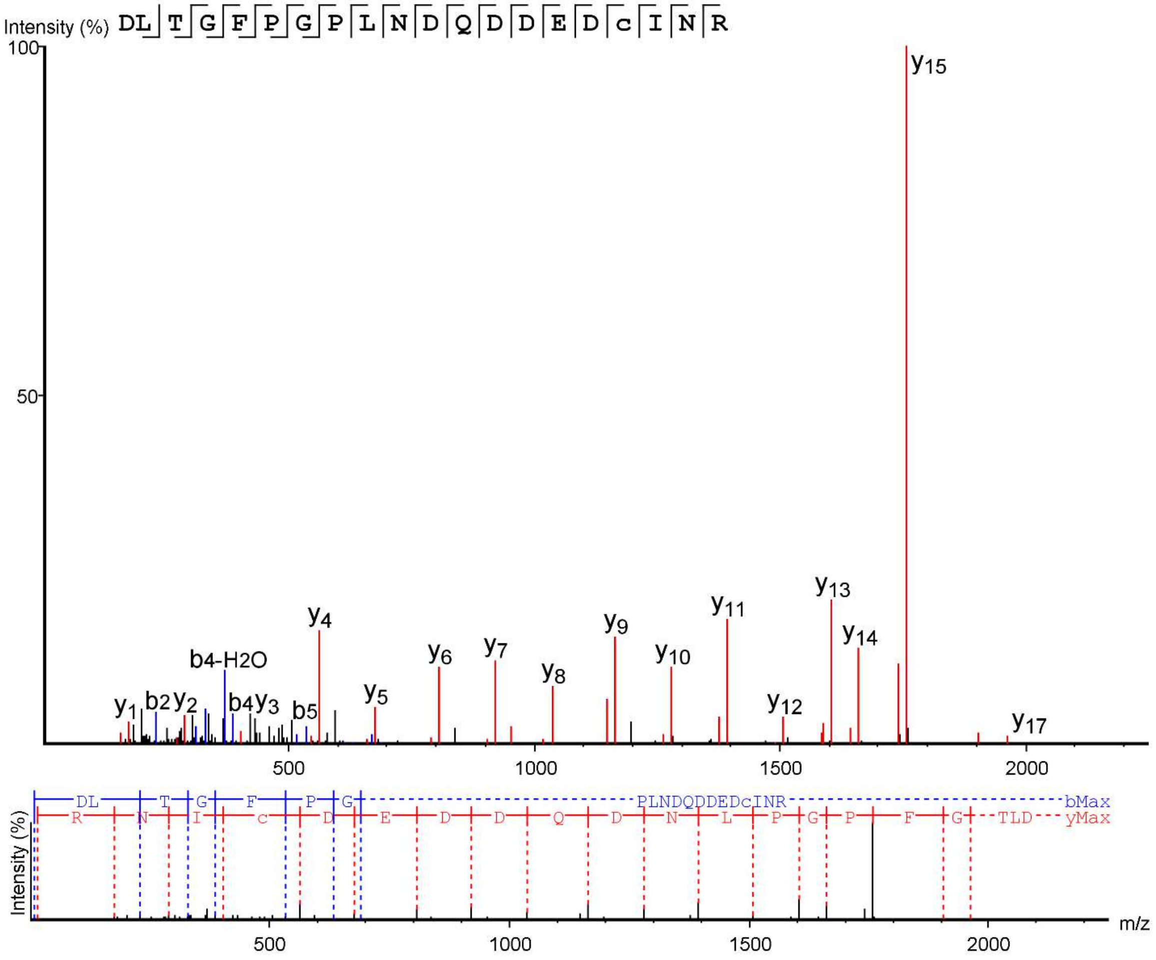

#	b	b-H2O	b-NH3	b (2+)	Seq	y	y-H2O	y-NH3	y (2+)	#
1	116.03	98.02	99.01	58.52	D					20
2	229.12	211.11	212.09	115.06	L	2175.96	2157.95	2158.93	1088.48	19
3	330.16	312.15	313.14	165.58	T	2062.88	2044.87	2045.85	1031.94	18
4	387.19	369.18	370.16	194.09	G	1961.83	1943.82	1944.80	981.41	17
5	534.26	516.24	517.23	267.63	F	1904.80	1886.80	1887.78	952.91	16
6	631.31	613.30	614.28	316.15	P	1757.74	1739.73	1740.72	879.37	15
7	688.32	670.32	671.30	344.67	G	1660.68	1642.67	1643.66	830.84	14
8	785.38	767.37	768.36	393.19	P	1603.66	1585.64	1586.64	802.33	13
9	898.47	880.46	881.44	449.73	L	1506.60	1488.60	1489.60	753.81	12
10	1012.51	994.50	995.48	506.76	N	1393.53	1375.53	1376.50	697.26	11
11	1127.54	1109.53	1110.51	564.27	D	1279.48	1261.47	1262.47	640.24	10
12	1255.60	1237.59	1238.57	628.30	Q	1164.46	1146.44	1147.43	582.73	9
13	1370.62	1352.61	1353.60	685.81	D	1036.40	1018.39	1019.38	518.70	8
14	1485.65	1467.64	1468.62	743.32	D	921.37	903.36	904.35	461.19	7
15	1614.69	1596.68	1597.67	807.85	E	806.34	788.33	789.33	403.67	6
16	1729.72	1711.71	1712.69	865.36	D	677.30	659.30	660.28	339.15	5
17	1889.75	1871.74	1872.72	945.37	C(+57.02)	562.28	544.27	545.25	281.64	4
18	2002.83	1984.82	1985.81	1001.92	I	402.25	384.24	385.22	201.62	3
19	2116.88	2098.87	2099.85	1058.94	N	289.16	271.15	272.13	145.08	2
20					R	175.12	157.11	158.09	88.06	1

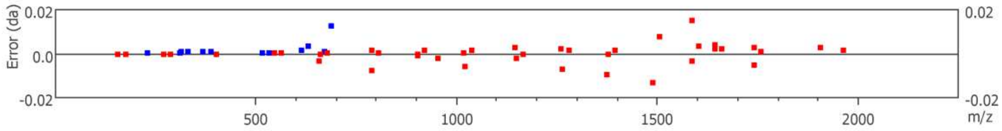

Brandsma C-A, *et al. Thorax* 2020; 75:180–183. doi: 10.1136/thoraxjnl-2019-213200

6 MS/MS spectra visualisation of synthetic and endogenous 23 non-reference peptides present only on COPD or control samples

HEXA

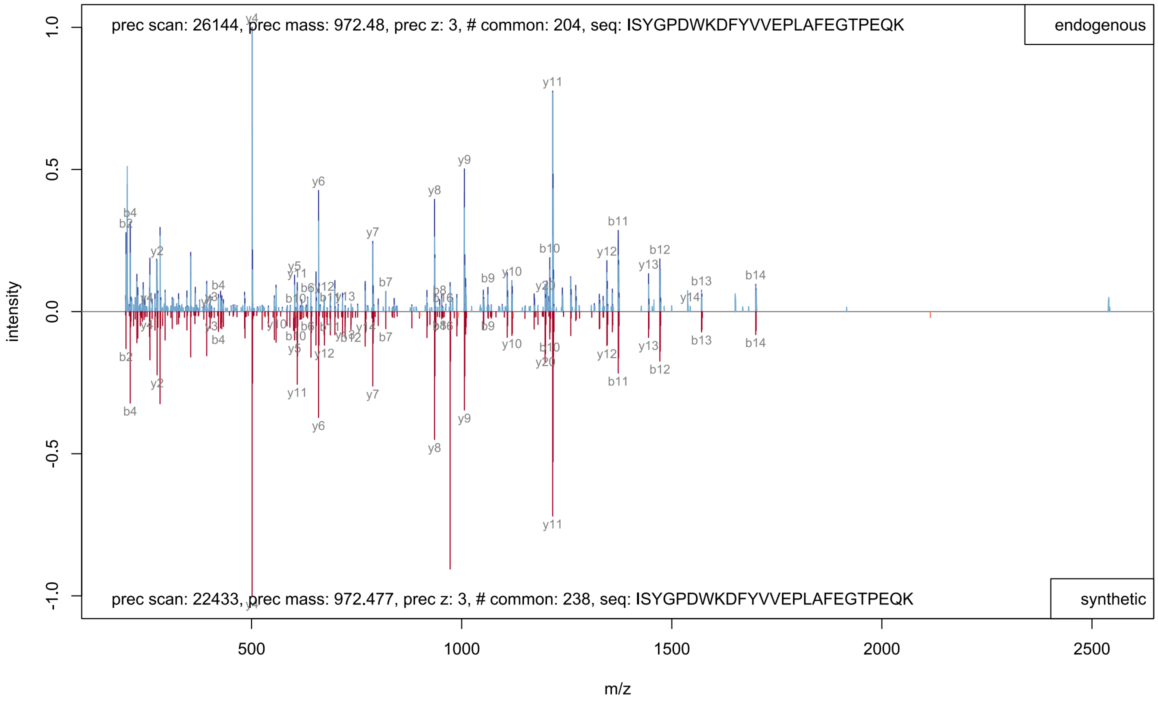

MYH11

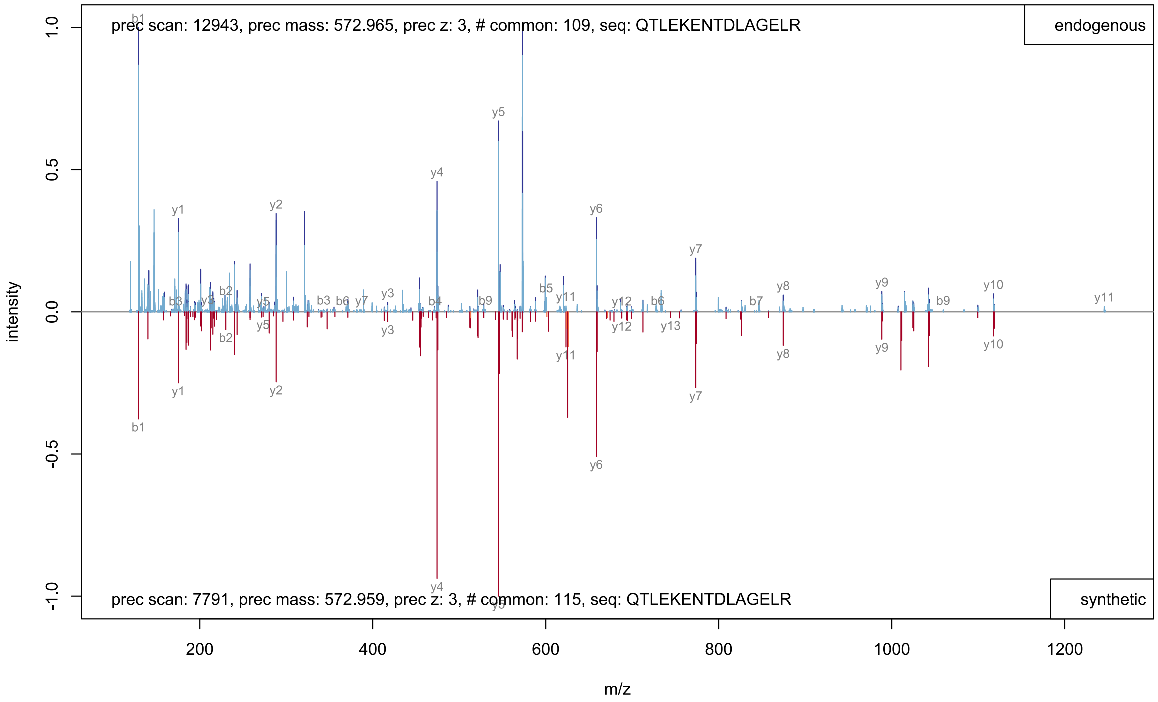

Brandsma C-A, *et al. Thorax* 2020; 75:180–183. doi: 10.1136/thoraxjnl-2019-213200

IGKV3-20

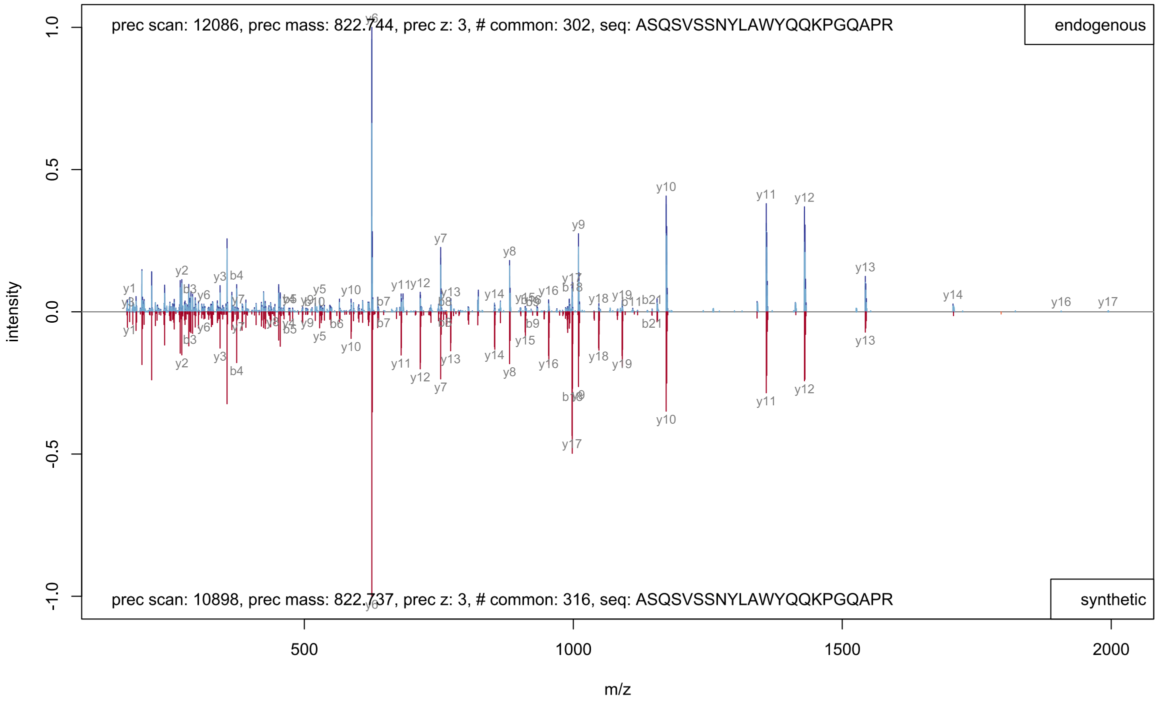
prec scan: 12086, prec mass: 822.744, prec z: 3, # common: 302, seq: ASQSVSSNYLAWYQQKPGQAPR
endogenous
prec scan: 10898, prec mass: 822.737, prec z: 3, # common: 316, seq: ASQSVSSNYLAWYQQKPGQAPR
synthetic
intensity
m/z

C7

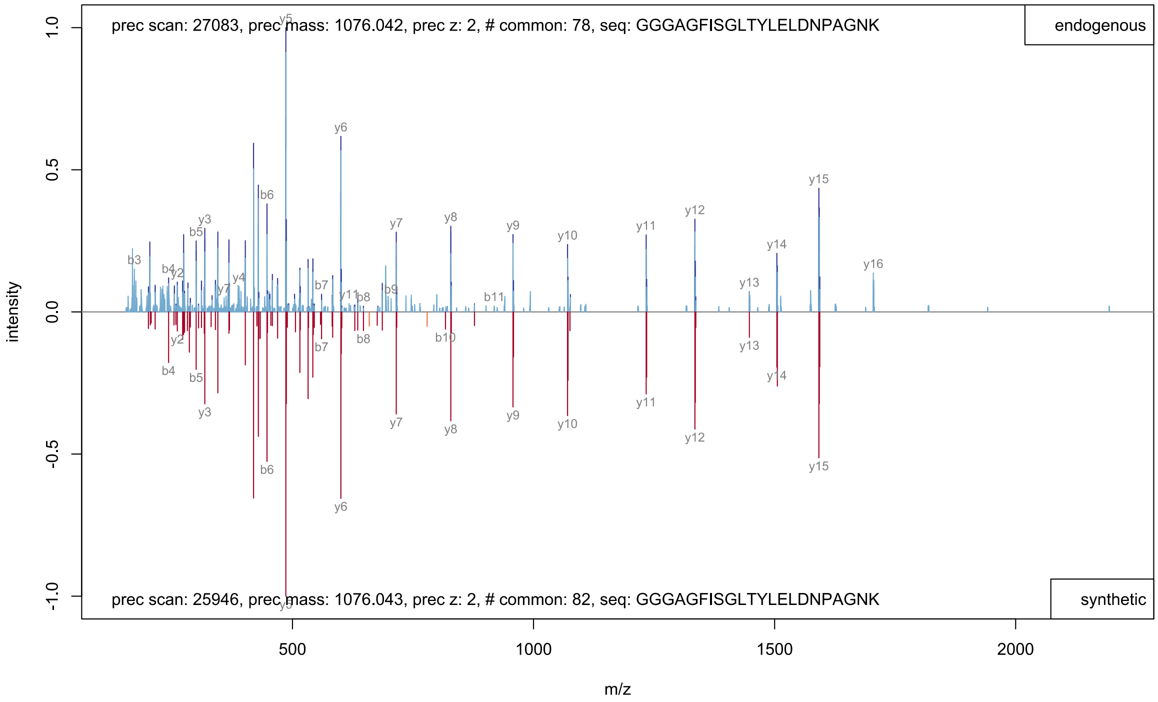
prec scan: 27083, prec mass: 1076.042, prec z: 2, # common: 78, seq: GGGAGFISGLTYLELDNPAGNK
endogenous
prec scan: 25946, prec mass: 1076.043, prec z: 2, # common: 82, seq: GGGAGFISGLTYLELDNPAGNK
synthetic
intensity
m/z

IGKV3-20

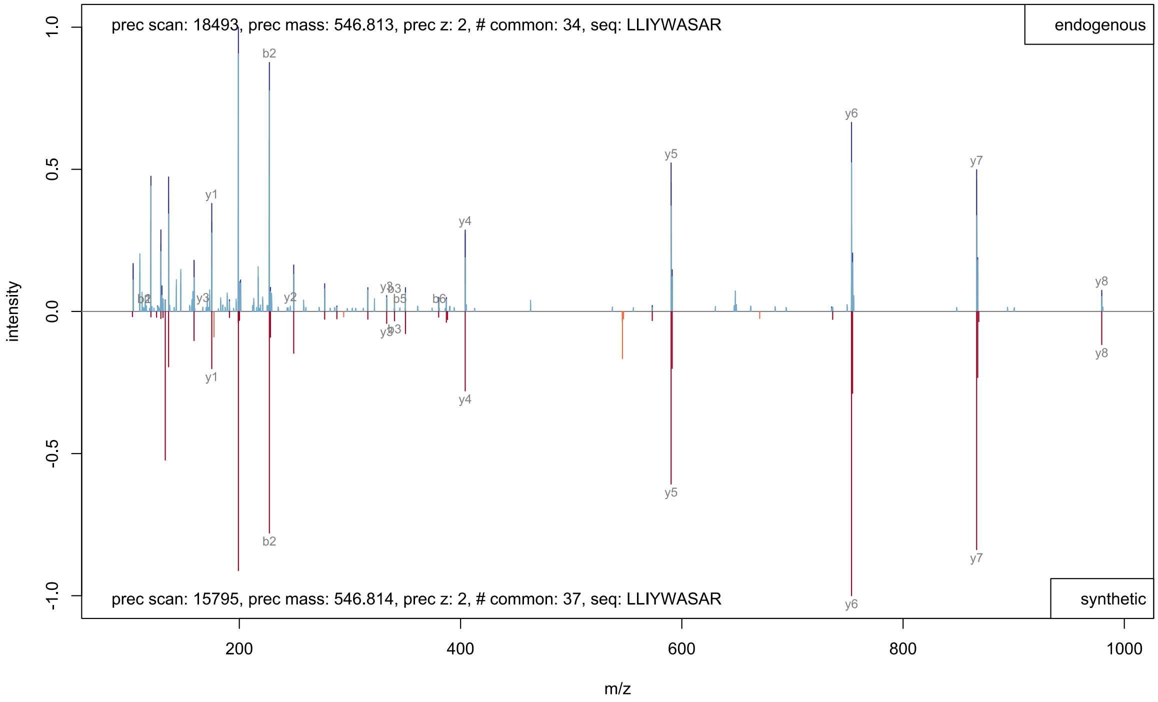

RRBP1

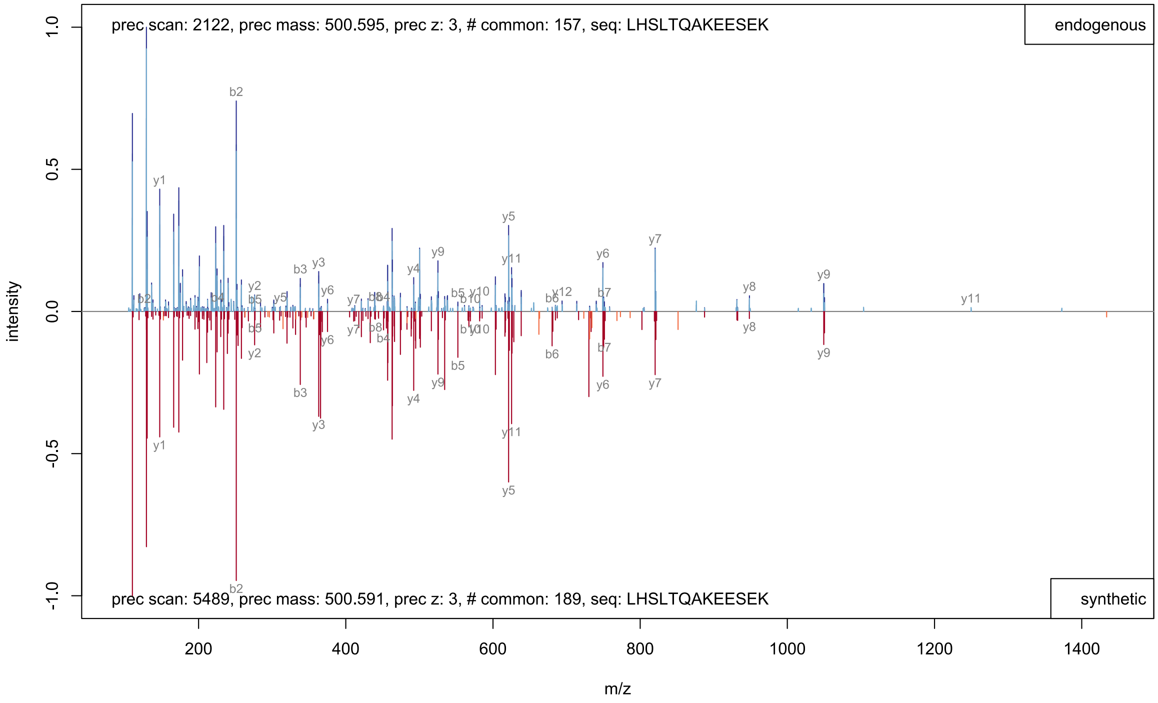

Brandsma C-A, *et al. Thorax* 2020; 75:180–183. doi: 10.1136/thoraxjnl-2019-213200

PDE4DIP

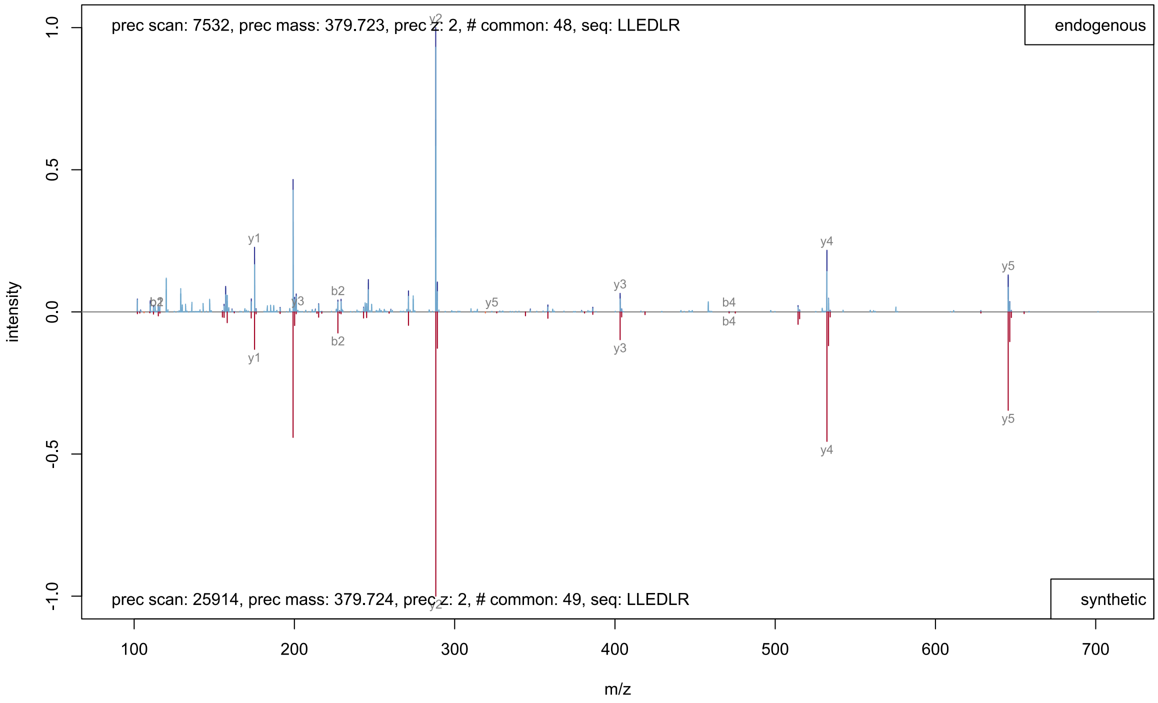

IGHV3-21

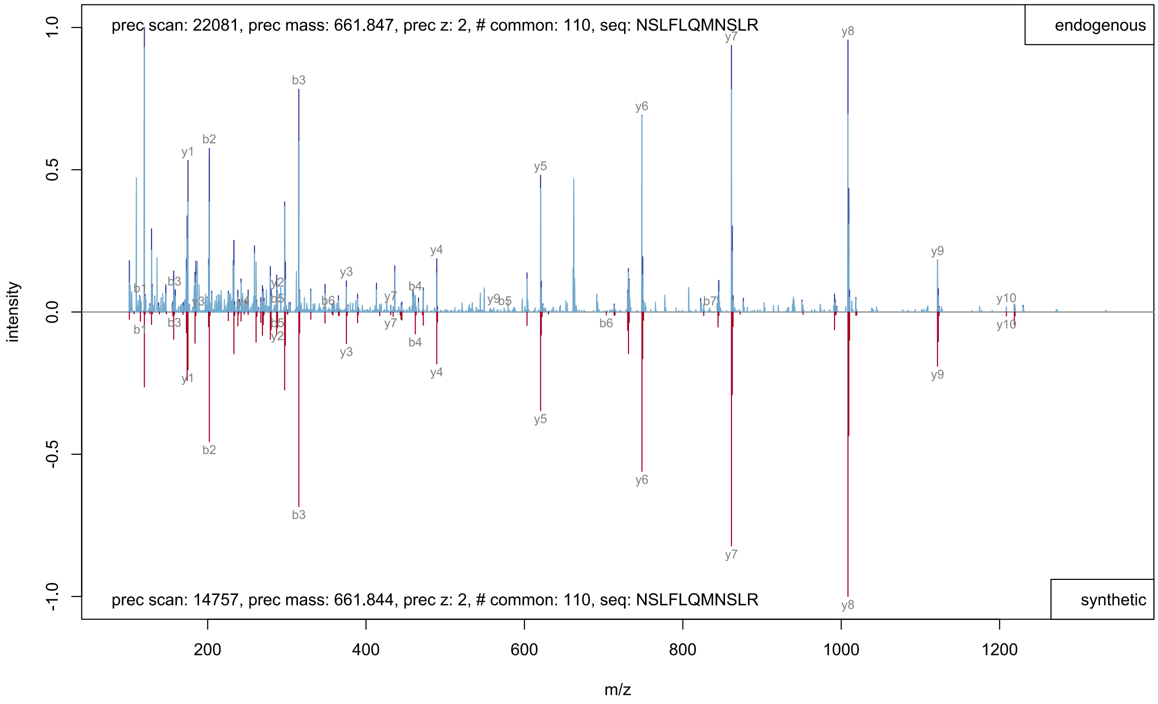

Brandsma C-A, *et al. Thorax* 2020; 75:180–183. doi: 10.1136/thoraxjnl-2019-213200

SORBS1

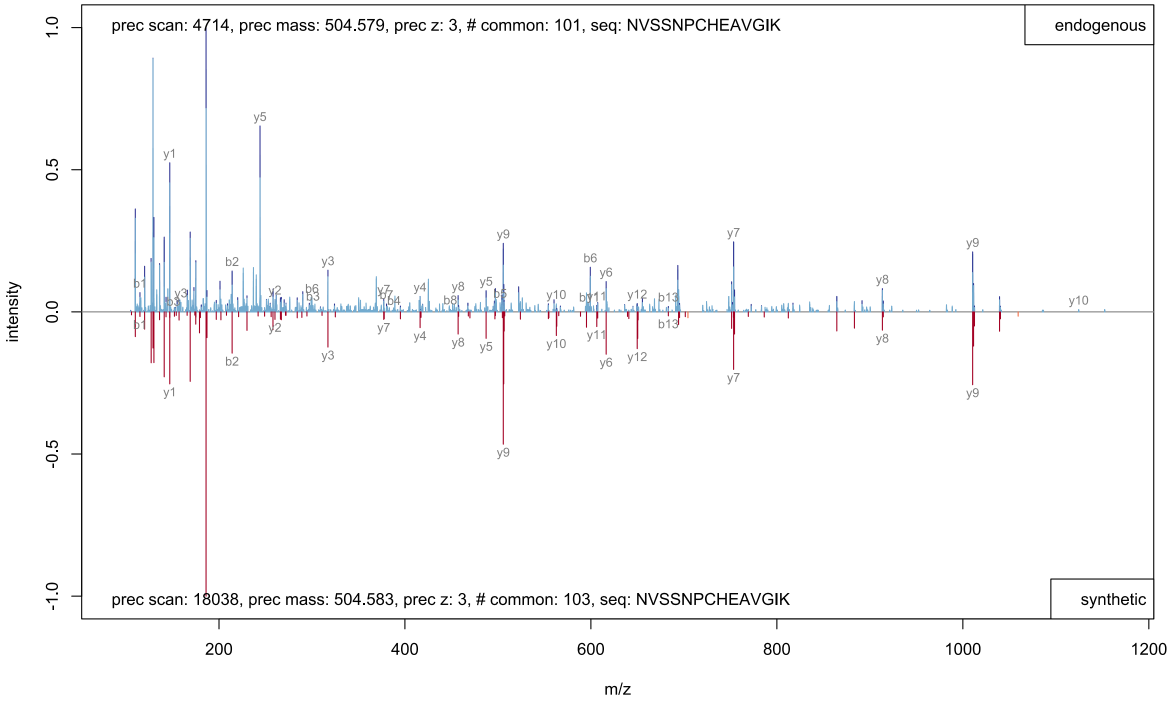

ZNF830

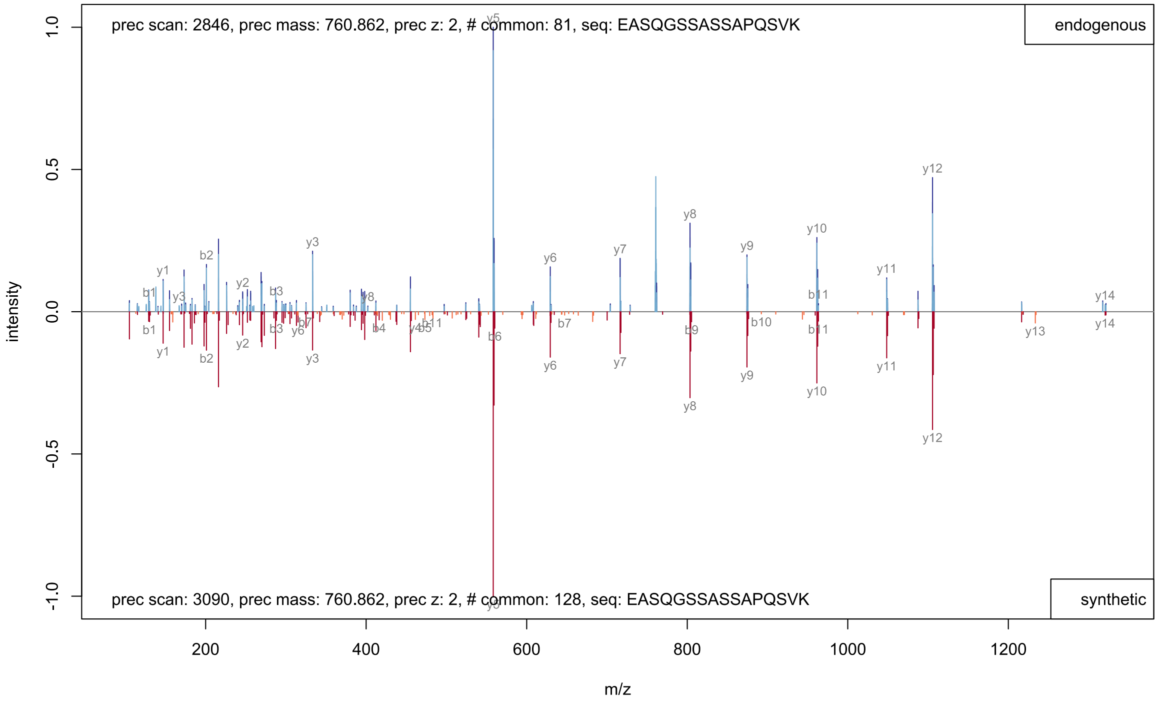

ZFYVE16

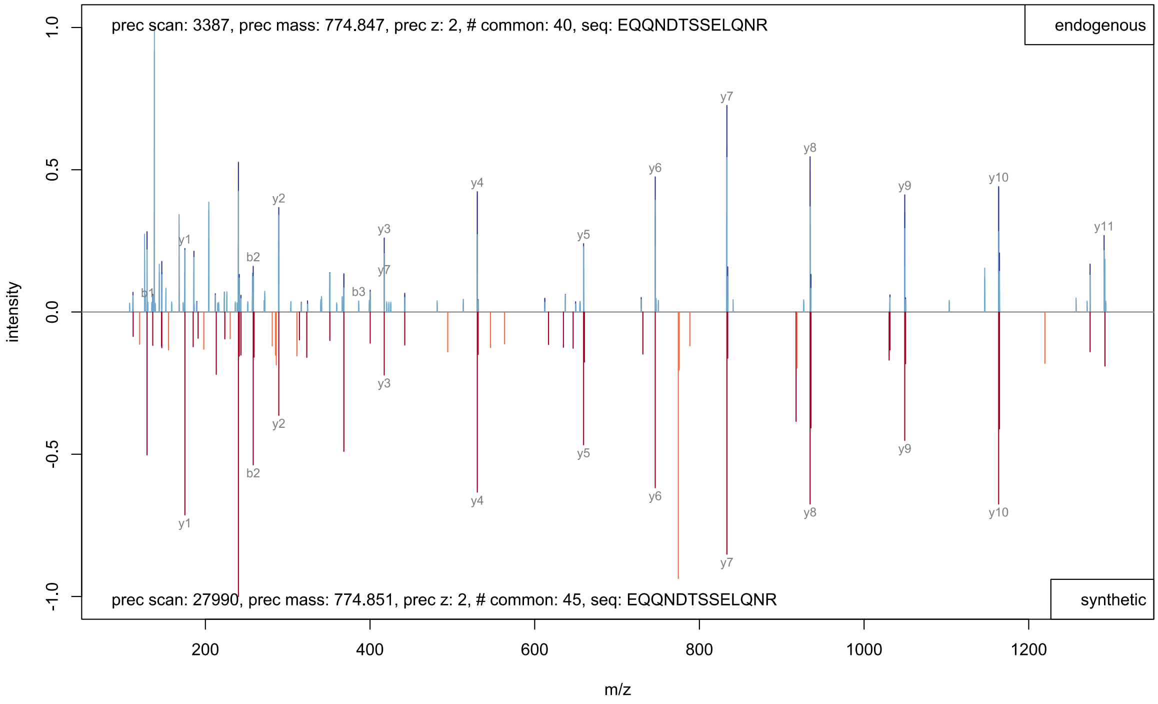

PCYOX1

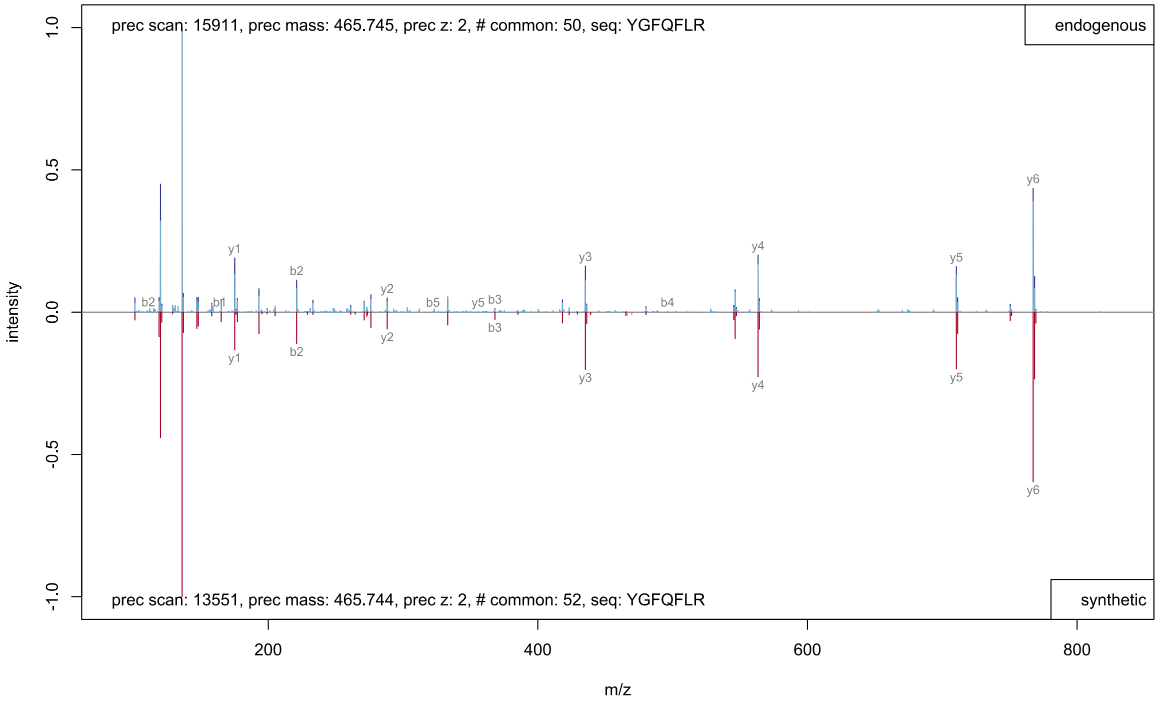

MPRIP

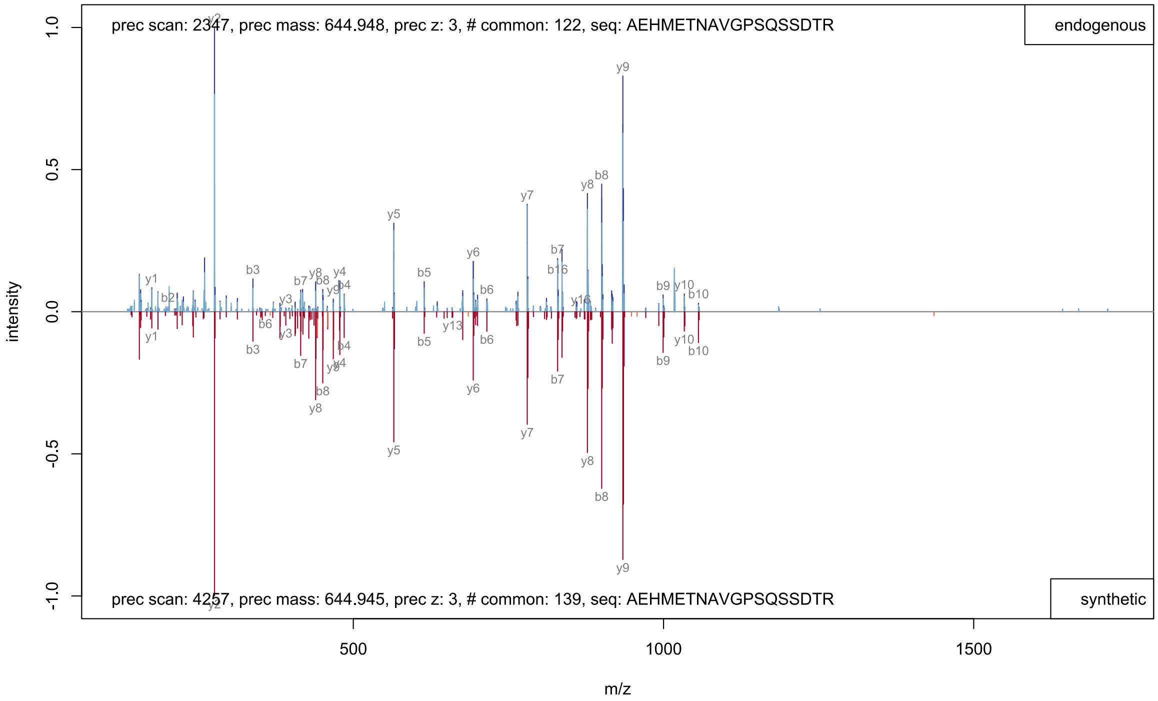

KRT7

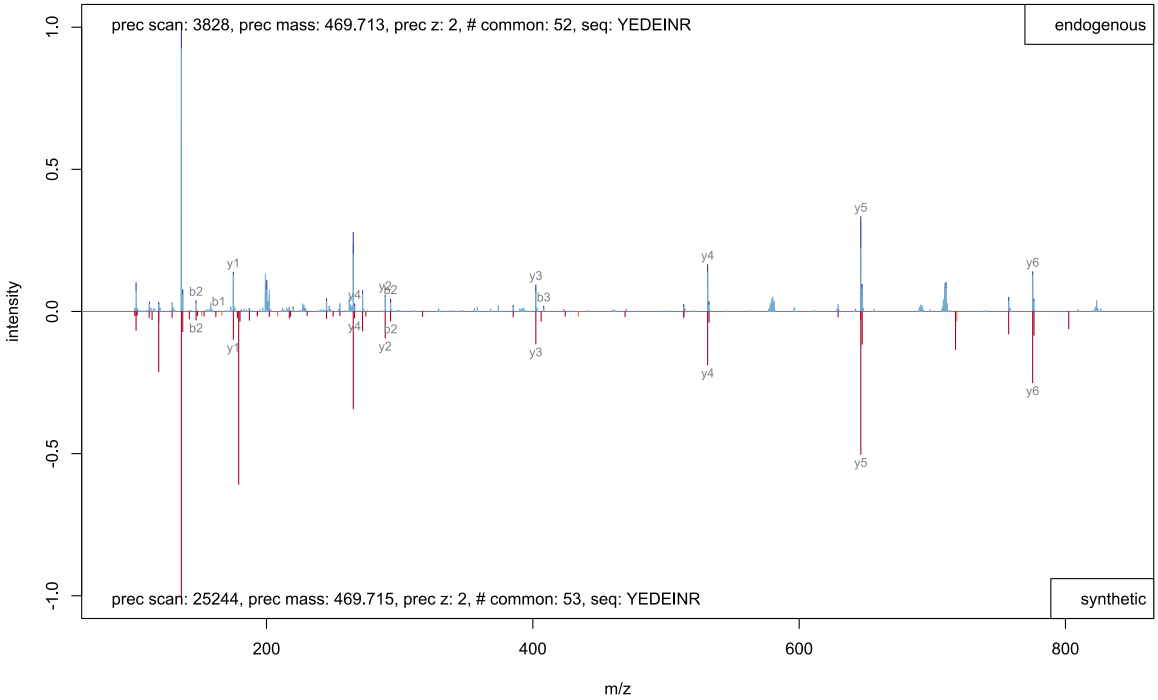

Brandsma C-A, *et al. Thorax* 2020; 75:180–183. doi: 10.1136/thoraxjnl-2019-213200

CYBRD1

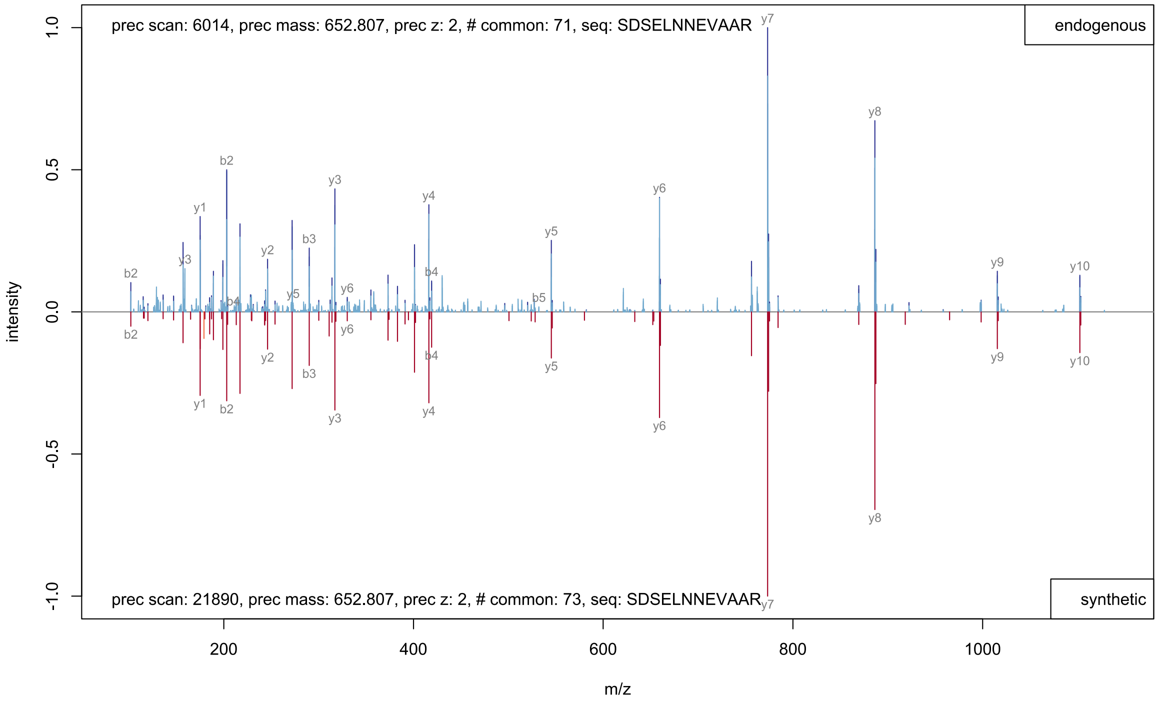

CAVIN2

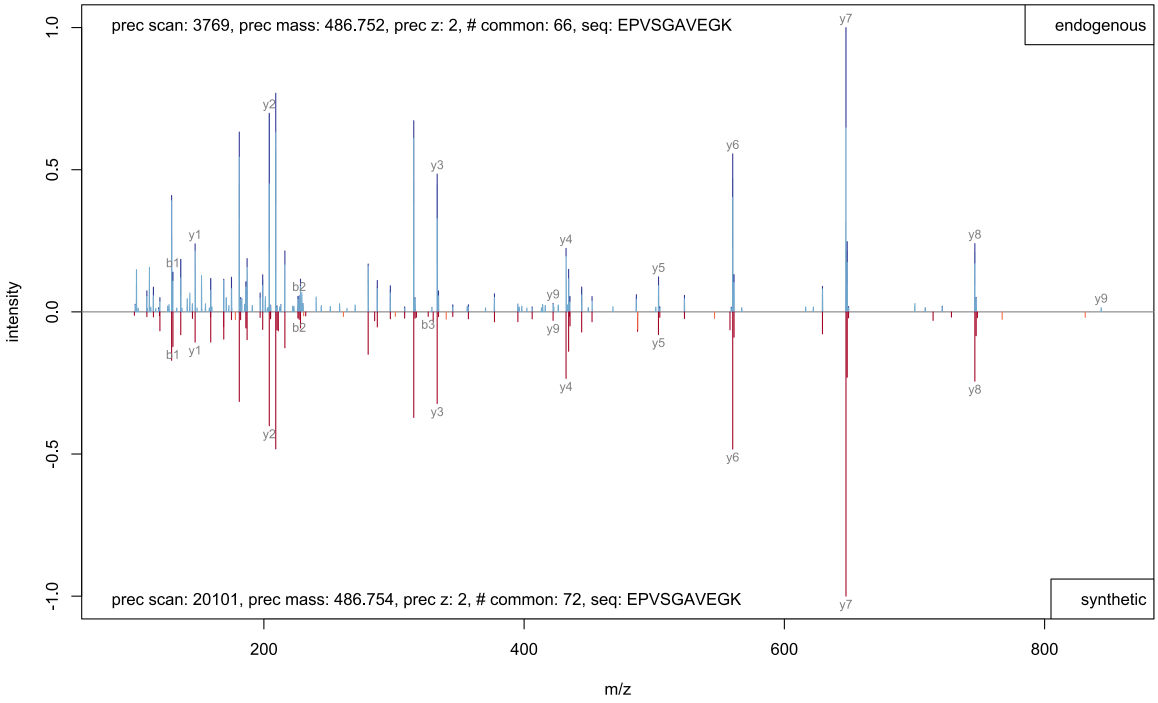

Brandsma C-A, *et al. Thorax* 2020; 75:180–183. doi: 10.1136/thoraxjnl-2019-213200

KRT7

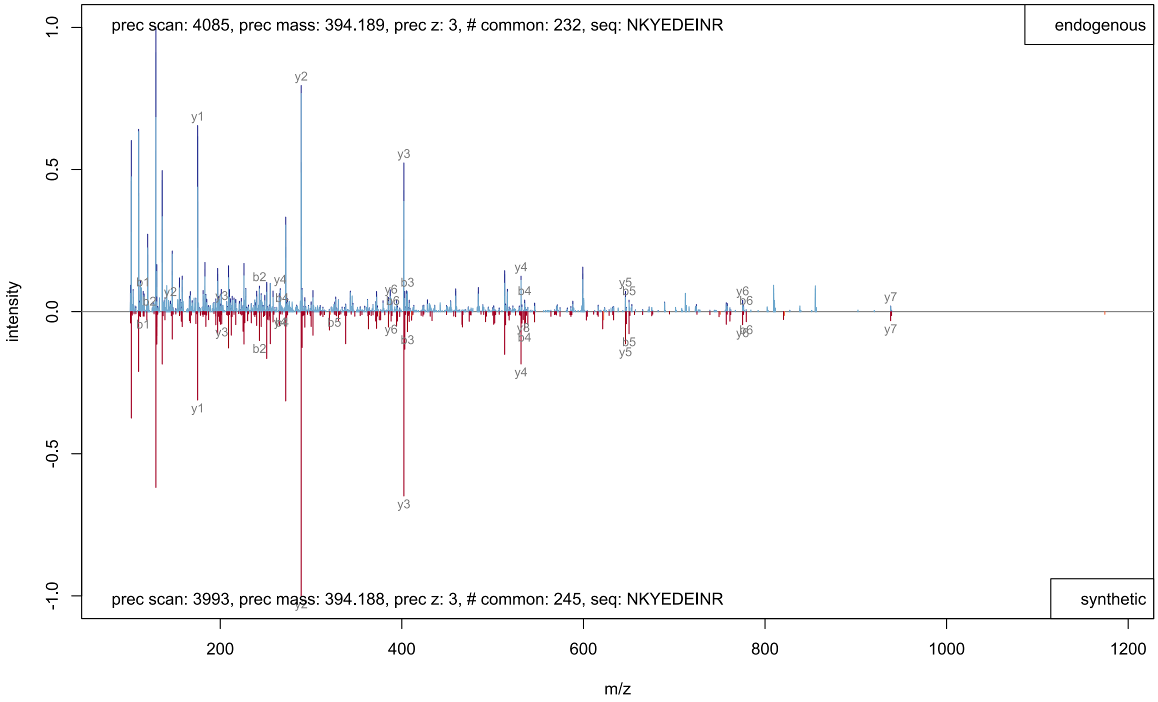
prec scan: 4085, prec mass: 394.189, prec z: 3, # common: 232, seq: NKYEDEINR
endogenous
prec scan: 3993, prec mass: 394.188, prec z: 3, # common: 245, seq: NKYEDEINR
synthetic
intensity
m/z

SORBS1

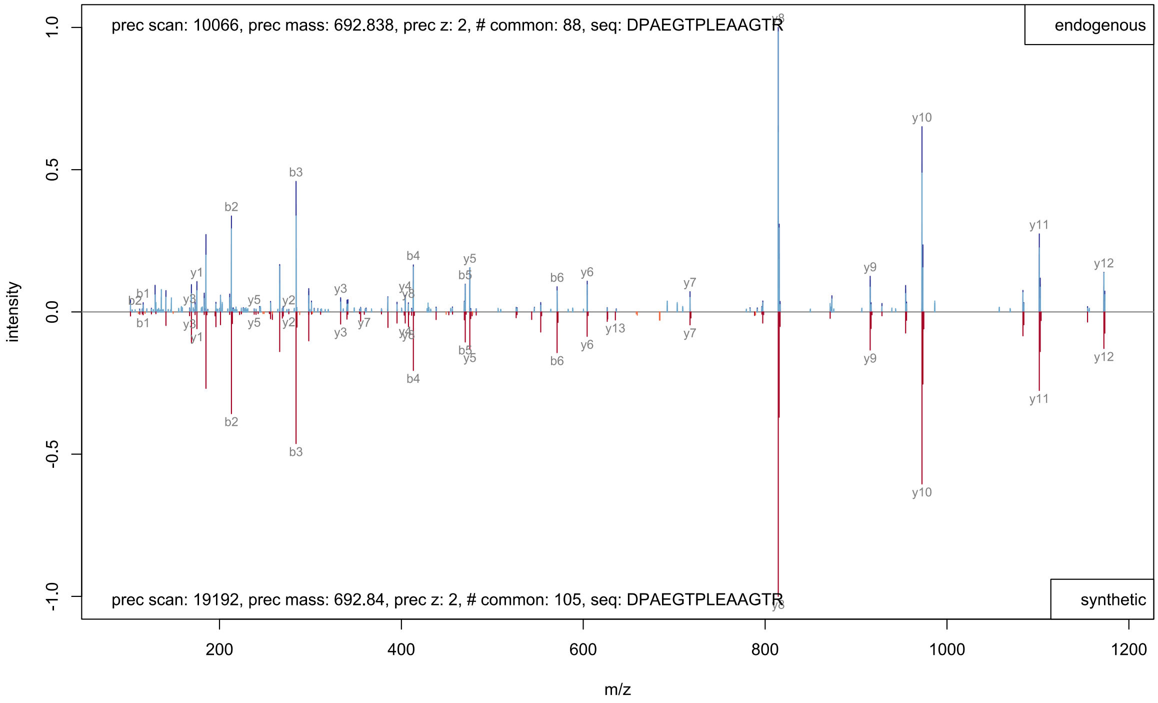
prec scan: 10066, prec mass: 692.838, prec z: 2, # common: 88, seq: DPAEGTPLEAAGTR
endogenous
prec scan: 19192, prec mass: 692.84, prec z: 2, # common: 105, seq: DPAEGTPLEAAGTR
synthetic
intensity
m/z

AKAP12

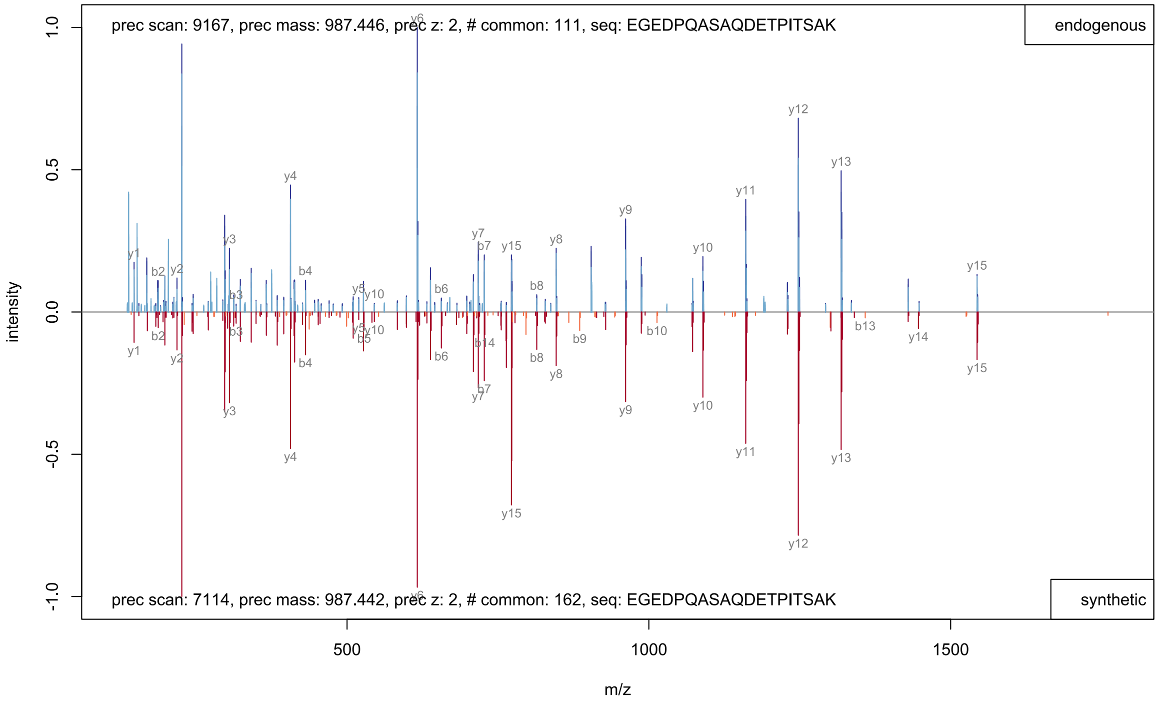

HCLS1

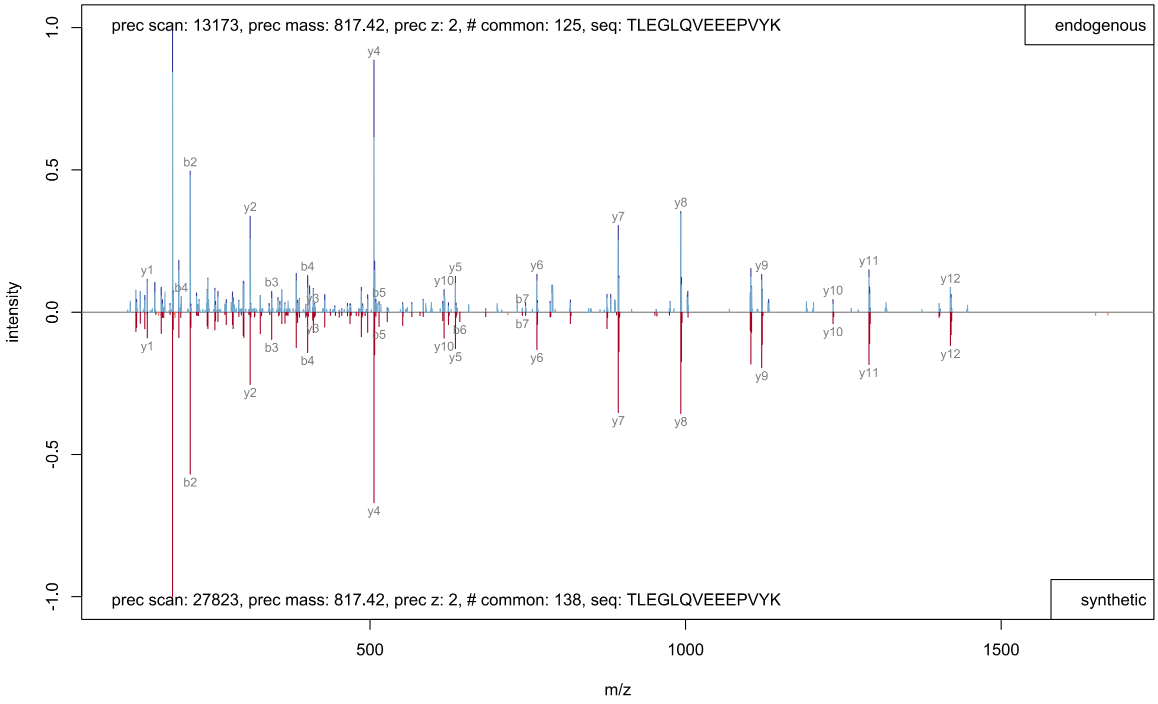

Brandsma C-A, *et al. Thorax* 2020; 75:180–183. doi: 10.1136/thoraxjnl-2019-213200

THRAP3

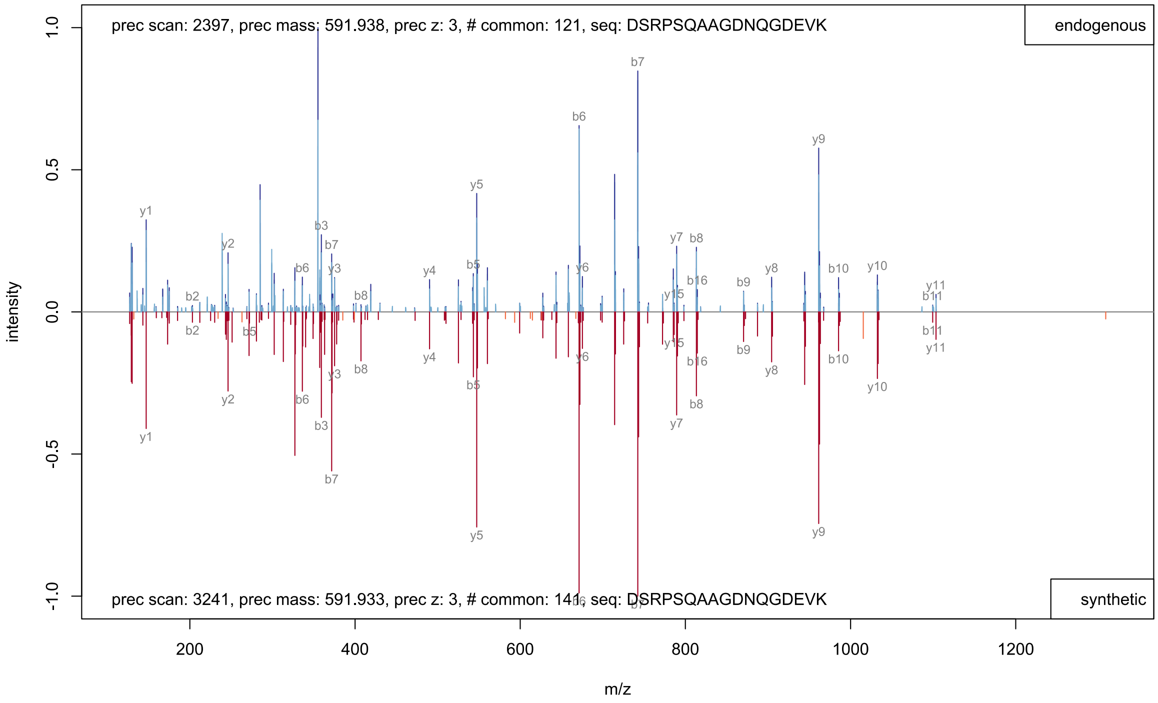
prec scan: 2397, prec mass: 591.938, prec z: 3, # common: 121, seq: DSRPSQAAGDNQGDEVK
endogenous
prec scan: 3241, prec mass: 591.933, prec z: 3, # common: 141, seq: DSRPSQAAGDNQGDEVK
synthetic
intensity
m/z

RNH1

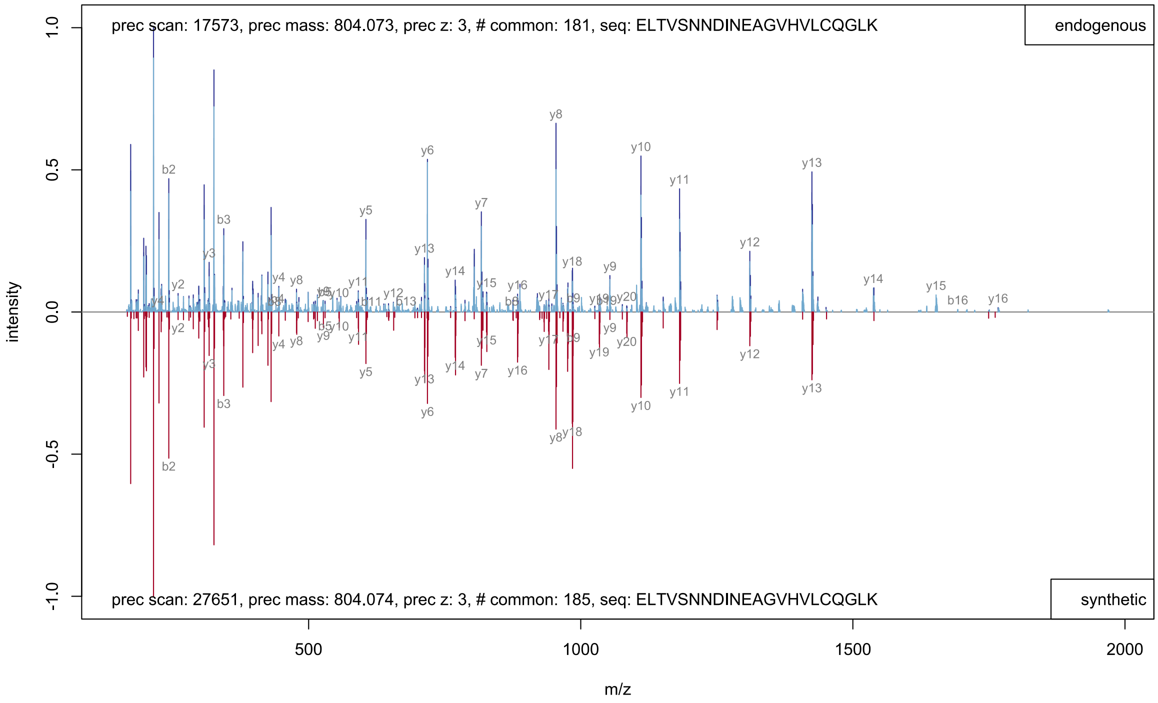
prec scan: 17573, prec mass: 804.073, prec z: 3, # common: 181, seq: ELTVSNNDINEAGVHVLCQGLK
endogenous
prec scan: 27651, prec mass: 804.074, prec z: 3, # common: 185, seq: ELTVSNNDINEAGVHVLCQGLK
synthetic
intensity
m/z

SWAP70

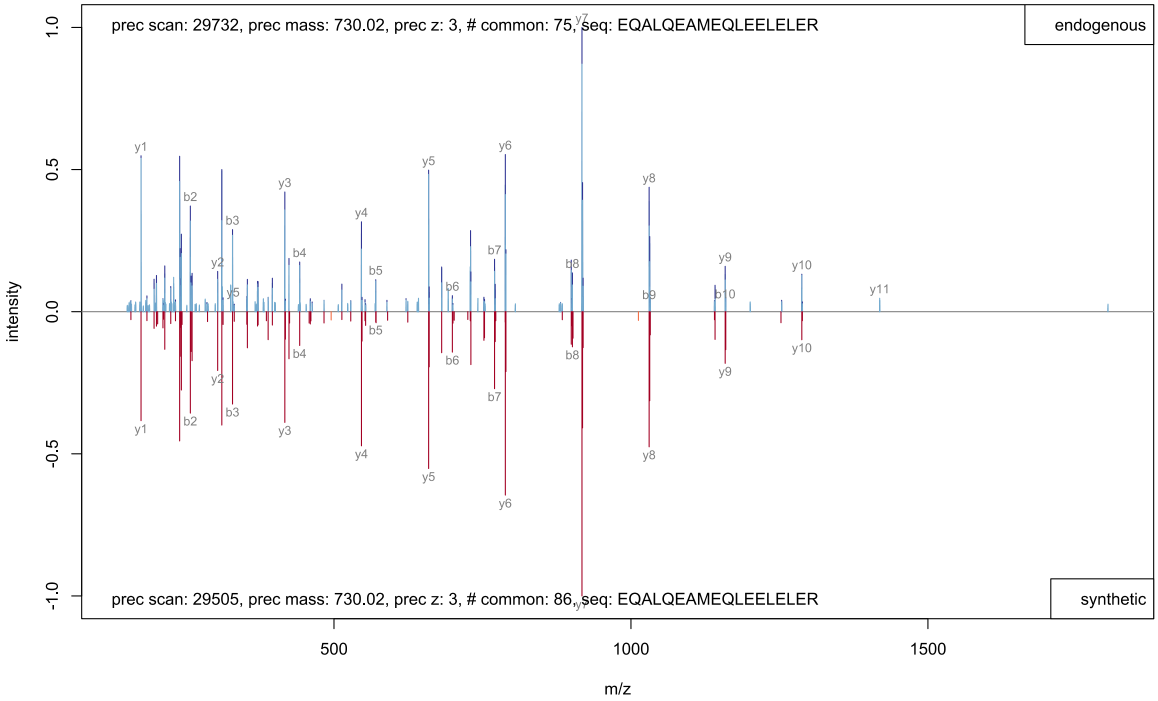
prec scan: 29732, prec mass: 730.02, prec z: 3, # common: 75, seq: EQALQEAMEQLEELELER
endogenous
prec scan: 29505, prec mass: 730.02, prec z: 3, # common: 86, seq: EQALQEAMEQLEELELER
synthetic
intensity
m/z
1.0
0.5
0.0
-0.5
-1.0
500
1000
1500
y1
b2
b3
y2
y3
b4
y4
b5
y5
b6
y6
b7
y7
b8
y8
b9
y9
b10
y10
y11
