# Supplementary material for: Integrated proteogenomic approach identifying a protein signature of COPD and a new splice variant of SORBS1
Source: Thorax. 2020 Jan 14;75(2):180–3. doi: 10.1136/thoraxjnl-2019-213200 (PMC7029225; doi:10.1136/thoraxjnl-2019-213200)
Supplement: Supplementary data [file thoraxjnl-2019-213200supp002.pdf]

**Column name**	**description**
SpectraFile	path and file name of mzML file contaning the assessed peptide spectra
ScanNum	scan number of assessed spectra
Peptide	sequence of assessed peptide with PTM
sub_pos	position of the altered amino acid
status	status indicating if the spectra was evaluated (checked) or not (skipped) by SpectrumAI
Sequence	sequence of assessed peptide without PTM
peptide_length	length of the assessed peptide
ions_support	boolean indicator for presence (YES) or absence (NO) of ion fragments contaning the altered amino acid
support_ions	list ion fragments contaning the altered amino acid
sum.supportions.intensity	sum of the intensity of the ion fragments containing the altered amino acid
flanking_ions_support	boolean indicator for presence (YES) or absence (NO) of flanking ion fragments neighboring the altered amino acid
flanking_ions	list of flanking ion fragments neighboring the altered amino acid
sum.flanking.ions.intensity	sum of the intensity of the flanking ion fragments neighboring the altered amino acid
matched_ions	list of fragment ions matched in the MS/MS spectra
sum.matchedions.intensity	sum of the intensity of all fragment ions matched in the MS/MS spectra
sum.fragmentions.intensity	sum of the intensity of all fragment ions in the MS/MS spectra
maxintensity	maximal fragment ion intensity
average_intensity	average fragment ion intensity in the MS/MS spectra
median_intensity	medium fragment ion intensity in the MS/MS spectra

SpectraFile	ScanNum	Peptide	sub_pos	status	Sequence	peptide_length	ions_support	support_ions	sum.supportions.intensity	flanking_ions_support	flanking_ions	sum.flanking.ions.intensity	matched_ions	sum.matchedions.intensity	sum.fragmentions.intensity	maxintensity	average_intensity	median_intensity
f:\data\MSData\2015\COPD_25cm_1D\mzML\150311_3_03.mzML	26144	ISYGPDWKDFYVVEPLAFEGTPEQK	12	checked	ISYGPDWKDFYVVEPLAFEGTPEQK	25	YES	b12,y14,	9160.64636	YES	b11,y14,b12,y13	27973.74451	b2,b4,b6,b7,b8,b9,b10,b11,b12,y2,y3,y4,y5,y6,y7,y8,y9,y10,y12,y13,y14	205287.75598	3420939.20965	44857.75000	641.58650	0
e:\data\MSData\Lund\MYakovleva\COPD_50cm_1D\mzML\150210_9_01.mzML	12943	QTLEKENTDLAGELR	8	checked	QTLEKENTDLAGELR	15	YES	b9,y8,y9,y10,y11,	28320.92053	YES	b7,y8,y7	32680.87012	b1,b2,b3,b4,b5,b6,b7,b9,y1,y2,y3,y4,y5,y6,y7,y8,y9,y10,y11	349407.68152	8627742.11253	128488.08594	1131.35879	0
e:\data\MSData\Lund\MYakovleva\COPD_25cm_2D\mzML\150330_3_100mM_01.mzML	12086	ASQSVSSNYLAWYQQKPGQAPR	8	checked	ASQSVSSNYLAWYQQKPGQAPR	22	YES	b8,b9,b11,	2309.28577	YES	b7,b8	2043.15613	b3,b4,b5,b7,b8,b9,b11,y1,y2,y3,y4,y5,y6,y7,y8,y9,y10,y11,y13	822889.64294	14480082.91033	267088.15625	1563.38619	0
e:\data\MSData\Lund\MYakovleva\COPD_50cm_1D\mzML\150210_3_01.mzML	27083	GGGAGFISGLTYLELDNPAGNK	11	checked	GGGAGFISGLTYLELDNPAGNK	22	YES	b11,y12,y13,y14,y15,y16,	52893.24579	YES	y12,b11,y11	27326.19159	b3,b4,b5,b6,b7,b8,b9,b11,y2,y3,y4,y5,y6,y7,y8,y9,y10,y11,y12,y13,y14,y15,y16	251771.72107	3608265.01507	44207.86719	705.15244	0
f:\data\MSData\2015\COPD_25cm_1D\mzML\150225_2_01.mzML	18493	LLIYWASAR	8	checked	LLIYWASAR	9	YES	y2,y3,y4,y5,y6,y7,y8,	82701.63782	YES	y2,y1	15535.23376	b1,b2,b3,y1,y2,y3,y4,y5,y6,y7,y8	130071.42441	2292307.63383	132206.68750	980.03747	0
e:\data\MSData\Lund\MYakovleva\COPD_25cm_2D\mzML\150414_10_500mM_01.mzML	2122	LHSLTQAKEESEK	2	checked	LHSLTQAKEESEK	13	YES	b2,b3,b4,b5,b6,b7,	44069.24841	NO	b2,y11	34688.13922	b2,b3,b4,b5,b6,b7,y1,y2,y3,y4,y5,y6,y7,y8,y9,y11	116500.79218	2444440.04447	45985.42578	635.24949	0
f:\data\MSData\2015\COPD_25cm_1D\mzML\150225_8_01.mzML	7532	LLEDLR	0	skiped	LLEDLR	6	NO		0	NO		0	b1,b2,b4,y1,y2,y3,y4,y5	774611.05951	15437057.13767	1193327.50000	3020.35945	0
e:\data\MSData\Lund\MYakovleva\COPD_50cm_1D\mzML\150210_3_01.mzML	22081	NSLFLQMNSLR	4	checked	NSLFLQMNSLR	11	YES	b4,b5,b7,y8,y9,y10,	191427.00000	YES	b3,y8,b4,y7	427611.61328	b1,b2,b3,b4,b5,b7,y1,y2,y3,y4,y5,y6,y7,y8,y9,y10	881755.59814	16932385.47102	156364.82813	1445.72963	0
e:\data\MSData\Lund\MYakovleva\COPD_25cm_2D\mzML\150330_6_20mM_01.mzML	12448	ISNSAAYSGSVAPANSALGQTQPSDQDTLVQR	6	checked	ISNSAAYSGSVAPANSALGQTQPSDQDTLVQR	32	YES	b6,b7,b8,b9,b10,b11,b12,	22504.91760	YES	b5,b6	8842.66626	b3,b4,b5,b6,b7,b8,b9,b10,b11,b12,y2,y3,y4,y5,y6,y7,y8,y9,y10,y11,y12,y13,y14,y15,y16,y17,y20	370844.30737	5174999.91392	134664.82813	934.11551	0
e:\data\MSData\Lund\MYakovleva\COPD_25cm_2D\mzML\150326_12_500mM_01.mzML	4714	NVSSNPC+57.02HEAVGIK	0	skiped	NVSSNPCHEAVGIK	14	NO		0	NO		0	b1,b2,b3,b5,y1,y2,y3,y4,y5,y6,y7,y8,y9,y10	231492.83514	10829007.05520	138082.71875	921.46078	0
e:\data\MSData\Lund\MYakovleva\COPD_25cm_2D\mzML\150326_15_40mM_01.mzML	7488	ADSQDAGQETEKEGEDPQASAQDETPITSAK	16	checked	ADSQDAGQETEKEGEDPQASAQDETPITSAK	31	YES	b18,y16,y18,y19,	10019.61511	YES	y16,y15	42817.76611	b3,b4,b5,b6,b7,b8,b9,b10,b11,b12,b13,b14,b18,y3,y4,y5,y6,y7,y8,y9,y10,y11,y12,y13,y14,y15,y16,y18,y19	524919.01807	7621632.72509	92127.79688	1087.40658	0
e:\data\MSData\Lund\MYakovleva\COPD_25cm_2D\mzML\150424_18_60mM_01.mzML	2846	EASQGSSASSAPQSVK	13	checked	EASQGSSASSAPQSVK	16	YES	y5,y6,y7,y8,y9,y10,y11,y12,y14,	56476.95392	YES	y3	4414.00635	b2,b3,b11,y1,y2,y3,y5,y6,y7,y8,y9,y10,y11,y12,y14	68976.30707	679860.80769	20718.52344	473.44067	0
e:\data\MSData\Lund\MYakovleva\COPD_25cm_2D\mzML\150318_11_40mM_01.mzML	3387	EQQNDTSSELQNR	6	checked	EQQNDTSSELQNR	13	YES	y8,y9,y10,y11,	19230.81787	YES	y8,y7	15179.80518	b2,b3,y1,y2,y3,y4,y5,y6,y7,y8,y9,y10,y11	53623.62155	555844.54841	11939.18457	424.63296	0
e:\data\MSData\Lund\MYakovleva\COPD_25cm_2D\mzML\150505_20_100mM_01.mzML	15911	YGFQFLR	5	checked	YGFQFLR	7	YES	y3,y4,y5,y6,	342169.82031	YES	b4,y3,y2	76357.45380	b1,b2,b3,b4,y1,y2,y3,y4,y5,y6	473906.59070	7655160.30710	356793.96875	1724.91219	0
e:\data\MSData\Lund\MYakovleva\COPD_50cm_1D\mzML\150210_13_01.mzML	29106	TGQEALSQTTISWAPFQDTSEYIISC+57.02HPVGTDEEPLQFR	0	skiped	TGQEALSQTTISWAPFQDTSEYIISCHPVGTDEEPLQFR	39	NO		0	NO		0	b4,b5,b6,b7,b8,b9,b10,b11,b12,b14,b15,y2,y3,y5,y6,y7,y8,y9,y10,y11,y13,y15,y16,y17,y20	117926.96619	3005913.78626	40984.57813	653.45952	0
e:\data\MSData\Lund\MYakovleva\COPD_25cm_2D\mzML\150326_14_500mM_01.mzML	2347	AEHM+15.99ETNAVGPSQSSDTR	13	checked	AEHMETNAVGPSQSSDTR	18	YES	y6,y7,y8,y9,y10,	99088.17163	YES	y6,y5	26080.49121	b2,b3,b4,b5,b6,b7,b8,b9,b10,y1,y2,y3,y4,y5,y6,y7,y8,y9,y10	232370.64716	2252036.78392	53338.45703	689.96225	0
e:\data\MSData\Lund\MYakovleva\COPD_25cm_2D\mzML\150326_15_40mM_01.mzML	3828	YEDEINR	7	checked	YEDEINR	7	YES	y1,y2,y3,y4,y5,y6,	279658.45703	YES	y1	38967.22656	b1,b2,b3,y1,y2,y3,y4,y5,y6	299259.84753	7453859.75510	305118.43750	1651.64187	0
e:\data\MSData\Lund\MYakovleva\COPD_25cm_2D\mzML\150424_16_40mM_01.mzML	6014	SDSELNNEVAAR	7	checked	SDSELNNEVAAR	12	YES	y6,y7,y8,y9,y10,	261580.79785	YES	y6,y5	72925.87109	b2,b3,b4,b5,y1,y2,y3,y4,y5,y6,y7,y8,y9,y10	532078.36853	6353644.36970	111427.41406	1048.62921	0
e:\data\MSData\Lund\MYakovleva\COPD_25cm_2D\mzML\150505_20_100mM_01.mzML	3769	EPVSGAVEGK	1	checked	EPVSGAVEGK	10	YES	b2,	1664.21240	YES	y9	473.85800	b2,y1,y2,y3,y4,y5,y6,y7,y8,y9	112147.47739	1922640.38195	60020.94922	801.10016	0
e:\data\MSData\Lund\MYakovleva\COPD_25cm_2D\mzML\150326_14_500mM_01.mzML	4085	NKYEDEINR	9	checked	NKYEDEINR	9	YES	y1,y2,y3,y4,y5,y6,y7,	405269.73071	YES	y1	121365.79688	b1,b2,b3,b4,b5,b6,y1,y2,y3,y4,y5,y6,y7	454514.63501	18032757.23679	352745.93750	1446.78733	56.37574
e:\data\MSData\Lund\MYakovleva\COPD_25cm_2D\mzML\150424_18_40mM_01.mzML	10066	DPAEGTPLEAAGTR	0	skiped	DPAEGTPLEAAGTR	14	NO		0	NO		0	b2,b3,b4,b5,b6,y1,y2,y3,y4,y5,y6,y7,y8,y9,y10,y11,y12	192552.93436	1658047.21516	49731.89453	591.10418	0
e:\data\MSData\Lund\MYakovleva\COPD_25cm_2D\mzML\150518_15_20mM_03.mzML	9167	EGEDPQASAQDETPITSAK	4	checked	EGEDPQASAQDETPITSAK	19	YES	b4,b6,b7,b8,	7809.22681	YES	b3,b4	2580.99384	b2,b3,b4,b6,b7,b8,y1,y2,y3,y4,y5,y6,y7,y8,y9,y10,y11,y12,y13	94508.22205	1145280.52248	18506.37695	523.19805	0
e:\data\MSData\Lund\MYakovleva\COPD_25cm_2D\mzML\150429_17_40mM_01.mzML	13173	TLEGLQVEEEPVYK	14	checked	TLEGLQVEEEPVYK	14	YES	y1,y2,y3,y4,y5,y6,y7,y8,y9,y10,y11,	187544.99377	YES	y1	8358.89648	b2,b3,b4,b5,b7,y1,y2,y3,y4,y5,y6,y7,y8,y9,y10,y11	241436.69220	3465533.20655	72233.62500	788.33785	0
f:\data\MSData\2015\COPD_25cm_1D\mzML\150311_19_03.mzML	2397	DSRPSQAAGDNQGDEVK	16	checked	DSRPSQAAGDNQGDEVK	17	YES	y2,y3,y4,y5,y6,y7,y8,y9,y10,y11,	60030.32959	YES	y2,y1	15183.24219	b2,b3,b5,b6,b7,b8,b9,b10,b11,y1,y2,y3,y4,y5,y6,y7,y8,y9,y10,y11	136721.72168	1636891.56872	28518.27344	632.98205	0
f:\data\MSData\2015\COPD_25cm_1D\mzML\150311_15_03.mzML	17573	ELTVSNNDINEAGVHVLC+57.02QGLK	15	checked	ELTVSNNDINEAGVHVLCQGLK	22	YES	b16,y8,y9,y10,y11,y12,y13,y15,y16,	458004.09448	YES	y8,y7	181786.45313	b2,b3,b4,b5,b8,b9,b16,y2,y3,y4,y5,y6,y7,y8,y9,y10,y11,y12,y13,y15,y16	877857.25055	19649631.47159	178899.65625	1563.09215	0
f:\data\MSData\2015\COPD_25cm_1D\mzML\150311_19_03.mzML	29732	EQALQEAMEQLEELELER	13	checked	EQALQEAMEQLEELELER	18	YES	y6,y7,y8,y9,y10,y11,	42449.44098	YES	y6,y5	19153.50195	b2,b3,b4,b5,b6,b7,b8,b9,b10,y1,y2,y3,y4,y5,y6,y7,y8,y9,y10,y11	100688.58527	1022782.82420	18225.72070	489.60403	0
e:\data\MSData\Lund\MYakovleva\COPD_25cm_2D\mzML\150330_6_100mM_01.mzML	15436	GATYNIIVEALKDQQR	6	checked	GATYNIIVEALKDQQR	16	YES	b6,b7,y11,y12,y13,	29666.84058	YES	b5,y11,b6,y10	63380.91333	b2,b3,b4,b5,b6,b7,y1,y2,y3,y4,y5,y6,y7,y8,y9,y10,y11,y12,y13	212451.89850	1917098.69502	43826.80859	807.87977	0
e:\data\MSData\Lund\MYakovleva\COPD_25cm_2D\mzML\150330_3_100mM_01.mzML	12809	SDNC+57.02EDTPEAGYFAVAVVKK	15	checked	SDNCEDTPEAGYFAVAVVKK	20	YES	y6,y7,y8,y9,y10,y11,y12,y13,y15,	339269.45215	YES	y6,y5	86237.01563	b2,b3,b4,b5,b6,b7,b9,b10,b12,y2,y3,y4,y5,y6,y7,y8,y9,y10,y11,y12,y13,y15	636406.50806	11237608.52917	162286.06250	1327.53792	0
f:\data\MSData\2015\COPD_25cm_1D\mzML\150311_10_03.mzML	26054	VMQVVDEKLPGLLGNFPGPFEEEMK	1	checked	VMQVVDEKLPGLLGNFPGPFEEEMK	25	YES	b2,b3,b4,b5,b6,b7,b9,b11,b12,b13,	68621.99310	NO		0	b2,b3,b4,b5,b6,b7,b9,b11,b12,b13,y2,y3,y4,y5,y6,y7,y8,y9,y10,y11,y12,y13,y15,y16	208391.83459	3302294.90726	69030.42969	609.72949	0
f:\data\MSData\2015\COPD_25cm_1D\mzML\150311_3_03.mzML	28282	AVDQSVLLMKPDAELSASSVYNLLPEKDLTGFPGPLNDQDDEDC+57.02INR	41	checked	AVDQSVLLMKPDAELSASSVYNLLPEKDLTGFPGPLNDQDDEDCINR	47	YES	y7,y8,y9,y10,y11,y12,y13,y14,y16,y18,y19,y22,	326572.97644	YES	y7,y6	85466.26953	b4,b5,b6,b7,b8,b9,b10,b12,b13,b14,b15,b17,b18,b19,b22,y3,y4,y5,y6,y7,y8,y9,y10,y11,y12,y13,y14,y16,y18,y19,y22	671969.83875	18003983.37546	164016.51563	1687.82070	0
e:\data\MSData\Lund\MYakovleva\COPD_25cm_2D\mzML\150330_3_500mM_01.mzML	3190	DSEDVSERDSDKEMATK	7	checked	DSEDVSERDSDKEMATK	17	YES	b8,b9,b10,b11,b12,b13,y12,	21001.29926	NO		0	b2,b3,b4,b5,b8,b9,b10,b11,b12,b13,y1,y2,y3,y4,y5,y6,y7,y8,y9,y12	216259.08850	5909589.83078	69873.35156	733.92820	0
e:\data\MSData\Lund\MYakovleva\COPD_25cm_2D\mzML\150330_4_40mM_01.mzML	12235	EVQLVESGGGVVQPGGSLR	11	checked	EVQLVESGGGVVQPGGSLR	19	YES	b11,b12,b13,b16,y9,y10,y11,y12,y13,y14,y15,y16,y17,	454291.62881	YES	b10,y9,b11,y8	62220.49365	b1,b2,b3,b4,b5,b6,b7,b9,b10,b11,b12,b13,b16,y1,y2,y3,y4,y5,y6,y7,y8,y9,y10,y11,y12,y13,y14,y15,y16,y17	886991.16837	8598612.53354	134026.39063	1553.21758	0
e:\data\MSData\Lund\MYakovleva\COPD_25cm_2D\mzML\150422_2_100mM_01.mzML	5632	SYPASDHTTPLSR	6	checked	SYPASDHTTPLSR	13	YES	y8,y9,y10,	39873.89941	YES	b5,y8,y7	48029.08398	b2,b5,y1,y2,y3,y4,y5,y6,y7,y8,y9,y10	375826.35254	12690407.51900	306747.43750	1314.25099	0
e:\data\MSData\Lund\MYakovleva\COPD_50cm_1D\mzML\150210_2_01.mzML	21200	ADIDVSGPKVDVDVPDVNIEGPDAK	0	skiped	ADIDVSGPKVDVDVPDVNIEGPDAK	25	NO		0	NO		0	b2,b3,b4,b5,b6,b7,b8,b9,b10,b11,b12,b13,b14,y2,y3,y4,y5,y6,y7,y8,y9,y10,y11,y12,y13,y14,y15	1739910.95715	25067476.16843	287588.78125	2390.56610	2.17051
e:\data\MSData\Lund\MYakovleva\COPD_25cm_2D\mzML\150422_8_500mM_01.mzML	14520	SAVQGPPDRDLC+57.02GC+57.02YSVSSVLPGC+57.02AEPWNHGK	8	checked	SAVQGPPDRDLCGCYSVSSVLPGCAEPWNHGK	32	YES	b9,b10,b11,b13,b14,b15,b18,b19,	161269.00684	YES		0	b3,b4,b5,b6,b9,b10,b11,b13,b14,b15,b18,b19,y3,y4,y5,y6,y7,y8,y9,y10,y11,y12	1095578.06848	21228735.74678	408509.15625	1714.89908	0
f:\data\MSData\2015\COPD_25cm_1D\mzML\150311_10_03.mzML	11218	M+15.99HKSSMPEEVK	0	skiped	MHKSSMPEEVK	11	NO		0	NO		0	b7,b8,y1,y2,y3,y4,y5,y6,y7	769877.96606	36542534.32770	460765.81250	2880.99451	69.90292
f:\data\MSData\2015\COPD_25cm_1D\mzML\150311_9_03.mzML	26504	SVFKPFIFGVGVAQAPQVLSPTFGAQDPVR	10	checked	SVFKPFIFGVGVAQAPQVLSPTFGAQDPVR	30	YES	b10,b11,b12,b13,b14,	28326.42822	YES	b9,b10	15717.94775	b3,b4,b5,b6,b7,b8,b9,b10,b11,b12,b13,b14,y3,y4,y5,y6,y7,y8,y9,y10,y11,y12,y13	288342.45166	4370130.28712	59883.81641	938.40032	0
e:\data\MSData\Lund\MYakovleva\COPD_50cm_1D\mzML\150210_20_01.mzML	22140	AAQAQGQSC+57.02EYSLMVGYQC+57.02GQVFR	24	checked	AAQAQGQSCEYSLMVGYQCGQVFR	24	YES	y2,y3,y4,y5,y6,y7,y8,y9,y10,y11,y12,y13,	290416.62598	NO		0	b3,b4,b5,b6,b7,b8,b9,b10,b11,b12,y2,y3,y4,y5,y6,y7,y8,y9,y10,y11,y12,y13	373436.90918	3899114.89459	97193.56250	901.32106	0
f:\data\MSData\2015\COPD_25cm_1D\mzML\150303_8_02.mzML	12317	LLIYDASTR	8	checked	LLIYDASTR	9	YES	y2,y3,y4,y5,y6,y7,y8,	1807302.33984	YES	y2,y1	196386.56641	b1,b2,b3,y1,y2,y3,y4,y5,y6,y7,y8	2682150.35632	40163528.13304	2247297.75000	4583.30801	0
e:\data\MSData\Lund\MYakovleva\COPD_25cm_2D\mzML\150422_8_60mM_01.mzML	9811	LLIYDTSNR	6	checked	LLIYDTSNR	9	YES	y4,y5,y6,y7,y8,	371939.49023	YES	y4,y3	106855.98828	b2,b3,y1,y2,y3,y4,y5,y6,y7,y8	526918.31738	6563569.07134	283638.43750	1576.26539	0
f:\data\MSData\2015\COPD_25cm_1D\mzML\150225_4_01.mzML	24113	TPEVTC+57.02VVVDVSHEDPEVQFNWYVDGMEVHNAK	27	checked	TPEVTCVVVDVSHEDPEVQFNWYVDGMEVHNAK	33	YES	y8,y9,y10,y11,y12,y13,	33430.72107	NO	y6	1075.40503	b3,b4,b5,b6,b7,b8,b10,b11,y3,y4,y5,y6,y8,y9,y10,y11,y12,y13	72863.52744	688743.63556	26847.66602	495.85575	0
f:\data\MSData\2015\COPD_25cm_1D\mzML\150311_10_03.mzML	26060	DAMWIGFLTR	2	checked	DAMWIGFLTR	10	YES	b2,b3,b4,b5,b6,	327177.56946	NO	b2,y8	194450.03906	b2,b3,b4,b5,b6,y1,y2,y3,y4,y5,y6,y7,y8	1602948.79993	22853786.80047	459811.84375	2409.72024	0
e:\data\MSData\Lund\MYakovleva\COPD_50cm_1D\mzML\150210_2_01.mzML	20195	LLISWASTR	4	checked	LLISWASTR	9	YES	b4,b6,y6,y7,y8,	259908.36505	YES	b3,y6,b4,y5	180914.74469	b2,b3,b4,b6,y1,y2,y3,y4,y5,y6,y7,y8	509321.62067	10015870.29467	529581.75000	1652.51119	0
e:\data\MSData\Lund\MYakovleva\COPD_25cm_2D\mzML\150414_10_500mM_01.mzML	9338	LLIYDNDKRPSGIPDR	7	checked	LLIYDNDKRPSGIPDR	16	YES	y10,	0	YES	y10	0	b2,b3,y1,y2,y3,y4,y5,y6,y7,y10	480662.90552	28631040.09746	304775.56250	2070.36229	0
f:\data\MSData\2015\COPD_25cm_1D\mzML\150225_20_01.mzML	4978	TYYADSVK	0	skiped	TYYADSVK	8	NO		0	NO		0	b1,b2,y1,y2,y3,y4,y5,y6,y7	69215.39526	2726136.13435	63551.49609	860.52277	0
e:\data\MSData\Lund\MYakovleva\COPD_25cm_2D\mzML\150422_2_60mM_01.mzML	4387	GTPVTVSSASTK	3	checked	GTPVTVSSASTK	12	YES	b3,b4,b5,b7,y10,	68774.10229	YES	b2,y10,b3,y9	176548.39648	b2,b3,b4,b5,b7,y1,y2,y3,y4,y5,y6,y7,y8,y9,y10	925954.97729	12487840.38769	365798.53125	1829.18418	0
e:\data\MSData\Lund\MYakovleva\COPD_25cm_2D\mzML\150422_8_40mM_01.mzML	13674	EQPGSPEWIQLDK	13	checked	EQPGSPEWIQLDK	13	YES	y1,y2,y3,y4,y5,y6,y7,y8,y9,y10,y11,	86301.03699	YES	y1	4197.57227	b2,y1,y2,y3,y4,y5,y6,y7,y8,y9,y10,y11	96028.29285	1811869.13118	21775.48047	622.63544	0
e:\data\MSData\Lund\MYakovleva\COPD_25cm_2D\mzML\150330_6_40mM_01.mzML	10897	SMPIPIPK	5	checked	SMPIPIPK	8	YES	b5,b6,y4,y5,y6,	435782.90112	YES	y4,b5,y3	349113.07727	b1,b2,b3,b5,b6,y1,y2,y3,y4,y5,y6	1371065.82544	23722129.92244	836100.87500	4145.05153	0
f:\data\MSData\2015\COPD_25cm_1D\mzML\150225_19_01.mzML	6263	ASTLESGVPSR	3	checked	ASTLESGVPSR	11	YES	b3,b4,b5,b9,y9,y10,	54425.57611	YES	b2,y9,b3,y8	119343.66602	b2,b3,b4,b5,b9,y1,y2,y3,y4,y5,y6,y7,y8,y9,y10	847779.37982	13490248.19814	231123.31250	1714.13573	0
e:\data\MSData\Lund\MYakovleva\COPD_50cm_1D\mzML\150210_15_01.mzML	11427	FIEDVK	0	skiped	FIEDVK	6	NO		0	NO		0	b1,b2,b3,b4,b5,y1,y2,y3,y4,y5	879704.09277	20933065.25139	1353677.50000	3981.18396	0
e:\data\MSData\Lund\MYakovleva\COPD_25cm_2D\mzML\150326_13_20mM_01.mzML	17358	DLYTNTVLSGGTTMYPGIADR	0	skiped	DLYTNTVLSGGTTMYPGIADR	21	NO		0	NO		0	b2,b3,y1,y2,y3,y4,y5,y6,y7,y8,y9,y10,y11,y12,y13	444982.83569	6226779.72095	161565.56250	805.22174	0
e:\data\MSData\Lund\MYakovleva\COPD_25cm_2D\mzML\150429_16_500mM_01.mzML	6619	VDNDEDEHQLSLR	0	skiped	VDNDEDEHQLSLR	13	NO		0	NO		0	b2,b3,b5,b6,y1,y2,y3,y4,y5,y6,y7,y8,y9,y10	376351.15479	10292088.63003	173802.95313	1038.66068	0
f:\data\MSData\2015\COPD_25cm_1D\mzML\150311_12_03.mzML	22577	VGEAGLLSVNC+57.02SEAGPGALGLEAVSDSGTK	10	checked	VGEAGLLSVNCSEAGPGALGLEAVSDSGTK	30	YES	b10,b11,b13,b14,b15,b18,	11471.69849	YES	b9,b10	5668.51514	b3,b4,b5,b6,b7,b8,b9,b10,b11,b13,b14,b15,b18,y2,y3,y4,y5,y6,y7,y8,y9,y10,y11,y12,y13,y14,y16,y17	235503.09125	2452258.75117	50921.41797	647.20474	0
e:\data\MSData\Lund\MYakovleva\COPD_50cm_1D\mzML\150210_20_01.mzML	33137	NALTGLPSGLFQASATLDTLVLK	8	checked	NALTGLPSGLFQASATLDTLVLK	23	YES	b9,b10,b11,y16,y17,	22536.27271	YES	b7,y16,y15	1887.54498	b2,b3,b4,b5,b6,b7,b9,b10,b11,y2,y3,y4,y5,y6,y7,y8,y9,y10,y11,y12,y13,y15,y16,y17	395160.74298	12978652.77335	46369.51563	1143.39290	213.34375
e:\data\MSData\Lund\MYakovleva\COPD_25cm_2D\mzML\150505_17_100mM_01.mzML	3034	AEAEDTSKDPVGR	7	checked	AEAEDTSKDPVGR	13	YES	b9,y7,y8,y9,y10,y11,	7973.38379	YES	y7,y6	2354.74414	b2,b3,b9,y1,y2,y4,y5,y6,y7,y8,y9,y10,y11	30541.63605	227458.64565	12997.48047	272.40556	0
e:\data\MSData\Lund\MYakovleva\COPD_25cm_2D\mzML\150505_17_500mM_01.mzML	4279	NKYEDEINRR	9	checked	NKYEDEINRR	10	YES	y2,y3,y4,y5,y6,y7,y8,	144690.29077	YES	y2,y1	335325.01563	b1,b2,b3,b4,b5,b6,b7,y1,y2,y3,y4,y5,y6,y7,y8	606225.38501	81109997.63183	1554019.50000	5257.32419	0
e:\data\MSData\Lund\MYakovleva\COPD_25cm_2D\mzML\150326_13_500mM_01.mzML	2117	AEDEENEKETAVSTEDDSHHK	3	checked	AEDEENEKETAVSTEDDSHHK	21	YES	b3,b4,b7,b9,b10,b11,	6487.33759	YES	b2,b3	5996.15820	b2,b3,b4,b7,b9,b10,b11,y2,y3,y4,y5,y6,y7,y8,y9,y10,y12	31176.11700	263969.42689	6460.60352	289.12314	0
e:\data\MSData\Lund\MYakovleva\COPD_25cm_2D\mzML\150422_8_500mM_01.mzML	6280	VSYGIGEEEHDQEGR	7	checked	VSYGIGEEEHDQEGR	15	YES	b9,y9,y10,y11,y12,	256809.13513	YES	b6,y9,y8	115246.22348	b2,b4,b5,b6,b9,y1,y2,y3,y4,y5,y6,y7,y8,y9,y10,y11,y12	1057791.30685	26287136.32600	377371.37500	1736.15589	0
e:\data\MSData\Lund\MYakovleva\COPD_25cm_2D\mzML\150429_16_100mM_01.mzML	13517	VLVPSLLHTETTEK	0	skiped	VLVPSLLHTETTEK	14	NO		0	NO		0	b2,b3,b9,y1,y2,y3,y4,y5,y6,y7,y8,y9,y10,y11	157999.79633	3042366.63454	70700.39844	690.50536	0
e:\data\MSData\Lund\MYakovleva\COPD_50cm_1D\mzML\150210_18_01.mzML	23536	GATYNIIVEALK	6	checked	GATYNIIVEALK	12	YES	b6,b7,y7,y8,y9,y10,	97304.38531	YES	b5,y7,b6,y6	118917.76416	b2,b3,b4,b5,b6,b7,y1,y2,y3,y4,y5,y6,y7,y8,y9,y10	460877.53925	8603739.34018	134686.51563	1261.73036	0
f:\data\MSData\2015\COPD_25cm_1D\mzML\150225_19_01.mzML	20611	DLTGFPGPLNDQDDEDC+57.02INR	14	checked	DLTGFPGPLNDQDDEDCINR	20	YES	y7,y8,y9,y11,y12,y13,y14,y16,y17,	227925.23511	YES	y7,y6	82993.81250	b2,b3,b4,b5,b6,b7,y1,y2,y3,y4,y5,y6,y7,y8,y9,y11,y12,y13,y14,y16,y17	501797.17682	8911711.95358	125422.37500	1665.74055	0
