# Supplementary material for: Integrated proteogenomic approach identifying a protein signature of COPD and a new splice variant of SORBS1
Source: Thorax. 2020 Jan 14;75(2):180–3. doi: 10.1136/thoraxjnl-2019-213200 (PMC7029225; doi:10.1136/thoraxjnl-2019-213200)
Supplement: Supplementary data [file thoraxjnl-2019-213200supp003.pdf]

List of differential proteins and transcript

sheets	Description
DE proteins comb FDR 0.05 up	Differential proteins, which increased and has FDR p-value < 0.05
DE proteins comb FDR 0.05 down	Differential proteins, which decreased and has FDR p-value < 0.05
DE mRNA comb FDR 0.05 up	Differential mRNA which increased and has FDR p-value < 0.05
DE mRNA comb FDR 0.05 down	Differential mRNA, which decreased and has FDR p-value < 0.05

Brandsma C-A, *et al. Thorax* 2020; 75:180–183. doi: 10.1136/thoraxjnl-2019-213200

DE proteins comb FDR 0.05 up

Ensembl gene	logFC	logCPM	LR	PValue	adj.p	gene	geneDescription
ENSG00000128595	1.19317	9.40518	58.38251	2.16E-14	3.72E-11	CALU	calumenin [Source:HGNC Symbol;Acc:HGNC:1458]
ENSG00000173267	1.89795	8.30998	43.90399	3.45E-11	1.14E-08	SNCG	synuclein gamma [Source:HGNC Symbol;Acc:HGNC:11141]
ENSG00000149428	0.98218	9.92682	43.63452	3.96E-11	1.14E-08	HYOU1	hypoxia up-regulated 1 [Source:HGNC Symbol;Acc:HGNC:16931]
ENSG00000162735	3.60491	7.03458	42.27054	7.95E-11	1.96E-08	PEX19	peroxisomal biogenesis factor 19 [Source:HGNC Symbol;Acc:HGNC:9713]
ENSG00000127022	1.87779	8.75570	39.30611	3.62E-10	6.94E-08	CANX	calnexin [Source:HGNC Symbol;Acc:HGNC:1473]
ENSG00000163431	3.30390	7.38293	36.48381	1.54E-09	2.41E-07	LMOD1	leiomodin 1 [Source:HGNC Symbol;Acc:HGNC:6647]
ENSG00000143420	2.39618	7.32907	35.70016	2.30E-09	3.11E-07	ENSA	endosulfine alpha [Source:HGNC Symbol;Acc:HGNC:3360]
ENSG00000004779	1.81747	7.98959	33.61459	6.72E-09	7.72E-07	NDUFAB1	NADH:ubiquinone oxidoreductase subunit AB1 [Source:HGNC Symbol;Acc:HGNC:7694]
ENSG00000099942	1.18977	8.52265	33.38503	7.56E-09	8.15E-07	CRKL	CRK like proto-oncogene, adaptor protein [Source:HGNC Symbol;Acc:HGNC:2363]
ENSG00000005194	2.24576	7.20748	30.37993	3.55E-08	3.06E-06	CIAPIN1	cytokine induced apoptosis inhibitor 1 [Source:HGNC Symbol;Acc:HGNC:28050]
ENSG00000138385	0.67268	9.66788	30.13051	4.04E-08	3.18E-06	SSB	Sjogren syndrome antigen B [Source:HGNC Symbol;Acc:HGNC:11316]
ENSG00000115677	0.97378	8.90102	29.85969	4.64E-08	3.37E-06	HDLBP	high density lipoprotein binding protein [Source:HGNC Symbol;Acc:HGNC:4857]
ENSG00000070081	1.16560	8.75927	28.85410	7.80E-08	4.98E-06	NUCB2	nucleobindin 2 [Source:HGNC Symbol;Acc:HGNC:8044]
ENSG00000054116	3.40844	6.59476	27.31377	1.73E-07	9.94E-06	TRAPPC3	trafficking protein particle complex 3 [Source:HGNC Symbol;Acc:HGNC:19942]
ENSG00000100650	2.89551	6.97919	25.46220	4.51E-07	2.43E-05	SRSF5	serine and arginine rich splicing factor 5 [Source:HGNC Symbol;Acc:HGNC:10787]
ENSG00000151150	2.71985	7.03398	25.39562	4.67E-07	2.44E-05	ANK3	ankyrin 3 [Source:HGNC Symbol;Acc:HGNC:494]
ENSG00000205581	2.27577	7.23870	25.20710	5.15E-07	2.61E-05	HMGN1	high mobility group nucleosome binding domain 1 [Source:HGNC Symbol;Acc:HGNC:4984]
ENSG00000187908	3.52544	7.93586	25.11755	5.39E-07	2.66E-05	DMBT1	deleted in malignant brain tumors 1 [Source:HGNC Symbol;Acc:HGNC:2926]
ENSG00000131016	0.54773	10.96913	24.75191	6.52E-07	3.12E-05	AKAP12	A-kinase anchoring protein 12 [Source:HGNC Symbol;Acc:HGNC:370]
ENSG00000117983	6.03579	9.09665	24.21007	8.64E-07	3.92E-05	MUC5B	mucin 5B, oligomeric mucus/gel-forming [Source:HGNC Symbol;Acc:HGNC:7516]
ENSG00000116455	3.27039	6.74275	23.44734	1.28E-06	5.68E-05	WDR77	WD repeat domain 77 [Source:HGNC Symbol;Acc:HGNC:29652]
ENSG00000101443	3.52974	7.12534	23.22345	1.44E-06	6.22E-05	WFDC2	WAP four-disulfide core domain 2 [Source:HGNC Symbol;Acc:HGNC:15939]
ENSG00000163754	1.23158	8.03692	22.89768	1.71E-06	7.05E-05	GYG1	glycogenin 1 [Source:HGNC Symbol;Acc:HGNC:4699]
ENSG00000164879	1.05705	9.46880	22.88752	1.72E-06	7.05E-05	CA3	carbonic anhydrase 3 [Source:HGNC Symbol;Acc:HGNC:1374]
ENSG00000125977	1.24397	8.02257	22.65407	1.94E-06	7.60E-05	EIF2S2	eukaryotic translation initiation factor 2 subunit beta [Source:HGNC Symbol;Acc:HGNC:3266]
ENSG00000198157	2.54469	6.91097	22.15908	2.51E-06	9.01E-05	HMGN5	high mobility group nucleosome binding domain 5 [Source:HGNC Symbol;Acc:HGNC:8013]
ENSG00000076554	2.23612	7.04991	21.25055	4.03E-06	0.000134	TPD52	tumor protein D52 [Source:HGNC Symbol;Acc:HGNC:12005]
ENSG00000130811	1.12696	8.17608	20.50046	5.96E-06	0.000180	EIF3G	eukaryotic translation initiation factor 3 subunit G [Source:HGNC Symbol;Acc:HGNC:3274]
ENSG00000090273	0.98190	8.53567	20.52810	5.88E-06	0.000180	NUDC	nuclear distribution C, dynein complex regulator [Source:HGNC Symbol;Acc:HGNC:8045]
ENSG00000078140	2.04346	7.11456	20.08800	7.40E-06	0.000216	UBE2K	ubiquitin conjugating enzyme E2 K [Source:HGNC Symbol;Acc:HGNC:4914]
ENSG00000166503	1.33234	7.91794	19.91935	8.08E-06	0.000232	RP11-382A20.3	Hepatoma-derived growth factor-related protein 3 [Source:UniProtKB/Swiss-Prot;Acc:Q9Y3E1]
ENSG00000187239	2.06160	6.95761	19.31237	1.11E-05	0.000304	FNBP1	formin binding protein 1 [Source:HGNC Symbol;Acc:HGNC:17069]
ENSG00000051620	0.75965	8.81799	19.05229	1.27E-05	0.000327	HEBP2	heme binding protein 2 [Source:HGNC Symbol;Acc:HGNC:15716]
ENSG00000104131	1.27930	7.74392	18.98338	1.32E-05	0.000334	EIF3J	eukaryotic translation initiation factor 3 subunit J [Source:HGNC Symbol;Acc:HGNC:3270]
ENSG00000126878	1.61936	7.27324	18.85871	1.41E-05	0.000352	AIF1L	allograft inflammatory factor 1 like [Source:HGNC Symbol;Acc:HGNC:28904]
ENSG00000170606	0.69524	9.73380	18.43806	1.76E-05	0.000415	HSPA4	heat shock protein family A (Hsp70) member 4 [Source:HGNC Symbol;Acc:HGNC:5237]
ENSG00000155660	0.43765	10.71761	18.44511	1.75E-05	0.000415	PDIA4	protein disulfide isomerase family A member 4 [Source:HGNC Symbol;Acc:HGNC:30167]
ENSG00000163636	1.13237	7.96507	17.71441	2.57E-05	0.000575	PSMD6	proteasome 26S subunit, non-ATPase 6 [Source:HGNC Symbol;Acc:HGNC:9564]
ENSG00000165416	1.07752	7.97071	17.36732	3.08E-05	0.000656	SUGT1	SGT1 homolog, MIS12 kinetochore complex assembly cochaperone [Source:HGNC Symbol;Acc:HGNC:16987]
ENSG00000159140	2.49785	6.91185	17.30322	3.19E-05	0.000670	SON	SON DNA binding protein [Source:HGNC Symbol;Acc:HGNC:11183]
ENSG00000147475	1.29421	7.70478	17.09069	3.56E-05	0.000728	ERLIN2	ER lipid raft associated 2 [Source:HGNC Symbol;Acc:HGNC:1356]
ENSG00000142864	0.84117	8.39429	17.10256	3.54E-05	0.000728	SERBP1	SERPINE1 mRNA binding protein 1 [Source:HGNC Symbol;Acc:HGNC:17860]
ENSG00000166741	2.42305	7.37522	16.60714	4.60E-05	0.000901	NNMT	nicotinamide N-methyltransferase [Source:HGNC Symbol;Acc:HGNC:7861]
ENSG00000277443	0.50345	9.75373	16.35836	5.24E-05	0.000987	MARCKS	myristoylated alanine rich protein kinase C substrate [Source:HGNC Symbol;Acc:HGNC:6759]
ENSG00000173692	0.88513	8.79821	16.20900	5.67E-05	0.001040	PSMD1	proteasome 26S subunit, non-ATPase 1 [Source:HGNC Symbol;Acc:HGNC:9554]
ENSG00000184432	0.89617	8.79262	16.01453	6.29E-05	0.001141	COPB2	coatomer protein complex subunit beta 2 [Source:HGNC Symbol;Acc:HGNC:2232]
ENSG00000163520	0.74055	9.80336	15.50788	8.22E-05	0.001475	FBLN2	fibulin 2 [Source:HGNC Symbol;Acc:HGNC:3601]
ENSG00000070756	1.18953	7.85224	15.40760	8.66E-05	0.001540	PABPC1	poly(A) binding protein cytoplasmic 1 [Source:HGNC Symbol;Acc:HGNC:8554]
ENSG00000078018	2.45636	7.11836	15.32270	9.06E-05	0.001594	MAP2	microtubule associated protein 2 [Source:HGNC Symbol;Acc:HGNC:6839]
ENSG00000158417	2.12771	6.89489	15.08687	0.000103	0.001753	EIF5B	eukaryotic translation initiation factor 5B [Source:HGNC Symbol;Acc:HGNC:30793]
ENSG00000100442	0.85163	8.20422	14.41330	0.000147	0.002481	FKBP3	FK506 binding protein 3 [Source:HGNC Symbol;Acc:HGNC:3719]
ENSG00000187955	0.61647	11.49944	14.22497	0.000162	0.002604	COL14A1	collagen type XIV alpha 1 chain [Source:HGNC Symbol;Acc:HGNC:2191]
ENSG00000135316	0.52667	9.38945	14.21464	0.000163	0.002604	SYNCRIP	synaptotagmin binding cytoplasmic RNA interacting protein [Source:HGNC Symbol;Acc:HGNC:16918]
ENSG00000102241	1.84621	6.98484	14.14472	0.000169	0.002606	HTATSF1	HIV-1 Tat specific factor 1 [Source:HGNC Symbol;Acc:HGNC:5276]
ENSG00000162191	1.62760	7.13907	14.15891	0.000168	0.002606	UBXN1	UBX domain protein 1 [Source:HGNC Symbol;Acc:HGNC:18402]
ENSG00000163453	1.47188	7.24300	13.89760	0.000193	0.002894	IGFBP7	insulin like growth factor binding protein 7 [Source:HGNC Symbol;Acc:HGNC:5476]
ENSG00000119640	1.65953	7.09315	13.76252	0.000207	0.003005	ACYP1	acylphosphatase 1 [Source:HGNC Symbol;Acc:HGNC:179]
ENSG00000166165	0.49900	10.49258	13.70337	0.000214	0.003067	CKB	creatine kinase B [Source:HGNC Symbol;Acc:HGNC:1991]
ENSG00000247596	1.31033	7.39852	13.27525	0.000269	0.003680	TWF2	twinfilin actin binding protein 2 [Source:HGNC Symbol;Acc:HGNC:9621]
ENSG00000180817	0.42389	9.86849	12.89317	0.000330	0.004407	PPA1	pyrophosphatase (inorganic) 1 [Source:HGNC Symbol;Acc:HGNC:9226]
ENSG00000170476	1.20862	8.70125	12.86970	0.000334	0.004429	MZB1	marginal zone B and B1 cell specific protein [Source:HGNC Symbol;Acc:HGNC:30125]
ENSG00000110075	2.37618	6.56709	12.83413	0.000340	0.004479	PPP6R3	protein phosphatase 6 regulatory subunit 3 [Source:HGNC Symbol;Acc:HGNC:1173]
ENSG00000127527	0.78242	8.34436	12.36664	0.000437	0.005666	EPS15L1	epidermal growth factor receptor pathway substrate 15 like 1 [Source:HGNC Symbol;Acc:HGNC:24634]
ENSG00000121769	1.20774	7.78707	12.33514	0.000445	0.005719	FABP3	fatty acid binding protein 3 [Source:HGNC Symbol;Acc:HGNC:3557]
ENSG00000150593	1.04568	8.19717	12.19895	0.000478	0.006089	PDCD4	programmed cell death 4 [Source:HGNC Symbol;Acc:HGNC:8763]
ENSG00000106804	0.77981	9.63506	12.19046	0.000480	0.006089	C5	complement C5 [Source:HGNC Symbol;Acc:HGNC:1331]
ENSG00000140905	1.79804	6.95702	12.07916	0.000510	0.006414	GCSH	glycine cleavage system protein H [Source:HGNC Symbol;Acc:HGNC:4208]
ENSG00000172403	1.71813	8.85307	12.06642	0.000513	0.006414	SYNPO2	synaptopodin 2 [Source:HGNC Symbol;Acc:HGNC:17732]
ENSG00000221823	2.04010	6.71928	12.00846	0.000530	0.006521	PPP3R1	protein phosphatase 3 regulatory subunit B, alpha [Source:HGNC Symbol;Acc:HGNC:9317]
ENSG00000114354	1.44964	7.33921	11.98907	0.000535	0.006521	TFG	TRK-fused gene [Source:HGNC Symbol;Acc:HGNC:11758]
ENSG00000117906	1.10966	7.66889	11.98210	0.000537	0.006521	RCN2	reticulocalbin 2 [Source:HGNC Symbol;Acc:HGNC:9935]
ENSG00000125844	0.55935	10.86402	11.91738	0.000556	0.006705	RRBP1	ribosome binding protein 1 [Source:HGNC Symbol;Acc:HGNC:10448]
ENSG00000143401	0.75234	8.41091	11.83139	0.000582	0.006973	ANP32E	acidic nuclear phosphoprotein 32 family member E [Source:HGNC Symbol;Acc:HGNC:16673]
ENSG00000100348	1.68418	6.93132	11.78711	0.000596	0.007091	TXN2	thioredoxin 2 [Source:HGNC Symbol;Acc:HGNC:17772]
ENSG00000172939	1.10644	7.76717	11.70659	0.000623	0.007304	OXSR1	oxidative stress responsive 1 [Source:HGNC Symbol;Acc:HGNC:8508]
ENSG00000101856	1.29083	7.27950	11.62953	0.000649	0.007461	PGRMC1	progesterone receptor membrane component 1 [Source:HGNC Symbol;Acc:HGNC:16090]
ENSG00000213619	0.99287	7.89313	11.56804	0.000671	0.007660	NDUFS3	NADH:ubiquinone oxidoreductase core subunit S3 [Source:HGNC Symbol;Acc:HGNC:7710]
ENSG00000164258	1.35936	7.43260	11.52145	0.000688	0.007784	NDUFS4	NADH:ubiquinone oxidoreductase subunit S4 [Source:HGNC Symbol;Acc:HGNC:7711]
ENSG00000116161	0.69384	8.35646	11.50585	0.000694	0.007784	CACYBP	calcyclin binding protein [Source:HGNC Symbol;Acc:HGNC:30423]
ENSG00000166482	0.66331	9.29411	11.40976	0.000731	0.008126	MFAP4	microfibrillar associated protein 4 [Source:HGNC Symbol;Acc:HGNC:7035]
ENSG00000184357	0.80869	8.05779	11.37737	0.000743	0.008216	HIST1H1B	histone cluster 1 H1 family member b [Source:HGNC Symbol;Acc:HGNC:4719]
ENSG00000183963	2.37825	7.21158	11.29993	0.000775	0.008457	SMTN	smoothelin [Source:HGNC Symbol;Acc:HGNC:11126]
ENSG00000111642	2.04250	6.73210	11.18843	0.000823	0.008925	CHD4	chromodomain helicase DNA binding protein 4 [Source:HGNC Symbol;Acc:HGNC:1919]
ENSG00000123131	1.08580	8.09242	11.11809	0.000855	0.009154	PRDX4	peroxiredoxin 4 [Source:HGNC Symbol;Acc:HGNC:17169]
ENSG00000141741	2.01209	6.98172	10.94742	0.000937	0.009794	MIEN1	migration and invasion enhancer 1 [Source:HGNC Symbol;Acc:HGNC:28230]
ENSG00000121022	1.60576	7.03050	10.88269	0.000971	0.009935	COPS5	COP9 signalosome subunit 5 [Source:HGNC Symbol;Acc:HGNC:2240]
ENSG00000145050	1.22339	7.49081	10.82759	0.001000	0.010141	MANF	mesencephalic astrocyte derived neurotrophic factor [Source:HGNC Symbol;Acc:HGNC:15461]
ENSG00000205339	1.41559	7.14156	10.78367	0.001024	0.010204	IPO7	importin 7 [Source:HGNC Symbol;Acc:HGNC:9852]
ENSG00000029725	2.10771	6.86284	10.50780	0.001189	0.011578	RABEP1	rabaptin, RAB GTPase binding effector protein 1 [Source:HGNC Symbol;Acc:HGNC:17677]
ENSG00000136842	0.93789	8.19258	10.48158	0.001206	0.011612	TMOD1	tropomodulin 1 [Source:HGNC Symbol;Acc:HGNC:11871]
ENSG00000142552	1.76826	7.08522	10.42204	0.001245	0.011799	RCN3	reticulocalbin 3 [Source:HGNC Symbol;Acc:HGNC:21145]
ENSG00000136026	1.40979	7.36096	10.32126	0.001315	0.012322	CKAP4	cytoskeleton associated protein 4 [Source:HGNC Symbol;Acc:HGNC:16991]
ENSG00000101150	0.51670	8.99213	10.32404	0.001313	0.012322	TPD52L2	tumor protein D52 like 2 [Source:HGNC Symbol;Acc:HGNC:12007]
ENSG00000131711	3.37228	8.49676	10.28846	0.001339	0.012475	MAP1B	microtubule associated protein 1B [Source:HGNC Symbol;Acc:HGNC:6836]
ENSG00000117691	1.78164	7.30049	10.21656	0.001392	0.012901	NENF	neudesin neurotrophic factor [Source:HGNC Symbol;Acc:HGNC:30384]
ENSG00000108829	1.47800	7.54807	10.17190	0.001426	0.013007	LRRC59	leucine rich repeat containing 59 [Source:HGNC Symbol;Acc:HGNC:28817]

Brandsma C-A, *et al. Thorax* 2020; 75:180–183. doi: 10.1136/thoraxjnl-2019-213200

DE proteins comb FDR 0.05 up

Ensembl gene	logFC	logCPM	LR	PValue	adj.p	gene	geneDescription
ENSG00000177954	1.58926	6.77895	10.10305	0.001480	0.013291	RPS27	ribosomal protein S27 [Source:HGNC Symbol;Acc:HGNC:10416]
ENSG00000152795	0.56674	8.74921	10.03331	0.001537	0.013592	HNRNPDL	heterogeneous nuclear ribonucleoprotein D like [Source:HGNC Symbol;Acc:HGNC:5037]
ENSG00000136854	1.69990	6.80898	9.93424	0.001622	0.014197	STXBP1	syntaxin binding protein 1 [Source:HGNC Symbol;Acc:HGNC:11444]
ENSG00000005893	1.08156	7.49738	9.87148	0.001679	0.014492	LAMP2	lysosomal associated membrane protein 2 [Source:HGNC Symbol;Acc:HGNC:6501]
ENSG00000155368	0.60902	9.34453	9.78387	0.001760	0.015025	DBI	diazepam binding inhibitor, acyl-CoA binding protein [Source:HGNC Symbol;Acc:HGNC:2690]
ENSG00000163956	1.27303	7.80955	9.73078	0.001812	0.015389	LRPAP1	LDL receptor related protein associated protein 1 [Source:HGNC Symbol;Acc:HGNC:6701]
ENSG00000099622	0.76580	8.02867	9.67956	0.001863	0.015747	CIRBP	cold inducible RNA binding protein [Source:HGNC Symbol;Acc:HGNC:1982]
ENSG00000125743	1.13487	7.37467	9.60067	0.001945	0.016278	SNRPD2	small nuclear ribonucleoprotein D2 polypeptide [Source:HGNC Symbol;Acc:HGNC:11159]
ENSG00000106263	0.66166	8.45687	9.58910	0.001957	0.016302	EIF3B	eukaryotic translation initiation factor 3 subunit B [Source:HGNC Symbol;Acc:HGNC:3280]
ENSG00000132485	1.94555	6.99599	9.48384	0.002073	0.017017	ZRANB2	zinc finger RANBP2-type containing 2 [Source:HGNC Symbol;Acc:HGNC:13058]
ENSG00000130175	0.31806	10.38903	9.46988	0.002089	0.017066	PRKCSH	protein kinase C substrate 80K-H [Source:HGNC Symbol;Acc:HGNC:9411]
ENSG00000115884	1.61976	6.75134	9.42656	0.002139	0.017305	SDC1	syndecan 1 [Source:HGNC Symbol;Acc:HGNC:10658]
ENSG00000023228	0.99326	8.36746	9.41848	0.002148	0.017305	NDUFS1	NADH:ubiquinone oxidoreductase core subunit S1 [Source:HGNC Symbol;Acc:HGNC:7707]
ENSG00000088367	1.12198	7.34533	9.39192	0.002179	0.017476	EPB41L1	erythrocyte membrane protein band 4.1 like 1 [Source:HGNC Symbol;Acc:HGNC:3378]
ENSG00000111786	0.88674	7.96805	9.38270	0.002190	0.017483	SRSF9	serine and arginine rich splicing factor 9 [Source:HGNC Symbol;Acc:HGNC:10791]
ENSG00000100941	1.01992	7.48999	9.19519	0.002427	0.018929	PNN	pinin, desmosome associated protein [Source:HGNC Symbol;Acc:HGNC:9162]
ENSG00000198380	1.83660	7.04375	9.17517	0.002453	0.018993	GFPT1	glutamine--fructose-6-phosphate transaminase 1 [Source:HGNC Symbol;Acc:HGNC:4241]
ENSG00000133706	1.26230	7.26335	9.17255	0.002457	0.018993	LARS	leucyl-tRNA synthetase [Source:HGNC Symbol;Acc:HGNC:6512]
ENSG00000164830	1.79061	7.00280	9.13785	0.002504	0.019184	OXR1	oxidation resistance 1 [Source:HGNC Symbol;Acc:HGNC:15822]
ENSG00000136193	0.76397	8.29945	9.07135	0.002596	0.019719	SCRN1	secernin 1 [Source:HGNC Symbol;Acc:HGNC:22192]
ENSG00000182512	1.19803	7.19532	9.02033	0.002670	0.020013	GLRX5	glutaredoxin 5 [Source:HGNC Symbol;Acc:HGNC:20134]
ENSG00000106853	1.59008	7.34472	9.00054	0.002699	0.020020	PTGR1	prostaglandin reductase 1 [Source:HGNC Symbol;Acc:HGNC:18429]
ENSG00000108821	1.24378	7.59302	8.99604	0.002706	0.020020	COL1A1	collagen type I alpha 1 chain [Source:HGNC Symbol;Acc:HGNC:2197]
ENSG00000108465	1.69346	7.11116	8.82765	0.002967	0.021696	CDK5RAP3	CDK5 regulatory subunit associated protein 3 [Source:HGNC Symbol;Acc:HGNC:18673]
ENSG00000164754	1.52829	6.99930	8.82578	0.002970	0.021696	RAD21	RAD21 cohesin complex component [Source:HGNC Symbol;Acc:HGNC:9811]
ENSG00000085063	0.97207	7.53217	8.80223	0.003009	0.021765	CD59	CD59 molecule [Source:HGNC Symbol;Acc:HGNC:1689]
ENSG00000065534	0.38083	10.34490	8.80091	0.003011	0.021765	MYLK	myosin light chain kinase [Source:HGNC Symbol;Acc:HGNC:7590]
ENSG00000113013	0.43555	10.30085	8.78026	0.003045	0.021874	HSPA9	heat shock protein family A (Hsp70) member 9 [Source:HGNC Symbol;Acc:HGNC:5244]
ENSG00000143321	0.36793	10.02498	8.75611	0.003086	0.021982	HDGF	hepatoma derived growth factor [Source:HGNC Symbol;Acc:HGNC:4856]
ENSG00000110958	0.71771	8.75332	8.74768	0.003100	0.021993	PTGES3	prostaglandin E synthase 3 [Source:HGNC Symbol;Acc:HGNC:16049]
ENSG00000038427	1.41757	7.74305	8.73422	0.003123	0.022065	VCAN	versican [Source:HGNC Symbol;Acc:HGNC:2464]
ENSG00000166228	1.35149	7.57456	8.64564	0.003278	0.022976	PCBD1	pterin-4 alpha-carbinolamine dehydratase 1 [Source:HGNC Symbol;Acc:HGNC:8646]
ENSG00000119318	0.38267	9.53995	8.64908	0.003272	0.022976	RAD23B	RAD23 homolog B, nucleotide excision repair protein [Source:HGNC Symbol;Acc:HGNC:9813]
ENSG00000116120	1.31679	7.58183	8.61504	0.003334	0.023131	FARSB	phenylalanyl-tRNA synthetase beta subunit [Source:HGNC Symbol;Acc:HGNC:17800]
ENSG00000125166	1.23091	8.21647	8.61275	0.003338	0.023131	GOT2	glutamic-oxaloacetic transaminase 2 [Source:HGNC Symbol;Acc:HGNC:4433]
ENSG00000103381	0.82433	7.75809	8.61127	0.003341	0.023131	CPPED1	calcineurin like phosphoesterase domain containing 1 [Source:HGNC Symbol;Acc:HGNC:25632]
ENSG00000163902	1.00140	8.13714	8.58105	0.003397	0.023331	RPN1	ribophorin I [Source:HGNC Symbol;Acc:HGNC:10381]
ENSG00000133789	0.89878	7.92157	8.58816	0.003384	0.023331	SWAP70	SWAP switching B-cell complex subunit 70 [Source:HGNC Symbol;Acc:HGNC:17070]
ENSG00000113758	0.77754	7.91167	8.54684	0.003461	0.023606	DBN1	drebrin 1 [Source:HGNC Symbol;Acc:HGNC:2695]
ENSG00000160213	0.61979	8.58023	8.47220	0.003606	0.024285	CSTB	cystatin B [Source:HGNC Symbol;Acc:HGNC:2482]
ENSG00000187109	0.55503	8.61420	8.47530	0.003600	0.024285	NAP1L1	nucleosome assembly protein 1 like 1 [Source:HGNC Symbol;Acc:HGNC:7637]
ENSG00000106244	0.96475	7.55883	8.42072	0.003710	0.024885	PDAP1	PDGFA associated protein 1 [Source:HGNC Symbol;Acc:HGNC:14634]
ENSG00000136450	0.43568	9.91084	8.36434	0.003827	0.025478	SRSF1	serine and arginine rich splicing factor 1 [Source:HGNC Symbol;Acc:HGNC:10780]
ENSG00000163818	1.62677	7.54969	8.27438	0.004021	0.026511	LZTFL1	leucine zipper transcription factor like 1 [Source:HGNC Symbol;Acc:HGNC:6741]
ENSG00000011454	1.59643	6.66985	8.27549	0.004018	0.026511	RABGAP1	RAB GTPase activating protein 1 [Source:HGNC Symbol;Acc:HGNC:17155]
ENSG00000108561	0.62754	8.81543	8.27074	0.004029	0.026511	C1QBP	complement C1q binding protein [Source:HGNC Symbol;Acc:HGNC:1243]
ENSG00000120705	0.84845	7.75251	8.24860	0.004078	0.026734	ETF1	eukaryotic translation termination factor 1 [Source:HGNC Symbol;Acc:HGNC:3477]
ENSG00000105887	0.61983	8.54982	8.21537	0.004154	0.027125	MTPN	myotrophin [Source:HGNC Symbol;Acc:HGNC:15667]
ENSG00000011304	0.37216	9.58864	8.15286	0.004299	0.027865	PTBP1	polypyrimidine tract binding protein 1 [Source:HGNC Symbol;Acc:HGNC:9583]
ENSG00000113643	1.13348	7.68170	8.12388	0.004369	0.028208	RARS	arginyl-tRNA synthetase [Source:HGNC Symbol;Acc:HGNC:9870]
ENSG00000135842	0.93124	7.84149	8.10459	0.004415	0.028403	FAM129A	family with sequence similarity 129 member A [Source:HGNC Symbol;Acc:HGNC:16784]
ENSG00000176788	0.44133	9.36601	8.08839	0.004455	0.028552	BASP1	brain abundant membrane attached signal protein 1 [Source:HGNC Symbol;Acc:HGNC:957]
ENSG00000107317	1.28415	7.12876	8.02227	0.004621	0.029286	PTGDS	prostaglandin D2 synthase [Source:HGNC Symbol;Acc:HGNC:9592]
ENSG00000159212	1.19115	8.73217	7.99497	0.004691	0.029514	CLIC6	chloride intracellular channel 6 [Source:HGNC Symbol;Acc:HGNC:2065]
ENSG00000126777	0.51360	10.43675	7.83855	0.005114	0.031831	KTN1	kinectin 1 [Source:HGNC Symbol;Acc:HGNC:6467]
ENSG00000133226	1.25995	7.17572	7.80812	0.005201	0.032255	SRRM1	serine and arginine repetitive matrix 1 [Source:HGNC Symbol;Acc:HGNC:16638]
ENSG00000147649	0.89013	7.60465	7.76625	0.005323	0.032659	MTDH	metadherin [Source:HGNC Symbol;Acc:HGNC:29608]
ENSG00000137124	1.82284	7.05053	7.72059	0.005459	0.033258	ALDH1B1	aldehyde dehydrogenase 1 family member B1 [Source:HGNC Symbol;Acc:HGNC:407]
ENSG00000070814	1.17629	7.67482	7.64035	0.005708	0.034526	TCOF1	treacle ribosome biogenesis factor 1 [Source:HGNC Symbol;Acc:HGNC:11654]
ENSG00000134440	0.77568	8.22441	7.61030	0.005804	0.034984	NARS	asparaginyl-tRNA synthetase [Source:HGNC Symbol;Acc:HGNC:7643]
ENSG00000126524	0.60139	8.25413	7.57852	0.005907	0.035336	SBDS	SBDS ribosome assembly guanine nucleotide exchange factor [Source:HGNC Symbol;Acc:HGNC:19440]
ENSG00000069275	0.96357	7.87618	7.56039	0.005967	0.035470	NUCKS1	nuclear casein kinase and cyclin dependent kinase substrate 1 [Source:HGNC Symbol;Acc:HGNC:29923]
ENSG00000117899	0.76598	7.84490	7.52377	0.006089	0.035916	MESDC2	mesoderm development candidate 2 [Source:HGNC Symbol;Acc:HGNC:13520]
ENSG00000065613	0.70897	7.92264	7.51318	0.006125	0.035916	SLK	STE20 like kinase [Source:HGNC Symbol;Acc:HGNC:11088]
ENSG00000196531	0.49920	8.68219	7.50599	0.006149	0.035938	NACA	nascent polypeptide-associated complex alpha subunit [Source:HGNC Symbol;Acc:HGNC:7629]
ENSG00000118939	0.68358	7.89735	7.43285	0.006404	0.037176	UCHL3	ubiquitin C-terminal hydrolase L3 [Source:HGNC Symbol;Acc:HGNC:12515]
ENSG00000131725	1.13110	7.27239	7.36518	0.006650	0.038471	WDR44	WD repeat domain 44 [Source:HGNC Symbol;Acc:HGNC:30512]
ENSG00000170017	1.61385	7.35713	7.20276	0.007279	0.041011	ALCAM	activated leukocyte cell adhesion molecule [Source:HGNC Symbol;Acc:HGNC:400]
ENSG00000004700	1.10938	7.30187	7.20464	0.007272	0.041011	RECQL	RecQ like helicase [Source:HGNC Symbol;Acc:HGNC:9948]
ENSG00000198589	1.10267	7.80885	7.20456	0.007272	0.041011	LRBA	LPS responsive beige-like anchor protein [Source:HGNC Symbol;Acc:HGNC:1742]
ENSG00000147010	0.91851	7.77556	7.21884	0.007214	0.041011	SH3KBP1	SH3 domain containing kinase binding protein 1 [Source:HGNC Symbol;Acc:HGNC:13867]
ENSG00000072110	0.33648	11.36299	7.10811	0.007674	0.042538	ACTN1	actinin alpha 1 [Source:HGNC Symbol;Acc:HGNC:163]
ENSG00000140939	1.19093	7.12788	7.00411	0.008132	0.044395	NOL3	nucleolar protein 3 [Source:HGNC Symbol;Acc:HGNC:7869]
ENSG00000039068	1.14570	7.57053	7.00300	0.008137	0.044395	CDH1	cadherin 1 [Source:HGNC Symbol;Acc:HGNC:1748]
ENSG00000132780	0.69986	8.21702	7.00866	0.008112	0.044395	NASP	nuclear autoantigenic sperm protein [Source:HGNC Symbol;Acc:HGNC:7644]
ENSG00000149021	0.91951	9.39535	6.95652	0.008351	0.045419	SCGB1A1	secretoglobin family 1A member 1 [Source:HGNC Symbol;Acc:HGNC:12523]
ENSG00000136068	0.26531	12.05942	6.94844	0.008389	0.045481	FLNB	filamin B [Source:HGNC Symbol;Acc:HGNC:3755]
ENSG00000082397	0.72031	8.59886	6.89066	0.008665	0.046765	EPB41L3	erythrocyte membrane protein band 4.1 like 3 [Source:HGNC Symbol;Acc:HGNC:3380]
ENSG00000167193	0.44423	9.11476	6.88747	0.008680	0.046765	CRK	CRK proto-oncogene, adaptor protein [Source:HGNC Symbol;Acc:HGNC:2362]
ENSG00000174059	1.03007	7.18482	6.83715	0.008928	0.047653	CD34	CD34 molecule [Source:HGNC Symbol;Acc:HGNC:1662]
ENSG00000122218	0.59310	8.90191	6.74017	0.009427	0.049834	COPA	coatomer protein complex subunit alpha [Source:HGNC Symbol;Acc:HGNC:2230]

Brandsma C-A, *et al. Thorax* 2020; 75:180–183. doi: 10.1136/thoraxjnl-2019-213200

DE proteins comb FDR 0.05 down

Ensembl gene	logFC	logCPM	LR	PValue	adj.p	gene	geneDescription
ENSG00000013016	-2.81437	7.60025	46.93976	7.32E-12	6.31E-09	EHD3	EH domain containing 3 [Source:HGNC Symbol;Acc:HGNC:3244]
ENSG00000049860	-1.62317	8.44118	45.30247	1.69E-11	9.70E-09	HEXB	hexosaminidase subunit beta [Source:HGNC Symbol;Acc:HGNC:4879]
ENSG00000115109	-4.22378	6.78620	44.16874	3.01E-11	1.14E-08	EPB41L5	erythrocyte membrane protein band 4.1 like 5 [Source:HGNC Symbol;Acc:HGNC:19819]
ENSG00000116962	-0.78593	9.99436	40.52745	1.94E-10	4.18E-08	NID1	nidogen 1 [Source:HGNC Symbol;Acc:HGNC:7821]
ENSG00000138772	-0.67329	10.55724	36.63479	1.42E-09	2.41E-07	ANXA3	annexin A3 [Source:HGNC Symbol;Acc:HGNC:541]
ENSG00000170248	-0.72888	10.03715	35.66082	2.35E-09	3.11E-07	PDCD6IP	programmed cell death 6 interacting protein [Source:HGNC Symbol;Acc:HGNC:8766]
ENSG00000164120	-2.97731	7.00123	34.18710	5.01E-09	6.16E-07	HPGD	hydroxyprostaglandin dehydrogenase 15-(NAD) [Source:HGNC Symbol;Acc:HGNC:5154]
ENSG00000034510	-2.63016	7.86195	32.94964	9.46E-09	9.59E-07	TMSB10	thymosin beta 10 [Source:HGNC Symbol;Acc:HGNC:11879]
ENSG00000169583	-1.28476	8.52447	31.50627	1.99E-08	1.90E-06	CLIC3	chloride intracellular channel 3 [Source:HGNC Symbol;Acc:HGNC:2064]
ENSG00000074800	-0.52822	11.21231	31.18070	2.35E-08	2.13E-06	ENO1	enolase 1 [Source:HGNC Symbol;Acc:HGNC:3350]
ENSG00000205542	-1.36636	10.22675	30.12424	4.05E-08	3.18E-06	TMSB4X	thymosin beta 4, X-linked [Source:HGNC Symbol;Acc:HGNC:11881]
ENSG00000198682	-2.68513	7.34941	29.84082	4.69E-08	3.37E-06	PAPSS2	3'-phosphoadenosine 5'-phosphosulfate synthase 2 [Source:HGNC Symbol;Acc:HGNC:8604]
ENSG00000130702	-0.70059	10.30611	29.57212	5.39E-08	3.72E-06	LAMA5	laminin subunit alpha 5 [Source:HGNC Symbol;Acc:HGNC:6485]
ENSG00000167315	-0.85452	9.35052	29.23111	6.42E-08	4.26E-06	ACAA2	acetyl-CoA acyltransferase 2 [Source:HGNC Symbol;Acc:HGNC:83]
ENSG00000168477	-1.89982	9.80196	28.36435	1.00E-07	6.19E-06	TNXB	tenascin XB [Source:HGNC Symbol;Acc:HGNC:11976]
ENSG00000148672	-0.60409	10.23071	28.04081	1.19E-07	7.06E-06	GLUD1	glutamate dehydrogenase 1 [Source:HGNC Symbol;Acc:HGNC:4335]
ENSG00000111275	-0.58721	11.13415	25.48981	4.45E-07	2.43E-05	ALDH2	aldehyde dehydrogenase 2 family (mitochondrial) [Source:HGNC Symbol;Acc:HGNC:404]
ENSG00000117305	-1.89449	7.09885	24.56437	7.19E-07	3.35E-05	HMGCL	3-hydroxymethyl-3-methylglutaryl-CoA lyase [Source:HGNC Symbol;Acc:HGNC:5005]
ENSG00000185651	-2.62409	6.68901	22.83721	1.76E-06	7.07E-05	UBE2L3	ubiquitin conjugating enzyme E2 L3 [Source:HGNC Symbol;Acc:HGNC:12488]
ENSG00000120708	-1.10469	8.79839	22.45474	2.15E-06	8.20E-05	TGFBI	transforming growth factor beta induced [Source:HGNC Symbol;Acc:HGNC:11771]
ENSG00000138758	-0.91505	8.56043	22.42345	2.19E-06	8.20E-05	sep-11	septin 11 [Source:HGNC Symbol;Acc:HGNC:25589]
ENSG00000198561	-1.00289	8.92156	22.29616	2.34E-06	8.57E-05	CTNND1	catenin delta 1 [Source:HGNC Symbol;Acc:HGNC:2515]
ENSG00000168028	-2.79684	6.67506	21.97445	2.76E-06	9.72E-05	RPSA	ribosomal protein SA [Source:HGNC Symbol;Acc:HGNC:6502]
ENSG00000178209	-0.44279	13.14757	21.54167	3.46E-06	0.000119	PLEC	plectin [Source:HGNC Symbol;Acc:HGNC:9069]
ENSG00000168610	-1.06382	8.50305	21.42241	3.68E-06	0.000125	STAT3	signal transducer and activator of transcription 3 [Source:HGNC Symbol;Acc:HGNC:11364]
ENSG00000164687	-0.78815	10.71858	20.91265	4.81E-06	0.000156	FABP5	fatty acid binding protein 5 [Source:HGNC Symbol;Acc:HGNC:3560]
ENSG00000168036	-0.60022	9.45389	20.74630	5.24E-06	0.000167	CTNNB1	catenin beta 1 [Source:HGNC Symbol;Acc:HGNC:2514]
ENSG00000165140	-0.76401	10.02198	20.66684	5.47E-06	0.000171	FBP1	fructose-bisphosphatase 1 [Source:HGNC Symbol;Acc:HGNC:3606]
ENSG00000075618	-0.87128	8.96380	20.47313	6.05E-06	0.000180	FSCN1	fascin actin-bundling protein 1 [Source:HGNC Symbol;Acc:HGNC:11148]
ENSG00000143149	-0.62506	9.42968	19.69623	9.08E-06	0.000257	ALDH9A1	aldehyde dehydrogenase 9 family member A1 [Source:HGNC Symbol;Acc:HGNC:412]
ENSG00000151552	-1.15301	7.92922	19.51608	9.98E-06	0.000277	QDPR	quinoid dihydropteridine reductase [Source:HGNC Symbol;Acc:HGNC:9752]
ENSG00000157500	-1.63113	7.83715	19.19308	1.18E-05	0.000311	APPL1	adaptor protein, phosphotyrosine interacting with PH domain and leucine zipper 1 [Source:HGNC Symbol;Acc:HGNC:24035]
ENSG00000079739	-0.55251	10.28301	19.17552	1.19E-05	0.000311	PGM1	phosphoglucomutase 1 [Source:HGNC Symbol;Acc:HGNC:8905]
ENSG00000167815	-0.42962	10.94033	19.22501	1.16E-05	0.000311	PRDX2	peroxiredoxin 2 [Source:HGNC Symbol;Acc:HGNC:9353]
ENSG00000131238	-1.59834	7.78955	18.74933	1.49E-05	0.000367	PPT1	palmitoyl-protein thioesterase 1 [Source:HGNC Symbol;Acc:HGNC:9325]
ENSG00000148484	-0.79475	8.96716	18.48101	1.72E-05	0.000415	RSU1	Ras suppressor protein 1 [Source:HGNC Symbol;Acc:HGNC:10464]
ENSG00000136830	-0.66774	9.05140	18.29120	1.90E-05	0.000442	FAM129B	family with sequence similarity 129 member B [Source:HGNC Symbol;Acc:HGNC:25282]
ENSG00000172725	-0.71013	8.75826	18.23431	1.95E-05	0.000449	CORO1B	coronin 1B [Source:HGNC Symbol;Acc:HGNC:2253]
ENSG00000186522	-1.42743	7.34817	18.12863	2.06E-05	0.000468	sep-10	septin 10 [Source:HGNC Symbol;Acc:HGNC:14349]
ENSG00000131236	-0.36498	10.96810	17.68973	2.60E-05	0.000575	CAP1	adenylate cyclase associated protein 1 [Source:HGNC Symbol;Acc:HGNC:20040]
ENSG00000117748	-2.21436	6.94211	17.61189	2.71E-05	0.000591	RPA2	replication protein A2 [Source:HGNC Symbol;Acc:HGNC:10290]
ENSG00000187498	-0.91617	8.16199	17.46597	2.92E-05	0.000630	COL4A1	collagen type IV alpha 1 chain [Source:HGNC Symbol;Acc:HGNC:2202]
ENSG00000111237	-1.05794	7.78086	17.07758	3.59E-05	0.000728	VPS29	VPS29, retromer complex component [Source:HGNC Symbol;Acc:HGNC:14340]
ENSG00000111640	-0.31294	11.28062	16.98394	3.77E-05	0.000756	GAPDH	glyceraldehyde-3-phosphate dehydrogenase [Source:HGNC Symbol;Acc:HGNC:4141]
ENSG00000087274	-0.57813	9.65633	16.65898	4.47E-05	0.000887	ADD1	adducin 1 [Source:HGNC Symbol;Acc:HGNC:243]
ENSG00000148671	-0.92272	8.62570	16.58183	4.66E-05	0.000903	ADIRF	adipogenesis regulatory factor [Source:HGNC Symbol;Acc:HGNC:24043]
ENSG00000089159	-1.13856	8.11424	16.48403	4.91E-05	0.000940	PXN	paxillin [Source:HGNC Symbol;Acc:HGNC:9718]
ENSG00000104763	-0.81676	9.88523	16.34924	5.27E-05	0.000987	ASAH1	N-acylsphingosine amidohydrolase 1 [Source:HGNC Symbol;Acc:HGNC:735]
ENSG00000117984	-0.66079	11.03541	16.29752	5.41E-05	0.001004	CTSD	cathepsin D [Source:HGNC Symbol;Acc:HGNC:2529]
ENSG00000142507	-0.89179	8.01377	15.29113	9.21E-05	0.001605	PSMB6	proteasome subunit beta 6 [Source:HGNC Symbol;Acc:HGNC:9543]
ENSG00000197879	-0.58587	10.90308	15.21060	9.62E-05	0.001658	MYO1C	myosin IC [Source:HGNC Symbol;Acc:HGNC:7597]
ENSG00000137486	-0.69422	8.72915	14.39144	0.000148	0.002485	ARRB1	arrestin beta 1 [Source:HGNC Symbol;Acc:HGNC:711]
ENSG00000100034	-0.96078	8.04498	14.22497	0.000162	0.002604	PPM1F	protein phosphatase, Mg2+/Mn2+ dependent 1F [Source:HGNC Symbol;Acc:HGNC:19388]
ENSG00000079308	-0.58554	10.67421	14.22027	0.000163	0.002604	TNS1	tensin 1 [Source:HGNC Symbol;Acc:HGNC:11973]
ENSG00000139684	-0.58006	9.11822	14.28374	0.000157	0.002604	ESD	esterase D [Source:HGNC Symbol;Acc:HGNC:3465]
ENSG00000188612	-1.62043	6.95224	14.19596	0.000165	0.002605	SUMO2	small ubiquitin-like modifier 2 [Source:HGNC Symbol;Acc:HGNC:11125]
ENSG00000071127	-0.41306	10.27655	14.17796	0.000166	0.002606	WDR1	WD repeat domain 1 [Source:HGNC Symbol;Acc:HGNC:12754]
ENSG00000111144	-0.36292	11.09456	14.07455	0.000176	0.002681	LTA4H	leukotriene A4 hydrolase [Source:HGNC Symbol;Acc:HGNC:6710]
ENSG00000198830	-1.91572	7.39637	13.96539	0.000186	0.002816	HMGN2	high mobility group nucleosomal binding domain 2 [Source:HGNC Symbol;Acc:HGNC:4986]
ENSG00000148180	-0.44652	11.75755	13.84097	0.000199	0.002957	GSN	gelsolin [Source:HGNC Symbol;Acc:HGNC:4620]
ENSG00000024422	-0.54612	11.60422	13.82184	0.000201	0.002962	EHD2	EH domain containing 2 [Source:HGNC Symbol;Acc:HGNC:3243]
ENSG00000141295	-1.90165	6.70962	13.77987	0.000206	0.003003	SCRN2	secernin 2 [Source:HGNC Symbol;Acc:HGNC:30381]
ENSG00000115091	-0.44529	10.19418	13.69312	0.000215	0.003067	ACTR3	ARP3 actin related protein 3 homolog [Source:HGNC Symbol;Acc:HGNC:170]
ENSG00000137106	-0.81005	8.35924	13.55918	0.000231	0.003267	GRHPR	glyoxylate and hydroxypyruvate reductase [Source:HGNC Symbol;Acc:HGNC:4570]
ENSG00000144791	-1.66163	6.95794	13.50726	0.000238	0.003331	LIMD1	LIM domains containing 1 [Source:HGNC Symbol;Acc:HGNC:6612]
ENSG00000143416	-0.30756	11.27770	13.46993	0.000242	0.003370	SELENBP1	selenium binding protein 1 [Source:HGNC Symbol;Acc:HGNC:10719]
ENSG00000152642	-0.71227	8.43791	13.38995	0.000253	0.003489	GPD1L	glycerol-3-phosphate dehydrogenase 1-like [Source:HGNC Symbol;Acc:HGNC:28956]
ENSG00000213719	-0.35412	10.08768	13.18037	0.000283	0.003840	CLIC1	chloride intracellular channel 1 [Source:HGNC Symbol;Acc:HGNC:2062]
ENSG00000147065	-0.51164	11.76985	12.94219	0.000321	0.004327	MSN	moesin [Source:HGNC Symbol;Acc:HGNC:7373]
ENSG00000091136	-0.56983	10.21310	12.81922	0.000343	0.004481	LAMB1	laminin subunit beta 1 [Source:HGNC Symbol;Acc:HGNC:6486]
ENSG00000135047	-1.14540	7.54001	12.00307	0.000531	0.006521	CTSL	cathepsin L [Source:HGNC Symbol;Acc:HGNC:2537]
ENSG00000211893	-0.93724	9.50038	11.75617	0.000606	0.007161	IGHG2	immunoglobulin heavy constant gamma 2 (G2m marker) [Source:HGNC Symbol;Acc:HGNC:5526]
ENSG00000128245	-0.41509	9.51027	11.69151	0.000628	0.007314	YWHAH	tyrosine 3-monooxygenase/tryptophan 5-monooxygenase activation protein eta [Source:HGNC Symbol;Acc:HGNC:12853]
ENSG00000152234	-0.26171	11.05594	11.67879	0.000632	0.007314	ATP5A1	ATP synthase, H+ transporting, mitochondrial F1 complex, alpha subunit 1, cardiac muscle [Source:HGNC Symbol;Acc:HGNC:823]
ENSG00000197894	-0.34734	9.94446	11.50163	0.000695	0.007784	ADH5	alcohol dehydrogenase 5 (class III), chi polypeptide [Source:HGNC Symbol;Acc:HGNC:253]
ENSG00000041357	-0.51922	8.92687	11.34079	0.000758	0.008326	PSMA4	proteasome subunit alpha 4 [Source:HGNC Symbol;Acc:HGNC:9533]
ENSG00000147140	-0.66917	8.81717	11.13491	0.000847	0.009128	NONO	non-POU domain containing, octamer-binding [Source:HGNC Symbol;Acc:HGNC:7871]
ENSG00000162909	-0.30881	10.68795	11.06825	0.000878	0.009346	CAPN2	calpain 2 [Source:HGNC Symbol;Acc:HGNC:1479]
ENSG00000132622	-1.19863	7.63038	11.01200	0.000905	0.009574	HSPA12B	heat shock protein family A (Hsp70) member 12B [Source:HGNC Symbol;Acc:HGNC:16193]
ENSG00000120913	-0.81599	9.24384	10.98782	0.000917	0.009641	PDLIM2	PDZ and LIM domain 2 [Source:HGNC Symbol;Acc:HGNC:13992]
ENSG00000089220	-0.30982	10.32549	10.91477	0.000954	0.009908	PEBP1	phosphatidylethanolamine binding protein 1 [Source:HGNC Symbol;Acc:HGNC:8630]
ENSG00000221914	-1.50599	6.89525	10.89007	0.000967	0.009935	PPP2R2A	protein phosphatase 2 regulatory subunit Balpha [Source:HGNC Symbol;Acc:HGNC:9304]
ENSG00000182718	-0.62839	12.76332	10.87649	0.000974	0.009935	ANXA2	annexin A2 [Source:HGNC Symbol;Acc:HGNC:537]
ENSG00000140945	-1.25614	7.17792	10.79869	0.001016	0.010204	CDH13	cadherin 13 [Source:HGNC Symbol;Acc:HGNC:1753]
ENSG00000170889	-1.14218	8.00508	10.79163	0.001020	0.010204	RPS9	ribosomal protein S9 [Source:HGNC Symbol;Acc:HGNC:10442]
ENSG00000138674	-1.19999	7.95625	10.71648	0.001062	0.010521	SEC31A	SEC31 homolog A, COPII coat complex component [Source:HGNC Symbol;Acc:HGNC:17052]
ENSG00000137710	-0.55929	10.31299	10.66668	0.001091	0.010746	RDX	radixin [Source:HGNC Symbol;Acc:HGNC:9944]
ENSG00000163931	-0.35704	10.98953	10.61169	0.001124	0.011008	TKT	transketolase [Source:HGNC Symbol;Acc:HGNC:11834]
ENSG00000140105	-0.50595	10.78908	10.48617	0.001203	0.011612	WARS	tryptophanyl-tRNA synthetase [Source:HGNC Symbol;Acc:HGNC:12729]
ENSG00000127946	-1.14115	7.31669	10.46498	0.001217	0.011652	HIP1	huntingtin interacting protein 1 [Source:HGNC Symbol;Acc:HGNC:4913]
ENSG00000182492	-0.42545	11.14827	10.42142	0.001246	0.011799	BGN	biglycan [Source:HGNC Symbol;Acc:HGNC:1044]
ENSG00000197747	-0.62301	10.84807	10.18940	0.001413	0.012953	S100A10	S100 calcium binding protein A10 [Source:HGNC Symbol;Acc:HGNC:10487]
ENSG00000114573	-0.54955	9.33550	10.19680	0.001407	0.012953	ATP6V1A	ATPase H+ transporting V1 subunit A [Source:HGNC Symbol;Acc:HGNC:851]
ENSG00000198848	-0.45424	11.06329	10.13717	0.001453	0.013185	CES1	carboxylesterase 1 [Source:HGNC Symbol;Acc:HGNC:1863]
ENSG00000007168	-0.68322	8.37757	10.10826	0.001476	0.013291	PAFAH1B1	platelet activating factor acetylhydrolase 1b regulatory subunit 1 [Source:HGNC Symbol;Acc:HGNC:8574]
ENSG00000106992	-0.37237	9.99635	10.07608	0.001502	0.013417	AK1	adenylate kinase 1 [Source:HGNC Symbol;Acc:HGNC:361]
ENSG00000136205	-1.77395	7.13992	10.06414	0.001512	0.013435	TNS3	tensin 3 [Source:HGNC Symbol;Acc:HGNC:21616]
ENSG00000109861	-0.84321	8.61147	9.98849	0.001575	0.013855	CTSC	cathepsin C [Source:HGNC Symbol;Acc:HGNC:2528]
ENSG00000128311	-1.13004	7.43060	9.90493	0.001648	0.014352	TST	thiosulfate sulfurtransferase [Source:HGNC Symbol;Acc:HGNC:12388]
ENSG00000168090	-1.49660	6.97199	9.86861	0.001681	0.014492	COPS6	COP9 signalosome subunit 6 [Source:HGNC Symbol;Acc:HGNC:21749]
ENSG00000100345	-0.41932	12.99143	9.78622	0.001758	0.015025	MYH9	myosin heavy chain 9 [Source:HGNC Symbol;Acc:HGNC:7579]
ENSG00000196262	-0.43836	10.93969	9.60653	0.001939	0.016278	PPIA	peptidylprolyl isomerase A [Source:HGNC Symbol;Acc:HGNC:9253]
ENSG00000147403	-1.10367	7.39807	9.50962	0.002044	0.016941	RPL10	ribosomal protein L10 [Source:HGNC Symbol;Acc:HGNC:10298]

DE proteins comb FDR 0.05 down

Ensembl gene	logFC	logCPM	LR	PValue	adj.p	gene	geneDescription
ENSG00000160209	-0.46378	9.08879	9.49042	0.002065	0.017017	PDXK	pyridoxal kinase [Source:HGNC Symbol;Acc:HGNC:8819]
ENSG00000123992	-0.84255	8.49926	9.45150	0.002110	0.017157	DNPEP	aspartyl aminopeptidase [Source:HGNC Symbol;Acc:HGNC:2981]
ENSG00000159377	-0.67867	8.23386	9.33643	0.002246	0.017847	PSMB4	proteasome subunit beta 4 [Source:HGNC Symbol;Acc:HGNC:9541]
ENSG00000104823	-0.41538	9.56378	9.26007	0.002342	0.018521	ECH1	enoyl-CoA hydratase 1 [Source:HGNC Symbol;Acc:HGNC:3149]
ENSG00000112782	-0.70042	9.39245	9.23870	0.002370	0.018653	CLIC5	chloride intracellular channel 5 [Source:HGNC Symbol;Acc:HGNC:13517]
ENSG00000102882	-0.60895	8.30738	9.20946	0.002408	0.018867	MAPK3	mitogen-activated protein kinase 3 [Source:HGNC Symbol;Acc:HGNC:6877]
ENSG00000099204	-1.14494	7.53686	9.16003	0.002474	0.019038	ABLIM1	actin binding LIM protein 1 [Source:HGNC Symbol;Acc:HGNC:78]
ENSG00000123130	-0.95589	7.55857	9.12413	0.002523	0.019243	ACOT9	acyl-CoA thioesterase 9 [Source:HGNC Symbol;Acc:HGNC:17152]
ENSG00000102606	-1.34728	6.93084	9.04925	0.002628	0.019872	ARHGEF7	Rho guanine nucleotide exchange factor 7 [Source:HGNC Symbol;Acc:HGNC:15607]
ENSG00000165092	-0.35338	11.07344	9.03835	0.002644	0.019903	ALDH1A1	aldehyde dehydrogenase 1 family member A1 [Source:HGNC Symbol;Acc:HGNC:402]
ENSG00000100106	-0.82425	7.80707	9.00586	0.002691	0.020020	TRIOBP	TRIO and F-actin binding protein [Source:HGNC Symbol;Acc:HGNC:17009]
ENSG00000092964	-0.30075	11.30749	8.82606	0.002970	0.021696	DPYSL2	dihydropyrimidinase like 2 [Source:HGNC Symbol;Acc:HGNC:3014]
ENSG00000122971	-1.70721	7.13334	8.79695	0.003017	0.021765	ACADS	acyl-CoA dehydrogenase, C-2 to C-3 short chain [Source:HGNC Symbol;Acc:HGNC:90]
ENSG00000117450	-0.27728	10.26244	8.75627	0.003085	0.021982	PRDX1	peroxiredoxin 1 [Source:HGNC Symbol;Acc:HGNC:9352]
ENSG00000093144	-1.04776	7.37762	8.54523	0.003464	0.023606	ECHDC1	ethylmalonyl-CoA decarboxylase 1 [Source:HGNC Symbol;Acc:HGNC:21489]
ENSG00000176014	-0.53004	8.55519	8.50462	0.003542	0.024044	TUBB6	tubulin beta 6 class V [Source:HGNC Symbol;Acc:HGNC:20776]
ENSG00000110880	-0.51622	9.30890	8.36385	0.003828	0.025478	CORO1C	coronin 1C [Source:HGNC Symbol;Acc:HGNC:2254]
ENSG00000213614	-0.99091	8.14121	8.17584	0.004245	0.027618	HEXA	hexosaminidase subunit alpha [Source:HGNC Symbol;Acc:HGNC:4878]
ENSG00000073350	-1.39623	6.93425	8.07375	0.004491	0.028578	LLGL2	LLGL2, scribble cell polarity complex component [Source:HGNC Symbol;Acc:HGNC:6629]
ENSG00000137497	-0.60240	10.04696	8.07329	0.004492	0.028578	NUMA1	nuclear mitotic apparatus protein 1 [Source:HGNC Symbol;Acc:HGNC:8059]
ENSG00000133835	-0.56203	10.13404	7.99864	0.004681	0.029514	HSD17B4	hydroxysteroid 17-beta dehydrogenase 4 [Source:HGNC Symbol;Acc:HGNC:5213]
ENSG00000064042	-0.66630	9.39209	7.90131	0.004940	0.030969	LIMCH1	LIM and calponin homology domains 1 [Source:HGNC Symbol;Acc:HGNC:29191]
ENSG00000105538	-1.58621	6.95644	7.88940	0.004973	0.031060	RASIP1	Ras interacting protein 1 [Source:HGNC Symbol;Acc:HGNC:24716]
ENSG00000115306	-0.23355	12.67609	7.78681	0.005263	0.032521	SPTBN1	spectrin beta, non-erythrocytic 1 [Source:HGNC Symbol;Acc:HGNC:11275]
ENSG00000147416	-0.75735	8.41172	7.77994	0.005283	0.032528	ATP6V1B2	ATPase H+ transporting V1 subunit B2 [Source:HGNC Symbol;Acc:HGNC:854]
ENSG00000168385	-0.38504	9.33881	7.74236	0.005394	0.032976	sep-02	septin 2 [Source:HGNC Symbol;Acc:HGNC:7729]
ENSG00000138796	-0.63230	8.70894	7.68641	0.005564	0.033775	HADH	hydroxyacyl-CoA dehydrogenase [Source:HGNC Symbol;Acc:HGNC:4799]
ENSG00000075239	-0.40604	9.42274	7.59587	0.005850	0.035142	ACAT1	acetyl-CoA acetyltransferase 1 [Source:HGNC Symbol;Acc:HGNC:93]
ENSG00000140416	-0.56025	10.81177	7.57342	0.005924	0.035336	TPM1	tropomyosin 1 (alpha) [Source:HGNC Symbol;Acc:HGNC:12010]
ENSG00000176658	-1.36749	7.18662	7.51791	0.006109	0.035916	MYO1D	myosin ID [Source:HGNC Symbol;Acc:HGNC:7598]
ENSG00000050405	-0.64343	8.66352	7.51324	0.006125	0.035916	LIMA1	LIM domain and actin binding 1 [Source:HGNC Symbol;Acc:HGNC:24636]
ENSG00000106624	-0.69523	8.11744	7.49032	0.006203	0.036129	AEBP1	AE binding protein 1 [Source:HGNC Symbol;Acc:HGNC:303]
ENSG00000240065	-0.69340	7.90568	7.33449	0.006764	0.039003	PSMB9	proteasome subunit beta 9 [Source:HGNC Symbol;Acc:HGNC:9546]
ENSG00000105227	-0.81062	9.81765	7.26831	0.007018	0.040331	PRX	periaxin [Source:HGNC Symbol;Acc:HGNC:13797]
ENSG00000121064	-0.96742	7.85582	7.22644	0.007184	0.041011	SCPEP1	serine carboxypeptidase 1 [Source:HGNC Symbol;Acc:HGNC:29507]
ENSG00000030582	-0.71457	7.80306	7.21704	0.007221	0.041011	GRN	granulin precursor [Source:HGNC Symbol;Acc:HGNC:4601]
ENSG00000167460	-0.46584	11.32226	7.17875	0.007377	0.041293	TPM4	tropomyosin 4 [Source:HGNC Symbol;Acc:HGNC:12013]
ENSG00000176619	-0.24790	10.88522	7.18292	0.007360	0.041293	LMNB2	lamin B2 [Source:HGNC Symbol;Acc:HGNC:6638]
ENSG00000104067	-0.52911	10.12346	7.16914	0.007417	0.041381	TJP1	tight junction protein 1 [Source:HGNC Symbol;Acc:HGNC:11827]
ENSG00000130985	-0.19063	11.08183	7.11151	0.007659	0.042538	UBA1	ubiquitin like modifier activating enzyme 1 [Source:HGNC Symbol;Acc:HGNC:12469]
ENSG00000159840	-0.34285	10.07166	7.07346	0.007823	0.043229	ZYX	zyxin [Source:HGNC Symbol;Acc:HGNC:13200]
ENSG00000116774	-0.76490	8.74558	7.05480	0.007905	0.043542	OLFML3	olfactomedin like 3 [Source:HGNC Symbol;Acc:HGNC:24956]
ENSG00000164924	-0.23436	10.75497	6.87974	0.008718	0.046821	YWHAZ	tyrosine 3-monooxygenase/tryptophan 5-monooxygenase activation protein zeta [Source:HGNC Symbol;Acc:HGNC:12855]
ENSG00000107164	-1.17588	6.87481	6.84991	0.008865	0.047461	FUBP3	far upstream element binding protein 3 [Source:HGNC Symbol;Acc:HGNC:4005]
ENSG00000137831	-1.09962	7.18332	6.82999	0.008964	0.047697	UACA	uveal autoantigen with coiled-coil domains and ankyrin repeats [Source:HGNC Symbol;Acc:HGNC:15947]
ENSG00000136235	-0.73719	7.99909	6.79111	0.009161	0.048597	GPNMB	glycoprotein nmb [Source:HGNC Symbol;Acc:HGNC:4462]
ENSG00000162517	-1.11204	6.98149	6.73532	0.009452	0.049834	PEF1	penta-EF-hand domain containing 1 [Source:HGNC Symbol;Acc:HGNC:30009]

DE mRNA comb FDR 0.05 up

Ensembl gene	logFC	logCPM	LR	PValue	adj.p	gene	Description
ENSG00000099860	2.56526	8.12904	31.47425	2.02E-08	0.000106	GADD45B	growth arrest and DNA-damage-inducible, beta [Source:HGNC Symbol;Acc:4096]
ENSG00000118503	1.85829	7.80211	30.35646	3.60E-08	0.000141	TNFAIP3	tumor necrosis factor, alpha-induced protein 3 [Source:HGNC Symbol;Acc:11896]
ENSG00000131016	2.43350	8.74365	27.31879	1.73E-07	0.000446	AKAP12	A kinase (PRKA) anchor protein 12 [Source:HGNC Symbol;Acc:370]
ENSG00000148926	2.24012	4.84307	27.42403	1.63E-07	0.000446	ADM	adrenomedullin [Source:HGNC Symbol;Acc:259]
ENSG00000134107	1.94766	8.79137	26.35337	2.84E-07	0.000558	BHLHE40	basic helix-loop-helix family, member e40 [Source:HGNC Symbol;Acc:1046]
ENSG00000167772	3.57709	4.09445	25.41347	4.63E-07	0.000661	ANGPTL4	angiopoietin-like 4 [Source:HGNC Symbol;Acc:16039]
ENSG00000172061	2.14974	2.54563	25.50311	4.42E-07	0.000661	LRRC15	leucine rich repeat containing 15 [Source:HGNC Symbol;Acc:20818]
ENSG00000166741	2.53745	5.48180	24.33523	8.09E-07	0.000978	NNMT	nicotinamide N-methyltransferase [Source:HGNC Symbol;Acc:7861]
ENSG00000163661	4.63690	4.79708	22.83057	1.77E-06	0.001829	PTX3	pentraxin 3, long [Source:HGNC Symbol;Acc:9692]
ENSG00000158859	4.25508	6.27463	21.71486	3.16E-06	0.002761	ADAMTS4	ADAM metallopeptidase with thrombospondin type 1 motif, 4 [Source:HGNC Symbol;Acc:220]
ENSG00000017427	1.88111	5.54702	20.61660	5.61E-06	0.004197	IGF1	insulin-like growth factor 1 (somatomedin C) [Source:HGNC Symbol;Acc:5464]
ENSG00000123496	4.28850	0.16830	20.46727	6.07E-06	0.004331	IL13RA2	interleukin 13 receptor, alpha 2 [Source:HGNC Symbol;Acc:5975]
ENSG00000133048	2.50974	5.54410	20.33529	6.50E-06	0.004439	CHI3L1	chitinase 3-like 1 (cartilage glycoprotein-39) [Source:HGNC Symbol;Acc:1932]
ENSG00000162692	2.36663	5.78648	20.22100	6.90E-06	0.004515	VCAM1	vascular cell adhesion molecule 1 [Source:HGNC Symbol;Acc:12663]
ENSG00000162711	2.15092	3.73298	20.06512	7.48E-06	0.004703	NLRP3	NLR family, pyrin domain containing 3 [Source:HGNC Symbol;Acc:16400]
ENSG00000151790	2.93302	1.64327	19.74117	8.87E-06	0.005357	TDO2	tryptophan 2,3-dioxygenase [Source:HGNC Symbol;Acc:11708]
ENSG00000141433	6.99457	2.74555	19.53261	9.89E-06	0.005477	ADCYAP1	adenylate cyclase activating polypeptide 1 (pituitary) [Source:HGNC Symbol;Acc:241]
ENSG00000124216	2.30620	3.77795	19.49035	1.01E-05	0.005477	SNAI1	snail family zinc finger 1 [Source:HGNC Symbol;Acc:11128]
ENSG00000187955	1.73106	6.95228	19.52663	9.92E-06	0.005477	COL14A1	collagen, type XIV, alpha 1 [Source:HGNC Symbol;Acc:2191]
ENSG00000137801	2.96959	10.22978	18.91707	1.37E-05	0.006178	THBS1	thrombospondin 1 [Source:HGNC Symbol;Acc:11785]
ENSG00000184557	2.82837	8.18633	18.93596	1.35E-05	0.006178	SOCS3	suppressor of cytokine signaling 3 [Source:HGNC Symbol;Acc:19391]
ENSG00000141682	2.01397	3.08060	18.95506	1.34E-05	0.006178	PMAIP1	phorbol-12-myristate-13-acetate-induced protein 1 [Source:HGNC Symbol;Acc:9108]
ENSG00000280143	1.48059	4.99889	18.79537	1.46E-05	0.006178	NA	NA
ENSG00000113083	1.21589	5.46295	18.87161	1.40E-05	0.006178	LOX	lysyl oxidase [Source:HGNC Symbol;Acc:6664]
ENSG00000102359	1.33735	3.29612	18.54783	1.66E-05	0.006849	SRPX2	sushi-repeat containing protein, X-linked 2 [Source:HGNC Symbol;Acc:30668]
ENSG00000105835	2.07754	9.28693	18.27169	1.92E-05	0.007554	NAMPT	nicotinamide phosphoribosyltransferase [Source:HGNC Symbol;Acc:30092]
ENSG00000104415	3.06844	3.44419	18.10769	2.09E-05	0.007610	WISP1	WNT1 inducible signaling pathway protein 1 [Source:HGNC Symbol;Acc:12769]
ENSG00000211942	3.00227	2.51402	18.06802	2.13E-05	0.007610	IGHV3-13	immunoglobulin heavy variable 3-13 [Source:HGNC Symbol;Acc:5581]
ENSG00000103196	2.24939	7.57113	18.20202	1.99E-05	0.007610	CRISPLD2	cysteine-rich secretory protein LCCL domain containing 2 [Source:HGNC Symbol;Acc:25248]
ENSG00000176692	1.61669	2.33406	18.12911	2.06E-05	0.007610	FOXC2	forkhead box C2 (MFH-1, mesenchyme forkhead 1) [Source:HGNC Symbol;Acc:3801]
ENSG00000186340	1.91426	5.31158	18.01316	2.19E-05	0.007658	THBS2	thrombospondin 2 [Source:HGNC Symbol;Acc:11786]
ENSG00000157214	2.06353	6.06279	17.84748	2.39E-05	0.008173	STEAP2	STEAP family member 2, metalloreductase [Source:HGNC Symbol;Acc:17885]
ENSG00000108691	3.23126	7.32024	17.44134	2.96E-05	0.008951	CCL2	chemokine (C-C motif) ligand 2 [Source:HGNC Symbol;Acc:10618]
ENSG00000137193	1.68775	6.07551	17.45702	2.94E-05	0.008951	PIM1	pim-1 oncogene [Source:HGNC Symbol;Acc:8986]
ENSG00000100644	1.64627	8.29976	17.53312	2.82E-05	0.008951	HIF1A	hypoxia inducible factor 1, alpha subunit (basic helix-loop-helix transcription factor) [Source:HGNC Symbol;Acc:4910]
ENSG00000164647	2.04834	2.64174	17.37376	3.07E-05	0.009100	STEAP1	six transmembrane epithelial antigen of the prostate 1 [Source:HGNC Symbol;Acc:11378]
ENSG00000112936	1.64310	8.52758	17.27891	3.23E-05	0.009302	C7	complement component 7 [Source:HGNC Symbol;Acc:1346]
ENSG00000175592	4.01599	5.02631	17.12491	3.50E-05	0.009494	FOSL1	FOS-like antigen 1 [Source:HGNC Symbol;Acc:13718]
ENSG00000163739	3.31244	5.60788	17.08943	3.57E-05	0.009494	CXCL1	chemokine (C-X-C motif) ligand 1 (melanoma growth stimulating activity, alpha) [Source:HGNC Symbol;Acc:4602]
ENSG00000182752	2.37031	4.43087	17.07801	3.59E-05	0.009494	PAPPA	pregnancy-associated plasma protein A, pappalysin 1 [Source:HGNC Symbol;Acc:8602]
ENSG00000151014	2.32929	2.73703	17.05743	3.63E-05	0.009494	CCRN4L	CCR4 carbon catabolite repression 4-like (S. cerevisiae) [Source:HGNC Symbol;Acc:14254]
ENSG00000185022	2.15545	5.75667	17.14028	3.47E-05	0.009494	MAFF	v-maf avian musculoaponeurotic fibrosarcoma oncogene homolog F [Source:HGNC Symbol;Acc:6780]
ENSG00000169429	4.12921	5.96401	16.97320	3.79E-05	0.009762	IL8	interleukin 8 [Source:HGNC Symbol;Acc:6025]
ENSG00000136244	3.90898	7.10357	16.75382	4.26E-05	0.010282	IL6	interleukin 6 (interferon, beta 2) [Source:HGNC Symbol;Acc:6018]
ENSG00000007908	3.41265	4.99855	16.74920	4.27E-05	0.010282	SELE	selectin E [Source:HGNC Symbol;Acc:10718]
ENSG00000188766	2.88636	1.27657	16.55399	4.73E-05	0.010282	SPRED3	sprouty-related, EVH1 domain containing 3 [Source:HGNC Symbol;Acc:31041]
ENSG00000178860	2.19983	2.81309	16.50821	4.84E-05	0.010282	MSC	musculin [Source:HGNC Symbol;Acc:7321]
ENSG00000115641	2.09482	3.98450	16.54332	4.76E-05	0.010282	FHL2	four and a half LIM domains 2 [Source:HGNC Symbol;Acc:3703]
ENSG00000165030	1.93433	5.88034	16.52314	4.81E-05	0.010282	NFIL3	nuclear factor, interleukin 3 regulated [Source:HGNC Symbol;Acc:7787]
ENSG00000116717	2.12424	5.65356	16.40922	5.10E-05	0.010411	GADD45A	growth arrest and DNA-damage-inducible, alpha [Source:HGNC Symbol;Acc:4095]
ENSG00000123610	2.04916	2.49775	16.42333	5.07E-05	0.010411	TNFAIP6	tumor necrosis factor, alpha-induced protein 6 [Source:HGNC Symbol;Acc:11898]
ENSG00000174348	1.32502	6.34457	16.36734	5.22E-05	0.010508	PODN	podocan [Source:HGNC Symbol;Acc:23174]
ENSG00000185274	1.51910	3.12744	16.30938	5.38E-05	0.010697	WBSCR17	Williams-Beuren syndrome chromosome region 17 [Source:HGNC Symbol;Acc:16347]
ENSG00000229644	3.14233	0.94639	16.24757	5.56E-05	0.010913	NAMPTL	nicotinamide phosphoribosyltransferase-like [Source:HGNC Symbol;Acc:17633]
ENSG00000124762	2.22944	6.85426	16.18125	5.76E-05	0.011163	CDKN1A	cyclin-dependent kinase inhibitor 1A (p21, Cip1) [Source:HGNC Symbol;Acc:1784]
ENSG00000136997	2.40111	6.78395	16.14128	5.88E-05	0.011262	MYC	v-myc avian myelocytomatosis viral oncogene homolog [Source:HGNC Symbol;Acc:7553]
ENSG00000147647	2.97186	0.77622	16.05677	6.15E-05	0.011634	DPYS	dihydropyrimidinase [Source:HGNC Symbol;Acc:3013]
ENSG00000137558	2.91362	2.89473	15.84608	6.87E-05	0.012127	PI15	peptidase inhibitor 15 [Source:HGNC Symbol;Acc:8946]
ENSG00000103888	2.08412	4.28584	15.92780	6.58E-05	0.012127	KIAA1199	KIAA1199 [Source:HGNC Symbol;Acc:29213]
ENSG00000057657	1.85009	5.92084	15.86008	6.82E-05	0.012127	PRDM1	PR domain containing 1, with ZNF domain [Source:HGNC Symbol;Acc:9346]
ENSG00000150347	1.26705	7.62913	15.88913	6.72E-05	0.012127	ARID5B	AT rich interactive domain 5B (MRF1-like) [Source:HGNC Symbol;Acc:17362]
ENSG00000090104	2.38247	6.19202	15.74658	7.24E-05	0.012519	RGS1	regulator of G-protein signaling 1 [Source:HGNC Symbol;Acc:9991]
ENSG00000187479	2.06247	6.89142	15.69476	7.44E-05	0.012519	C11orf96	chromosome 11 open reading frame 96 [Source:HGNC Symbol;Acc:38675]
ENSG00000124772	1.89584	2.60777	15.68251	7.49E-05	0.012519	CPNE5	copine V [Source:HGNC Symbol;Acc:2318]
ENSG00000184588	1.67777	5.90214	15.74051	7.27E-05	0.012519	PDE4B	phosphodiesterase 4B, cAMP-specific [Source:HGNC Symbol;Acc:8781]
ENSG00000137573	1.47661	5.93783	15.70383	7.41E-05	0.012519	SULF1	sulfatase 1 [Source:HGNC Symbol;Acc:20391]
ENSG00000172548	4.22509	0.96997	15.46461	8.41E-05	0.013755	NIPAL4	NIPA-like domain containing 4 [Source:HGNC Symbol;Acc:28018]
ENSG00000122121	2.09503	1.06883	15.47504	8.36E-05	0.013755	XPNPEP2	X-prolyl aminopeptidase (aminopeptidase P) 2, membrane-bound [Source:HGNC Symbol;Acc:12823]
ENSG00000108700	2.86214	2.02288	15.38336	8.78E-05	0.014066	CCL8	chemokine (C-C motif) ligand 8 [Source:HGNC Symbol;Acc:10635]
ENSG00000115009	3.01211	4.55039	15.27509	9.29E-05	0.014376	CCL20	chemokine (C-C motif) ligand 20 [Source:HGNC Symbol;Acc:10619]
ENSG00000106366	2.65137	7.57336	15.29729	9.18E-05	0.014376	SERPINE1	serpin peptidase inhibitor, clade E (nexin, plasminogen activator inhibitor type 1), member 1 [Source:HGNC Symbol;Acc:8583]
ENSG00000173276	1.24195	5.92132	15.18983	9.72E-05	0.014827	ZBTB21	zinc finger and BTB domain containing 21 [Source:HGNC Symbol;Acc:13083]
ENSG00000112562	1.62636	5.24320	15.01947	0.000106	0.015683	SMOC2	SPARC related modular calcium binding 2 [Source:HGNC Symbol;Acc:20323]
ENSG00000171560	3.37459	3.77409	14.96535	0.000110	0.015927	FGA	fibrinogen alpha chain [Source:HGNC Symbol;Acc:3661]
ENSG00000184956	6.36009	2.51086	14.88945	0.000114	0.015985	MUC6	mucin 6, oligomeric mucus/gel-forming [Source:HGNC Symbol;Acc:7517]
ENSG00000196136	4.56901	2.44816	14.83998	0.000117	0.015985	SERPINA3	serpin peptidase inhibitor, clade A (alpha-1 antiproteinase, antitrypsin), member 3 [Source:HGNC Symbol;Acc:16]
ENSG00000170961	2.56619	5.18239	14.85226	0.000116	0.015985	HAS2	hyaluronan synthase 2 [Source:HGNC Symbol;Acc:4819]
ENSG00000137331	2.29458	5.33819	14.84231	0.000117	0.015985	IER3	immediate early response 3 [Source:HGNC Symbol;Acc:5392]
ENSG00000125148	2.26836	7.69790	14.87769	0.000115	0.015985	MT2A	metallothionein 2A [Source:HGNC Symbol;Acc:7406]
ENSG00000213694	1.47282	5.17540	14.85354	0.000116	0.015985	S1PR3	sphingosine-1-phosphate receptor 3 [Source:HGNC Symbol;Acc:3167]
ENSG00000151917	2.03054	1.03659	14.80956	0.000119	0.016105	BEND6	BEN domain containing 6 [Source:HGNC Symbol;Acc:20871]
ENSG00000272636	1.20679	4.10180	14.78188	0.000121	0.016126	DOC2B	double C2-like domains, beta [Source:HGNC Symbol;Acc:2986]
ENSG00000133063	3.68660	4.12134	14.73074	0.000124	0.016270	CHIT1	chitinase 1 (chitotriosidase) [Source:HGNC Symbol;Acc:1936]
ENSG00000163520	1.27503	5.44396	14.72640	0.000124	0.016270	FBLN2	fibulin 2 [Source:HGNC Symbol;Acc:3601]
ENSG00000280411	2.99807	3.58603	14.68427	0.000127	0.016500	NA	NA
ENSG00000171223	1.91345	8.56827	14.63683	0.000130	0.016501	JUNB	jun B proto-oncogene [Source:HGNC Symbol;Acc:6205]
ENSG00000064300	1.79516	2.82751	14.62953	0.000131	0.016501	NGFR	nerve growth factor receptor [Source:HGNC Symbol;Acc:7809]
ENSG00000146555	1.23186	5.26254	14.62289	0.000131	0.016501	SDK1	sidekick cell adhesion molecule 1 [Source:HGNC Symbol;Acc:19307]
ENSG00000110848	1.72638	4.43865	14.59040	0.000134	0.016655	CD69	CD69 molecule [Source:HGNC Symbol;Acc:1694]
ENSG00000235531	1.68531	2.65199	14.53392	0.000138	0.017026	RP11-383H13.1	Protein LOC100132891; cDNA FLJ53548 [Source:UniProtKB/TrEMBL;Acc:B7Z945]
ENSG00000275063	3.48237	2.08280	14.39476	0.000148	0.017773	NA	NA
ENSG00000126368	2.25272	5.34339	14.35501	0.000151	0.017773	NR1D1	nuclear receptor subfamily 1, group D, member 1 [Source:HGNC Symbol;Acc:7962]
ENSG00000162433	1.46688	3.13489	14.33801	0.000153	0.017773	AK4	adenylate kinase 4 [Source:HGNC Symbol;Acc:363]
ENSG00000101825	1.12546	6.72023	14.35152	0.000152	0.017773	MXRA5	matrix-remodelling associated 5 [Source:HGNC Symbol;Acc:7539]
ENSG00000178878	2.68363	6.37093	14.31591	0.000155	0.017851	APOLD1	apolipoprotein L domain containing 1 [Source:HGNC Symbol;Acc:25268]
ENSG00000139144	4.68976	1.89654	14.28209	0.000157	0.017911	PIK3C2G	phosphatidylinositol-4-phosphate 3-kinase, catalytic subunit type 2 gamma [Source:HGNC Symbol;Acc:8973]
ENSG00000218336	1.95895	3.50527	14.28808	0.000157	0.017911	TENM3	teneurin transmembrane protein 3 [Source:HGNC Symbol;Acc:29944]
ENSG00000049323	0.78373	6.80279	14.11069	0.000172	0.018933	LTBP1	latent transforming growth factor beta binding protein 1 [Source:HGNC Symbol;Acc:6714]
ENSG00000086544	1.96489	5.86051	13.95427	0.000187	0.020016	ITPKC	inositol-trisphosphate 3-kinase C [Source:HGNC Symbol;Acc:14897]
ENSG00000146072	1.20716	5.99932	13.81385	0.000202	0.021079	TNFRSF21	tumor necrosis factor receptor superfamily, member 21 [Source:HGNC Symbol;Acc:13469]
ENSG00000172572	1.12828	5.06683	13.78180	0.000205	0.021079	PDE3A	phosphodiesterase 3A, cGMP-inhibited [Source:HGNC Symbol;Acc:8778]
ENSG00000100979	1.02308	5.21173	13.78427	0.000205	0.021079	PLTP	phospholipid transfer protein [Source:HGNC Symbol;Acc:9093]
ENSG00000103257	2.13943	4.33284	13.75861	0.000208	0.021129	SLC7A5	solute carrier family 7 (amino acid transporter light chain, L system), member 5 [Source:HGNC Symbol;Acc:11063]
ENSG00000138166	2.01732	4.52638	13.74829	0.000209	0.021129	DUSP5	dual specificity phosphatase 5 [Source:HGNC Symbol;Acc:3071]
ENSG00000090659	1.24760	3.52095	13.74085	0.000210	0.021129	CD209	CD209 molecule [Source:HGNC Symbol;Acc:1641]
ENSG00000115590	2.39777	1.95960	13.69378	0.000215	0.021391	IL1R2	interleukin 1 receptor, type II [Source:HGNC Symbol;Acc:5994]
ENSG00000162458	1.17806	5.39969	13.60994	0.000225	0.022089	FBLIM1	filamin binding LIM protein 1 [Source:HGNC Symbol;Acc:24686]
ENSG00000240864	2.99907	3.20922	13.55039	0.000232	0.022267	IGKV1-16	immunoglobulin kappa variable 1-16 [Source:HGNC Symbol;Acc:5732]
ENSG00000119508	2.56979	6.90160	13.54846	0.000232	0.022267	NR4A3	nuclear receptor subfamily 4, group A, member 3 [Source:HGNC Symbol;Acc:7982]
ENSG00000091986	1.67629	8.01627	13.56312	0.000231	0.022267	CCDC80	coiled-coil domain containing 80 [Source:HGNC Symbol;Acc:30649]
ENSG00000102265	1.45161	7.08591	13.46734	0.000243	0.022484	TIMP1	TIMP metallopeptidase inhibitor 1 [Source:HGNC Symbol;Acc:11820]
ENSG00000176531	1.39849	2.48360	13.46285	0.000243	0.022484	PHLDB3	pleckstrin homology-like domain, family B, member 3 [Source:HGNC Symbol;Acc:30499]
ENSG00000178031	1.39262	2.54205	13.48141	0.000241	0.022484	ADAMTSL1	ADAMTS-like 1 [Source:HGNC Symbol;Acc:14632]

DE mRNA comb FDR 0.05 up

Ensembl gene	logFC	logCPM	LR	PValue	adj.p	gene	Description
ENSG00000119699	1.32857	4.40318	13.49076	0.000240	0.022484	TGFB3	transforming growth factor, beta 3 [Source:HGNC Symbol;Acc:11769]
ENSG00000166147	1.03778	7.23359	13.50449	0.000238	0.022484	FBN1	fibrillin 1 [Source:HGNC Symbol;Acc:3603]
ENSG00000270607	3.17813	0.49564	13.43923	0.000246	0.022504	RP11-359E10.1	
ENSG00000196611	4.38243	2.27941	13.41045	0.000250	0.022589	MMP1	matrix metallopeptidase 1 (interstitial collagenase) [Source:HGNC Symbol;Acc:7155]
ENSG00000111912	1.48623	7.97399	13.41714	0.000249	0.022589	NCOA7	nuclear receptor coactivator 7 [Source:HGNC Symbol;Acc:21081]
ENSG00000163599	1.79729	1.77830	13.35767	0.000257	0.022924	CTLA4	cytotoxic T-lymphocyte-associated protein 4 [Source:HGNC Symbol;Acc:2505]
ENSG00000112096	1.27700	9.12382	13.34026	0.000260	0.022924	SOD2	superoxide dismutase 2, mitochondrial [Source:HGNC Symbol;Acc:11180]
ENSG00000148841	1.22682	6.40856	13.25126	0.000272	0.023886	ITPRIP	inositol 1,4,5-trisphosphate receptor interacting protein [Source:HGNC Symbol;Acc:29370]
ENSG00000160862	2.48352	1.99589	13.22681	0.000276	0.023950	AZGP1	alpha-2-glycoprotein 1, zinc-binding [Source:HGNC Symbol;Acc:910]
ENSG00000178607	1.22929	5.68529	13.20652	0.000279	0.024078	ERN1	endoplasmic reticulum to nucleus signaling 1 [Source:HGNC Symbol;Acc:3449]
ENSG00000221866	1.23108	3.52335	13.18700	0.000282	0.024107	PLXNA4	plexin A4 [Source:HGNC Symbol;Acc:9102]
ENSG00000054598	1.93191	4.98813	13.16565	0.000285	0.024210	FOXC1	forkhead box C1 [Source:HGNC Symbol;Acc:3800]
ENSG00000168421	1.41515	3.20004	13.11782	0.000292	0.024570	RHOH	ras homolog family member H [Source:HGNC Symbol;Acc:686]
ENSG00000128342	2.84416	5.41639	13.00533	0.000311	0.025815	LIF	leukemia inhibitory factor [Source:HGNC Symbol;Acc:6596]
ENSG00000140511	1.42217	3.02868	12.91578	0.000326	0.026937	HAPLN3	hyaluronan and proteoglycan link protein 3 [Source:HGNC Symbol;Acc:21446]
ENSG00000171724	2.20078	1.03051	12.89711	0.000329	0.026946	VAT1L	vesicle amine transport 1-like [Source:HGNC Symbol;Acc:29315]
ENSG00000144655	1.82448	6.65370	12.89556	0.000329	0.026946	CSRNP1	cysteine-serine-rich nuclear protein 1 [Source:HGNC Symbol;Acc:14300]
ENSG00000077274	2.03654	1.45591	12.87628	0.000333	0.027084	CAPN6	calpain 6 [Source:HGNC Symbol;Acc:1483]
ENSG00000053702	1.82759	1.14100	12.85212	0.000337	0.027294	NRIP2	nuclear receptor interacting protein 2 [Source:HGNC Symbol;Acc:23078]
ENSG00000115461	1.00860	8.61729	12.82946	0.000341	0.027485	IGFBP5	insulin-like growth factor binding protein 5 [Source:HGNC Symbol;Acc:5474]
ENSG00000134460	1.89714	2.94855	12.71156	0.000363	0.028994	IL2RA	interleukin 2 receptor, alpha [Source:HGNC Symbol;Acc:6008]
ENSG00000155380	1.25733	3.57673	12.71044	0.000364	0.028994	SLC16A1	solute carrier family 16 (monocarboxylate transporter), member 1 [Source:HGNC Symbol;Acc:10922]
ENSG00000154734	2.06860	7.71059	12.67945	0.000370	0.029217	ADAMTS1	ADAM metallopeptidase with thrombospondin type 1 motif, 1 [Source:HGNC Symbol;Acc:217]
ENSG00000169071	1.56928	2.98386	12.67603	0.000370	0.029217	ROR2	receptor tyrosine kinase-like orphan receptor 2 [Source:HGNC Symbol;Acc:10257]
ENSG00000116132	1.20866	6.14234	12.66784	0.000372	0.029217	PRRX1	paired related homeobox 1 [Source:HGNC Symbol;Acc:9142]
ENSG00000134531	1.74395	8.88782	12.57079	0.000392	0.030319	EMP1	epithelial membrane protein 1 [Source:HGNC Symbol;Acc:3333]
ENSG00000167034	2.44955	3.55456	12.52535	0.000401	0.030726	NKX3-1	NK3 homeobox 1 [Source:HGNC Symbol;Acc:7838]
ENSG00000128016	2.10952	10.17545	12.50289	0.000406	0.030726	ZFP36	ZFP36 ring finger protein [Source:HGNC Symbol;Acc:12862]
ENSG00000275894	2.05303	0.90055	12.52609	0.000401	0.030726	NA	NA
ENSG00000174705	1.03119	5.52508	12.50042	0.000407	0.030726	SH3PXD2B	SH3 and PX domains 2B [Source:HGNC Symbol;Acc:29242]
ENSG00000176678	1.67042	1.57214	12.46577	0.000414	0.031003	FOXL1	forkhead box L1 [Source:HGNC Symbol;Acc:3817]
ENSG00000158050	1.86748	2.97524	12.41110	0.000427	0.031595	DUSP2	dual specificity phosphatase 2 [Source:HGNC Symbol;Acc:3068]
ENSG00000124466	1.78893	1.29060	12.41961	0.000425	0.031595	LYPD3	LY6/PLAUR domain containing 3 [Source:HGNC Symbol;Acc:24880]
ENSG00000221869	1.23158	7.62104	12.37821	0.000434	0.031884	CEBPD	CCAAT/enhancer binding protein (C/EBP), delta [Source:HGNC Symbol;Acc:1835]
ENSG00000124813	0.93546	4.22265	12.32791	0.000446	0.032333	RUNX2	runt-related transcription factor 2 [Source:HGNC Symbol;Acc:10472]
ENSG00000115594	1.05719	8.71549	12.29443	0.000454	0.032735	IL1R1	interleukin 1 receptor, type I [Source:HGNC Symbol;Acc:5993]
ENSG00000253276	1.49333	4.73142	12.28460	0.000457	0.032758	CCDC71L	coiled-coil domain containing 71-like [Source:HGNC Symbol;Acc:26685]
ENSG00000164318	1.58950	2.16823	12.22226	0.000472	0.033497	EGFLAM	EGF-like, fibronectin type III and laminin G domains [Source:HGNC Symbol;Acc:26810]
ENSG00000168334	3.92335	1.99704	12.10458	0.000503	0.034931	XIRP1	xin actin-binding repeat containing 1 [Source:HGNC Symbol;Acc:14301]
ENSG00000152377	1.93420	2.83709	12.09787	0.000505	0.034931	SPOCK1	sparc/osteonectin, cwcv and kazal-like domains proteoglycan (testican) 1 [Source:HGNC Symbol;Acc:11251]
ENSG00000196843	1.52085	5.73321	12.06792	0.000513	0.035193	ARID5A	AT rich interactive domain 5A (MRF1-like) [Source:HGNC Symbol;Acc:17361]
ENSG00000151892	1.11246	5.01076	12.06759	0.000513	0.035193	GFRA1	GDNF family receptor alpha 1 [Source:HGNC Symbol;Acc:4243]
ENSG00000075426	1.75485	8.25525	12.04064	0.000521	0.035550	FOSL2	FOS-like antigen 2 [Source:HGNC Symbol;Acc:3798]
ENSG00000123500	2.86865	2.41021	11.99261	0.000534	0.036008	COL10A1	collagen, type X, alpha 1 [Source:HGNC Symbol;Acc:2185]
ENSG00000134443	4.24415	7.44585	11.95381	0.000545	0.036609	GRP	gastrin-releasing peptide [Source:HGNC Symbol;Acc:4605]
ENSG00000145623	1.02575	7.46487	11.90923	0.000559	0.036913	OSMR	oncostatin M receptor [Source:HGNC Symbol;Acc:8507]
ENSG00000054803	3.66741	0.69675	11.88490	0.000566	0.037194	CBLN4	cerebellin 4 precursor [Source:HGNC Symbol;Acc:16231]
ENSG00000184731	1.18518	3.61468	11.86351	0.000572	0.037397	FAM110C	family with sequence similarity 110, member C [Source:HGNC Symbol;Acc:33340]
ENSG00000106809	1.32990	5.76436	11.84844	0.000577	0.037459	OGN	osteoglycin [Source:HGNC Symbol;Acc:8126]
ENSG00000151012	1.39101	2.97448	11.77545	0.000600	0.038637	SLC7A11	solute carrier family 7 (anionic amino acid transporter light chain, xc- system), member 11 [Source:HGNC Symbol;Acc:11059]
ENSG00000251348	2.80365	0.41699	11.73969	0.000612	0.038833	HSPD1P11	heat shock 60kDa protein 1 (chaperonin) pseudogene 11 [Source:HGNC Symbol;Acc:35142]
ENSG00000138135	2.53026	4.01963	11.71588	0.000620	0.038833	CH25H	cholesterol 25-hydroxylase [Source:HGNC Symbol;Acc:1907]
ENSG00000135069	2.25434	1.11560	11.71339	0.000621	0.038833	PSAT1	phosphoserine aminotransferase 1 [Source:HGNC Symbol;Acc:19129]
ENSG00000171621	1.99337	6.00924	11.71662	0.000619	0.038833	SPSB1	splA/ryanodine receptor domain and SOCS box containing 1 [Source:HGNC Symbol;Acc:30628]
ENSG00000273604	1.96251	1.88698	11.68570	0.000630	0.039104	NA	NA
ENSG00000148948	1.75336	2.17366	11.67399	0.000634	0.039196	LRRC4C	leucine rich repeat containing 4C [Source:HGNC Symbol;Acc:29317]
ENSG00000131873	1.31607	6.42325	11.61578	0.000654	0.040283	CHSY1	chondroitin sulfate synthase 1 [Source:HGNC Symbol;Acc:17198]
ENSG00000205420	7.34982	2.54470	11.56142	0.000673	0.040399	KRT6A	keratin 6A [Source:HGNC Symbol;Acc:6443]
ENSG00000163874	2.44096	5.86879	11.55104	0.000677	0.040399	ZC3H12A	zinc finger CCCH-type containing 12A [Source:HGNC Symbol;Acc:26259]
ENSG00000172724	1.95397	3.29850	11.55921	0.000674	0.040399	CCL19	chemokine (C-C motif) ligand 19 [Source:HGNC Symbol;Acc:10617]
ENSG00000148339	1.78313	5.60394	11.54750	0.000678	0.040399	SLC25A25	solute carrier family 25 (mitochondrial carrier; phosphate carrier), member 25 [Source:HGNC Symbol;Acc:20663]
ENSG00000121966	1.58279	5.87810	11.58527	0.000665	0.040399	CXCR4	chemokine (C-X-C motif) receptor 4 [Source:HGNC Symbol;Acc:2561]
ENSG00000065911	1.54419	4.71333	11.54593	0.000679	0.040399	MTHFD2	methylenetetrahydrofolate dehydrogenase (NADP+ dependent) 2, methenyltetrahydrofolate cyclohydrolase [Source:HGNC Symbol;Acc:7434]
ENSG00000138061	1.47860	6.16185	11.58066	0.000666	0.040399	CYP1B1	cytochrome P450, family 1, subfamily B, polypeptide 1 [Source:HGNC Symbol;Acc:2597]
ENSG00000110680	4.93078	3.99998	11.44553	0.000717	0.042321	CALCA	calcitonin-related polypeptide alpha [Source:HGNC Symbol;Acc:1437]
ENSG00000131459	2.34992	4.56906	11.40761	0.000731	0.043032	GFPT2	glutamine-fructose-6-phosphate transaminase 2 [Source:HGNC Symbol;Acc:4242]
ENSG00000165507	1.77846	8.53285	11.32512	0.000765	0.044322	C10orf10	chromosome 10 open reading frame 10 [Source:HGNC Symbol;Acc:23355]
ENSG00000205362	2.81773	3.77660	11.30580	0.000773	0.044402	MT1A	metallothionein 1A [Source:HGNC Symbol;Acc:7393]
ENSG00000131711	1.51644	7.27115	11.30133	0.000775	0.044402	MAP1B	microtubule-associated protein 1B [Source:HGNC Symbol;Acc:6836]
ENSG00000172817	1.10464	2.94335	11.30361	0.000774	0.044402	CYP7B1	cytochrome P450, family 7, subfamily B, polypeptide 1 [Source:HGNC Symbol;Acc:2652]
ENSG00000169508	1.84737	5.00255	11.27908	0.000784	0.044611	GPR183	G protein-coupled receptor 183 [Source:HGNC Symbol;Acc:3128]
ENSG00000145362	1.80290	6.14064	11.28035	0.000783	0.044611	ANK2	ankyrin 2, neuronal [Source:HGNC Symbol;Acc:493]
ENSG00000130037	1.37375	2.29699	11.25152	0.000796	0.044933	KCNA5	potassium voltage-gated channel, shaker-related subfamily, member 5 [Source:HGNC Symbol;Acc:6224]
ENSG00000008056	2.25382	2.62906	11.23221	0.000804	0.044989	SYN1	synapsin I [Source:HGNC Symbol;Acc:11494]
ENSG00000230673	1.72959	0.81842	11.17879	0.000827	0.045550	PABPC1P3	poly(A) binding protein, cytoplasmic 1 pseudogene 3 [Source:HGNC Symbol;Acc:8560]
ENSG00000069399	1.61388	6.27719	11.17440	0.000829	0.045550	BCL3	B-cell CLL/lymphoma 3 [Source:HGNC Symbol;Acc:998]
ENSG00000162924	1.47150	6.37958	11.14608	0.000842	0.045572	REL	v-rel avian reticuloendotheliosis viral oncogene homolog [Source:HGNC Symbol;Acc:9954]
ENSG00000140285	1.46996	6.31497	11.16497	0.000834	0.045572	FGF7	fibroblast growth factor 7 [Source:HGNC Symbol;Acc:3685]
ENSG00000149256	1.41991	3.35110	11.10063	0.000863	0.046415	TENM4	teneurin transmembrane protein 4 [Source:HGNC Symbol;Acc:29945]
ENSG00000167470	1.32106	7.60879	11.09466	0.000866	0.046415	MIDN	midnolin [Source:HGNC Symbol;Acc:16298]
ENSG00000118985	1.39741	7.04448	11.08594	0.000870	0.046475	ELL2	elongation factor, RNA polymerase II, 2 [Source:HGNC Symbol;Acc:17064]
ENSG00000091879	1.16733	4.58683	11.07830	0.000873	0.046508	ANGPT2	angiopoietin 2 [Source:HGNC Symbol;Acc:485]
ENSG00000003987	2.30834	2.60351	11.05775	0.000883	0.046623	MTMR7	myotubularin related protein 7 [Source:HGNC Symbol;Acc:7454]
ENSG00000161960	1.94774	1.95922	11.04448	0.000890	0.046623	EIF4A1	eukaryotic translation initiation factor 4A1 [Source:HGNC Symbol;Acc:3282]
ENSG00000222041	1.51426	3.76558	11.04257	0.000890	0.046623	LINC00152	long intergenic non-protein coding RNA 152 [Source:HGNC Symbol;Acc:28717]
ENSG00000205364	2.65191	4.49950	11.00429	0.000909	0.046890	MT1M	metallothionein 1M [Source:HGNC Symbol;Acc:14296]
ENSG00000167941	2.48993	0.66407	10.97790	0.000922	0.046890	SOST	sclerostin [Source:HGNC Symbol;Acc:13771]
ENSG00000205502	2.36492	3.48337	11.00445	0.000909	0.046890	C2CD4B	C2 calcium-dependent domain containing 4B [Source:HGNC Symbol;Acc:33628]
ENSG00000081041	2.30551	6.75988	10.96524	0.000928	0.046890	CXCL2	chemokine (C-X-C motif) ligand 2 [Source:HGNC Symbol;Acc:4603]
ENSG00000211950	2.17215	3.00643	10.97162	0.000925	0.046890	IGHV1-24	immunoglobulin heavy variable 1-24 [Source:HGNC Symbol;Acc:5551]
ENSG00000109265	1.38957	2.59990	10.96968	0.000926	0.046890	KIAA1211	KIAA1211 [Source:HGNC Symbol;Acc:29219]
ENSG00000277632	2.38488	2.55016	10.95053	0.000936	0.046895	NA	NA
ENSG00000188257	2.35880	4.34638	10.93540	0.000943	0.046895	PLA2G2A	phospholipase A2, group IIA (platelets, synovial fluid) [Source:HGNC Symbol;Acc:9031]
ENSG00000162772	2.01491	6.14149	10.94618	0.000938	0.046895	ATF3	activating transcription factor 3 [Source:HGNC Symbol;Acc:785]
ENSG00000172738	2.43298	1.10037	10.88391	0.000970	0.047006	TMEM217	transmembrane protein 217 [Source:HGNC Symbol;Acc:21238]
ENSG00000104213	1.93417	2.08765	10.90468	0.000959	0.047006	PDGFRL	platelet-derived growth factor receptor-like [Source:HGNC Symbol;Acc:8805]
ENSG00000189056	1.42705	3.37627	10.88741	0.000968	0.047006	RELN	reelin [Source:HGNC Symbol;Acc:9957]
ENSG00000170485	1.17907	4.36847	10.87910	0.000973	0.047006	NPAS2	neuronal PAS domain protein 2 [Source:HGNC Symbol;Acc:7895]
ENSG00000158615	1.00763	6.79229	10.88308	0.000970	0.047006	PPP1R15B	protein phosphatase 1, regulatory subunit 15B [Source:HGNC Symbol;Acc:14951]
ENSG00000115598	2.46384	0.27140	10.86215	0.000981	0.047148	IL1RL2	interleukin 1 receptor-like 2 [Source:HGNC Symbol;Acc:5999]
ENSG00000104635	1.58208	4.79323	10.86713	0.000979	0.047148	SLC39A14	solute carrier family 39 (zinc transporter), member 14 [Source:HGNC Symbol;Acc:20858]
ENSG00000277856	3.21221	0.80493	10.81692	0.001006	0.047854	NA	NA
ENSG00000132510	1.57356	7.47395	10.81211	0.001008	0.047854	KDM6B	lysine (K)-specific demethylase 6B [Source:HGNC Symbol;Acc:29012]
ENSG00000101187	1.93606	3.39815	10.78114	0.001025	0.048224	SLCO4A1	solute carrier organic anion transporter family, member 4A1 [Source:HGNC Symbol;Acc:10953]
ENSG00000230979	2.32960	0.65134	10.73009	0.001054	0.048622	AC079250.1	
ENSG00000179431	1.86449	2.04463	10.73335	0.001052	0.048622	FJX1	four jointed box 1 (Drosophila) [Source:HGNC Symbol;Acc:17166]
ENSG00000139289	1.80037	6.57526	10.72756	0.001056	0.048622	PHLDA1	pleckstrin homology-like domain, family A, member 1 [Source:HGNC Symbol;Acc:8933]
ENSG00000121152	1.59867	1.18347	10.74347	0.001046	0.048622	NCAPH	non-SMC condensin I complex, subunit H [Source:HGNC Symbol;Acc:1112]
ENSG00000196562	0.90278	5.60750	10.73984	0.001049	0.048622	SULF2	sulfatase 2 [Source:HGNC Symbol;Acc:20392]
ENSG00000106624	0.87682	8.04731	10.73465	0.001051	0.048622	AEBP1	AE binding protein 1 [Source:HGNC Symbol;Acc:303]
ENSG00000198901	0.98620	2.81357	10.64899	0.001101	0.049854	PRC1	protein regulator of cytokinesis 1 [Source:HGNC Symbol;Acc:9341]
ENSG00000119630	1.49019	1.44023	10.64255	0.001105	0.049884	PGF	placental growth factor [Source:HGNC Symbol;Acc:8893]
ENSG00000183578	1.26410	3.13138	10.63692	0.001109	0.049893	TNFAIP8L3	tumor necrosis factor, alpha-induced protein 8-like 3 [Source:HGNC Symbol;Acc:20620]

DE mRNA comb FDR 0.05 down

Ensembl gene	logFC	logCPM	LR	PValue	adj.p	gene	Description
ENSG00000231322	-3.07431	3.23551	58.28354	2.27E-14	3.56E-10	RPL13AP17	ribosomal protein L13a pseudogene 17 [Source:HGNC Symbol;Acc:36167]
ENSG00000186188	-2.65953	2.87334	34.69453	3.86E-09	3.03E-05	FFAR4	free fatty acid receptor 4 [Source:HGNC Symbol;Acc:19061]
ENSG00000183019	-2.13544	3.96816	27.04545	1.99E-07	0.000446	C19orf59	chromosome 19 open reading frame 59 [Source:HGNC Symbol;Acc:27291]
ENSG00000280099	-1.74596	3.04767	25.62358	4.15E-07	0.000661	NA	NA
ENSG00000227502	-2.11205	1.95524	24.52990	7.32E-07	0.000958	RP1-249H1.4	
ENSG00000205086	-2.60799	1.56772	23.75222	1.10E-06	0.001229	C2orf91	chromosome 2 open reading frame 91 [Source:HGNC Symbol;Acc:42966]
ENSG00000165490	-1.74827	1.98199	22.73104	1.86E-06	0.001829	C11orf82	chromosome 11 open reading frame 82 [Source:HGNC Symbol;Acc:26351]
ENSG00000177494	-1.97682	3.27748	21.76961	3.07E-06	0.002761	ZBED2	zinc finger, BED-type containing 2 [Source:HGNC Symbol;Acc:20710]
ENSG00000279312	-1.89422	3.25250	21.19005	4.16E-06	0.003439	NA	NA
ENSG00000111863	-1.83930	3.23586	20.86834	4.92E-06	0.003864	ADTRP	androgen-dependent TFPI-regulating protein [Source:HGNC Symbol;Acc:21214]
ENSG00000102886	-1.89367	1.33559	19.13650	1.22E-05	0.006178	GDPD3	glycerophosphodiester phosphodiesterase domain containing 3 [Source:HGNC Symbol;Acc:28638]
ENSG00000273877	-1.73812	1.45175	18.83734	1.42E-05	0.006178	NA	NA
ENSG00000233836	-1.24166	3.05035	19.08904	1.25E-05	0.006178	RP11-255H23.2	
ENSG00000168661	-1.50084	1.53976	18.26336	1.92E-05	0.007554	ZNF30	zinc finger protein 30 [Source:HGNC Symbol;Acc:13090]
ENSG00000165140	-1.41840	6.79165	17.72047	2.56E-05	0.008551	FBP1	fructose-1,6-bisphosphatase 1 [Source:HGNC Symbol;Acc:3606]
ENSG00000197933	-1.40929	1.98489	17.67734	2.62E-05	0.008565	ZNF823	zinc finger protein 823 [Source:HGNC Symbol;Acc:30936]
ENSG00000267530	-1.78582	2.05237	17.46581	2.93E-05	0.008951	AC006273.5	
ENSG00000109084	-1.12343	4.54093	17.26167	3.26E-05	0.009302	TMEM97	transmembrane protein 97 [Source:HGNC Symbol;Acc:28106]
ENSG00000101280	-2.35348	2.19223	16.52967	4.79E-05	0.010282	ANGPT4	angiopoietin 4 [Source:HGNC Symbol;Acc:487]
ENSG00000166928	-2.00636	4.29593	16.66520	4.46E-05	0.010282	MS4A14	membrane-spanning 4-domains, subfamily A, member 14 [Source:HGNC Symbol;Acc:30706]
ENSG00000154309	-1.55690	4.98686	16.68774	4.41E-05	0.010282	DISP1	dispatched homolog 1 (Drosophila) [Source:HGNC Symbol;Acc:19711]
ENSG00000186074	-1.47624	4.09755	16.61976	4.57E-05	0.010282	CD300LF	CD300 molecule-like family member f [Source:HGNC Symbol;Acc:29883]
ENSG00000144285	-1.30572	3.92562	16.68210	4.42E-05	0.010282	SCN1A	sodium channel, voltage-gated, type I, alpha subunit [Source:HGNC Symbol;Acc:10585]
ENSG00000197471	-1.28156	6.32352	16.51354	4.83E-05	0.010282	SPN	sialophorin [Source:HGNC Symbol;Acc:11249]
ENSG00000055732	-1.10872	3.95160	16.57976	4.66E-05	0.010282	MCOLN3	mucolipin 3 [Source:HGNC Symbol;Acc:13358]
ENSG00000182010	-1.56050	8.02949	16.47161	4.94E-05	0.010343	RTKN2	rhotekin 2 [Source:HGNC Symbol;Acc:19364]
ENSG00000134216	-1.68643	2.64706	15.93909	6.54E-05	0.012127	CHIA	chitinase, acidic [Source:HGNC Symbol;Acc:17432]
ENSG00000163154	-1.63822	1.79222	15.87453	6.77E-05	0.012127	TNFAIP8L2	tumor necrosis factor, alpha-induced protein 8-like 2 [Source:HGNC Symbol;Acc:26277]
ENSG00000038945	-1.54726	7.30109	15.41974	8.61E-05	0.013940	MSR1	macrophage scavenger receptor 1 [Source:HGNC Symbol;Acc:7376]
ENSG00000279289	-1.53363	2.18354	15.26661	9.34E-05	0.014376	NA	NA
ENSG00000196268	-1.04599	4.72817	15.30275	9.16E-05	0.014376	ZNF493	zinc finger protein 493 [Source:HGNC Symbol;Acc:23708]
ENSG00000206557	-2.53617	2.02738	15.01206	0.000107	0.015683	TRIM71	tripartite motif containing 71, E3 ubiquitin protein ligase [Source:HGNC Symbol;Acc:32669]
ENSG00000224215	-1.97496	1.67573	15.02507	0.000106	0.015683	RP11-371A19.2	
ENSG00000184635	-1.29200	1.84297	15.04015	0.000105	0.015683	ZNF93	zinc finger protein 93 [Source:HGNC Symbol;Acc:13169]
ENSG00000261786	-1.43300	3.03675	14.84921	0.000116	0.015985	RP4-555D20.2	
ENSG00000167588	-1.70160	4.36066	14.77489	0.000121	0.016126	GPD1	glycerol-3-phosphate dehydrogenase 1 (soluble) [Source:HGNC Symbol;Acc:4455]
ENSG00000268518	-2.00011	0.60830	14.65128	0.000129	0.016501	CTD-2545M3.8	
ENSG00000187808	-2.07373	0.82422	14.48452	0.000141	0.017342	SOWAHD	sosondowah ankyrin repeat domain family member D [Source:HGNC Symbol;Acc:32960]
ENSG00000143167	-2.44864	3.02947	14.43688	0.000145	0.017648	GPA33	glycoprotein A33 (transmembrane) [Source:HGNC Symbol;Acc:4445]
ENSG00000185614	-1.86753	0.62686	14.34695	0.000152	0.017773	FAM212A	family with sequence similarity 212, member A [Source:HGNC Symbol;Acc:32480]
ENSG00000155465	-1.12167	4.92244	14.33824	0.000153	0.017773	SLC7A7	solute carrier family 7 (amino acid transporter light chain, y+L system), member 7 [Source:HGNC Symbol;Acc:11065]
ENSG00000279425	-1.59897	1.49439	14.26448	0.000159	0.017949	NA	NA
ENSG00000019169	-1.73063	7.78359	14.22603	0.000162	0.018189	MARCO	macrophage receptor with collagenous structure [Source:HGNC Symbol;Acc:6895]
ENSG00000233038	-1.51340	1.97987	14.20414	0.000164	0.018271	AC011899.9	
ENSG00000143365	-1.50999	3.41918	14.11790	0.000172	0.018933	RORC	RAR-related orphan receptor C [Source:HGNC Symbol;Acc:10260]
ENSG00000133246	-1.51073	4.02600	14.03662	0.000179	0.019557	PRAM1	PML-RARA regulated adaptor molecule 1 [Source:HGNC Symbol;Acc:30091]
ENSG00000183647	-1.38241	1.20807	14.01903	0.000181	0.019605	ZNF530	zinc finger protein 530 [Source:HGNC Symbol;Acc:29297]
ENSG00000164120	-1.32211	6.84085	13.97325	0.000185	0.019950	HPGD	hydroxyprostaglandin dehydrogenase 15-(NAD) [Source:HGNC Symbol;Acc:5154]
ENSG00000170909	-1.54860	4.04146	13.87146	0.000196	0.020776	OSCAR	osteoclast associated, immunoglobulin-like receptor [Source:HGNC Symbol;Acc:29960]
ENSG00000279518	-1.77727	1.75584	13.79118	0.000204	0.021079	NA	NA
ENSG00000198574	-1.38575	1.31642	13.82165	0.000201	0.021079	SH2D1B	SH2 domain containing 1B [Source:HGNC Symbol;Acc:30416]
ENSG00000143355	-2.54493	2.06730	13.72690	0.000211	0.021151	LHX9	LIM homeobox 9 [Source:HGNC Symbol;Acc:14222]
ENSG00000139292	-1.61875	1.64914	13.65609	0.000220	0.021688	LGR5	leucine-rich repeat containing G protein-coupled receptor 5 [Source:HGNC Symbol;Acc:4504]
ENSG00000197044	-1.06663	3.96501	13.57234	0.000230	0.022267	ZNF441	zinc finger protein 441 [Source:HGNC Symbol;Acc:20875]
ENSG00000159640	-1.26635	5.80508	13.50399	0.000238	0.022484	ACE	angiotensin I converting enzyme [Source:HGNC Symbol;Acc:2707]
ENSG00000186326	-1.90941	0.34521	13.44008	0.000246	0.022504	RGS9BP	regulator of G protein signaling 9 binding protein [Source:HGNC Symbol;Acc:30304]
ENSG00000166347	-1.07042	7.35009	13.34534	0.000259	0.022924	CYB5A	cytochrome b5 type A (microsomal) [Source:HGNC Symbol;Acc:2570]
ENSG00000136352	-1.06764	6.89094	13.37088	0.000256	0.022924	NKX2-1	NK2 homeobox 1 [Source:HGNC Symbol;Acc:11825]
ENSG00000162704	-0.71457	7.06887	13.24224	0.000274	0.023886	ARPC5	actin related protein 2/3 complex, subunit 5, 16kDa [Source:HGNC Symbol;Acc:708]
ENSG00000169442	-1.19106	6.22658	13.18378	0.000282	0.024107	CD52	CD52 molecule [Source:HGNC Symbol;Acc:1804]
ENSG00000196154	-1.14745	7.16932	13.14696	0.000288	0.024321	S100A4	S100 calcium binding protein A4 [Source:HGNC Symbol;Acc:10494]
ENSG00000137819	-1.00375	4.17572	13.07666	0.000299	0.024982	PAQR5	progestin and adipoQ receptor family member V [Source:HGNC Symbol;Acc:29645]
ENSG00000104880	-1.40721	1.25702	12.64312	0.000377	0.029458	ARHGEF18	Rho/Rac guanine nucleotide exchange factor (GEF) 18 [Source:HGNC Symbol;Acc:17090]
ENSG00000236333	-1.44039	1.78336	12.59270	0.000387	0.030114	TRHDE-AS1	TRHDE antisense RNA 1 [Source:HGNC Symbol;Acc:27471]
ENSG00000140600	-1.54488	1.76138	12.51154	0.000404	0.030726	SH3GL3	SH3-domain GRB2-like 3 [Source:HGNC Symbol;Acc:10832]
ENSG00000164048	-1.29416	4.41419	12.48971	0.000409	0.030755	ZNF589	zinc finger protein 589 [Source:HGNC Symbol;Acc:16747]
ENSG00000091106	-1.67865	2.83262	12.40395	0.000428	0.031595	NLRC4	NLR family, CARD domain containing 4 [Source:HGNC Symbol;Acc:16412]
ENSG00000170396	-1.47702	1.28848	12.35579	0.000440	0.032119	ZNF804A	zinc finger protein 804A [Source:HGNC Symbol;Acc:21711]
ENSG00000032444	-1.06178	5.32809	12.32612	0.000447	0.032333	PNPLA6	patatin-like phospholipase domain containing 6 [Source:HGNC Symbol;Acc:16268]
ENSG00000134962	-1.41792	2.66826	12.22001	0.000473	0.033497	KLB	klotho beta [Source:HGNC Symbol;Acc:15527]
ENSG00000086300	-1.19473	5.37061	12.21757	0.000473	0.033497	SNX10	sorting nexin 10 [Source:HGNC Symbol;Acc:14974]
ENSG00000261685	-1.23402	4.80874	12.19197	0.000480	0.033808	RP11-401P9.4	
ENSG00000135929	-1.05054	6.17623	12.18220	0.000482	0.033834	CYP27A1	cytochrome P450, family 27, subfamily A, polypeptide 1 [Source:HGNC Symbol;Acc:2605]
ENSG00000234616	-1.03820	3.28341	12.11818	0.000499	0.034860	JRK	jerky homolog (mouse) [Source:HGNC Symbol;Acc:6199]
ENSG00000197124	-1.07233	2.65758	12.01768	0.000527	0.035835	ZNF682	zinc finger protein 682 [Source:HGNC Symbol;Acc:28857]
ENSG00000100078	-1.55672	0.91795	11.99306	0.000534	0.036008	PLA2G3	phospholipase A2, group III [Source:HGNC Symbol;Acc:17934]
ENSG00000099194	-1.18796	8.85737	11.90836	0.000559	0.036913	SCD	stearoyl-CoA desaturase (delta-9-desaturase) [Source:HGNC Symbol;Acc:10571]
ENSG00000274349	-1.07794	2.50834	11.91038	0.000558	0.036913	NA	NA
ENSG00000171291	-1.02423	3.32195	11.90682	0.000559	0.036913	ZNF439	zinc finger protein 439 [Source:HGNC Symbol;Acc:20873]
ENSG00000137872	-1.19177	5.71449	11.85924	0.000574	0.037397	SEMA6D	sema domain, transmembrane domain (TM), and cytoplasmic domain, (semaphorin) 6D [Source:HGNC Symbol;Acc:16770]
ENSG00000137976	-1.88449	1.88299	11.78792	0.000596	0.038537	DNASE2B	deoxyribonuclease II beta [Source:HGNC Symbol;Acc:28875]
ENSG00000276409	-1.98823	1.35850	11.71362	0.000620	0.038833	NA	NA
ENSG00000146166	-1.93876	0.93318	11.74996	0.000608	0.038833	LGSN	lengsin, lens protein with glutamine synthetase domain [Source:HGNC Symbol;Acc:21016]
ENSG00000111144	-1.11123	8.16197	11.72359	0.000617	0.038833	LTA4H	leukotriene A4 hydrolase [Source:HGNC Symbol;Acc:6710]
ENSG00000115648	-0.90705	6.99280	11.69131	0.000628	0.039104	MLPH	melanophilin [Source:HGNC Symbol;Acc:29643]
ENSG00000204420	-1.33403	2.10145	11.55626	0.000675	0.040399	C6orf25	chromosome 6 open reading frame 25 [Source:HGNC Symbol;Acc:13937]
ENSG00000083814	-1.05999	2.14547	11.58014	0.000667	0.040399	ZNF671	zinc finger protein 671 [Source:HGNC Symbol;Acc:26279]
ENSG00000280399	-1.04645	3.51487	11.52535	0.000687	0.040695	NA	NA
ENSG00000130598	-1.66960	1.67328	11.37485	0.000744	0.043472	TNNI2	troponin I type 2 (skeletal, fast) [Source:HGNC Symbol;Acc:11946]
ENSG00000242640	-1.27400	2.09154	11.37487	0.000744	0.043472	RP11-302F12.1	
ENSG00000260361	-1.41449	1.57306	11.36577	0.000748	0.043523	CTD-2313J17.5	
ENSG00000128268	-1.03122	4.10459	11.25277	0.000795	0.044933	MGAT3	mannosyl (beta-1,4-)-glycoprotein beta-1,4-N-acetylglucosaminyltransferase [Source:HGNC Symbol;Acc:7046]
ENSG00000143971	-0.87887	4.52574	11.24570	0.000798	0.044933	ETAA1	Ewing tumor-associated antigen 1 [Source:HGNC Symbol;Acc:24648]
ENSG00000184730	-1.20820	4.80847	11.23012	0.000805	0.044989	APOBR	apolipoprotein B receptor [Source:HGNC Symbol;Acc:24087]
ENSG00000169905	-0.64610	6.29022	11.20608	0.000815	0.045414	TOR1AIP2	torsin A interacting protein 2 [Source:HGNC Symbol;Acc:24055]
ENSG00000271971	-1.34560	1.21604	11.19417	0.000821	0.045545	CTD-2006H14.2	
ENSG00000247373	-1.51065	1.43984	11.18336	0.000825	0.045550	RP11-486O12.2	
ENSG00000152213	-1.41694	2.39502	11.15503	0.000838	0.045572	ARL11	ADP-ribosylation factor-like 11 [Source:HGNC Symbol;Acc:24046]
ENSG00000108846	-1.00965	4.82562	11.14213	0.000844	0.045572	ABCC3	ATP-binding cassette, sub-family C (CFTR/MRP), member 3 [Source:HGNC Symbol;Acc:54]
ENSG00000115419	-0.67350	6.69722	11.14136	0.000844	0.045572	GLS	glutaminase [Source:HGNC Symbol;Acc:4331]
ENSG00000152804	-1.30047	3.72252	11.04738	0.000888	0.046623	HHEX	hematopoietically expressed homeobox [Source:HGNC Symbol;Acc:4901]
ENSG00000165186	-1.25021	2.29810	11.06390	0.000880	0.046623	PTCHD1	patched domain containing 1 [Source:HGNC Symbol;Acc:26392]
ENSG00000257550	-1.31238	3.16157	10.98752	0.000917	0.046890	RP11-793H13.3	
ENSG00000278768	-1.21999	2.02787	10.99328	0.000914	0.046890	NA	NA
ENSG00000175318	-1.10098	4.50847	10.99083	0.000916	0.046890	GRAMD2	GRAM domain containing 2 [Source:HGNC Symbol;Acc:27287]
ENSG00000198189	-0.93453	5.68593	10.99268	0.000915	0.046890	HSD17B11	hydroxysteroid (17-beta) dehydrogenase 11 [Source:HGNC Symbol;Acc:22960]
ENSG00000139055	-0.90260	4.42255	11.01336	0.000905	0.046890	ERP27	endoplasmic reticulum protein 27 [Source:HGNC Symbol;Acc:26495]
ENSG00000146469	-1.43259	1.20218	10.93073	0.000946	0.046895	VIP	vasoactive intestinal peptide [Source:HGNC Symbol;Acc:12693]
ENSG00000274536	-1.39444	1.91759	10.92963	0.000946	0.046895	NA	NA
ENSG00000165168	-1.27774	7.99215	10.95607	0.000933	0.046895	CYBB	cytochrome b-245, beta polypeptide [Source:HGNC Symbol;Acc:2578]
ENSG00000261136	-1.62425	0.49870	10.88676	0.000969	0.047006	RP11-37C7.3	
ENSG00000111181	-1.58357	0.95979	10.91865	0.000952	0.047006	SLC6A12	solute carrier family 6 (neurotransmitter transporter), member 12 [Source:HGNC Symbol;Acc:11045]
ENSG00000166471	-0.74477	5.13569	10.89168	0.000966	0.047006	TMEM41B	transmembrane protein 41B [Source:HGNC Symbol;Acc:28948]
ENSG00000186715	-1.55221	2.25450	10.81222	0.001008	0.047854	MST1L	macrophage stimulating 1-like [Source:HGNC Symbol;Acc:7390]
ENSG00000177125	-0.86623	3.57258	10.81372	0.001008	0.047854	ZBTB34	zinc finger and BTB domain containing 34 [Source:HGNC Symbol;Acc:31446]
ENSG00000168631	-1.33349	6.68533	10.80348	0.001013	0.047933	DPCR1	diffuse panbronchiolitis critical region 1 [Source:HGNC Symbol;Acc:21666]

Brandsma C-A, *et al. Thorax* 2020; 75:180–183. doi: 10.1136/thoraxjnl-2019-213200

DE mRNA comb FDR 0.05 down

Ensembl gene	logFC	logCPM	LR	PValue	adj.p	gene	Description
ENSG00000164683	-1.54699	4.37894	10.78562	0.001023	0.048224	HEY1	hes-related family bHLH transcription factor with YRPW motif 1 [Source:HGNC Symbol;Acc:4880]
ENSG00000280048	-1.90525	0.28699	10.74691	0.001045	0.048622	NA	NA
ENSG00000130544	-0.99904	3.42857	10.71347	0.001064	0.048850	ZNF557	zinc finger protein 557 [Source:HGNC Symbol;Acc:28632]
ENSG00000150337	-1.13465	3.54856	10.69821	0.001072	0.048968	FCGR1A	Fc fragment of IgG, high affinity Ia, receptor (CD64) [Source:HGNC Symbol;Acc:3613]
ENSG00000256229	-0.94205	2.66936	10.70280	0.001070	0.048968	ZNF486	zinc finger protein 486 [Source:HGNC Symbol;Acc:20807]
ENSG00000273033	-0.82338	4.25747	10.69044	0.001077	0.049032	RP11-67L2.2	
ENSG00000262222	-1.38648	1.06365	10.66043	0.001095	0.049690	RP11-876N24.4	
ENSG00000174837	-1.52189	2.74022	10.63087	0.001112	0.049913	EMR1	egf-like module containing, mucin-like, hormone receptor-like 1 [Source:HGNC Symbol;Acc:3336]

Brandsma C-A, *et al. Thorax* 2020; 75:180–183. doi: 10.1136/thoraxjnl-2019-213200
